# An efficient and practical entry to 2-amido-dienes and 3-amido-trienes from allenamides through stereoselective 1,3-hydrogen shifts

**DOI:** 10.3762/bjoc.7.53

**Published:** 2011-04-07

**Authors:** Ryuji Hayashi, John B Feltenberger, Andrew G Lohse, Mary C Walton, Richard P Hsung

**Affiliations:** 1 Department of Chemistry and Division of Pharmaceutical Sciences, University of Wisconsin, Madison, WI 53705

**Keywords:** allenamides, 2-amido-dienes, 3-amido-trienes, electrocyclic ring-closure, 1,3-hydrogen shift, isomerization

## Abstract

Preparations of de novo acyclic 2-amido-dienes and 3-amido-trienes through 1,3-hydrogen shifts from allenamides are described. These 1,3-hydrogen shifts could be achieved thermally or they could be promoted by the use of Brønsted acids. Under either condition, these processes are highly regioselective in favour of the α-position, and highly stereoselective in favour of the *E-*configuration. In addition, 6π-electron electrocyclic ring-closure could be carried out with 3-amido-trienes to afford cyclic 2-amido-dienes, and such electrocyclic ring-closure could be rendered in tandem with the 1,3-hydrogen shift.

## Introduction

**Scheme 1:**
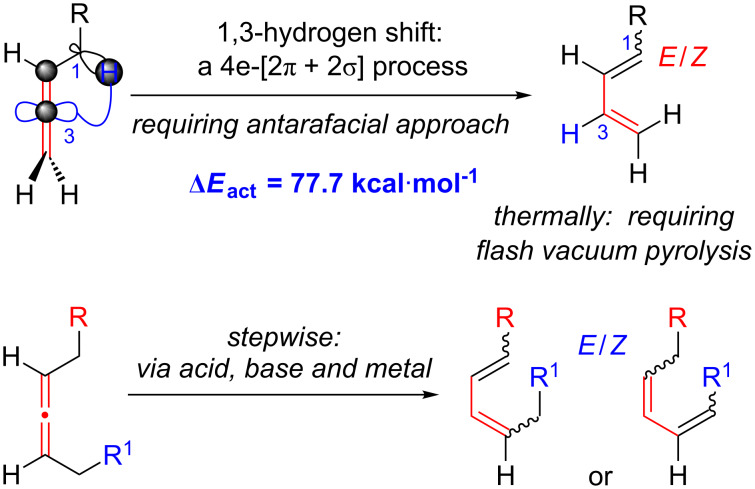
1,3-Hydrogen shifts of allenes.

While allene isomerization to afford conjugated dienes is a well-known and thermodynamically favourable process, it is not trivial kinetically. A concerted allene isomerization leading to a diene involves a 1,3-hydrogen shift, which constitutes a four-electron (2π + 2σ) process that needs an antarafacial approach to fulfil the anti-Hückel (or Möbius) transition state based upon the Woodward–Hoffman rules [1]. Although there is no experimental precedent in an actual allylic system, it is relatively more feasible for an allenic system due to the presence of orthogonally oriented p-orbitals of the sp-hybridized central allenic carbon (Scheme 1), allowing a formal phase change required for an anti-Hückel transition state (in blue, for references on a possible radical pathway, see [2]), and a six-electron (2π + 2σ + 2π) process when considering the possible involvement of the second set of allenic π-electrons. Nevertheless, the calculated ∆*E*_act_ value remains high at 77.7 kcal·mol^−1^ [2]. Whether concerted or not, most thermal isomerizations of allenes require severe reaction conditions (for general reviews on allenes see [3], for some examples of thermal isomerization of exocyclic allenes to dienes via radical intermediates see [4–12]), whereby controlling *E*/*Z* ratios of the resulting diene remains a difficult problem. On the other hand, a stepwise isomerization of allenes via acid-, base-, or metal-mediated conditions seem to be more practical, but known examples have issues in controlling stereo- and regioselectivity [3] (for some examples see [13–20]). Therefore, solving these problems can be highly significant.

**Scheme 2:**
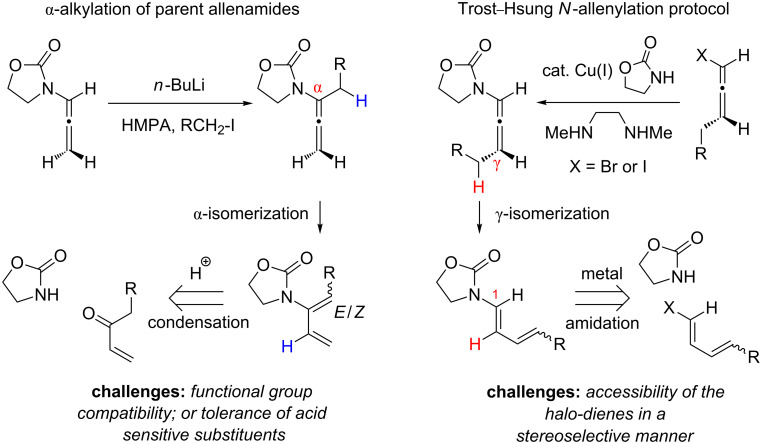
Synthesizing amido-dienes from allenamides.

Because of the popularity of dienes as one of the most utilized organic building blocks, a number of stereoselective preparations are known. The major question here is how viable is it to access conjugated dienes from structurally more challenging allenes through a kinetically difficult and stereochemically undistinguished isomerization. It might not seem like a logical approach; however, our justification is that since there are few well established routes for preparing amido-dienes, our allenamide isomerization strategy (for reviews on allenamide chemistry see [21–23], for reports in 2009, 2010 and 2011 see [24–43], for earlier studies on allenamides see [44–46]) can open the door to construct synthetically useful amido-dienes (for a review on the synthesis of enamides see [47], for reviews on the chemistry of dienamides see [48–50], for reviews on the chemistry of 2-amino or 2-amido-dienes see [51–52]). Problems with the two primary approaches to access amido-dienes [47] are that acid-mediated condensations suffer from functional group tolerances, and metal-mediated coupling methods (for reviews on Cu-mediated C–N and C–O bond formations see [53–55], for some examples see [56–58]) suffer from limited access as well as the instability of halo-dienes (Scheme 2).

**Scheme 3:**
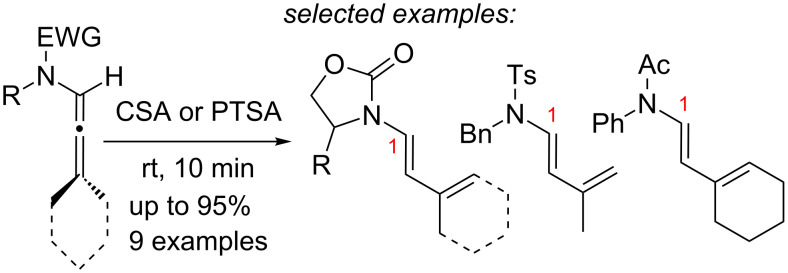
Synthesis of 1-amido-dienes from allenamides.

In contrast, multi-substituted allenamides can be concisely prepared through α-alkylations of a parent allenamide [47,59] (for the synthesis of parent allenamides see [60]) or amidative cross-couplings of allenyl halides [61–62]. Therefore, our allenamide isomerization strategy has a much greater synthetic potential in constructing amido-dienes. While the chemistry of 1-amido-dienes has been explored in some detail (see Scheme 3 for success in preparing 1-amido-dienes via allenamide isomerizations) [63–64] (for examples see [65–72]), herein, we report details of an efficient entry to synthetically rare 2-amido-dienes [73–77] via a regio- and stereoselective 1,3-hydrogen shift of allenamides.

## Results and Discussion

As part of our initial screening efforts, both the thermal and acidic conditions were investigated as shown in Table 1. Allenamide **1** smoothly underwent isomerization via a 1,3-hydrogen shift when heated at 115 °C in CH_3_CN (sealed tube) to give the desired 2-amido-diene product **2** in 78% isolated yield with a 16:1 *E*/*Z* selectivity (Table 1, entry 1). There appears to be some solvent effect on the *E*/*Z* selectivity with more polar solvents providing the best ratio (Table 1, entries 2–4). In addition to thermal conditions, we screened several Brønsted acids at room temperature in order to investigate a milder condition. While PTSA resulted in poor *E*/*Z* ratio (Table 1, entry 5), a range of Brønsted acids were quite effective in affording the desired 2-amido-diene **2** (Supporting Information File 1 and Supporting Information File 2) with excellent *E*/*Z* selectivity [Table 1, entries 6–9].

**Table 1:** 1,3-Hydrogen shift of allenamides.

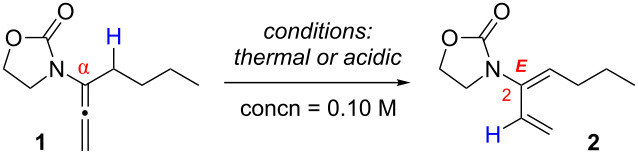						

entry	solvent	acid [10 mol %]	temp [°C]	time [h]	yield [%]^a,b^	*E*:*Z*^c^

1	CH_3_CN	—	115	16	91 (78)	16:1
2	THF	—	115	16	51	9:1
3	ClCH_2_CH_2_Cl	—	115	16	79	7:1
4	toluene	—	150	16	55	4:1
5	CH_2_Cl_2_	*p*-toluenesulfonic acid (PTSA)	25	1	66	2:1
6	CH_2_Cl_2_	*p*-NO_2_-ArCO_2_H	25	16	81	15:1
7	CH_2_Cl_2_	PhCO_2_H	25	16	85 (55)	18:1
8	CH_2_Cl_2_	pyridinium *p*-toluenesulfonate (PPTS)	25	16	77	15:1
9	CH_2_Cl_2_	camphorsulfonic acid (CSA)	25	10 min	95 (74)	18:1

^a^NMR yields. ^b^Isolated yields are shown in brackets. ^c^Ratios were determined by ^1^H NMR.

**Figure 1:**
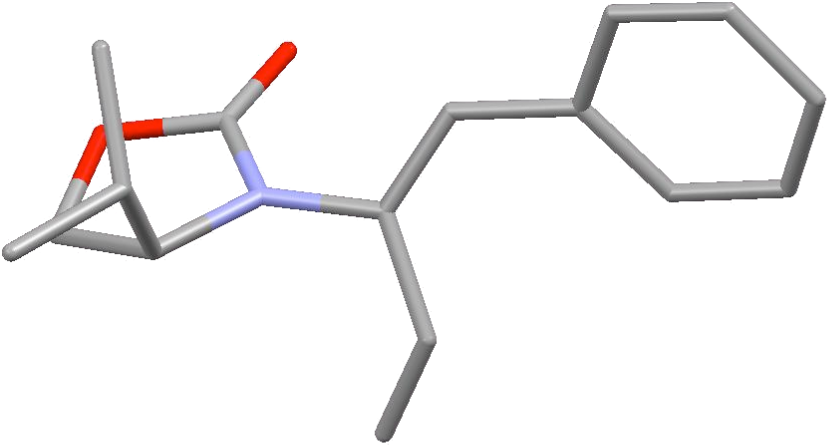
X-ray Structure of **10b**.

After having established the 1,3-hydrogen shift under thermal and protic conditions, a diverse array of 2-amido-dienes was prepared as summarized in Table 2. Some notable features are: (1) a variety of novel chiral 2-amido-dienes **8**–**10** were obtained from chiral allenamides **5**–**7** in synthetically useful yields and with high *E*/*Z* ratios (≥95:5) under both thermal or acidic conditions (Table 2, entries 2–12); (2) unsubstituted 2-amido-dienes **8d** and **9c** could also be prepared in good yields (see R = H in Table 2, entries 7 and 10); (3) even allenamide containing an acyclic carbamate such as **11** underwent an efficient 1,3-hydrogen shift; and (4) the X-ray structure (Supporting Information File 3) of a single crystal of 2-amido-diene **10b** was successfully obtained to assign unambiguously the *E*-configuration (Figure 1).

**Table 2:** Synthesis of 2-amido-dienes.

entry	allenamides		conditions (time)^a^	amido-dienes		yield [%]^b,c^

1	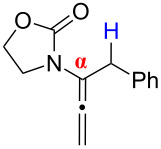	**3**	115 °C (16 h)	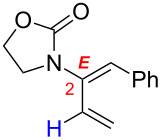	**4**	71

2	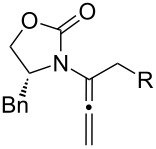	**5a:** R = *n*-Pr	115 °C (6 h)	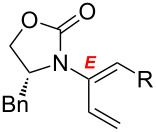	**8a**	77
3		**5a:** R = *n*-Pr	CSA (4 h)^d^		**8a**	87
4		**5b:** R = Ph	115 °C (16 h)		**8b**	74
5		**5b:** R = Ph	CSA (2 h)		**8b**	83
6		**5a:** R = ^2^Nap^e^	115 °C (16 h)^f^		**8c**	73
7		**5a:** R = H	115 °C (16 h)		**8d**	69

8	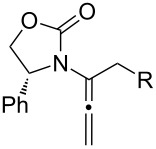	**6a:** R = *n*-Pr	CSA (10 min)	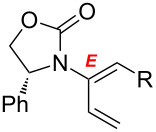	**9a**	82
9		**6b:** R = Ph	CSA (10 min)		**9b**	76
10		**6c:** R = H	115 °C (16 h)		**9c**	69

11	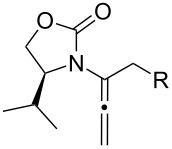	**7a:** R = *n*-Pr	115 °C (16 h)	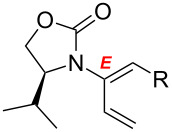	**10a**	62
12		**7b:** R = Ph	115 °C (16 h)		**10b**	82

13	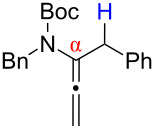	**11**	135 °C (16 h)	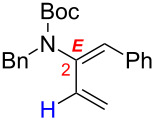	**12**	45
14		**11**	CSA^g^ (2 h)		**12**	61

^a^Unless otherwise indicated, CH_3_CN was the solvent for thermal conditions and CH_2_Cl_2_ was the solvent when using 10 mol % of CSA at rt. For all reactions, concn = 0.10 M. ^b^All are isolated yields. ^c^All 1,3-H shifts were highly *E*-selective [≥95:5] except for entry 1 in which the *E*:*Z* ratio is 6:1 for **4**. Ratios were determined by ^1^H NMR. ^d^Temp started at –78 °C. ^e^The group ^2^Nap stands for 2-naphthyl. ^f^ClCH_2_CH_2_Cl was used. ^g^4Å MS was used.

Encouraged by this highly stereoselective isomerization, we turned our attention to the possibility of constructing synthetically much more challenging 3-amido-trienes from allenamides through 1,3-hydrogen shifts. As shown in Table 3, to our satisfaction, a wide variety of 3-amido-trienes could be readily accessed from corresponding α-allylated allenamides. When using a catalytic amount of CSA, both achiral and chiral 3-amido-trienes were obtained in high yields with exclusive *E-*selectivity, including structurally intriguing examples such as **24**–**28** (Table 3, entries 9–15). Moreover, a protected alcohol or amine in the allenamide did not impede the isomerization process (Table 3, entries 10–14), leading to more functionalized trienes.

**Table 3:** Synthesis of 3-amido-trienes.^a^

entry	α-allylated allenamides		3-amido-trienes		yield [%]^b^

1	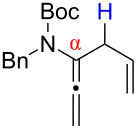	**13**	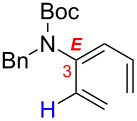	**14**	86

2	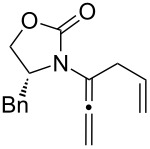	**15a**	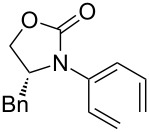	**22**	79

3	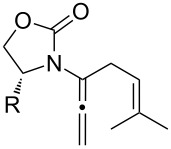	**16a:** R = Bn	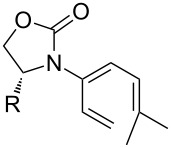	**23a**	89
4		**16b:** R = Ph		**23b**	89
5		**16c:** R = iPr		**23c**	91

6	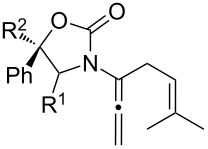	**16d:** R^1^ = (*R*)-Me, R^2^ = H	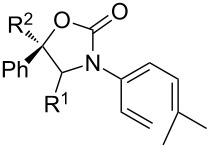	**23d**	74
7		**16e:** R^1^ = (*S*)-Ph, R^2^ = H		**23e**	89
8		**16f:** R^1^ = (*R*)-Ph, R^2^ = Ph		**23f**	86

9	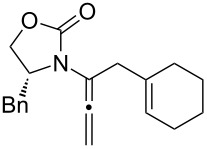	**17**	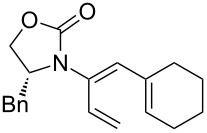	**24**	95

10	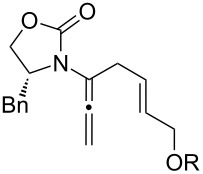	**18a:** R = TBDPS	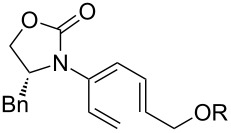	**25a**	84
11		**18b:** R = allyl		**25b**	75
12		**18c:** R = cinnamyl		**25c**	54

13	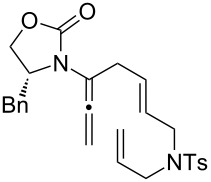	**19**	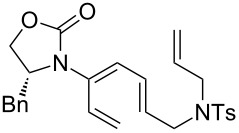	**26**	62

14	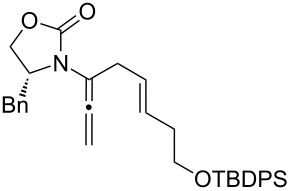	**20**	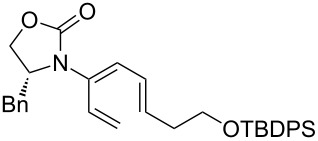	**27**	72

15	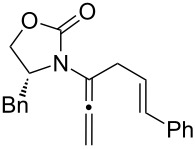	**21**	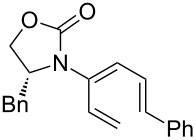	**28**	72

^a^All reactions were run in CH_2_Cl_2_ [concn = 0.10 M] with 10 mol % of CSA for 10 min at rt. ^b^All were isolated yields.

To continue elevating the level of complexity, we examined allenamides with both α- and γ-substitutions and hoped to observe regioselectivity during the 1,3-hydrogen shift. Consequently, as shown in Table 4, isomerizations of tetra-substituted allenamides were examined. When heating α- and γ-substituted allenamides **29a** and **30** in CH_3_CN at 115 °C in a sealed tube, 1,3-hydrogen shift took place exclusively from the α-position affording highly substituted (*E*)*-*2-amido-dienes **33a** and **34** in 71% and 79% yields, respectively (Table 4, entries 1 and 3). The *E-*geometry in **33a** and **34** was assigned by NOE (Supporting Information File 2).

**Table 4:** A regioselective 1,3-hydrogen shift.^a^

entry	allenamides		conditions (time)	amido-alkenes		yield [%]^b,c^

1	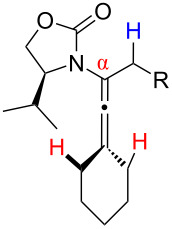	**29a:** R = Ph	115 °C (16 h)	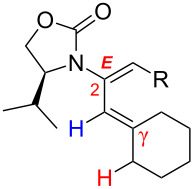	**33a**	71
2		**29b:** R = H	—^d^		**33b**	90

3	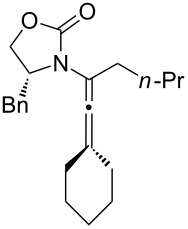	**30**	115 °C (16 h)	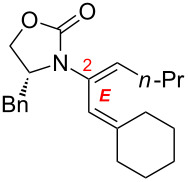	**34**	79

4	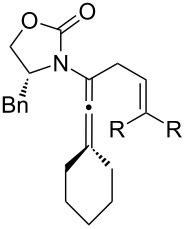	**31a:** R = H	CSA (10 min)	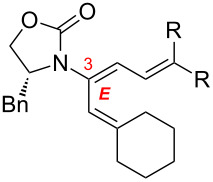	**35a**	68
5		**31b:** R = Me	CSA (10 min)		**35b**	80

6	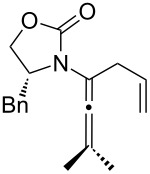	**32**	CSA (10 min)	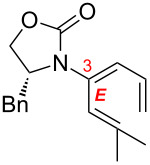	**36**	84

^a^Unless otherwise noted, CH_3_CN was the solvent for thermal conditions and CH_2_Cl_2_ was the solvent when using 10 mol % of CSA at rt. For all reactions, concn = 0.10 M. ^b^All were isolated yields. ^c^All amido-di- and trienes were exclusively *E-*selective [≥95:5]. ^d^See text for this isomerization.

Intriguingly, allenamide **29b** underwent a 1,3-hydrogen shift at room temperature when simply in contact with silica gel during the purification stage; but again, only the 1,3-hydrogen shift was favoured proceeding from the α-position to give (*E*)*-*2-amido-diene **33b** (Table 4, entry 2). In addition, highly substituted 3-amido-trienes **35a**, **35b**, and **36** were regioselectively synthesized in overall high yields using the CSA-catalyzed conditions (Table 4, entries 4–6). Not only are the products from this regioselective isomerization structurally unique, but also mechanistically intriguing.

**Figure 2:**
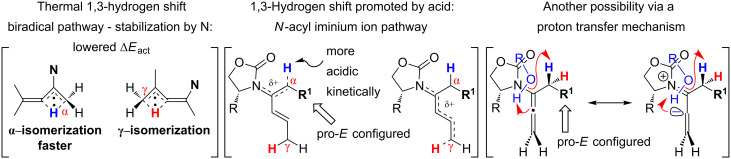
Proposed mechanistic models.

One of the probable explanations for the significantly lowered thermal activation barrier of 1,3-hydrogen-shifts of allenamides is that the nitrogen atom can serve to stabilize the biradical intermediate [2,4–12] (for another leading reference on related radical intermediates see [78]) which are presumed to be electron deficient. Based on the model in Figure 2 (left side), stabilization of the biradical intermediate is direct when isomerizations proceed from the α-position, whereas the isomerization from the γ-position is “vinylogous”, or remotely stabilized through the olefin. Therefore, thermal isomerizations at the α-position should be faster than at the γ-position.

While under thermal conditions, a biradical intermediate is at play [2,4,24], under acidic conditions, the isomerization clearly proceeds through an *N*-acyl iminium intermediate via protonation of the allenamide (Figure 2, center). Consequently, a similar argument could be used to rationalize the regioselective 1,3-hydrogen shift when acid was used. It is noteworthy that this charged transition state could also be adopted for the thermal isomerization. While still being a neutral transition state, the nitrogen atom could facilitate a polarized transition state through increasing negative charge density at the β-carbon. This action would lead to an *N*-acyl iminium ion-like character with the migrating hydrogen being proton-like with the α-position being favoured. This polarized transition state should also have a lower thermal activation barrier for the 1,3-hydrogen shift than the neutral one.

**Scheme 4:**
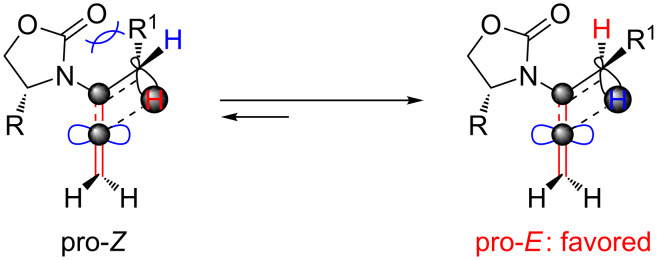
A favored pro-*E* TS.

Lastly, a non-radical proton-transfer like mechanism could also be at play under conditions using protic solvents or owing to the presence of trace of amount of water (Figure 2, right). These last two models also reveal some insight into the *E-*selectivity given the pro-*E* configured transition state (TS) (see the R^1^ group). Along the same line, if the reaction proceeds through a radical pathway, the observed *E*-selectivity in the thermal 1,3-hydrogen shift should be favoured because the pro-*Z* transition state experiences a greater allylic strain compared to the pro-*E* transition state (Scheme 4). A thermodynamically driven equilibration from (*Z*)- to (*E*)-enamide post-isomerization is a real possibility that cannot be ruled out, and the observed solvent effect on the (*E*/*Z*)-selectivity would particularly support this possible notion.

**Scheme 5:**
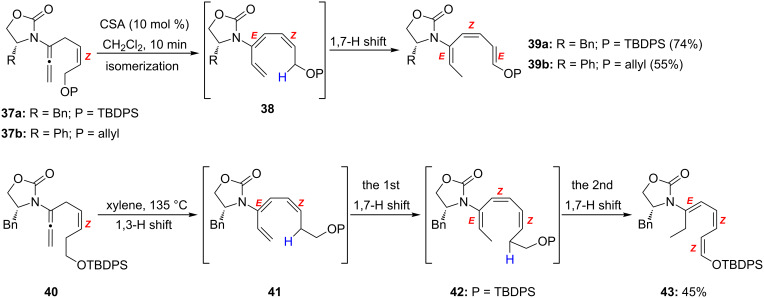
Unexpected competing 1,7-hydrogen shifts.

An interesting discovery was made during this work. As shown in Scheme 5, when subjected to CSA catalyzed isomerization for protected allyl alcohol-substituted allenamides, the reactions with **37a** and **37b** did not stop at the intermediate **38**, but an unexpected 1,7-H-shift (for some examples of an antarafacial 1,7-H shift see [79–82]) took place at room temperature to afford 5-amido-trienes **39a** and **39b** stereoselectively in good yields. Furthermore, when heating the protected homo-allyl alcohol-substituted allenamide **40**, after the 1,3-hydrogen shift an unprecedented double 1,7-H-shift through intermediate **41** and **42** took place to afford the 6-amido-triene **43** in 45% yield. It is noteworthy that amido-triene **41** could be isolated in 65% yield when using 10 mol % CSA.

**Scheme 6:**
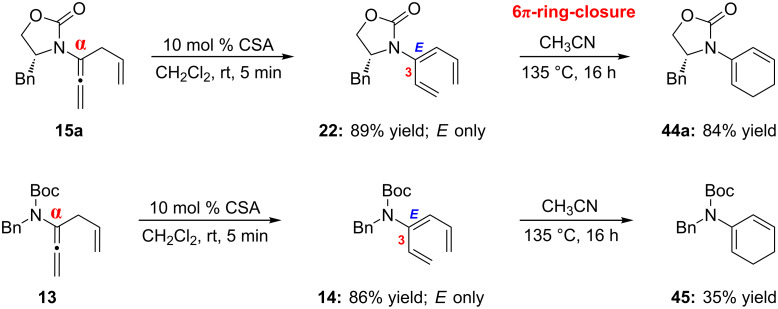
Applications in pericyclic ring-closure.

The synthesis of 3-amido-trienes from α-isomerization of allenamides allowed us to explore an important pericyclic process for yet another amido-diene synthesis. As shown in Scheme 6, isomerizations of α-allylated allenamides **15a** and **13** under acidic conditions can afford 3-amido-trienes **22** and **14** in excellent yields. Given the *E*-selectivity of this isomerization, these 3-amido-trienes are perfectly suited for thermal 6π-electron electrocyclic ring-closure (for reviews on pericyclic ring-closures see [83–84], for reviews on ring-closure in natural product synthesis see [85–86], for recent examples of 6π-electron electrocyclic ring-closure see [87–93], for examples on accelerated ring-closures of 1,3,5-hexatrienes see [94–99]) to access cyclic 2-amido-dienes that are quite rare (for examples see [100–102]). Chiral amido-triene **22** underwent electrocyclization efficiently to give chiral cyclic 2-amido-diene **44a** in 84% yield. Although obtained in only 35% yield, the achiral cyclic 2-amido-diene **45** could also be prepared.

**Scheme 7:**
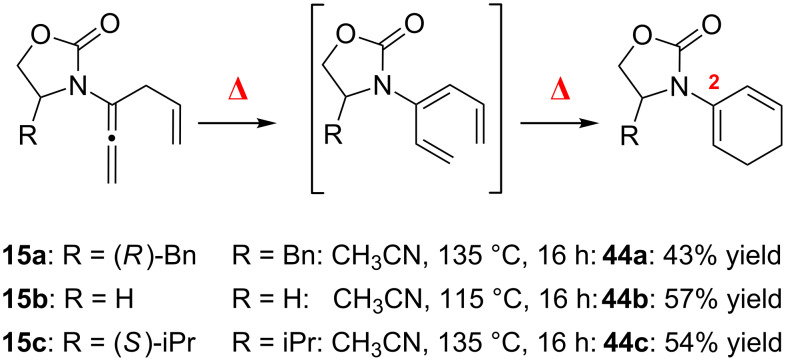
Cyclic 2-amido-diene synthesis.

Finally, this overall process was rendered in tandem under thermal conditions to directly prepare cyclic 2-amido-dienes **44a**–**c** from allenamides **15a**–**c**, respectively, in good yields (Scheme 7). Notably these 6π-electron pericyclic ring-closures took place at relatively low temperature (135 °C), thereby implying an accelerated process of electrocyclization. This feature is consistently observed in related ring-closures of 1,3,5-hexatrienes with an electron-donating substituent at the C3 position of the triene [94–99] (for theoretical studies on substituent effects on electrocyclic ring-closures of 1,3,5-hexatrienes see [97,103–105]. It is also noteworthy that while acyclic 2-amido-dienes and 3-amido-trienes are synthetically challenging to make, cyclic amido-dienes are almost inaccessible synthetically [100–102].

## Conclusion

Herein, we have accomplished the preparation of de novo acyclic 2-amido-dienes and 3-amido-trienes through 1,3-hydrogen shifts from allenamides. These 1,3-hydrogen shifts could be achieved under thermal conditions or they could be promoted with Brønsted acids. Under either condition, these processes are highly regioselective in favour of the α-position, and highly stereoselective in favour of the *E-*configuration. Additionally, 6π-electron electrocyclic ring-closure could be carried out from 3-amido-trienes to afford cyclic 2-amido-dienes, and such electrocyclic ring-closure could be rendered in tandem with the 1,3-hydrogen shift, thereby constituting a facile construction of synthetically rare cyclic 2-amido-dienes.

## Supporting Information

Supporting Information features detailed information on synthesis, purification and characterization data of all substances given in this article, proton and selected carbon NMR spectra, and X-ray data of compound **10b**.

File 1 Experimental section.

File 2 Proton and Carbon NMR spectra, and NOE data.

File 3 X-Ray structural analysis and information for compound **10b**.
