# Supplementary material for: An efficient and practical entry to 2-amido-dienes and 3-amido-trienes from allenamides through stereoselective 1,3-hydrogen shifts
Source: Beilstein J Org Chem. 2011 Apr 7;7:410–20. doi: 10.3762/bjoc.7.53 (PMC3079123; doi:10.3762/bjoc.7.53)
Supplement: File 1 — Experimental section. [file Beilstein_J_Org_Chem-07-410-s001.pdf]

Supporting Information

for

An efficient and practical entry to 2-amido-dienes and 3-amido-trienes from allenamides through stereoselective 1,3-hydrogen shifts.

Ryuji Hayashi, John B. Feltenberger, Andrew G. Lohse, Mary C. Walton and Richard P. Hsung*

Address: Department of Chemistry and Division of Pharmaceutical Sciences, University of Wisconsin, Madison, WI 53705

Email: Richard P. Hsung* - rhsung@wisc.edu

*Corresponding author

Experimental section

General experimental information

All reactions were performed in flame-dried glassware under a nitrogen atmosphere. Solvents were distilled prior to use. Reagents were used as purchased from Aldrich, Acros, Alfa Aesar, or TCI) unless otherwise noted. Chromatographic separations were performed on Silicycle 43-60 Å SiO_2. ^{1}H and ^{13}C NMR spectra were obtained on Varian VI-400 and VI-500 spectrometers in $CDCl_3$ with TMS or residual solvent as internal standards unless otherwise noted. Melting points were determined with a Laboratory Devices MEL-TEMP and are uncorrected/calibrated. Infrared spectra were obtained on Bruker EQUINOX 55 FTIR. TLC analysis was performed using Aldrich 254 nm polyester-backed plates (60 Å, 250 μm) and visualized with UV and $KMnO_4$ stains. Low-resolution mass spectra were obtained using an Agilent 1100 series LS/MSD and are APCI. High-resolution mass spectral analysis performed at University of Wisconsin School of Pharmacy and Department of Chemistry Mass Spectrometry Laboratories. All spectral data obtained for new compounds are reported here.

General procedure 1 for preparations of allenamides via α-alkylations

n-BuLi (1.5 equiv, 2.5 M in hexanes) was added dropwise to a cooled (−78 °C) solution of a given allenamide (1.0 equiv) and HMPA (1.5 equiv) in anhydrous THF (0.1 M). After stirring for 45 min for complete deprotonation, the required halide (1.5 equiv) was added dropwise. The resulting solution was stirred at −78 °C for 1 h and gradually allowed to warm to rt over ~2 h. The solution was washed twice with saturated aq NaCl, dried over Na_2SO_4, and concentrated under reduced pressure. Separation and purification of the resulting crude residue by silica gel flash column chromatography (gradient eluent: EtOAc in hexane) afforded the desired α-substituted allenamides.

See Reference for more details: Xiong, H.; Hsung, R. P.; Wei, L.–L.; Berry, C. R.; Mulder, J. A.; Stockwell, B. *Org. Lett.* **2000,** *2*, 2869.

Please note that allenamides **3, 5c**, **5d**, and **6a–c**, which are in the manuscript but not described here, are published known allenamides. Only new allenamides are described here.

Characterizations of allenamides

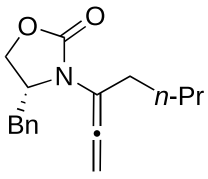

5a

Allenamide **5a** (58.0 mg, 0.20 mmol) was prepared in 39% yield according to general procedure 1.

5a: R_f = 0.42 [25% EtOAc/hexanes]; $[\alpha]_D^{23}$ = -3.48° [c 0.027, benzene]; colorless oil;
^{1}H NMR (400 MHz, $CDCl_3$) δ 0.93 (t, 3H, *J* = 5.6 Hz), 1.22–1.49 (m, 4H), 2.53 (ddd, 2H, *J* = 2.8, 5.2, 8.4 Hz), 2.65 (dd, 1H, *J* = 7.6, 10.8 Hz), 3.24 (dd, 1H, *J* = 3.2, 11.2 Hz), 4.05-4.14 (m, 2H), 4.14 (t, 1H, *J* = 6.4 Hz), 5.24 (dt, 1H, *J* = 2.4, 8.0 Hz), 5.35 (dt, 1H, *J* = 1.6, 8.0 Hz), 7.16 (d, 1H, *J* = 6.8 Hz), 7.26 (d, 2H, *J* = 6.0 Hz), 7.32 (t, 2H, *J* = 5.6 Hz);
^{13}C NMR (100 MHz, $CDCl_3$) δ 14.2, 22.4, 29.4, 29.6, 38.7, 58.0, 66.9, 84.5, 109.6, 127.4, 129.1, 129.3, 135.9, 155.8, 204.3;
IR (neat) cm^{-1} 3035w, 2963w, 1751s, 1479m, 1392w;
mass spectrum (APCI): m/e (% relative intensity) 272.2 (100) $(M+H)^+$.

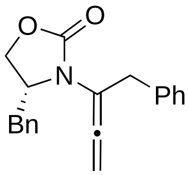

5b

Allenamide **5b** (200.0 mg, 0.66 mmol) was prepared in 66% yield according to general procedure 1.

5b: R_f = 0.33 [25% EtOAc/hexanes]; $[\alpha]_D^{23}$ = -4.33° [c 0.009, benzene]; colorless oil;
^{1}H NMR (400 MHz, $CDCl_3$) δ 2.24 (dd, 1H, *J* = 9.6, 13.6 Hz), 2.99 (dd, 1H, *J* = 3.2, 13.6 Hz), 3.73 (dt, 1H, *J* = 2.0, 15.2 Hz), 3.90-4.13 (m, 3H), 5.22 (ddd, 1H, *J* = 2.0, 3.2, 6.4 Hz), 5.34 (ddd, 1H, *J* = 2.0, 3.2, 6.4 Hz), 7.22-7.32 (m, 10H);
^{13}C NMR (100 MHz, $CDCl_3$) δ 37.0, 38.3, 58.1, 67.1, 84.0, 108.7, 127.0, 127.3, 128.6, 129.0, 129.2, 129.5, 135.9, 138.2, 156.0, 204.9;
IR (neat) cm^{-1} 3035w, 2963w, 1754s, 1478m, 1396w, 1356w;
mass spectrum (APCI): m/e (% relative intensity) 306.2 (100) $(M+H)^+$.

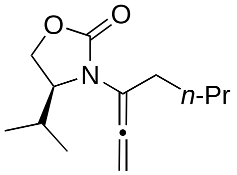

7a

Allenamide **7a** (68.0 mg, 0.28 mmol) was prepared in 27% yield according to general procedure 1.

7a: R_f = 0.30 [25% EtOAc/hexanes]; $[\alpha]_D^{23}$ = +52.4° [c 0.012, benzene]; colorless oil;
^{1}H NMR (500 MHz, $CDCl_3$) δ 0.87-0.94 (m, 9H), 1.25-1.48 (m, 4H), 2.08-2.17 (m, 1H), 2.37-2.51 (m, 2H), 3.86 (ddd, 1H, *J* = 4.0, 6.0, 9.5 Hz), 4.10 (dd, 1H, *J* = 6.0, 9.0 Hz), 4.26 (t, 1H, *J* = 9.0 Hz), 5.12 (dt, 1H, *J* = 3.5, 10.0 Hz), 5.24 (dt, 1H, *J* = 3.5, 10.0 Hz);
^{13}C NMR (125 MHz, $CDCl_3$) δ 14.1, 14.7, 18.0, 22.5, 28.3, 29.3, 29.6, 60.7, 63.1, 83.9, 108.9, 159.4, 204.8;
IR (neat) cm^{-1} 2961w, 2874w, 1750s, 1701m, 1480w, 1466w;
mass spectrum (APCI): m/e (% relative intensity) 224.1 (100) $(M+H)^+$.

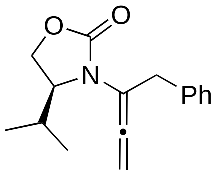

7b

Allenamide **7b** (71.0 mg, 0.28 mmol) was prepared in 28% yield according to general procedure 1.

7b: R_f = 0.28 [25% EtOAc/hexanes]; $[\alpha]_D^{23}$ = +73.0 ° [c 0.010, benzene]; colorless oil;
^{1}H NMR (500 MHz, $CDCl_3$) δ 0.56 (d, 3H, *J* = 6.5 Hz), 0.77 (d, 3H, *J* = 7.0 Hz), 1.91 -1.99 (m, 1H), 3.67-3.73 (m, 2H), 3.96 (dt, 1H, *J* = 3.0, 6.5 Hz), 4.00 (dd, 1H, *J* = 5.5, 9.0 Hz), 4.14 (t, 1H, *J* = 9.5 Hz), 5.11 (dt, 1H, *J* = 2.0, 10.5 Hz), 5.25 (dt, 1H, *J* = 2.0, 10.5 Hz), 7.22 (dd, 1H, *J* = 4.0, 8.5 Hz), 7.27 (d, 4H, *J* = 4.5 Hz);
^{13}C NMR (125 MHz, $CDCl_3$) δ 14.1, 18.0, 28.6, 37.0, 60.9, 63.0, 83.7, 108.5, 127.0, 128.6, 129.6, 138.2, 156.5, 205.2;
IR (neat) cm^{-1} 3035w, 2963w, 1751s, 1479m, 1392w;
mass spectrum (APCI): m/e (% relative intensity) 258.2 (100) $(M+H)^+$.

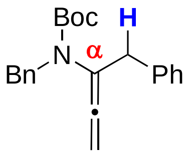

11

Allenamide **11** (114.0 mg, 0.34 mmol) was prepared in 65% yield according to general procedure 1.

11: R_f = 0.38 [9% EtOAc/hexanes]; pale yellow oil;

^{1}H NMR (500 MHz, $CDCl_3$, 55 ^{o}C) δ 1.44 (s, 9H), 3.48 (s, 2H), 4.42 (s, 2H), 4.79 (s, 2H), 7.06 (d, 2H, *J* = 7.0 Hz), 7.13-7.23 (m, 8H);

^{13}C NMR (125 MHz, $CDCl_{3,}$ 55 ^{o}C) δ 28.6, 39.0, 52.9, 80.6, 81.3, 112.5, 126.5, 127.1, 128.3, 128.4, 129.6, 138.3, 138.7, 154.6, 207.5 *(one sp^2 carbon missing due to overlap or line-broadening)*;

IR (thin film) cm^{-1} 2957m, 1698s, 1453m, 1367m, 1150s;

mass spectrum (APCI): m/e (% relative intensity) 236 (100) (M+H-Boc)$^+$.

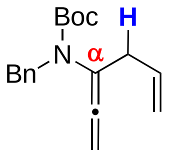

13

Allenamide **13** (214.0 mg, 0.75 mmol) was prepared in 49% yield according to general procedure 1. ^{13}C NMR spectra for the *N*-Boc examples were taken at 50-55 ^{o}C due to broadening and complication of signals caused by rotamers.

13: R_f = 0.43 [9% EtOAc/hexanes]; clear oil;

^{1}H NMR (500 MHz, $CDCl_3$) δ 1.45 (s, 9H), 2.92 (brs, 2H), 4.54 (s, 1H), 4.93-4.95 (m, 2H), 4.96-5.01 (m, 2H), 5.59 (ddt, 1H, *J* = 17.2, 10, 7.2 Hz), 7.23-7.30 (m, 5H);

^{13}C NMR (125 MHz, $CDCl_{3,}$ 50 ^{o}C) δ 28.6, 36.8, 52.9, 80.5, 80.7, 111.4, 116.7, 127.2, 128.2, 128.3, 134.5, 138.8, 154.5, 207.0;

IR (film) cm^{-1} 2978m, 1695s, 1443m, 1382m, 1161s;

mass spectrum (APCI): m/e (% relative intensity) 186 (100) (M+H-Boc)$^+$.

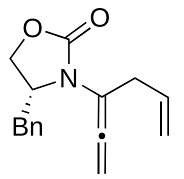

15a

Allenamide **15a** (42.9 mg, 0.37 mmol) was prepared in 37% yield according to general procedure 1.

15a: R_f = 0.25 [25% EtOAc/hexanes]; $[\alpha]_D^{23}$ = +3.85° [c 0.007, benzene]; colorless oil;
^{1}H NMR (500 MHz, $CDCl_3$) δ 2.67 (dd, 1H, *J* = 9.5, 14.0 Hz), 3.22 (dd, 1H, *J* = 3.5, 13.0 Hz), 3.29-3.36 (m, 2H), 4.06 (dd, 1H, *J* = 5.5, 8.5 Hz), 4.11 (ddd, 1H, *J* = 3.0, 7.5, 11.5 Hz), 4.20 (t, 1H, *J* = 8.0 Hz), 5.10 (ddd, 1H, *J* = 1.0, 3.0, 10.0 Hz), 5.20 (dd, 1H, *J* =1.5, 15. Hz), 5.25 (dt, 1H, *J* = 3.0, 10.5 Hz), 5.84 (dq, 1H, *J* = 7.0, 17.0 Hz), 7.15 (d, 2H, *J* = 7.0 Hz), 7.20-7.34 (m, 3H);
^{13}C NMR (125 MHz, $CDCl_3$) δ 34.7, 38.7, 58.1, 67.0, 84.8, 108.1, 117.8, 127.5, 129.2, 129.3, 134.2, 136.0, 204.5;
IR (neat) cm^{-1} 3029w, 2930w, 2363, 1754s, 1604w, 1497m, 1479m, 1454m;
mass spectrum (APCI): m/e (% relative intensity) 256.1 (100) $(M+H)^+$.

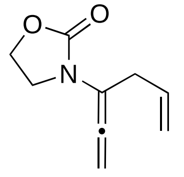

15b

Allenamide **15b** (79.1 mg, 0.63 mmol) was prepared in 63% yield according to general procedure 1.

15b: R_f = 0.45 [40% EtOAc/hexanes]; colorless oil;
^{1}H NMR (400 MHz, $CDCl_3$) δ 3.34-3.37(m, 2H), 3.66 (dd, 2H, *J* = 6.8, 8.0 Hz), 4.35 (dd, 2H, 7.2, 8.0 Hz), 5.07 (ddd, 1H, *J* = 1.2, 2.8, 10.0 Hz), 5.15 (ddd, 1H, *J* = 1.6, 3.2, 16.8 Hz), 5.26 (t, 2H, 3.2 Hz), 5.83 (dq, 1H, *J* = 5.6, 13.6 Hz 1H);
^{13}C NMR (100 MHz, $CDCl_3$) δ 33.9, 46.5, 61.9, 85.3, 109.8, 117.2, 134.4, 155.6, 203.2;
IR (neat) cm^{-1} 2981w, 2919w, 2362w, 1743s, 1641w, 1481m, 1451m, 1401m;
mass spectrum (APCI): m/e (% relative intensity) 166.1 (100) (M+H).

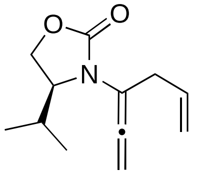

15c

Allenamide **15c** (80.0 mg, 0.39 mmol) was prepared in 39% yield according to general procedure 1.

15c: R_f = 0.32 [25% EtOAc/hexanes]; $[\alpha]_D^{23}$ = +21.1° [c 0.008, benzene]; colorless oil;
^{1}H NMR (500 MHz, $CDCl_3$) δ 0.89 (t, 3H, *J* = 6.5 Hz), 2.10-2.15 (m, 1H), 3.22-3.26 (m, 1H), 3.86 (ddd, 1H, *J* = 4.0, 6.0, 9.5 Hz), 4.11 (dd, 1H, 5.5, 9.0 Hz), 4.26 (t, 1H, *J* = 9.0 Hz), 5.09 (ddd, 1H, *J* = 1.0, 2.5, 7.0 Hz), 5.12-5.18 (m, 2H), 5.81 (dq, 1H, *J* = 7.0, 17.0 Hz);
^{13}C NMR (125 MHz, $CDCl_3$) δ 14.7, 18.0, 28.3, 34.8, 60.8, 63.1, 84.2, 107.4, 117.7, 134.2, 156.4, 204.9;
IR (neat) cm^{-1} 2965m, 2361w, 1744s, 1486m, 1391m, 1324;
mass spectrum (APCI): m/e (% relative intensity) 208.1 (100) (M+H).

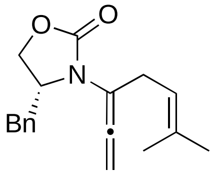

16a

Allenamide **16a** (150.0 mg, 0.53 mmol) was prepared in 53% yield according to general procedure 1.

16a: R_f = 0.31 [25% EtOAc/hexanes]; $[\alpha]_D^{23}$ = -1.67° [c 0.015, benzene]; colorless oil;
^{1}H NMR (500 MHz, $CDCl_3$) δ 1.67 (s, 3H), 1.73 (s, 3H), 2.65 (dd, 1H, *J* = 11.5, 17.0 Hz), 3.17-3.26 (m, 3H), 4.03-4.4.16 (m, 2H), 4.19 (t, 1H, *J* = 9.5 Hz), 5.18-5.23 (m, 2H), 5.33 (dt, 1H, *J* = 4.0, 13.0 Hz), 7.14 (d, 2H, *J* = 8.0 Hz), 7.21-7.34 (m, 3H);
^{13}C NMR (125 MHz, $CDCl_3$) δ 18.3, 25.9, 29.3, 38.6, 58.1, 66.9, 84.3, 108.7, 119.7, 127.4, 129.1, 129.3, 135.0, 136.0, 155.9, 204.6;
IR (neat) cm^{-1} 2969w, 2358w, 1752s, 1701m, 1604w, 1497m, 1478m, 1453m, 1397m;
mass spectrum (APCI): m/e (% relative intensity) 284.1 (100) $(M+H)^+$.

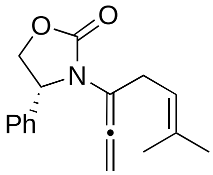

16b

Allenamide **16b** (170.0 mg, 0.63 mmol) was prepared in 42% yield according to general procedure 1.

16b: R_f = 0.35 [25% EtOAc/hexanes]; $[\alpha]_D^{23}$ = -86.6 ° [c 0.007, benzene]; colorless oil;
^{1}H NMR (500 MHz, $CDCl_3$) δ 1.50 (s, 3H), 1.67 (s, 3H), 3.07 (dd, 1H, *J* = 7.0, 16.5 Hz), 3.17 (ddd, 1H, *J* = 3.5, 11.0, 19.5 Hz), 4.12 (t, 1H, *J* = 8.0 Hz), 4.62 (t, 1H, *J* = 9.0 Hz), 4.90-4.94 (m, 2H), 5.00 (ddd, 1H, *J* = 3.0, 10.0, 13.0 Hz), 5.04 (dt, 1H, *J* = 1.5, 8.5 Hz), 7.28-7.39 (m, 5H);
^{13}C NMR (125 MHz, $CDCl_3$) δ 18.1, 25.9, 29.3, 61.6, 70.1, 84.4, 108.5, 119.6, 127.4, 129.0, 129.1, 135.1, 138.6, 156.4, 205.0;
mass spectrum (APCI): m/e (% relative intensity) 293.4 (100) $(M+Na)^+$.

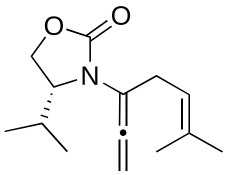

16c

Allenamide **16c** (210.0 mg, 0.89 mmol) was prepared in 60% yield according to general procedure 1.

16c: R_f = 0.43 [25% EtOAc/hexanes]; $[\alpha]_D^{23}$ = -23.4° [c 0.007, benzene]; colorless oil;
^{1}H NMR (400 MHz, $CDCl_3$) δ 0.88 (dd, 6H, *J* = 4.4, 6.8 Hz), 1.64 (s, 3H), 1.71 (d, 3H, *J* = 1.2 Hz), 2.10 (ddd, 1H, *J* = 4.5, 6.8, 13.6 Hz), 3.17 (t, 2H, *J* = 3.6 Hz), 3.84 (ddd, 1H, *J* = 3.6, 5.6, 9.2 Hz), 4.10 (dd, 1H, *J* = 5.6, 8.4 Hz), 4.25 (t, 1H, *J* = 8.8 Hz), 5.10 (dt, 1H, *J* = 3.2, 10.0 Hz), 5.16-5.25 (m, 2H);
^{13}C NMR (125 MHz, $CDCl_3$) δ 14.6, 17.9, 18.2, 19.1, 25.9, 28.2, 29.3, 63.1, 83.8, 108.2, 134.9, 156.4, 205.0;
mass spectrum (APCI): m/e (% relative intensity) 259.9 (100) $(M+Na)^+$.

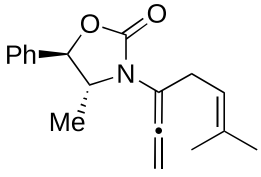

16d

Allenamide **16d** (90.0 mg, 0.32 mmol) was prepared in 40% yield according to general procedure 1.

16d: R_f = 0.50 [25% EtOAc/hexanes]; $[\alpha]_D^{23}$ = +30.5° [c 0.002, benzene]; colorless oil;
^{1}H NMR (500 MHz, $CDCl_3$) δ 0.78 (d, 3H, J = 6.0 Hz), 1.66 (s, 3H), 1.71 (s, 3H), 3.17 (dd, 1H, J = 7.0, 16.0 Hz), 3.17 (ddd, 1H, J = 3.5, 12.5, 16.0 Hz), 4.20 (ddd, 1H, J = 6.0, 7.5, 12.5 Hz), 5.12-5.18 (m, 2H), 5.24 (dt, 1H, J = 3.0, 10.5 Hz), 5.60 (d, 1H, J = 8.0 Hz), 7.27-7.40 (m, 5H);
^{13}C NMR (125 MHz, $CDCl_3$) δ 15.1, 18.3, 26.0, 29.6, 57.1, 79.0, 83.8, 108.2, 119.8, 126.5, 128.7, 128.8, 135.1, 135.4, 155.5, 204.9;
mass spectrum (APCI): m/e (% relative intensity) 284.1(40) $(M+H)^+$.

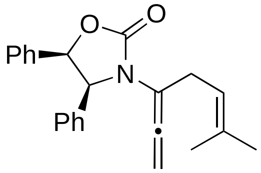

16e

Allenamide **16e** (160 mg, 0.46 mmol) was prepared in 46% yield according to general procedure 1.
16e: R_f = 0.39 [25% EtOAc/hexanes]; clear oil;
^{1}H NMR (500 MHz, $CDCl_3$) δ 1.57 (s, 3H), 1.69 (s, 3H), 3.19 (m, 2H), 4.87 (dt, 1H, J = 3.0, 10.0 Hz), 4.98 (dt, 1H, J = 3.0, 10.0 Hz), 5.14 (tsep, 1H, J = 1.0, 7.5 Hz), 5.17 (d, 1H, J = 7.0 Hz), 5.83 (d, 1H, J = 7.5 Hz), 6.83-6.85 (m, 2H), 6.92-6.97 (m, 2H), 7.03-7.10 (m, 6H);
^{13}C NMR (125 MHz, $CDCl_3$) δ 26.0, 29.3, 66.4, 79.7, 84.5, 108.8, 110.0, 119.8, 126.4, 128.0, 128.1, 128.2, 128.2, 128.3, 134.9, 135.0, 135.1, 156.0, 205.0;
mass spectrum (APCI): m/e (% relative intensity) 238.2 (40) $(Aux+H)^+$.

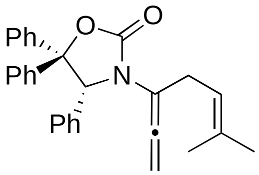

16f

Allenamide **16f** (160.0 mg, 0.46 mmol) was prepared in 46% yield according to general procedure 1.

16f: R_f = 0.47 [25% EtOAc/hexanes]; $[\alpha]_D^{23}$ = +165.5° [c 0.019, benzene]; colorless oil;
^{1}H NMR (400 MHz, $CDCl_3$) δ 1.48 (s, 3H), 1.58 (s, 3H), 3.09 (t, 2H, *J* = 4.0 Hz), 4.89-4.99 (m, 2H), 5.59 (s, 1H), 6.69- 7.10 (m, 10H), 7.31-7.39 (m, 1H), 7.41 (t, 1H, *J* = 8.0 Hz0), 7.65 (dd, 2H, *J* = 1.6, 7.2 Hz);
^{13}C NMR (125 MHz, $CDCl_3$) δ 18.1, 25.8, 29.5, 70.8, 84.2, 87.9, 108.5, 119.8, 126.4, 126.9, 127.5, 127.7, 128.1, 128.4, 128.7, 128.9, 128.9, 134.9, 136.1, 139.3, 143.4, 154.8, 205.1;
mass spectrum (APCI): m/e (% relative intensity) 420.2 (100) $(M-H)^+$.

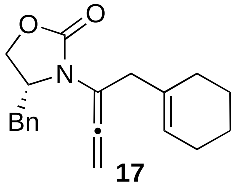

17

Allenamide **17** (106.0 mg, 0.34 mmol) was prepared in 46% yield according to general procedure 1.

17: R_f = 0.35 [25% EtOAc/hexanes]; $[\alpha]_D^{23}$ = -10.3° [c 0.015, CH_2Cl_2];
^{1}H NMR (500 MHz, $CDCl_3$) δ 1.60 (ddt, 4H, *J* = 2.4, 8.8, 14.0 Hz), 2.02 (dt, 1H, *J* = 4.8, 13.2 Hz), 2.62 (dd, 1H, *J* = 7.6, 10.8 Hz), 3.06 (d, 1H, *J* = 12.0 Hz), 3.23 (dt, 1H, *J* = 3.2, 12.8 Hz), 4.03 (dd, 1H, *J* = 4.4, 6.4 Hz), 4.08-4.13 (m, 1H), 4.16 (t, 1H, *J* = 6.4 Hz), 5.20 (ddt, 1H, *J* = 1.2, 1.6, 8.4 Hz), 5.34 (ddt, 1H, *J* = 0.4, 2.0, 8.4 Hz), 5.59 (s, 1H), 7.15 (d, 2H, *J* = 5.6 Hz), 7.26 (dd, 1H, *J* = 1.0, 5.6 Hz), 7.32 (t, 2H, *J* = 6.0 Hz);
^{13}C NMR (125 MHz, $CDCl_3$) δ 22.6, 23.2, 25.6, 28.3, 38.8, 39.2, 58.0, 67.0, 83.2, 106.8, 125.0, 127.4, 129.1, 129.3, 133.8, 136.0, 155.8, 205.5;
mass spectrum (APCI): m/e (% relative intensity) 310.2 (15) $(M+H)^+$.

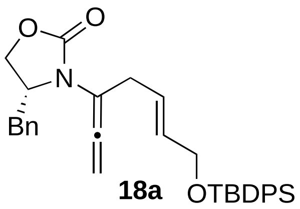

18a

Allenamide **18a** (336.6 mg, 0.64 mmol) was prepared in 50% yield according to general procedure 1.

18a: R_f = 0.33 [25% EtOAc/hexanes]; $[\alpha]_D^{23}$ = -11.5° [c 0.020, benzene]; colorless oil;

^{1}H NMR (500 MHz, $CDCl_3$) δ 1.06 (s, 9H), 2.64 (dd, 1H, *J* = 9.0, 13.5 Hz), 3.22 (dd, 1H, *J* = 4.0, 14.0 Hz), 3.30-3.33 (m, 2H), 4.04 (dd, 1H, *J* = 6.0, 8.5 Hz), 4.05-4.21 (m, 4H), 5.24 (dt, 1H, *J* = 3.0, 10.0 Hz), 5.33 (dt, 1H, *J* = 3.0, 10.0 Hz), 5.70-5.79 (m, 2H), 7.11 (d, 2H, *J* = 7.0 Hz), 7.25 (t, 1H, *J* = 6.5 Hz), 7.30 (t, 2H, *J* = 7.5 Hz), 7.34-7.42 (m, 6H), 7.67 (ddd, 4H, *J* = 1.0, 2.5, 7.5 Hz);

^{13}C NMR (125 MHz, $CDCl_3$) δ 14.4, 19.5, 22.9, 27.1, 31.8, 33.2, 35.0, 38.8, 58.2, 64.3, 67.0, 84.8, 108.4, 125.8, 127.5, 127.9, 129.2, 129.4, 129.9, 132.2, 134.0, 134.1, 135.8, 136.0, 155.9, 204.5;

mass spectrum (APCI): m/e (% relative intensity) 558.2 (15) $(M+H)^+$.

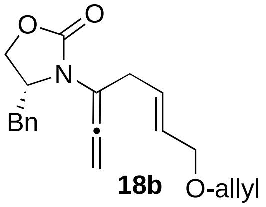

18b

Allenamide **18b** (254.8 mg, 0.78 mmol) was prepared in 59% yield according to general procedure 1.

18b: R_f = 0.28 [25% EtOAc/hexanes]; $[\alpha]_D^{23}$ = -30.0° [c 0.012, benzene]; colorless oil;

^{1}H NMR (400 MHz, $CDCl_3$) δ 2.66 (dd, 1H, *J* = 8.8, 13.2 Hz), 3.23 (dd, 1H, *J* = 3.2, 13.6 Hz), 3.30-3.33 (m, 2H), 3.96-3.98 (m, 3H), 4.04-4.14 (m, 2H), 4.20 (t, 1H, *J* = 7.6 Hz), 5.16-5.19 (m, 1H), 5.23-5.30 (m, 2H), 5.37 (dt, 1H, *J* = 3.2, 10.4 Hz), 5.72-5.74 (m, 2H), 5.86-5.96 (m, 1H), 7.15 (dd, 2H, *J* = 2.0, 8.4 Hz), 7.24-7.34 (m, 3H);

^{13}C NMR (100 MHz, $CDCl_3$) δ 33.2, 38.6, 58.0, 66.9, 70.6, 71.1, 84.9, 108.1, 117.2, 127.4, 129.1, 129.3, 129.7, 135.0, 135.9, 155.8, 204.4;

mass spectrum (APCI): m/e (% relative intensity) 326.2 (100) $(M+H)^+$.

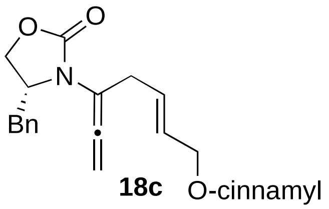

Allenamide **18c** (190.3 mg, 0.47 mmol) was prepared in 47% yield according to general procedure 1.

18c: R_f = 0.22 [25% EtOAc/hexanes]; colorless oil;
^{1}H NMR (400 MHz, $CDCl_3$) δ 2.66 (dd, 1H, *J* = 9.0, 14.0 Hz), 3.21 (dd, 1H, *J* = 3.5, 14.0 Hz), 3.28-3.38 (m, 2H), 3.96-3.98 (m, 3H), 4.01-4.16 (m, 6H), 4.19 (t, 1H, *J* = 8.0 Hz), 5.26 (dt, 1H, *J* = 3.0, 10.5 Hz), 5.37 (dt, 1H, *J* = 3.0, 10.5 Hz), 5.76 (m, 2H), 6.28 (dt, 1H, *J* = 6.0, 16.0 Hz), 6.60 (d, 1H, *J* = 16.0 Hz), 7.12-7.16 (m, 1H), 7.20-7.34 (m, 6H), 7.35-7.40 (m, 2H);
^{13}C NMR (100 MHz, $CDCl_3$) δ 33.3, 38.7, 58.1, 67.0, 70.7, 70.8, 85.0, 108.2, 126.4, 126.8, 127.5, 127.9, 128.8, 128.9, 129.2, 129.4, 129.5, 129.7, 132.7, 135.9, 137.0, 155.8, 204.5;
mass spectrum (APCI): m/e (% relative intensity) 176.1 (100) $(Aux+H)^+$.

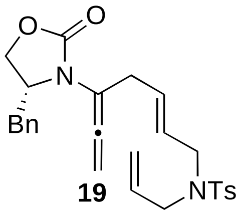

Allenamide **19** (200.1 mg, 0.42 mmol) was prepared in 42% yield according to general procedure 1.

19: R_f = 0.45 [40% EtOAc/hexanes]; $[\alpha]_D^{23}$ = +1.5^o [c 0.002, benzene]; colorless oil;
^{1}H NMR (500 MHz, $CDCl_3$) δ 2.42 (s, 3H), 2.66 (dd, 1H, *J* = 9.0, 13.5 Hz), 3.21 (dd, 1H, *J* = 3.5, 13.5 Hz), 3.23-3.26 (m, 2H), 3.78 (t, 4H, *J* = 5.0 Hz), 4.04-4.14 (m, 2H), 4.20 (t, 1H, *J* = 8.0 Hz), 5.09-5.16 (m, 2H), 5.25 (dt, 1H, *J* = 3.5, 10.0 Hz), 5.36 (dt, 1H, *J* = 3.5, 10.0 Hz), 5.55-5.64 (m, 2H), 7.13 (d, 2H, *J* = 7.0 Hz), 7.25-7.33 (m, 5H), 7.71 (d, 2H, *J* = 8.5 Hz);
^{13}C NMR (100 MHz, $CDCl_3$) δ 21.8, 33.1, 38.6, 48.7, 49.5, 58.1, 67.0, 85.2, 108.2, 119.2, 127.5, 127.5, 127.6, 129.2, 129.3, 130.0, 130.7, 133.0, 135.9, 137.7, 143.5, 155.8, 204.3;
mass spectrum (APCI): m/e (% relative intensity) 501.2 (20) $(M+Na)^+$.

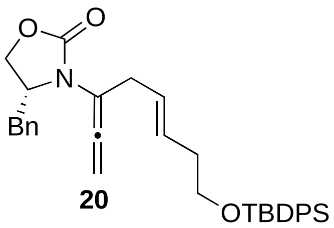

20

Allenamide **20** (322.8 mg, 0.60 mmol) was prepared in 65% yield according to general procedure 1.

20: R_f = 0.30 [1:4 EtOAc/hexanes]; clear oil;

^{1}H NMR (400 MHz, $CDCl_3$) δ 1.04 (s, 9H), 2.30 (q, 2H, *J* = 6.8 Hz), 2.61 (dd, 1H, *J* = 8.8, 13.2 Hz), 3.18 (dd, 1H, *J* = 3.2, 13.2 Hz), 3.23 (dt, 2H, *J* = 3.2, 6.8 Hz), 3.68 (t, 2H, *J* = 6.8 Hz), 4.01 (dd, 1H, *J* = 5.2, 7.6 Hz), 4.08 (tdd, 2H, *J* = 3.6, 4.0, 8.8 Hz), 4.15 (t, 1H, *J* = 7.6 Hz), 5.19 (dt, 1H, *J* = 3.2, 10.4 Hz), 5.30 (dt, 1H, *J* = 3.2, 10.4 Hz), 5.47 (dt, 1H, *J* = 6.8, 15.2 Hz), 5.59 (dt, 1H, *J* = 6.8, 15.2 Hz), 7.10-7.14 (m, 2H), 7.22-7.33 (m, 3H), 7.34-7.44 (m, 6H), 7.64-7.69 (m, 4H);

^{13}C NMR (100 MHz, $CDCl_3$) δ 19.5, 27.1, 33.6, 36.2, 38.7, 58.1, 64.1, 66.9, 84.6, 108.4, 127.4, 127.5, 127.8, 129.1, 129.3, 129.8, 130.2, 134.2, 135.8, 135.9, 155.8, 204.5.

mass spectrum (APCI): m/e (% relative intensity) 514.2 (80) $(M\text{-}CO\text{+}H_2)^+$.

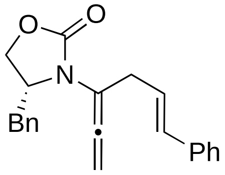

21

Allenamide **21** (302 mg, 0.91 mmol) was prepared in 38% yield according to general procedure 1.

21: R_f = 0.25 [25% EtOAc/hexanes]; $[\alpha]_D^{23}$ = -0.83^o [c 0.060, benzene]; colorless oil;

^{1}H NMR (500 MHz, $CDCl_3$) δ 2.67 (dd, 1H, *J* = 9.5, 13.5 Hz), 3.27 (dd, 1H, *J* = 3.5, 13.5 Hz), 3.41-3.54 (m, 2H), 4.05 (dd, 1H, *J* = 5.5, 8.5 Hz), 4.12 (ddd, 1H, *J* = 3.0, 7.5, 11.5 Hz), 4.20 (t, 1H, *J* = 8.0 Hz), 5.26 (dt, 1H, *J* = 3.0, 10.5 Hz), 5.39 (dt, 1H, *J* = 3.0, 10.5 Hz), 6.22 (dt, 1H, *J* = 7.0, 14.0 Hz), 6.55 (bd, 1H, *J* = 15.5 Hz), 7.12 (d, 2H, *J* = 7.0 Hz), 7.20-7.32 (m, 6H), 7.38 (d, 2H, *J* = 7.5 Hz); ^{13}C NMR (125 MHz, $CDCl_3$) δ 34.0, 38.7, 58.2, 67.0, 85.0, 125.8, 126.5, 127.5, 127.6, 129.2, 129.2, 129.4, 132.9, 135.9, 137.6, 155.9, 204.5;

IR (neat) cm^{-1} 3031w, 2927w, 2363w. 1752s, 1703m, 1498m, 1480m, 1455m, 1400m, 1358;

mass spectrum (APCI): m/e (% relative intensity) 332.1 (100) $(M+H)^+$.

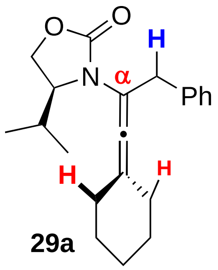

29a

Allenamide **29a** (120.2 mg, 0.37 mmol) was prepared in 37% yield according to general procedure 1.

29a: R_f = 0.32 [25% EtOAc/hexanes]; $[\alpha]_D^{23}$ = -80.4° [c 0.005, benzene]; colorless oil;
^{1}H NMR (500 MHz, $CDCl_3$) δ 0.62 (d, 3H, *J* = 8.5 Hz), 0.77 (d, 1H, *J* = 9.0 Hz), 0.83-0.90 (m, 1H), 1.32-1.56 (m, 6H), 1.96-2.12 (m, 4H), 3.69-3.77 (m, 2H), 3.83 (bd, 1H, *J* = 19.0 Hz), 4.02 (dd, 1H, 7.5, 11.0 Hz), 4.16 (t, 1H, *J* = 11.5 Hz), 7.17-7.28 (m, 5H);
^{13}C NMR (125 MHz, $CDCl_3$) δ 14.1, 18.2, 26.1, 27.5, 27.6, 27.7, 27.7, 32.2, 32.3, 37.7, 61.1, 62.7, 105.8, 112.2, 126.6, 128.4, 129.7, 139.2, 156.7, 193.1;
IR (neat) cm^{-1} 2928m, 1961w, 1750s, 1479m, 1445m, 1392m;
mass spectrum (APCI): m/e (% relative intensity) 326.3 (100) (M+H).

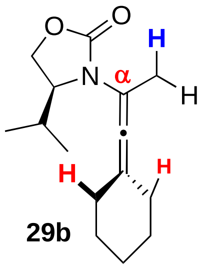

29b

Allenamide **29b** isomerized to **33b** during its purification.

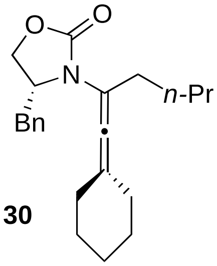

30

Allenamide **30** (68.0 mg, 0.20 mmol) was prepared in 40% yield according to general procedure 1.

30: R_f = 0.50 [25% EtOAc/hexanes]; $[\alpha]_D^{23}$ = +23.6° [c 0.015, benzene]; colorless oil;
^{1}H NMR (400 MHz, $CDCl_3$) δ 0.85-0.91 (m, 2H), 0.94 (t, 3H, *J* = 7.2 Hz), 1.26-1.71 (m, 10H), 2.16-2.29 (m, 3H), 2.46-2.67 (m, 2H), 3.25 (dd, 1H, *J* = 3.2, 13.2 Hz), 3.99-4.22 (m, 3H), 7.14 (d, 2H, *J*

= 6.8 Hz), 7.23-7.34 (m, 3H);
^{13}C NMR (100 MHz, $CDCl_3$) δ 14.3, 22.3, 26.2, 27.7, 27.9, 29.6, 30.2, 32.7, 32.9, 38.2, 58.2, 66.6, 106.4, 112.5, 127.3, 129.1, 129.3, 129.3, 136.1, 156.0, 191.4;
IR (neat) cm^{-1} 2929m, 1960w, 1754s, 1476m, 1395m;
mass spectrum (APCI): m/e (% relative intensity) 340.2 (100) (M+H).

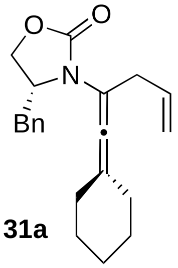

31a

Allenamide **31a** was prepared in 40% yield according to general procedure 1.

31a: R_f = 0.45 [1:4 EtOAc/hexanes]; clear oil;
^{1}H NMR (400 MHz, $CDCl_3$) δ 0.83-0.90 (m, 2H), 1.24-1.30 (m, 2H), 1.52-1.72 (m, 2H), 2.18-2.24 (m, 2H), 2.29 (t, 2H, *J* = 6 Hz), 2.68 (dd, 1H, *J* = 9.2, 13.6 Hz), 3.33 (dp, 2H, *J* = 1.2, 6.8 Hz), 4.01-4.11 (m, 2H), 4.17 (t, 1H, *J* = 8 Hz), 5.09 (dp, 1H, *J* = 1.2, 10 Hz), 5.18 (dq, 1H, *J* = 1.6, 17.2 Hz), 5.88 (ddt, 1H, *J* = 6.8, 10, 16.8 Hz), 7.11-7.16 (m, 2H), 7.24-7.28 (m, 1H), 7.30-7.34 (m, 2H);
^{13}C NMR (125 MHz, $CDCl_3$) δ 26.2, 27.7, 27.9, 31.8, 32.7, 32.8, 35.5, 38.1, 58.2, 66.6, 104.9, 113.0, 116.9, 127.3, 129.1, 129.2, 129.3, 135.1, 136.1, 156.0, 191.8;
mass spectrum (APCI): m/e (% relative intensity) 324.1 (5) $(M+H)^+$.

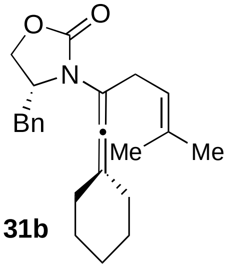

31b

Allenamide **31b** (31.8 mg, 0.09 mmol) was prepared in 18% yield according to general procedure 1.

31b: R_f = 0.22 [25% EtOAc/hexanes]; colorless oil;
^{1}H NMR (500 MHz, $CDCl_3$) δ 1.50-1.76 (m, 12H), 2.12-2.30 (m, 4H), 2.63 (dd, 1H, *J* = 9.0, 13.0 Hz), 3.16-3.23 (m, 2H), 3.27 (dd, 1H, *J* = 7.5, 16.0 Hz), 4.00-4.10 (m, 2H), 4.15 (t, 1H, *J* = 8.0 Hz), 5.21 (tsep, 1H, *J* = 1.5, 7.0 Hz), 7.12 (d, 2H, *J* = 7.0 Hz), 7.22-7.28 (m, 1H), 7.32 (t, 2H, *J* = 7.5 Hz);
^{13}C NMR (125 MHz, $CDCl_3$) δ 26.0, 26.1, 26.3, 27.8, 28.0, 30.0, 32.8, 33.0, 38.2, 58.4, 66.7, 105.5,

112.7, 120.8, 127.3, 129.2, 129.4, 134.2, 136.2, 156.1, 191.9;
mass spectrum (APCI): m/e (% relative intensity) 326.3 (40) $(M\text{-}CO+H_2)^+$.

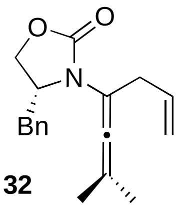

32

Allenamide **32** (161 mg, 0.57 mmol) was isolated in 55% yield according to general procedure 1.

32: R_f = 0.36 [1:4 EtOAc/hexanes]; $[\alpha]_D^{23}$ = +57.6° [c 0.046, benzene] yellow oil;
^{1}H NMR (500 MHz, $CDCl_3$) δ 1.83 (s, 3H), 1.88 (s, 3H), 2.63 (dd, 2H, *J* = 9.5, 13.5 Hz), 3.23 (dd, 1H, *J* = 2.5, 13.5 Hz), 3.28 (ddt, 1H, *J* = 1.5, 7.0, 16.0 Hz), 3.31 (ddt, 1H, *J* = 1.5, 7.0, 16.0 Hz), 4.01-4.08 (m, 2H), 4.11-4.18 (m, 1H), 5.07 (ddt, 1H, *J* = 1.0, 1.5, 10.5 Hz), 5.16 (ddt, 1H, *J* = 1.5, 1.5, 17.0 Hz), 5.82 (ddt, 1H, *J* = 7.0, 10.5, 17.0 Hz), 7.12 (d, 2H, *J* = 7.0 Hz), 7.24-7.28 (m, 1H), 7.30-7.34 (m, 2H);
^{13}C NMR (125 MHz, $CDCl_3$) δ 21.8, 21.9, 35.4, 38.3, 58.3, 66.7, 105.2, 105.7, 117.0, 127.4, 129.2, 129.4, 135.0, 136.1, 156.0, 195.5;
mass spectrum (APCI): m/e (% relative intensity) 284.2 (100) $(M+H)^+$.

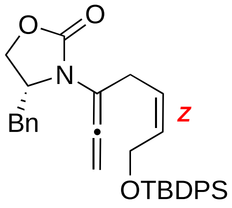

37a

Allenamide **37a** (311.3 mg, 0.60 mmol) was prepared in 44% yield according to general procedure 1.

37a: R_f = 0.38 [25% EtOAc/hexanes]; $[\alpha]_D^{23}$ = -1.0° [c 0.004, benzene]; colorless oil;
^{1}H NMR (400 MHz, $CDCl_3$) δ 1.04 (s, 9H), 2.58 (ddt, 1H, *J* = 2.8, 6.4, 13.2 Hz), 3.09-3.16 (m, 3H), 3.99-4.17 (m, 3H), 4.29 (ddd, 2H, *J* = 0.4, 1.6, 7.5 Hz), 5.17 (dt, 1H, *J* = 3.2, 10.4 Hz), 5.26 (dt, 1H, *J* = 3.2, 10.4 Hz), 5.46-5.53 (m, 1H), 5.72-5.78 (m, 1H), 7.09 (dd, 2H, *J* = 3.6, 8.0 Hz), 7.22-7.43 (m, 9H), 7.67-7.70 (m, 4H);
^{13}C NMR (100 MHz, $CDCl_3$) δ 14.3, 19.4, 22.9, 27.0, 28.6, 31.8, 38.6, 58.0, 60.6, 66.9, 85.0, 108.2, 125.7, 127.4, 127.9, 129.1, 129.3, 129.8, 132.2, 134.0, 135.8, 135.8, 204.2;
mass spectrum (APCI): m/e (% relative intensity) 558.3 (10) $(M+H)^+$.

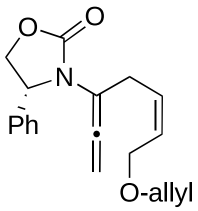

37b

Allenamide **37b** (201.1 mg, 0.65 mmol) was prepared in 65% yield according to general procedure 1.

37b: R_f = 0.20 [25% EtOAc/hexanes]; colorless oil;

^{1}H NMR (400 MHz, $CDCl_3$) δ 3.15-3.32 (m, 2H), 3.88-4.02 (m, 4H), 4.13 (dd, 1H, *J* = 8.0, 8.8 Hz), 4.63 (t, 1H, *J* = 8.8 Hz), 4.90-4.98 (m, 2H), 5.04 (dt, 1H, *J* = 3.2, 10.0 Hz), 5.14-5.20 (m, 1H), 5.25 (dq, 1H, *J* = 1.6, 17.2 Hz), 5.42-5.52 (m, 1H), 5.60-5.68 (dtt, 1H, *J* = 1.6, 6.4, 10.8 Hz), 5.88 (ddt, 1H, *J* = 5.6, 10.4, 17.2 Hz), 7.28-7.42 (m, 5H);

^{13}C NMR (125 MHz, $CDCl_3$) δ 28.8, 61.7, 66.0, 70.2, 71.4, 85.2, 107.9, 117.3, 127.4, 128.1, 129.2, 129.2, 129.4, 135.1, 138.5, 156.4, 204.8;

mass spectrum (APCI): m/e (% relative intensity) 126.2 (100) $(Aux+H)^+$.

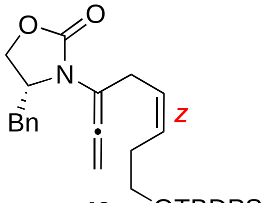

40

Allenamide **40** (186.8 mg, 0.347 mmol) was prepared in 69% yield according to general procedure 1.

40: R_f = 0.33 [1:4 EtOAc/hexanes]; clear oil;

^{1}H NMR (400 MHz, $CDCl_3$) δ 1.04 (s, 9H), 2.37 (q, 2H, *J* = 6.8 Hz), 2.62 (dd, 1H, *J* = 9.6, 13.6 Hz), 3.18 (dd, 1H, *J* = 3.2, 13.6 Hz), 3.28 (m, 2H), 3.68 (t, 2H, *J* = 6.8 Hz), 4.00-4.11 (m, 2H), 4.17 (t, 1H, *J* = 7.6 Hz), 5.20 (dt, 1H, *J* = 2.8, 10.4 Hz), 5.30 (dt, 1H, *J* = 3.2, 10.0 Hz), 5.45-5.63 (m, 2H), 7.10-7.14 (m, 2H), 7.24-7.34 (m, 3H), 7.34-7.44 (m, 6H), 7.64-7.69 (m, 4H);

^{13}C NMR (100 MHz, $CDCl_3$) δ 19.4, 27.1, 28.5, 31.2, 38.6, 58.1, 63.7, 67.0, 84.8, 108.5, 126.5, 127.4, 127.8, 129.0, 129.3, 129.8, 134.1, 135.8, 135.9, 204.4.

General procedure 2 for the thermal isomerization of allenamides

A solution of the respective allenamide in anhydrous CH_3CN (0.1 M) in a sealed tube was heated to appropriate temperature (115 °C or 135 °C). Upon completion of the reaction (16 h), the solution was cooled to RT and solvent removed in vacuo to afford the crude product. Separation and purification of the resulting crude residue by silica gel flash column chromatography (gradient eluent: EtOAc in hexane) afforded the desired 2-amido-diene.

General procedure 3 for the acid-catalyzed isomerization of allenamides

To a solution of the respective allenamide (1.0 equiv) in anhydrous CH_2Cl_2 (0.1 M) in a small screw-cap scintillation vial equipped with a magnetic stirring bar, was added the appropriate acid (PTSA or CSA, 10 mol %). The solution was stirred for 10 min and filtered through a short pad of silica gel. Elution with EtOAc/hexanes (1:1) followed by concentration in vacuo afforded the crude product. Separation and purification of the resulting crude residue by silica gel flash column chromatography (gradient eluent: EtOAc in hexane) afforded the desired 2-amido-diene.

Characterizations of 2-amido-dienes

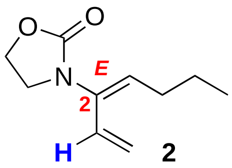

2

Thermal condition: 2-Amido-diene **2** (29.0 mg, 0.16 mmol) was isolated in 78% yield according to general procedure 2.

Acid-catalyzed condition: 2-Amido-diene **2** (14.0 mg, 0.077 mmol) was isolated in 74% yield according to general procedure 3.

2: R_f = 0.50 [40% EtOAc/hexanes]; colorless oil;
^{1}H NMR (500 MHz, $CDCl_3$) δ 0.94 (t, 3H, J = 7.5 Hz), 1.49 (ddd, 1H, J = 3.0, 7.5, 14.5 Hz), 2.24 (q, 2H, J = 7.5 Hz), 3.76 (t, 3H, J = 8.0 Hz), 4.41 (t, 2H, J = 8.0 Hz), 5.25 (dd, 2H, J = 7.0, 17.0 Hz), 5.69 (t, 1H, J = 7.5 Hz), 6.52 (dd, 1H, J = 11.0, 17.0 Hz);
^{13}C NMR (125 MHz, $CDCl_3$) δ 14.0, 22.7, 29.5, 47.6, 62.2, 115.6, 127.9, 132.7, 134.0, 157.4;
IR (neat) cm^{-1} 2959w, 1745s, 1645w, 1598w, 1481w, 1406m;
mass spectrum (APCI): m/e (% relative intensity) 182.1 (100) $(M+H)^+$.

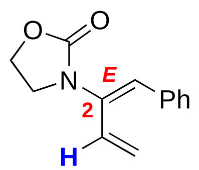

4

Thermal condition: 2-Amido-diene **4** (39.0 mg, 0.18 mmol) was isolated in 71% yield according to general procedure 2.

4: R_f = 0.48 [40% EtOAc/hexanes]; colorless oil;
^{1}H NMR (500 MHz, $CDCl_3$) δ 3.87 (t, 2H, *J* = 8.0 Hz), 4.47 (t, 2H, *J* = 7.5 Hz), 5.36 (d, 1H, *J* = 11 Hz), 5.38 (d, 1H, *J* = 17.5Hz), 6.66 (dd, 1H, *J* = 11.0, 12.5 Hz), 6.70 (s, 1H), 7.20-7.38 (m, 5H);
^{13}C NMR (125 MHz, $CDCl_3$) δ 47.4, 62.3, 118.0, 128.1, 128.6, 128.6, 128.8, 129.0, 129.7, 130.0, 134.8, 135.1, 157.2;
IR (neat) cm^{-1} 2921w, 1747s, 1594w, 1482w, 1404s;
mass spectrum (APCI): m/e (% relative intensity) 216.1 (100) $(M+H)^+$.

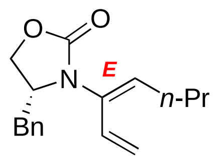

8a

Thermal condition: 2-Amido-diene **8a** (46.0 mg, 0.16 mmol) was isolated in 77% yield according to general procedure 2.

Acid-catalyzed condition: 2-Amido-diene **8a** (20.0 mg, 0.074 mmol) was isolated in 87% yield according to general procedure 3.

8a: R_f = 0.25 [25% EtOAc/hexanes]; $[\alpha]_D^{23}$ = -23.3° [c 0.022, benzene]; colorless oil;
^{1}H NMR (500 MHz, $CDCl_3$) δ 0.97 (t, 3H, *J* = 7.5 Hz), 1.44 (dt, 2H, *J* = 2.5, 7.0 Hz), 2.29 (dd, 2H, *J* = 7.5, 15.0 Hz), 2.60 (dd, 1H, *J* = 10.0, 13.5 Hz), 3.07 (dd, 1H, *J* = 3.5, 13.5 Hz), 4.10 (dd, 1H, *J* = 5.5, 8.0 Hz), 4.18-4.25 (m, 1H), 4.27 (t, 1H, *J* = 8.5 Hz), 5.29 (dd, 2H, *J* = 9.5, 20.5 Hz), 5.74 (t, 1H, *J* = 8.0 Hz), 6.58 (dd, 1H, *J* = 11.0, 17.5 Hz), 7.12 (d, 2H, *J* = 7.5 Hz), 7.26 (dd, 1H, *J* = 3.0, 10 Hz), 7.31 (t, 2H, *J* = 6.5 Hz);
^{13}C NMR (125 MHz, $CDCl_3$) δ 11.9, 20.6, 27.5, 37.4, 56.1, 65.4, 113.7, 125.2, 126.3, 127.0, 127.0, 127.1, 127.1, 130.1, 132.9, 133.8, 155.0;
IR (neat) cm^{-1} 2961w, 2361w, 1753s, 1600w, 1454w, 1402m;
mass spectrum (APCI): m/e (% relative intensity) 272.1 (100) $(M+H)^+$.

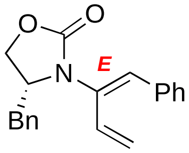

8b

Thermal condition: 2-Amido-diene **8b** (88.0 mg, 0.29 mmol) was isolated in 74% yield according to general procedure 2.

Acid-catalyzed condition: 2-Amido-diene **8b** (38.0 mg, 0.12 mmol) was isolated in 83% yield according to general procedure 3.

8b: R_f = 0.25 [25% EtOAc/hexanes]; $[\alpha]_D^{23}$ = +80.9° [c 0.021, benzene]; colorless oil;
^{1}H NMR (500 MHz, $CDCl_3$) δ 2.70 (dd, 1H, *J* = 8.0, 14.0 Hz), 3.13 (dd, 1H, *J* = 3.5, 13.0 Hz), 4.19 (dd, 1H, *J* = 6.5, 11.5 Hz), 4.31-4.39 (m, 2H), 4.39 (d, 1H, *J* = 10.5 Hz), 5.45 (d, 1H, *J* = 17.0 Hz), 6.71 (d, 1H, *J* = 6.5, 17.5 Hz), 6.75 (s, 1H), 7.12 (d, 2H, *J* = 7.5 Hz), 7.25 (d, 2H, *J* = 6.5 Hz), 7.30 (t, 3H, *J* = 7.0 Hz), 7.35 (t, 4H, *J* = 6.5 Hz);
^{13}C NMR (125 MHz, $CDCl_3$) δ 37.2, 55.9, 65.3, 115.9, 125.3, 126.1, 126.7, 126.8, 127.0, 127.1, 127.3, 127.7, 130.1, 130.1, 130.8, 132.9, 133.6, 154.8;
IR (neat) cm^{-1} 3057w, 2363w, 1752s, 1602w, 1478w, 1446w, 1405, 1401m;
mass spectrum (APCI): m/e (% relative intensity) 306.1 (100) $(M+H)^+$.

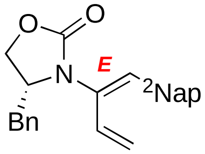

8c

Thermal condition: 2-Amido-diene **8c** (22.0 mg, 0.062 mmol) was isolated in 73% yield according to general procedure 2.

8c: R_f = 0.15 [25% EtOAc/hexanes]; $[\alpha]_D^{23}$ = +87.6° [c 0.005, benzene]; colorless oil;
^{1}H NMR (500 MHz, $CDCl_3$) δ 2.74 (dd, 1H, *J* = 9.5, 13.5 Hz), 3.18 (dd, 1H, *J* = 4.0, 13.5 Hz), 4.19 (dd, 1H, *J* = 5.5, 8.0 Hz), 4.32-4.43 (m, 2H), 5.46 (d, 1H, *J* = 11.0 Hz), 5.53 (d, 1H, *J* = 17.0 Hz), 6.79 (dd, 1H, *J* = 10.5, 17.0 Hz), 6.90 (s, 1H), 7.16 (d, 2H, *J* = 7.0 Hz), 7.23-7.26 (m, 1H), 7.25 (t, 2H, *J* = 7.5 Hz), 7.45 (dd, 1H, *J* = 1.5, 8.5 Hz), 7.50 (ddd, 2H, *J* =2.5, 3.5, 5.5 Hz), 7.83 (dt, 4H, *J* = 3.5, 4.0 Hz);
^{13}C NMR (125 MHz, $CDCl_3$) δ 39.5, 58.1, 67.5, 118.4, 126.7, 126.8, 127.4, 127.5, 128.0, 128.2, 128.4, 129.2, 129.3, 129.3, 129.6, 132.3, 133.1, 133.3, 133.4, 135.8, 156.9;
IR (neat) cm^{-1} 3057w, 2925w, 1751s, 1601w, 1498w, 1478w, 1475w, 1454w, 1400m,;
mass spectrum (APCI): m/e (% relative intensity) 356.1 (100) $(M+H)^+$.

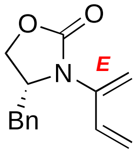

8d

Thermal condition: 2-Amido-diene **8d** (17.0 mg, 0.075 mmol) was isolated in 69% yield according to general procedure 2. An alumina column was used for the isolation.

8d: R_f = 0.19 [25% EtOAc/hexanes]; $[\alpha]_D^{23}$ = -2.89° [c 0.003, benzene]; colorless oil;
^{1}H NMR (400 MHz, $CDCl_3$) δ 2.61-2.70 (m, 1H), 3.14 (dd, 1H, *J* = 3.2, 13.6 Hz), 4.11-4.30 (m, 3H), 5.27 (s, 1H), 5.32 (d, 1H, *J* = 10.8 Hz), 5.40 (s, 1H), 5.46 (d, 1H, *J* = 17.2 Hz), 6.41 (dd, 1H, *J* =10.8, 17.2 Hz), 7.14 (d, 2H, *J* = 7.2 Hz), 7.20-7.34 (m, 3H);
^{13}C NMR (100 MHz, $CDCl_3$) δ 38.7, 58.0, 66.3, 67.1, 113.6, 117.1, 127.4, 129.2, 129.2, 129.6, 133.1, 135.6, 140.4, 156.4;
IR (neat) cm^{-1} 2921m, 2853m, 2360w, 1758s, 1702w, 1595w, 1478w, 1455w, 1404m, 1376w;
mass spectrum (APCI): m/e (% relative intensity) 230.2 (100) $(M+H)^+$.

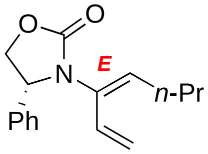

9a

Acid-catalyzed condition: 2-Amido-diene **9a** (45.1 mg, 0.16 mmol) was isolated in 82% yield according to general procedure 3.

9a: R_f = 0.22 [25% EtOAc/hexanes]; $[\alpha]_D^{23}$ = -86.4° [c 0.037, benzene]; yellow solid; mp 35-37 °C;
^{1}H NMR (400 MHz, $CDCl_3$) δ 0.64 (t, 3H, *J* = 7.6 Hz), 1.12-1.27 (m, 2H), 2.05 (dd, 2H, *J* = 7.2, 12.4 Hz), 4.34 (dd, 1H, *J* = 7.2, 8.8 Hz), 4.74 (dt, 1H, *J* = 2.0, 8.8 Hz), 5.02 (dd, 1H, *J* = 7.2, 8.8 Hz), 5.24 (t, 1H, *J* = 11.2 Hz), 5.35 (t, 1H, *J* = 8.8 Hz), 6.40 (dd, 1H, *J* = 10.8, 17.2 Hz), 7.26-7.41 (m, 5H);
^{13}C NMR (125 MHz, $CDCl_3$) δ 13.5, 22.4, 29.3, 61.6, 67.0, 115.5, 127.8, 128.0, 129.2, 129.2, 131.9, 135.3, 138.3, 157.2;
IR (neat) cm^{-1} 2959w, 1756s, 1479w, 1458w, 1396w;
mass spectrum (APCI): m/e (% relative intensity) 258.2 (100) $(M+H)^+$.

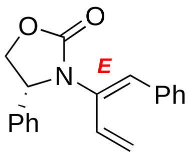

9b

Acid-catalyzed condition: 2-Amido-diene **9b** (404.0 mg, 1.39 mmol) was isolated in 76% yield according to general procedure 3.

9b: R_f = 0.33 [40% EtOAc/hexanes]; $[\alpha]_D^{23}$ = +25.3° [c 0.103, benzene]; yellow oil;
^{1}H NMR (500 MHz, $CDCl_3$) δ 4.42 (dd, 1H, *J* = 6.5, 8.5 Hz), 4.79 (t, 1H, *J* = 8.5 Hz), 5.15 (dd, 1H, *J* = 6.5, 8.5 Hz), 5.36 (dt, 1H, *J* = 1.0, 11.0 Hz), 5.49 (bd, 1H, *J* = 17.0 Hz), 6.39 (s, 1H), 6.50 (dd, 1H, *J* = 10.5, 17.5 Hz), 7.10 (d, 2H, *J* = 7.5 Hz), 7.19-7.40 (m, 8H);
^{13}C NMR (125 MHz, $CDCl_3$) δ 61.5, 70.0, 117.8, 127.8, 128.0, 128.9, 129.0, 129.2, 129.4, 129.4, 129.6, 132.4, 132.9, 135.1, 138.1, 157.0;
IR (neat) cm^{-1} 3032w, 1750s, 1599w, 1495w, 1457w, 1395m;
mass spectrum (APCI): m/e (% relative intensity) 292.2 (100) $(M+H)^+$.

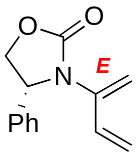

9c

Thermal condition: 2-Amido-diene **9c** (104.0 mg, 0.48 mmol) was isolated in 69% yield according to general procedure 2. An alumina column was used for the isolation.

9c: R_f = 0.15 [25% EtOAc/hexanes]; $[\alpha]_D^{23}$ = -40.9° [c 0.021, benzene]; colorless oil;
^{1}H NMR (500 MHz, $CDCl_3$) δ 4.24 (dd, 1H, *J* = 7.0, 9.0 Hz), 4.72 (t, 1H, *J* = 8.5 Hz), 4.82 (s, 1H), 5.06 (s, 1H), 5.13 (dd, 1H, *J* = 6.5, 8.5 Hz), 5.22 (d, 1H, *J* = 11.0 Hz), 5.43 (d, 1H, *J* = 17.0 Hz), 6.29 (dd, 1H, *J* =11.0, 17.5 Hz), 7.27-7.40 (m, 5H);
^{13}C NMR (100 MHz, $CDCl_3$) δ 61.5, 70.2, 111.7, 116.7, 127.1, 129.3, 129.4, 132.9, 138.1, 140.5, 156.4;
IR (neat) cm^{-1} 2953w, 2361w, 1748s, 1637w, 1590w, 1458m, 1392m;
mass spectrum (APCI): m/e (% relative intensity) 216.1 (100) $(M+H)^+$.

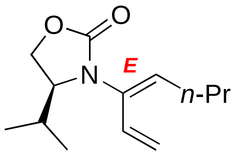

10a

Thermal condition: 2-Amido-diene **10a** (19.0 mg, 0.082 mmol) was isolated in 62% yield according to general procedure 2.

10a: R_f = 0.22 [25% EtOAc/hexanes]; $[\alpha]_D^{23}$ = -15.0° [c 0.007, benzene]; colorless oil;
^{1}H NMR (500 MHz, $CDCl_3$) δ 0.88 (d, 3H, *J* = 7.5 Hz), 0.92 (d, 3H, *J* = 7.0 Hz), 0.95 (d, 3H, *J* = 7.5 Hz), 1.49 (sep, 1H, *J* = 7.0 Hz), 1.90 (dt, 1H, *J* = 3.5, 7.5 Hz), 2.27 (q, 2H, *J* = 7.5 Hz), 3.94 (ddd, 1H, *J* = 4.0, 6.0, 9.0 Hz), 4.16 (dd, 1H, *J* = 5.5, 8.5 Hz), 4.35 (t, 1H, *J* = 9.0 Hz), 5.23 (dd, 2H, *J* = 9.5, 17.5 Hz), 5.70 (t, 1H, *J* = 8.0 Hz), 6.52 (dd, 1H, *J* = 9.5, 17.5 Hz);
^{13}C NMR (125 MHz, $CDCl_3$) δ 14.0, 14.9, 18.3, 22.7, 29.0, 29.6, 60.8, 63.5, 115.8, 128.1, 132.3, 134.6, 157.7;
IR (neat) cm^{-1} 2960w, 2361w, 1750s, 1646w, 1599w, 1464w, 1406m;
mass spectrum (APCI): m/e (% relative intensity) 224.1 (100) $(M+H)^+$.

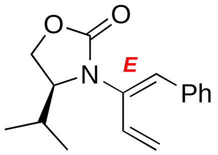

10b

Thermal condition: 2-Amido-diene **10b** (27.0 mg, 0.11 mmol) was isolated in 82% yield according to general procedure 2.

10b: R_f = 0.21 [25% EtOAc/hexanes]; $[\alpha]_D^{23}$ = -110.0° [c 0.005, benzene]; white solid; mp 128-131 ºC;
^{1}H NMR (500 MHz, $CDCl_3$) δ 0.92 (d, 3H, *J* = 7.0 Hz), 0.99 (t, 3H, *J* = 7.0 Hz), 1.98 (dt, 1H, *J* = 3.5, 7.5 Hz), 4.09 (ddd, 1H, *J* = 4.0, 5.5, 9.5 Hz), 4.21 (dd, 1H, *J* = 5.5, 9.0 Hz), 4.39 (t, 1H, *J* = 9.0 Hz), 5.37 (d, 1H, *J* = 11 Hz), 5.44 (bd, 1H, *J* =17.0 Hz) 6.64 (dd, 1H, *J* = 10.5, 17.5 Hz), 6.72 (s, 1H), 7.27-7.37 (m, 5H);
^{13}C NMR (125 MHz, $CDCl_3$) δ 15.1, 18.3, 29.3, 60.7, 63.7, 118.1, 128.1, 128.5, 129.2, 129.8, 131.6, 131.7, 133.3, 135.3, 157.5;
IR (neat) cm^{-1} 2964w, 2929w, 1750s, 1632w, 1591w, 1485w, 1402m;
mass spectrum (APCI): m/e (% relative intensity) 258.2 (100) $(M+H)^+$.

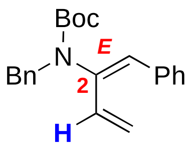

12

Thermal condition: 2-amido-diene **12** (15.0 mg, 0.045 mmol) was isolated in 45% yield according to general procedure 2.

Acid-catalyzed condition: 2-amido-diene **12** (20.0 mg, 0.060 mmol) was isolated in 61% yield according to general procedure 3.

12: R_f = 0.38 [10% EtOAc/hexanes];
^{1}H NMR (500 MHz, $CDCl_3$, 55 °C) δ 1.45 (s, 9H), 4.60 (s, 2H), 5.16 (d, 1H, *J* = 10.0 Hz), 5.26 (d, 1H, *J* = 17.0 Hz), 6.59 (dd, 1H, *J* = 17.0, 10.5 Hz), 7.13 (d, 2H, *J* = 7.5 Hz), 7.20-7.35 (m, 8H);
^{13}C NMR (125 MHz, $CDCl_3$, 55 °C) δ 28.5, 52.4, 80.1, 116.1, 127.5, 127.6, 128.4, 128.5, 129,2, 129.4, 130.4, 131.9, 136.0, 138.9, 139.1, 143.8, 155.5;
IR (thin film) cm^{-1} 2972m, 1736s, 1366s, 1227m, 1147s;
mass spectrum (APCI): m/e (% relative intensity) 236 (100) $(M+H\text{-}Boc)^+$.

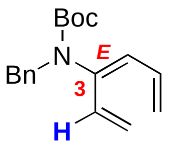

14

Acid-catalyzed condition: 3-amido-triene **14** (31.0 mg, 0.11 mmol) was isolated in 86% yield according to general procedure 3.

14: R_f = 0.12 [20% EtOAc/hexanes]; clear oil;
^{1}H NMR (500 MHz, $CDCl_3$, 55 °C) δ 1.41 (s, 9H), 4.53 (s, 2H), 5.12-5.17 (m, 4H), 5.76 (d, 1H *J* = 11.5 Hz), 6.58-6.68 (m, 2H), 7.22-7.28 (m, 5H);
^{13}C NMR (125 MHz, $CDCl_3$, 55 °C) δ 28.5, 52.8, 80.2, 115.6, 119.9, 127.4, 128.4, 128.8, 129.1, 131.2, 131.4, 135.7, 139.0, 155.3;
IR (thin film) cm^{-1} 2979m, 1694s, 1384m, 1167s;
mass spectrum (APCI): m/e (% relative intensity) 186 (100) $(M+H\text{-}Boc)^+$.

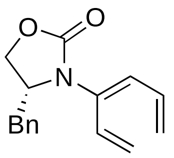

22

Acid-catalyzed condition: 3-Amido-triene **22** (44.0 mg, 0.17 mmol) was isolated in 89% yield according to general procedure 3.

22: R_f = 0.20 [25% EtOAc/hexanes]; $[\alpha]_D^{23}$ = +1.33 ^{o} [c 0.015, benzene]; colorless oil;
δ ^{1}H NMR (500 MHz, $CDCl_3$) δ 2.62 (ddd, 1H, *J* =4.5, 9.0, 15.0 Hz), 3.08 (dd, 1H, *J* = 3.5, 13.5 Hz), 4.15 (dt, 1H, *J* = 4.0, 10.0 Hz), 4.24-4.30 (m, 2H), 5.39 (ddd, 4H, *J* = 10.0, 18.5, 20.0 Hz), 6.29 (d, 1H, *J* = 11.0 Hz); 6.66-6.80 (m, 2H), 7.12 (d, 2H, *J* = 7.5 Hz), 7.23-7.32 (m, 3H);
^{13}C NMR (125 MHz, $CDCl_3$) δ 39.3, 58.3, 67.4, 117.7, 121.7, 128.4, 129.2, 129.2, 131.0, 131.6, 132.7, 135.7, 156.7;
IR (neat) cm^{-1} 3092w, 2365w, 1759s, 1480m, 1456m, 1412m, 1397w;
mass spectrum (APCI): m/e (% relative intensity) 256.2 (100) $(M+H)^+$.

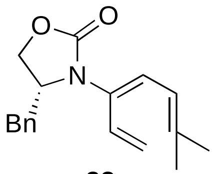

23a

Acid-catalyzed condition: 3-Amido-triene **23a** was isolated in 89% yield according to general procedure 3.

23a: R_f = 0.18 [25% EtOAc/hexanes]; $[\alpha]_D^{23}$ = +8.0^o [c 0.002, benzene]; colorless oil;
δ ^{1}H NMR (400 MHz, $CDCl_3$) δ 1.82 (s, 3H), 1.89 (s, 3H), 2.61 (dd, 1H, *J* =2.0, 10.4, 13.6 Hz), 3.09 (dd, 1H, *J* = 3.6, 13.6 Hz), 4.09-4.15 (m, 1H), 4.21-4.30 (m, 2H), 5.27 (d, 1H, *J* = 3.6 Hz), 5.30 (d, 1H, *J* = 5.2 Hz), 6.26 (dp, 1H, *J* =1.2, 11.6 Hz), 6.45 (d, 1H, *J* = 12.0 Hz); 6.73 (dd, 1H, *J* = 10.8, 17.6 Hz), 7.10 (d, 2H, *J* = 8.4 Hz), 7.22-7.32 (m, 3H);
^{13}C NMR (125 MHz, $CDCl_3$) δ 18.9, 26.9, 39.4, 58.2, 67.4, 115.7, 119.4, 127.3, 128.4, 128.5, 129.1, 129.2, 129.4, 135.9, 141.3, 157.1;
IR (neat) cm^{-1} 3032w, 2913w, 1754s, 1639w, 1574w, 1500w, 1456w, 1403m;
mass spectrum (APCI): m/e (% relative intensity) 284.1 (100) $(M+H)^+$.

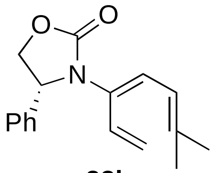

23b

Acid-catalyzed condition: Triene **23b** (85.0 mg, 0.32 mmol) was prepared in 89% yield according to general procedure 3.

23b: R_f = 0.22 [25% EtOAc/hexanes]; $[\alpha]_D^{23}$ = -14.7^o [c 0.018, benzene]; white wax;
^{1}H NMR (500 MHz, $CDCl_3$) δ ^{1}H NMR (500 MHz, $CDCl_3$) δ 1.55 (s, 3H), 1.75 (s, 3H), 4.35 (dd, 1H, *J* = 6.5, 8.5 Hz), 4.75 (t, 1H, *J* = 8.5 Hz), 5.04 (dd, 1H, *J* = 6.5, 9.0 Hz), 5.26 (dd, 2H, *J* = 11.0, 17.0 Hz), 6.03 (s, 2H), 6.53 (dd, 1H, *J* = 11.0, 17.0 Hz), 7.31-7.39 (m, 5H);
^{13}C NMR (125 MHz, $CDCl_3$) δ 18.6, 26.8, 61.8, 70.0, 115.3, 119.3, 127.7, 128.2, 128.6, 129.2, 129.3, 129.4, 138.7, 141.0, 157.2;
mass spectrum (APCI): m/e (% relative intensity) 270.1 (30) $(M+H)^+$.

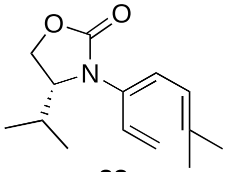

23c

Acid-catalyzed condition: Triene **23c** (154.0 mg, 0.66 mmol) was prepared in 91% yield according to general procedure 3.

23c: R_f = 0.16 [25% EtOAc/hexanes]; $[\alpha]_D^{23}$ = +28.9^o [c 0.022, benzene]; colorless oil;
^{1}H NMR (400 MHz, $CDCl_3$) δ 0.88 (d, 3H, *J* = 6.8 Hz), 0.93 (d, 3H, *J* = 6.8 Hz), 1.80 (s, 3H), 1.88 (s, 3H), 1.88-1.95 (m, 1H), 4.01 (dt, 1H, *J* = 3.6, 8.8 Hz), 4.17 (dd, 1H, *J* = 5.6, 8.8 Hz), 4.36 (t, 1H, *J* = 9.2 Hz), 5.26 (dd, 2H, *J* = 4.0, 11.2 Hz), 6.23 (dd, 1H, *J* = 1.2, 11.6 Hz), 6.42 (d, 1H, *J* =11.6 Hz), 6.63 (dd, 1H, *J* = 10.8, 17.2 Hz);
^{13}C NMR (125 MHz, $CDCl_3$) δ 14.9, 18.2, 18.8, 26.9, 29.1, 61.0, 63.5, 115.8, 119.5, 128.0, 128.2, 129.8, 140.9, 157.7;
mass spectrum (APCI): m/e (% relative intensity) 284.1 (100) $(M+H)^+$.

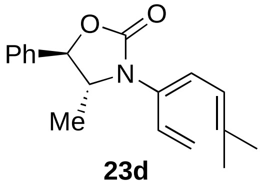

23d

Acid-catalyzed condition: Triene **23d** (25.3 mg, 0.089 mmol) was prepared in 74% yield according to general procedure 3.

23d: R_f = 0.40 [25% EtOAc/hexanes]; $[\alpha]_D^{23}$ = +18.0° [c 0.004, benzene]; white wax;

^{1}H NMR (500 MHz, $CDCl_3$) δ 0.73 (d, 3H, J = 6.5 Hz), 1.80 (s, 3H), 1.87 (s, 3H), 4.35 (dt, 1H, J = 7.0, 13.5 Hz), 5.26 (d, 1H, J = 10.0 Hz), 5.35 (d, 1H, J = 17.5 Hz), 5.74 (d, 1H, J = 8.0 Hz), 6.22 (dt, 1H, J = 1.5, 12.0 Hz), 6.36 (d, 1H, J =11.5 Hz), 6.72 (dd, 1H, J = 10.5, 17.0 Hz) 7.26-7.43 (m, 5H);

^{13}C NMR (125 MHz, $CDCl_3$) δ 15.5, 18.9, 27.0, 57.3, 78.9, 115.5, 119.5, 126.3, 128.4, 128.7, 128.8, 129.5, 135.5, 141.3, 156.8;

mass spectrum (APCI): m/e (% relative intensity) 258.1 (40) $(M+H)^+$.

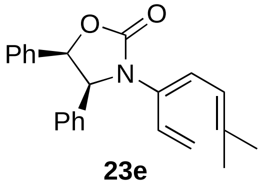

23e

Acid-catalyzed condition: Triene **23e** (20.8 mg, 0.059 mmol) was prepared in 89% yield according to general procedure 3.

23e: R_f = 0.37 [25% EtOAc/hexanes]; white wax;

^{1}H NMR (500 MHz, $CDCl_3$) δ 1.55 (s, 3H), 1.80 (s, 3H), 1.75 (s, 3H), 5.29 (d, 1H, J = 8.5 Hz), 5.31 (d, 1H, J = 12.0 Hz), 5.44 (d, 1H, J = 17.0 Hz), 6.00 (d, 1H, J = 8.5 Hz), 6.04 (dt, 1H, J = 1.0, 12.0 Hz), 6.21 (d, 1H, J = 11.5 Hz), 6.58 (dd, 1H, J = 11.0, 17.0 Hz), 6.92-6.97 (m, 2H), 7.04-7.16 (m, 8H);

^{13}C NMR (125 MHz, $CDCl_3$) δ 18.6, 26.8, 66.8, 79.9, 115.5, 119.4, 126.2, 127.7, 128.1, 128.2, 128.4, 128.4, 128.5, 128.6, 129.8, 134.9, 135.2, 140.9, 157.1;

mass spectrum (APCI): m/e (% relative intensity) 346.9 (40) $(M+H)^+$.

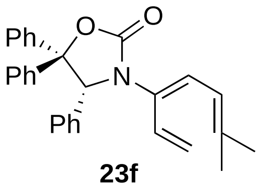

23f

Acid-catalyzed condition: Triene **23f** (160.0 mg, 0.38 mmol) was prepared in 86% yield according to general procedure 3.

23f: R_f = 0.48 [25% EtOAc/hexanes]; $[\alpha]_D^{23}$ = +49.4^o [c 0.055, benzene]; white wax;
^{1}H NMR (500 MHz, $CDCl_3$) δ 1.43 (s, 3H), 1.71 (s, 3H), 4.61 (d, 1H, *J* = 17.0 Hz), 4.94 (d, 1H, *J* = 11.0 Hz), 5.57 (s, 1H), 5.98 (t, 1H, *J* = 13.0 Hz), 6.45 (dd, 1H, *J* = 11.0, 17.5 Hz), 6.98-7.06 (m, 3H), 7.10-7.15 (m, 5H), 7.21 (dd, 2H, *J* = 1.5, 8.5 Hz), 7.34 (t, 1H, *J* = 5.5 Hz), 7.42 (t, 2H, *J* = 7.5 Hz), 7.80 (d, 2H, *J* = 8.5 Hz);
^{13}C NMR (125 MHz, $CDCl_3$) δ 18.5, 26.8, 72.2, 88.1, 115.4, 119.3, 125.5, 126.5, 127.4, 128.0, 128.3, 128.5, 128.6, 128.7, 129.1, 129.2, 129.5, 136.9, 139.7, 141.1, 144.4, 156.0;
mass spectrum (APCI): m/e (% relative intensity) 395.3 (40) $(M+H)^+$.

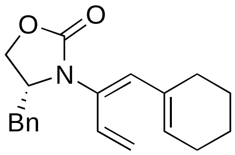

24

Acid-catalyzed condition: Triene **24** (16.0 mg, 0.05 mmol) was prepared in 95% yield according to general procedure 3.

24: R_f = 0.09 [25% EtOAc/hexanes]; clear oil;
^{1}H NMR (500 MHz, $CDCl_3$) δ 1.58-1.72 (m, 4H), 2.15-2.24 (m, 4H), 2.61 (dd, 1H, *J* = 10.0, 13.5 Hz), 3.08 (dd, 1H, *J* = 3.0, 13.5 Hz), 4.05-4.18 (m, 1H), 4.22-4.31 (m, 2H), 5.23 (d, 1H, *J* = 11.0 Hz), 5.27 (d, 1H, *J* = 18.0 Hz), 5.89 (m, 1H), 6.01 (s, 1H), 6.77 (dd, 1H, *J* = 11.0, 18.0 Hz), 7.12 (d, 2H, *J* = 7.0 Hz), 7.24 (t, 1H, *J* = 7.5 Hz), 7.30 (t, 2H, *J* = 7.0 Hz);
^{13}C NMR (125 MHz, $CDCl_3$) δ 22.0, 22.9, 26.3, 29.1, 39.4, 58.0, 67.4, 115.6, 127.3, 129.1, 129.2, 130.0, 130.2, 133.3, 133.6, 135.9, 136.1, 157.0;
mass spectrum (APCI): m/e (% relative intensity) 311 (100) $(M+H)^+$.

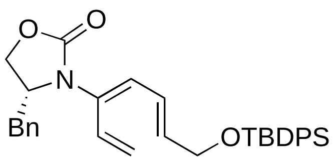

25a

Acid-catalyzed condition: Triene **25a** (250.5 mg, 0.48 mmol) was prepared in 84% yield according to general procedure 3.

25a: R_f = 0.23 [25% EtOAc/hexanes]; $[\alpha]_D^{23}$ = +8.7° [c 0.030, benzene]; colorless oil;
^{1}H NMR (500 MHz, C_6D_6) δ 1.09 (s, 9H), 2.61 (dd, 1H, *J* = 3.0, 10.5 Hz), 3.09 (dd, 1H, *J* = 3.5, 14.0 Hz), 4.13 (ddd, 1H, *J* = 4.5, 9.0, 16.0 Hz), 4.21-4.29 (m, 2H), 4.33 (d, 2H, *J* = 1.5 Hz), 5.40 (d, 1H, *J* = 4.5 Hz), 5.36 (s, 1H), 5.95 (dt, 1H, *J* = 4.5, 15.0 Hz), 6.31 (d, 1H, *J* = 11.5 Hz), 6.68 (dd, 1H, *J* = 10.5, 17.0 Hz), 6.81 (tt, 1H, *J* = 2.0, 13.5 Hz), 7.11 (d, 2H, *J* = 7.0 Hz), 7.24 (t, 1H, *J* = 7.0 Hz), 7.31 (d, 2H, *J* = 7.0 Hz), 7.35-7.45 (m, 6H), 7.69 (dt, 4H, *J* = 2.0, 8.0 Hz);
^{13}C NMR (100 MHz, $CDCl_3$) δ 14.4, 19.6, 23.0, 27.1, 31.9, 39.4, 58.4, 64.2, 67.4, 116.9, 123.4, 127.4, 128.1, 128.4, 129.2, 129.3, 130.1, 131.1, 131.5, 133.6, 133.7, 135.8, 135.8, 137.2, 156.9;
mass spectrum (APCI): m/e (% relative intensity) 538.8 (10) $(M+H)^+$.

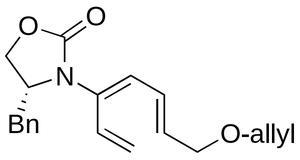

25b

Acid-catalyzed condition: Triene **25b** (135.8 mg, 0.42 mmol) was prepared in 75% yield according to general procedure 3.

25b: R_f = 0.43 [40% EtOAc/hexanes]; $[\alpha]_D^{23}$ = +7.4° [c 0.015, benzene]; white wax;
^{1}H NMR (400 MHz, C_6D_6) δ 2.62 (dd, 1H, *J* = 10.4, 14.0 Hz), 3.01 (dd, 1H, *J* = 3.6, 16.5 Hz), 4.02 (dt, 1H, *J* = 1.6, 6.0 Hz), 4.10-4.16 (m, 3H), 4.22-4.31 (m, 2H), 5.22 (dq, 1H, *J* = 1.2, 10.4 Hz), 5.28-5.29 (m, 1H), 5.38 (d, 1H, *J* = 9.6 Hz), 5.88-6.00 (m, 2H), 6.30 (d, 1H, *J* = 11.6 Hz), 6.66-6.74 (m, 2H), 7.11 (d, 2H, *J* = 6.8 Hz), 7.22-7.32 (m, 3H);
^{13}C NMR (100 MHz, $CDCl_3$) δ 14.3, 22.9, 31.8, 39.3, 58.3, 67.3, 70.2, 71.6, 117.3, 117.5, 126.0, 127.4, 128.3, 129.1, 129.2, 130.6, 132.2, 134.4, 134.8, 135.7, 156.7;
mass spectrum (APCI): m/e (% relative intensity) 326.2 (100) $(M+H)^+$.

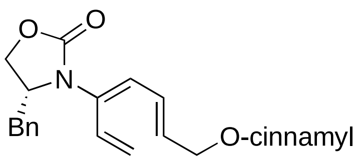

25c

Acid-catalyzed condition: Triene **25c** (70.0 mg, 0.17 mmol) was prepared in 54% yield according to general procedure 3.

25c: R_f = 0.38 [40% EtOAc/hexanes]; white wax;
^{1}H NMR (500 MHz, C_6D_6) δ 2.62 (dd, 1H, *J* = 10.0, 14.5 Hz), 3.07 (dd, 1H, *J* = 3.5, 13.5 Hz), 4.10-4.32 (m, 7H), 5.38 (dt, 2H, *J* = 11.5, 17.5 Hz), 6.01 (dt, 1H, *J* = 5.5, 15.0 Hz), 6.27-6.33 (m, 2H), 6.62 (d, 1H, *J* = 16.0 Hz), 6.68-6.75 (m, 2H), 7.11 (d, 2H, *J* = 6.5 Hz), 7.24-7.35 (m, 6H), 7.41 (dd, 2H, *J* = 1.5, 8.5 Hz);
^{13}C NMR (125 MHz, $CDCl_3$) δ 39.3, 58.3, 67.4, 70.3, 71.3, 117.5, 126.1, 126.2, 126.8, 127.4, 128.0, 128.4, 128.9, 129.2, 129.3, 130.6, 132.3, 134.4, 135.7, 136.9, 156.8 *(one sp^2 carbon missing due to overlap)*;
mass spectrum (APCI): m/e (% relative intensity) 402.1 (100) $(M+H)^+$.

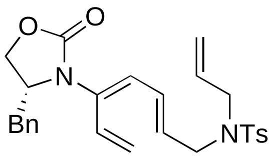

26

Acid-catalyzed condition: Triene **26** (70.0 mg, 0.19 mmol) was prepared in 62% yield according to general procedure 3.

26: R_f = 0.27 [40% EtOAc/hexanes]; $[\alpha]_D^{23}$ = +17.9° [c 0.011, benzene]; white wax;
^{1}H NMR (400 MHz, C_6D_6) δ 2.43 (s, 3H), 2.62 (dd, 1H, *J* = 9.5, 13.5 Hz), 3.04 (dd, 1H, *J* = 3.5, 13.5 Hz), 3.82 (dd, 2H, *J* = 6.5 Hz), 3.92 (d, 2H, *J* = 6.0 Hz), 4.12 (dd, 1H, *J* = 5.0, 8.0 Hz), 4.22-4.30 (m, 2H), 5.22 (dt, 2H, *J* = 1.5, 7.5 Hz), 5.38 (dd, 2H, *J* = 7.0, 11.0 Hz), 5.59-5.70 (m, 2H), 6.17 (d, 1H, *J* = 11.0 Hz), 6.49 (dd, 1H, *J* = 11.5, 15.5 Hz), 6.57 (dd, 1H, *J* = 11.0, 17.5 Hz), 7.00 (d, 2H, *J* = 7.0 Hz), 7.24-7.32 (m, 5H), 7.70 (d, 2H, *J* = 8.5 Hz),;
^{13}C NMR (100 MHz, $CDCl_3$) δ 14.4, 21.8, 22.9, 31.9, 39.3, 48.7, 50.0, 58.2, 67.3, 117.9, 119.5, 127.5, 127.7, 128.2, 129.2, 129.3, 129.8, 130.1, 132.1, 132.6, 132.9, 132.1, 132.6, 135.6, 137.6, 143.7, 156.7;
mass spectrum (APCI): m/e (% relative intensity) 479.2 (20) $(M+H)^+$.

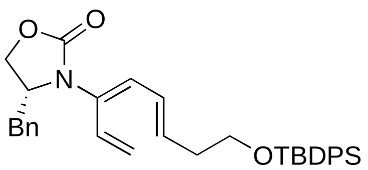

27

Acid-catalyzed condition: Amido triene **27** (102 mg, 0.190 mmol) was prepared in 72% yield according to general procedure 3.

27: R_f = 0.25 [1:4 EtOAc/hexanes]; clear oil;

^{1}H NMR (500 MHz, $CDCl_3$) δ 1.05 (s, 9H), 2.42 (q, 2H, *J* = 6.5 Hz), 2.59 (dd, 1H, *J* = 10.0, 13.5 Hz), 3.05 (dd, 1H, *J* = 3.0, 14.0 Hz), 3.74 (t, 2H, *J* = 6.5 Hz), 4.12 (dd, 1H, *J* = 5.5, 8.5 Hz), 4.22 (m, 1H), 4.26 (q, 1H, *J* = 8.0 Hz), 5.29 (d, 1H, *J* = 5.5 Hz), 5.32 (s, 1H), 5.91 (dt, 1H, *J* = 7.0, 14.5 Hz), 6.21 (d, 1H, *J* = 11.5 Hz), 6.49 (dd, 1H, *J* = 11.5, 15.0 Hz), 6.68 (dd, 1H, *J* = 10.5, 17.0 Hz), 7.10 (d, 2H, *J* = 7.0 Hz), 7.21-7.31 (m, 3H), 7.34-7.44 (m, 6H), 7.64-7.70 (m, 4H);

^{13}C NMR (125 MHz, $CDCl_3$) δ 19.5, 27.1, 36.7, 39.4, 58.3, 63.5, 67.4, 116.3, 126.3, 127.4, 128.0, 128.4, 129.2, 129.3, 129.9, 130.5, 132.0, 134.1, 135.8, 135.9, 136.5, 156.9.

mass spectrum (APCI): m/e (% relative intensity) 332.1 (100) $(M+H)^+$.

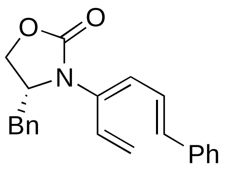

28

Acid-catalyzed condition: Triene **28** (32.4 mg, 0.098 mmol) was prepared in 72% yield according to general procedure 3.

28: R_f = 0.20 [25% EtOAc/hexanes]; $[\alpha]_D^{23}$ = +1.33 ^{o} [c 0.015, benzene]; white wax;

^{1}H NMR (500 MHz, $CDCl_3$) δ ^{1}H NMR (500 MHz, $CDCl_3$) δ 2.65 (ddd, 1H, *J* =3.0, 6.5, 15.0 Hz), 3.09 (dd, 1H, *J* = 3.5, 13.5 Hz), 4.15 (dt, 1H, *J* = 7.5, 13.0 Hz), 4.26-4.32 (m, 2H), 5.42 (ddd, 4H, *J* = 4.0, 11.0, 21.5 Hz), 6.45 (d, 1H, *J* = 11.5 Hz); 6.73 (bd, 1H, *J* = 15.5 Hz), 6.81 (dd, 1H, *J* = 10.5, 17.0 Hz), 7.13 (d, 2H, *J* = 7.0 Hz), 7.14-7.37 (m, 6H), 7.45 (d, 2H, *J* = 7.5 Hz); ^{13}C NMR (125 MHz, $CDCl_3$) δ 39.4, 58.4, 67.4, 117.4, 122.9, 127.1, 127.3, 127.5, 128.5, 128.6, 129.0, 129.2, 129.2, 129.3, 131.5, 132.2, 135.8, 136.5, 137.1, 156.8;

IR (neat) cm^{-1} 3057w, 2307w, 1753s, 1402m;

mass spectrum (APCI): m/e (% relative intensity) 538.4 (100) $(M+H)^+$.

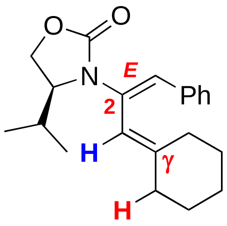

33a

Thermal condition: 2-Amido-diene **33a** (43.0 mg, 0.13 mmol) was isolated in 71% yield according to general procedure 2.

33a: R_f = 0.20 [25% EtOAc/hexanes]; $[\alpha]_D^{23}$ = -44.3° [c 0.003, benzene]; white solid; mp 102-104 ºC;
^{1}H NMR (500 MHz, $CDCl_3$) δ 0.87 (d, 3H, J = 7.0 Hz), 0.94 (d, 3H, J = 6.5 Hz), 1.24-1.38 (m, 2H), 1.38-1.45 (m, 1H), 1.48-1.64 (m, 3H), 1.80 (p, 1H, J =6.5 Hz), 1.93 (sep, 1H, J = 3.0 Hz), 2.04-2.26 (m, 3H), 3.97 (p, 1H, J = 3.5 Hz), 4.13 (dd, 1H, J = 5.0, 9.5 Hz), 4.26 (t, 1H, J = 9.0 Hz), 5.65 (s, 1H), 6.69 (s, 1H), 7.17 (t, 1H, J = 7.5 Hz), 7.26 (t, 1H, J = 7.0 Hz), 7.38 (d, 2H, J = 7.5 Hz);
^{13}C NMR (125 MHz, $CDCl_3$) δ 14.7, 18.0, 26.5, 26.9, 28.4, 29.5, 30.3, 37.1, 59.9, 62.6, 115.3, 126.7, 127.1, 128.3, 129.4, 130.8, 136.6, 148.7, 156.6;
IR (neat) cm^{-1} 2929w, 1744s, 1626w, 1485w, 1446m, 1400m, 1371m;
mass spectrum (APCI): m/e (% relative intensity) 326.2 (100) $(M+H)^+$.

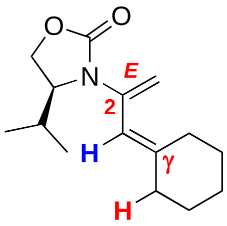

33b

2-Amido-diene **33b** was isolated in 90% yield during purification of allenamide **29b**.

33b: R_f = 0.46 [25% EtOAc/hexanes]; $[\alpha]_D^{23}$ = -120.5° [c 0.013, benzene]; colorless oil;
^{1}H NMR (400 MHz, $CDCl_3$) δ 0.88 (t, 6H, J = 6.8 Hz), 1.46-1.67 (m, 6H), 1.99 (dtt, 1H, J = 4.0, 7.2, 10.8 Hz), 2.17 (dd, 2H, J = 5.2, 6.0), 2.33 (ddd, 1H, J = 3.2, 8.8, 12.0 Hz), 2.44 (ddd, 1H, J = 4.4, 6.8, 13.6 Hz), 3.96 (dt, 1H, J =4.0, 8.0 Hz), 4.10 (dd, 1H, J = 4.4, 8.8 Hz), 4.24 (t, 1H, J =8.8 Hz), 4.92 (d, 1H, J = 1.2Hz), 5.37 (s, 1H), 5.57 (d, 1H, J = 0.8 Hz);
^{13}C NMR (100 MHz, $CDCl_3$) δ 14.6, 17.9, 26.7, 28.1, 28.8, 29.3, 29.9, 37.5, 60.3, 62.5, 109.3, 117.8, 137.3, 148.1, 156.2;
IR (neat) cm^{-1} 2928m, 2362w, 1750s, 1619w, 1447w, 1402m;
mass spectrum (APCI): m/e (% relative intensity) 250.2 (100) (M+H).

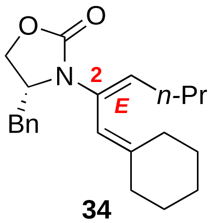

34

Thermal condition: 2-Amido-diene **34** (41.0 mg, 0.12 mmol) was synthesized according to the general procedure 2.

34: R_f = 0.50 [25% EtOAc/hexanes]; $[\alpha]_D^{23}$ = +131.1° [c 0.009, benzene]; colorless oil;
^{1}H NMR (500 MHz, $CDCl_3$) δ 0.84-0.92 (m, 2H), 0.94 (t, 3H, *J* = 7.0 Hz), 1.49 (dt, 2H, *J* = 7.5, 15.0 Hz), 1.53-1.71 (m, 4H), 2.01-2.25 (m, 3H), 2.24 (ddd, 3H, *J* = 4.5, 9.5, 15.5), 2.60 (ddd, 1H, *J* = 3.0, 6.5, 9.5 Hz), 3.05 (dd, 1H, *J* = 3.0, 13.5 Hz), 4.00 (dd, 1H, *J* =11.0, 20.5 Hz), 4.14 (ddd, 2H, *J* = 7.0, 8.0, 13.0 Hz), 5.50 (s, 1H), 5.77, (t, 1H, *J* = 7.5 Hz), 7.12 (d, 2H, *J* = 7.0 Hz), 7.26 (d, 1H, *J* = 6.5 Hz), 7.31 (t, 2H, *J* =7.0 Hz);
^{13}C NMR (100 MHz, $CDCl_3$) δ 14.2, 22.7, 26.7, 27.6, 28.8, 30.6, 30.7, 37.1, 39.3, 57.4, 66.4, 115.1, 127.3, 128.9, 129.2, 129.4, 129.4, 136.1, 148.0, 156.5;
IR (neat) cm^{-1} 2928m, 2360w, 1753s, 1647w, 1479w, 1449m, 1399m;
mass spectrum (APCI): m/e (% relative intensity) 340.2 (100) (M+H).

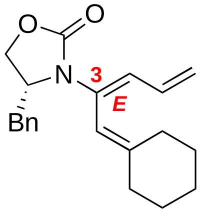

35a

Acid-catalyzed condition: Amido triene **35a** (102.0 mg, 0.32 mmol) was prepared in 68% yield according to general procedure 3.
35a: R_f = 0.30 [1:4 EtOAc/hexanes]; clear oil;
^{1}H NMR (500 MHz, $CDCl_3$) δ 0.84-0.90 (m, 1H), 1.26-1.32 (m, 1H), 1.45-1.53 (m, 1H), 1.54-1.66 (m, 2H), 1.69-1.75 (m, 2H), 2.10-2.15 (m, 1H), 2.26-2.34 (m, 2H), 2.65 (dd, 1H, *J* = 9.5, 13 Hz), 3.04 (dd, 1H, *J* = 3.5, 13.5 Hz), 4.02-4.06 (m, 1H), 4.17 (t, 1H, *J* = 8.5 Hz), 4.18-4.26 (m, 1H), 5.14 (dt, 1H, *J* = 1, 10.5 Hz), 5.31 (dd, 1H, *J* = 1.5, 16.5 Hz), 5.62 (s, 1H), 6.41 (dt, 1H, *J* = 10, 21 Hz), 6.53 (d, 1H, *J* = 11 Hz), 7.12 (d, 2H, *J* = 7 Hz), 7.24-7.27 (m, 1H), 7.29-7.33 (m, 2H);
^{13}C NMR (125 MHz, $CDCl_3$) δ 14.4, 22.9, 26.6, 27.5, 28.8, 30.8, 31.9, 37.2, 39.4, 57.7, 115.3, 117.8, 124.9, 127.5, 129.2, 129.4, 131.7, 133.7, 135.9, 149.8, 155.7;
mass spectrum (APCI): m/e (% relative intensity) 324.1 (10) (M+H).

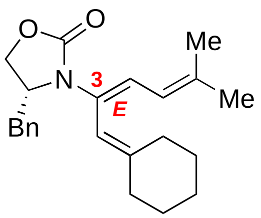

35b

Acid-catalyzed condition: Amido triene **35b** (20.8 mg, 0.059 mmol) was prepared in 80% yield according to general procedure 3.

35b: R_f = 0.40 [1:4 EtOAc/hexanes]; clear oil;

^{1}H NMR (500 MHz, $CDCl_3$) δ 1.47-1.72 (m, 6H), 1.81 (s, 3H), 1.83 (s, 3H), 2.08 (dt, 1H, *J* = 3.5, 9.5 Hz), 2.22-2.30 (m, 3H), 2.65 (dd, 1H, *J* = 10.0, 13.5 Hz), 3.04 (dd, 1H, *J* = 3.0, 13.5 Hz), 4.02 (dd, 1H, *J* = 4.0, 7.5 Hz), 4.15-4.20 (m, 2H), 5.59 (s, 1H), 5.86 (dt, 1H, *J* = 1.5, 11.5 Hz), 6.60 (d, 1H, *J* = 11.5 Hz), 7.12 (d, 2H, *J* = 7.0 Hz), 7.24-7.27 (m, 1H), 7.29-7.33 (m, 2H);

^{13}C NMR (125 MHz, $CDCl_3$) δ 19.0, 26.7, 27.6, 28.9, 30.8, 37.3, 39.4, 57.8, 66.2, 115.5, 121.8, 122.9, 127.4, 128.3, 129.2. 129.4, 136.1, 137.1, 148.9, 156.2;

mass spectrum (APCI): m/e (% relative intensity) 351.1 (10) (M+H).

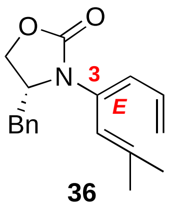

36

Acid-catalyzed condition: Triene **36** (52 mg, 0.18 mmol) was isolated in 84% yield according to general procedure 3.

36: R_f = 0.23 [1:4 EtOAc/hexanes]; $[\alpha]_D^{23}$ = +9.18° [c 0.0476, benzene]; clear oil;

^{1}H NMR (400 MHz, $CDCl_3$) δ 1.74 (d, 3H, *J* = 1.2 Hz), 1.93 (d, 3H, *J* = 1.6 Hz), 2.63 (ddd, 1H, *J* = 2.0, 8.0, 13.6 Hz), 2.99 (dd, 1H, *J* = 3.2, 13.6 Hz), 4.02-4.08 (m, 1H), 4.14-4.22 (m, 2H), 5.14 (dd, 1H, *J* = 2.0, 10.0 Hz), 5.31 (dd, 1H, *J* = 1.2, 16.8 Hz), 5.70 (brs, 1H), 6.32 (ddd, 1H, *J* = 10.0, 11.2, 16.8 Hz), 6.48 (d, 1H, *J* = 11.2 Hz), 7.09 (m, 2H), 7.24-7.35 (m, 3H);

^{13}C NMR (100 MHz, $CDCl_3$) δ 20.4, 26.0, 39.3, 57.7, 66.1, 117.9, 118.5, 124.7, 127.4, 129.2, 129.3, 132.2, 133.6, 135.9, 142.3, 155.7;

mass spectrum (APCI): m/e (% relative intensity) 284.2 (100) $(M+H)^+$.

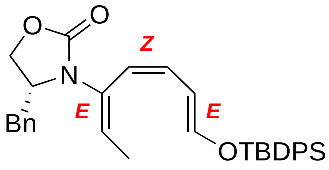

39a

Acid-catalyzed condition: Triene **39a** (40.9 mg, 0.078 mmol) was prepared in 74% yield according to general procedure 3.

39a: R_f = 0.20 [25% EtOAc/hexanes]; $[\alpha]_D^{23}$ = -8.0° [c 0.004, benzene]; colorless oil;

^{1}H NMR (500 MHz, C_6D_6) δ 0.76 (s, 9H), 1.28 (dd, 3H, *J* = 1.0, 7.5 Hz), 1.79 (dd, 1H, *J* = 10.5, 13.5 Hz), 2.83 (dd, 1H, *J* = 3.5, 13.5 Hz), 3.15-3.23 (m, 2H), 3.35 (ddd, 1H, *J* = 4.5, 8.5, 13.0 Hz), 5.02 (d, 1H, *J* = 11.5 Hz), 5.50 (t, 1H, *J* = 11.5 Hz), 5.68 (dd, 1H, *J* = 7.5, 14.5 Hz), 5.89 (t, 1H, *J* = 12.0 Hz), 6.42 (d, 1H, *J* = 12.0 Hz), 6.48 (d, 2H, *J* = 7.0 Hz), 6.73-6.87 (m, 11H), 7.37-7.40 (m, 4H);

^{13}C NMR (100 MHz, $CDCl_3$) δ 14.2, 19.1, 19.4, 26.6, 27.1, 39.2, 57.6, 66.5, 110.9, 117.3, 124.3, 127.2, 128.0, 128.1, 128.2, 129.0, 129.2, 129.2, 129.4, 130.0, 130.4, 130.5, 132.1, 132.2, 135.6, 135.6, 135.7, 136.2, 149.1, 156.2;

mass spectrum (APCI): m/e (% relative intensity) 258.2 (20) $(M+H)^+$.

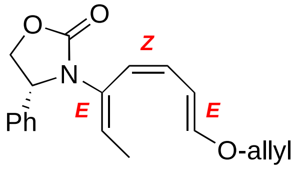

39b

Acid-catalyzed condition: Triene **39b** (77.6 mg, 0.25 mmol) was prepared in 55% yield according to general procedure 3.

39b: R_f = 0.10 [25% EtOAc/hexanes]; colorless oil;

^{1}H NMR (500 MHz, $CDCl_3$) δ 1.55 (dd, 3H, *J* = 1.0, 7.0 Hz), 4.05 (dd, 2H, *J* = 0.5, 5.5 Hz), 4.11 (dd, 1H, *J* = 7.0, 8.5 Hz), 4.63 (t, 1H, *J* = 8.5 Hz), 4.93 (dd, 1H, *J* = 7.0, 9.0 Hz), 5.11 (t, 1H, *J* = 12.5 Hz), 5.23 (dq, 1H, *J* = 1.0, 10.5 Hz), 5.28 (dq, 1H, *J* = 1.5, 17.0 Hz), 5.37 (d, 1H, *J* = 11.0 Hz), 5.66 (q, 1H, *J* = 7.5 Hz), 5.87 (tq, 1H, *J* = 5.5, 11.0 Hz), 6.04 (t, 1H, *J* = 11.0 Hz), 6.59 (d, 1H, *J* = 12.5 Hz), 7.20-7.25 (m, 2H), 7.28-7.42 (m, 3H);

^{13}C NMR (125 MHz, $CDCl_3$) δ 14.1, 60.8, 69.9, 70.3, 103.8, 116.9, 118.2, 124.7, 127.6, 128.8, 129.2, 129.4, 130.7, 130.9, 133.0, 138.9, 152.9;

mass spectrum (APCI): m/e (% relative intensity) 162.1 (25) $(Aux+H)^+$.

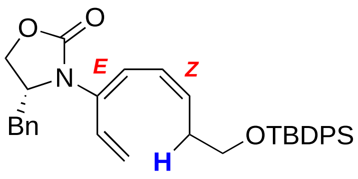

41

Acid-catalyzed condition: Amido triene **41** (65.3 mg, 0.121 mmol) was prepared in 65% yield according to general procedure 3 (CSA-catalyzed isomerization from **40**).

41: R_f = 0.24 [1:4 EtOAc/hexanes]; clear oil;

^{1}H NMR (400 MHz, $CDCl_3$) δ 1.04 (s, 9H), 2.50 (m, 2H), 2.55 (dd, 1H, *J* = 9.6, 13.6 Hz), 3.01 (dd, 1H, *J* = 3.6, 13.6 Hz), 3.73 (m, 2H), 4.12 (dd, 1H, *J* = 5.6, 8.0 Hz), 4.18-4.30 (m, 2H), 5.33 (d, 1H, *J* = 7.6 Hz), 5.37 (s, 1H), 5.75 (dq, 1H, *J* = 2.0, 7.6 Hz), 6.47 (m, 2H), 6.69 (dd, 1H, *J* = 10.8, 16.8 Hz), 7.06-7.09 (m, 2H), 7.22-7.31 (m, 3H), 7.33-7.43 (m, 6H), 7.64-7.69 (m, 4H);

^{13}C NMR (100 MHz, $CDCl_3$) δ 19.4, 27.1, 31.7, 39.3, 58.3, 63.4, 67.4, 117.2, 123.9, 126.5, 127.3, 127.9, 128.3, 129.1, 129.2, 129.9, 132.1, 133.1, 134.0, 135.8.

mass spectrum (APCI): m/e (% relative intensity) 514.2 (100) $(M\text{-}CO+H_2)^+$.

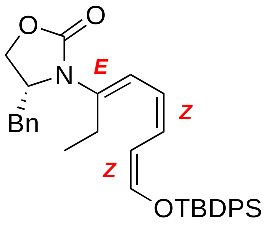

43

Thermal condition: Amido triene **43** (26.7 mg, 0.050 mmol) was prepared in 45% yield according to general procedure 2 (thermal isomerization from **40**).

43: R_f = 0.27 [1:4 EtOAc/hexanes]; clear oil;

^{1}H NMR (400 MHz, $CDCl_3$) δ 1.04-1.14 (m, 12H), 2.52 (dq, 1H, *J* = 7.2, 14.4 Hz), 2.60 (dq, 1H, *J* = 7.2, 14.4 Hz), 2.64 (dd, 1H, *J* = 10.0, 13.6 Hz), 3.20 (dd, 1H, *J* = 3.6, 13.6 Hz), 4.11 (dd, 1H, *J* = 5.6, 8.8 Hz), 4.24 (t, 1H, *J* = 8.4 Hz), 4.31 (m, 1H), 5.89 (t, 1H, *J* = 11.2 Hz), 5.97 (t, 1H, *J* = 11.2 Hz), 6.29 (t, 1H, *J* = 11.6 Hz), 6.36 (d, 1H, *J* = 11.2 Hz), 6.63 (d, 1H, *J* = 11.6 Hz), 7.15-7.19 (m, 2H), 7.25-7.48 (m, 9H), 7.64-7.74 (m, 4H);

^{13}C NMR (100 MHz, $CDCl_3$) δ 12.9, 19.5, 21.3, 26.7, 39.0, 57.6, 66.7, 110.0, 118.5, 119.1, 126.8, 127.4, 128.2, 128.2, 129.3, 130.4, 132.5, 135.6, 135.8, 136.2, 147.0, 155.9.

mass spectrum (APCI): m/e (% relative intensity) 514.2 (80) $(M\text{-}CO+H_2)^+$.

General procedure 4 for the electrocyclic ring-closure

A solution of the respective allenamide or triene in anhydrous CH_3CN (0.1 M) in a sealed tube was heated to 135 °C. Upon completion of the reaction (16 h), the solution was cooled to RT and solvent removed in vacuo to afford the crude product. Separation and purification of the resulting crude residue by silica gel flash column chromatography (gradient eluent: EtOAc in hexane) afforded the desired 2-amido-1,3-cyclohexadienes.

Characterizations of cyclic 2-amido-dienes.

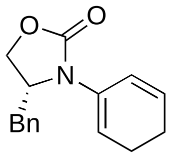

44a

Cyclic 2-amido-diene **44a** (120.0 mg, 0.46 mmol) was isolated in 84% yield from the 3-amido-triene **22** (143.0 mg, 0.55 mmol) according to the general procedure 4.

Cyclic 2-amido-diene **44a** (14.0 mg, 0.053 mmol) was isolated in 43% yield from the allenamide **15a** (32.0 mg, 0.14 mmol) in a tandem sequence according to general procedure 4.

44a: R_f = 0.22 [25% EtOAc/hexanes]; $[\alpha]_D^{23}$ = +25.0 ^{o} [c 0.004, benzene]; colorless oil;
^{1}H NMR (500 MHz, $CDCl_3$) δ ^{1}H NMR (500 MHz, $CDCl_3$) δ 2.17-2.40 (m, 4H), 2.71 (dd, 1H, J = 9.0, 13.5 Hz), 3.16 (dd, 1H, J = 4.0, 14.0 Hz), 4.11 (dd, 1H, J = 5.5, 8.5 Hz), 4.24 (t, 1H, J =8.5 Hz)), 4.28-4.37 (m, 1H), 5.71 (dt, 1H, J = 1.5, 5.0 Hz), 6.02 (dt, 1H, J = 5.0, 9.5 Hz); 6.17 (dq, 1H, J = 1.5, 9.5 Hz), 7.14 (d, 2H, J = 6.5 Hz), 7.27 (d, 1H, J = 1.5, 7.5), 7.31 (t, 2H, J = 7.0);
^{13}C NMR (125 MHz, $CDCl_3$) δ 22.0, 22.0, 38.3, 57.2, 66.6, 116.1, 122.9, 127.4, 129.2, 129.2, 129.4, 129.5, 131.8, 135.8, 155.9;
IR (neat) cm^{-1} 2926w, 2361w, 1748s, 1600w, 1498w, 1479w, 1405m;
mass spectrum (APCI): m/e (% relative intensity) 254.1 (100) $(M+H)^+$.

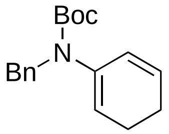

45

Cyclic 2-amido-diene **45** (11.0 mg, 0.04 mmol) was isolated in 35% yield from 3-amido-triene **14** (31.0 mg, 0.11 mmol) according to general procedure 4.

45: R_f = 0.33 [17% EtOAc/hexanes]; pale yellow oil;
^{1}H NMR (500 MHz, $CDCl_3$) δ 1.42 (s, 9H), 2.09-2.17 (m, 4H), 4.59 (s, 2H), 5.43 (brs, 1H), 5.76-5.82 (m, 2H), 7.23-7.31 (m, 5H);
^{13}C NMR (125 MHz, $CDCl_3$, 55 ^{o}C) δ 21.9, 22.2, 28.5, 53.1, 80.3, 118.7, 126.8, 127.0, 127.1, 127.8, 128.5, 138.2, 139.3, 154.9;
IR (thin film) cm^{-1} 3031m, 1698s, 1387m, 1165s;
mass spectrum (APCI): m/e (% relative intensity) 186 (100) (M+H-Boc)$^+$.

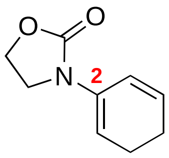

44b

Cyclic 2-amido-diene **44b** (23.0 mg, 0.14 mmol) was isolated in 57% yield from the allenamide **15b** in a tandem sequence according to general procedure 4.

44b: R_f = 0.39 [40% EtOAc/hexanes]; colorless oil;
^{1}H NMR (500 MHz, $CDCl_3$) δ 2.11-2.28 (m, 4H), 3.79 (t, 2H, J = 9.0 Hz), 4.39 (t, 2H, J = 8.5 Hz), 5.27 (t, 1H, J = 4.0 Hz), 5.99 (dt, 1H, J = 4.5, 9.0 Hz), 6.58 (dt, 1H, J = 2.0, 12.0 Hz);
^{13}C NMR (125 MHz, $CDCl_3$) δ 22.8, 23.2, 45.5, 61.6, 106.5, 122.6, 123.7, 136.7, 154.8;
IR (neat) cm^{-1} 2922w, 1737s, 1649w, 1599w, 1479w, 1405m;
mass spectrum (APCI): m/e (% relative intensity) 166.2 (100) (M-H_2+H)$^+$.

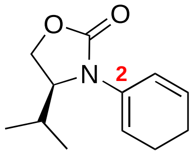

44c

Cyclic 2-amido-diene **44c** (39.0 mg, 0.19 mmol) was isolated in 54% yield from the allenamide **15c** in a tandem sequence according to general procedure 4.

44c: R_f = 0.20 [25% EtOAc/hexanes]; $[\alpha]_D^{23}$ = -27.1 ^{o} [c 0.009, benzene]; colorless oil;

^{1}H NMR (500 MHz, $CDCl_3$) δ 0.88 (t, 6H, *J* = 7.0 Hz), 2.11 (sep, 1H, *J* =3.5 Hz), 2.16-2.38 (m, 4H), 4.06 (ddd, 1H, *J* = 3.5, 8.5, 12.5 Hz), 4.11 (dd, 1H, *J* = 5.5, 9.0 Hz), 4.29 (t, 1H, *J* = 9.0 Hz), 5.67 (ddd, 1H, *J* = 1.0, 1.5, 4.5 Hz), 5.98 (dt, 1H, *J* = 4.0, 9.0 Hz), 6.05 (dt, 1H, *J* = 2.0, 10.0 Hz);

^{13}C NMR (125 MHz, $CDCl_3$) δ 14.8, 17.9, 21.9, 21.9, 60.3, 63.0, 116.9, 123.0, 129.2, 131.8, 156.4;

IR (neat) cm^{-1} 2964w, 2361w, 1746s, 1482w, 1408m, 1392m;

mass spectrum (APCI): m/e (% relative intensity) 206.1 (30) $(M-H_2+H)^+$.
