# Supplementary material for: An efficient and practical entry to 2-amido-dienes and 3-amido-trienes from allenamides through stereoselective 1,3-hydrogen shifts
Source: Beilstein J Org Chem. 2011 Apr 7;7:410–20. doi: 10.3762/bjoc.7.53 (PMC3079123; doi:10.3762/bjoc.7.53)

Supporting Information

for

An efficient and practical entry to 2-amido-dienes and 3-amido-trienes from allenamides through stereoselective 1,3-hydrogen shifts.

Ryuji Hayashi, John B. Feltenberger, Andrew G. Lohse, Mary C. Walton and Richard P. Hsung*

Address: Department of Chemistry and Division of Pharmaceutical Sciences, University of Wisconsin, Madison, WI 53705

Email: Richard P. Hsung* - rhsung@wisc.edu

*Corresponding author

Proton and carbon NMR spectra, and NOE data.

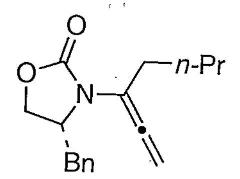

5a

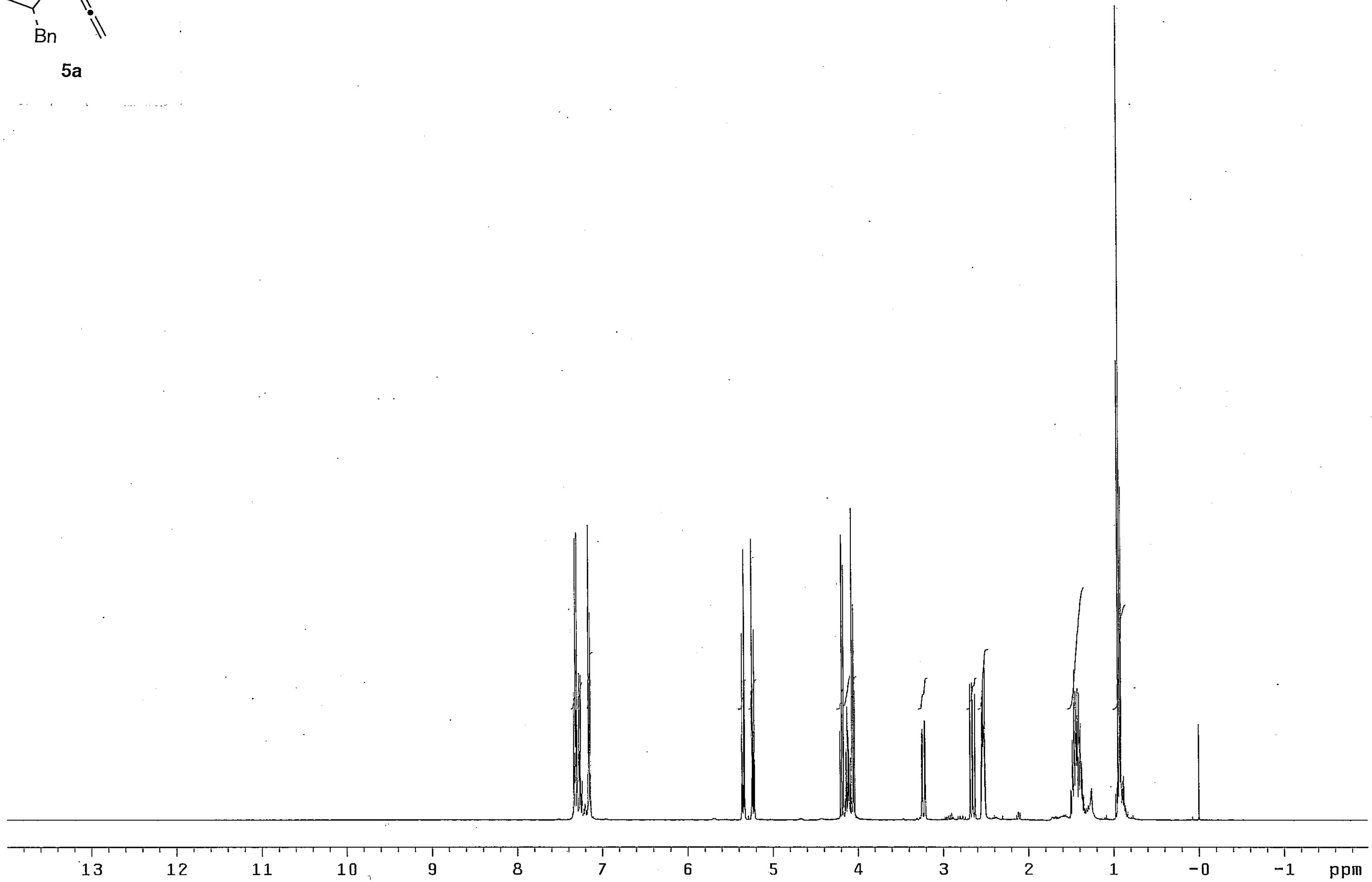

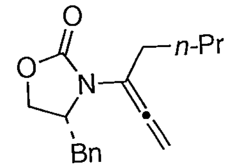

5a

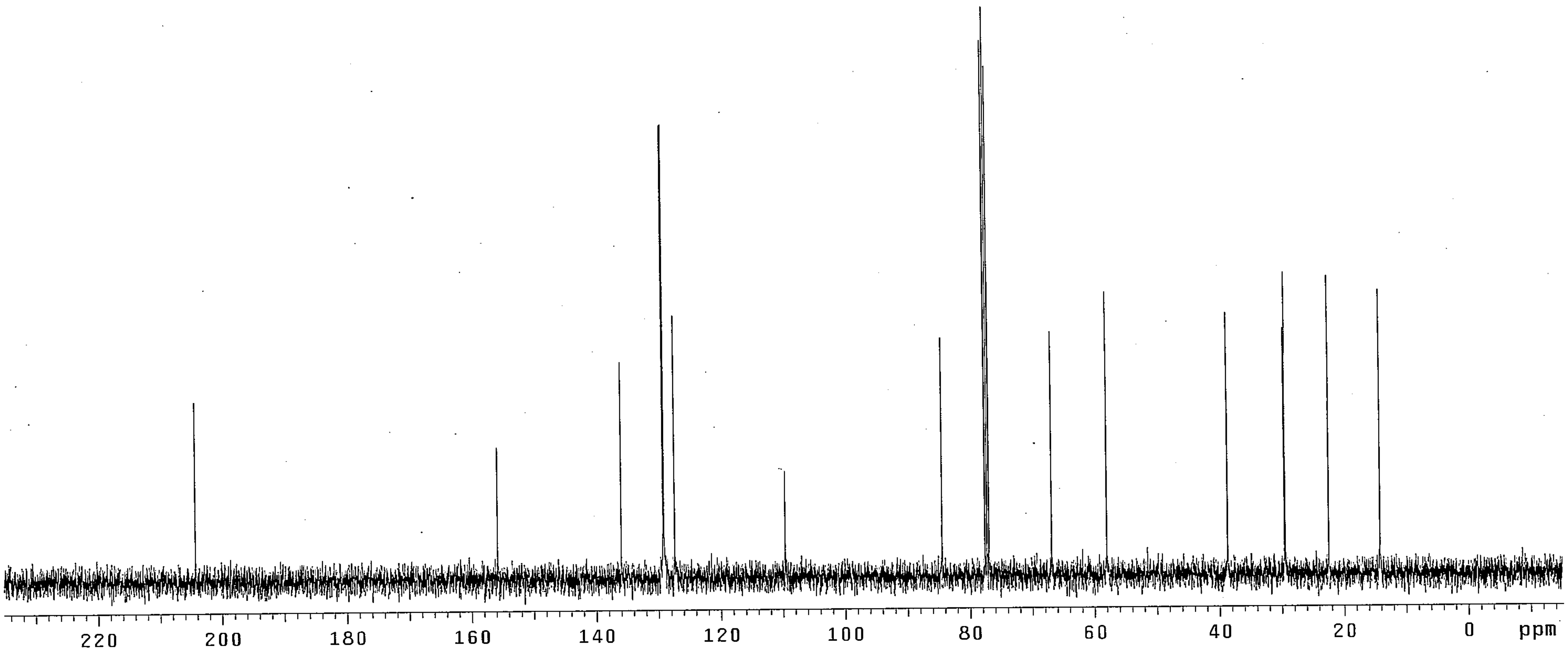

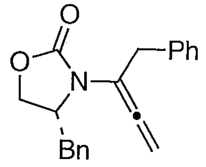

5b

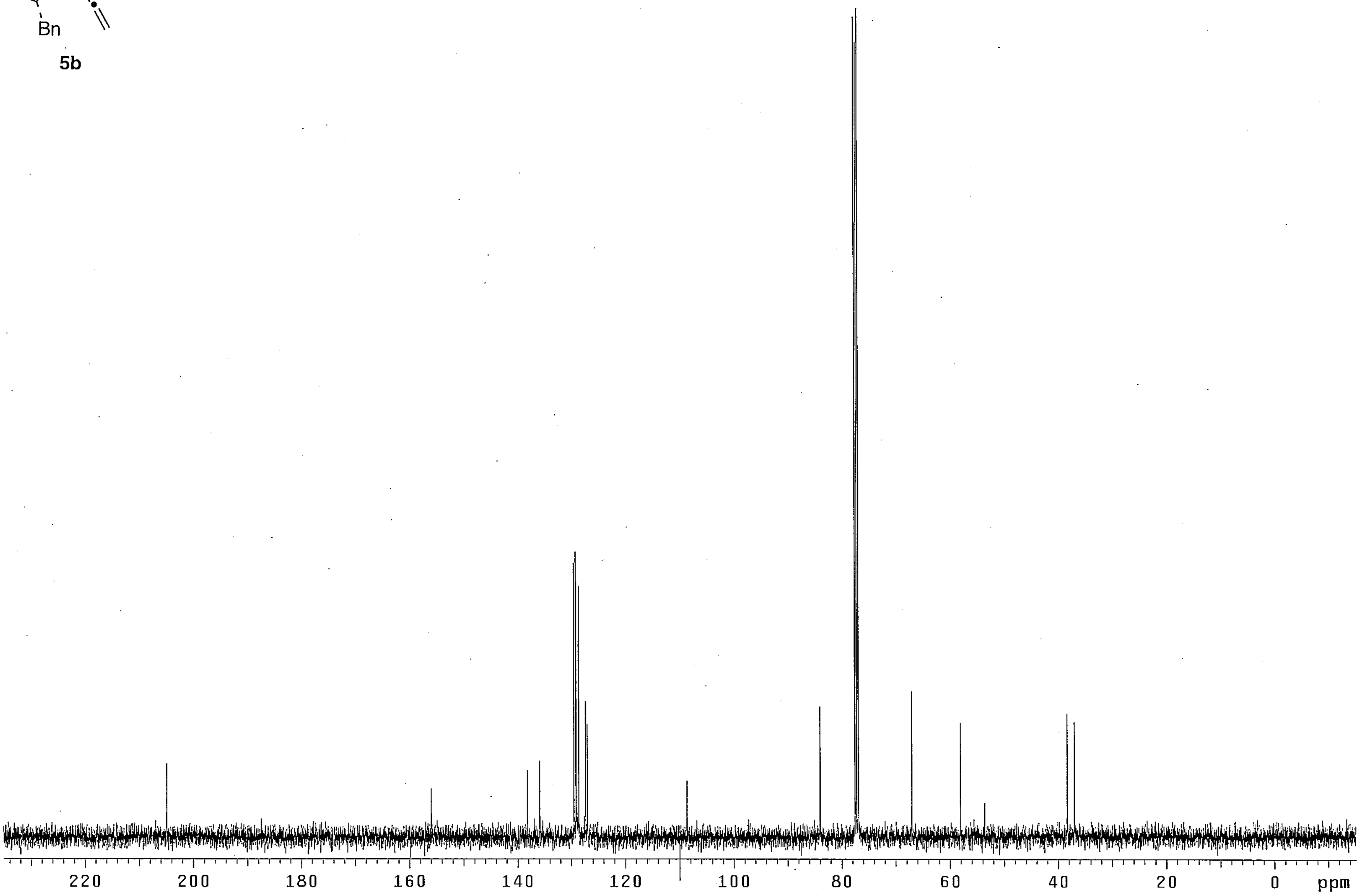

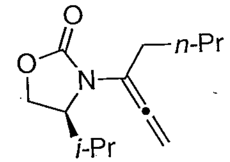

7a

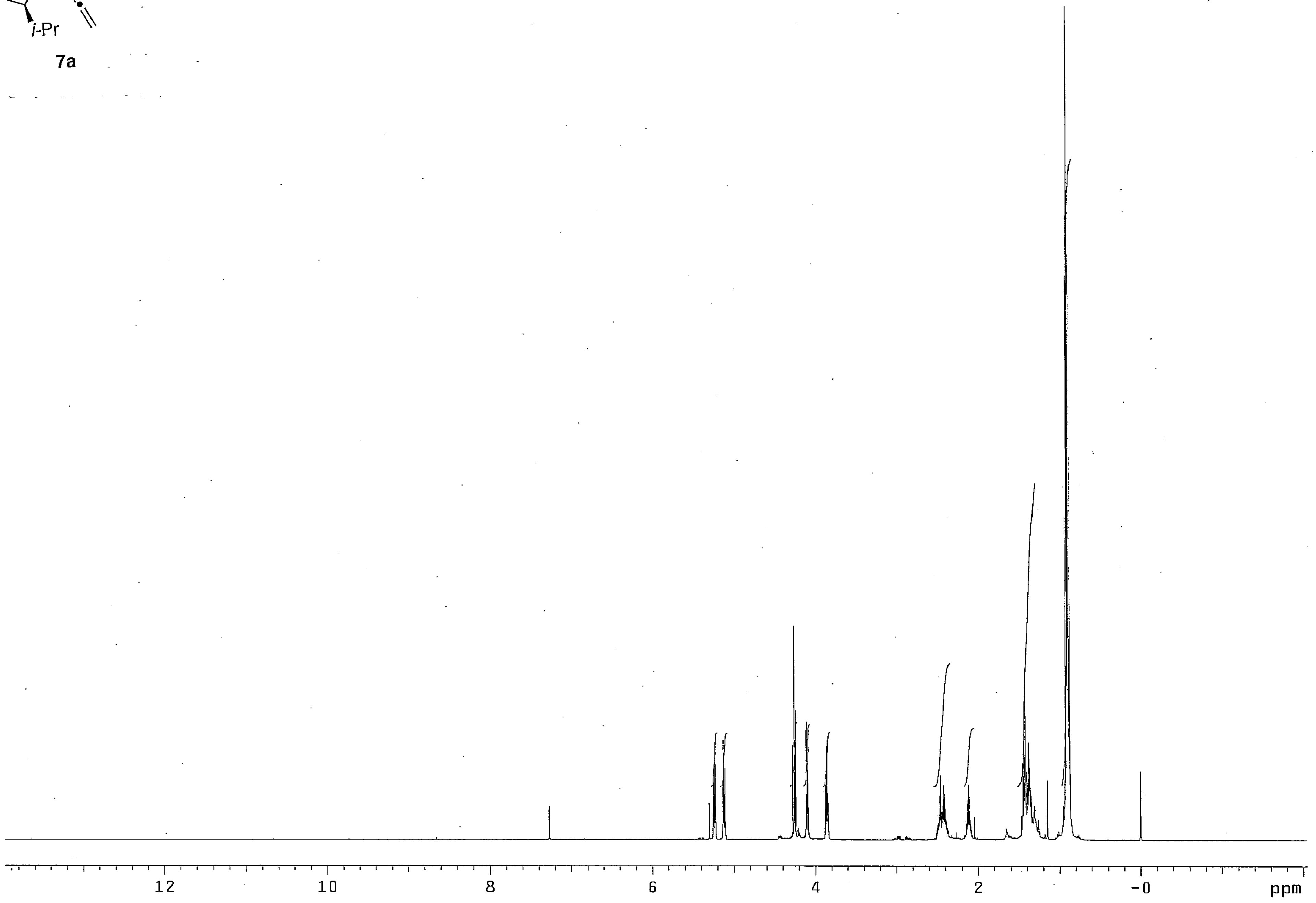

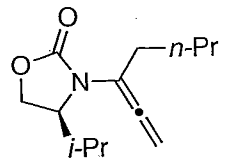

7a

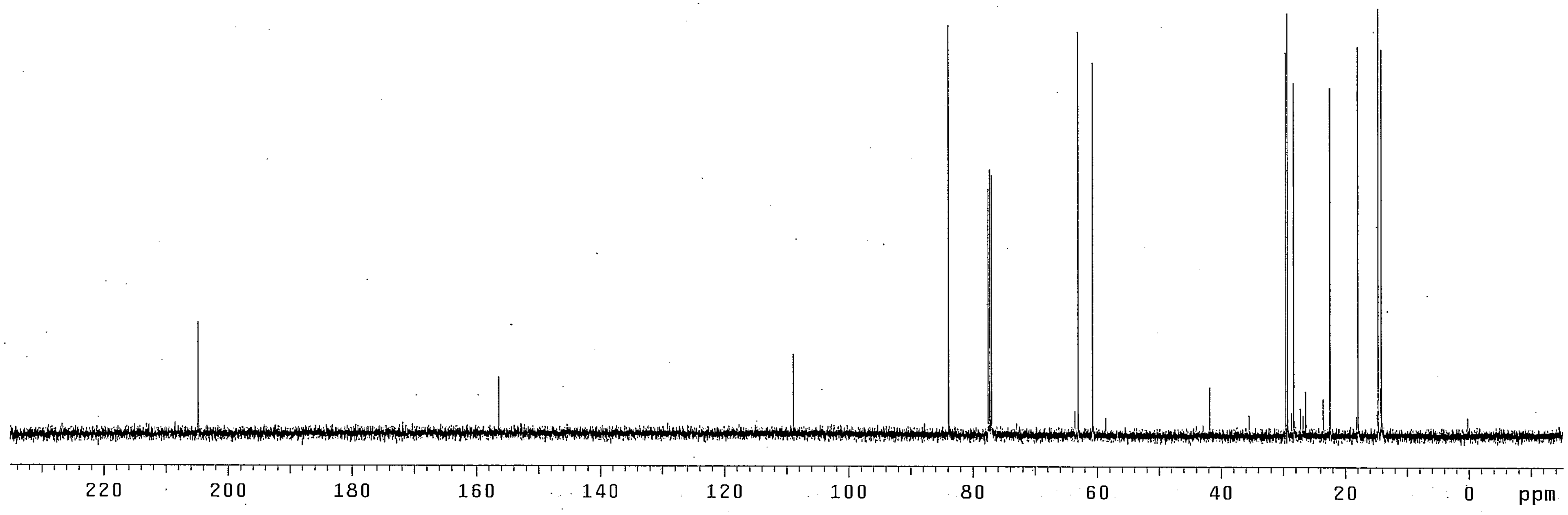

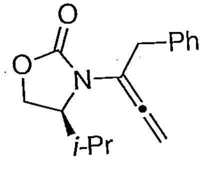

7b

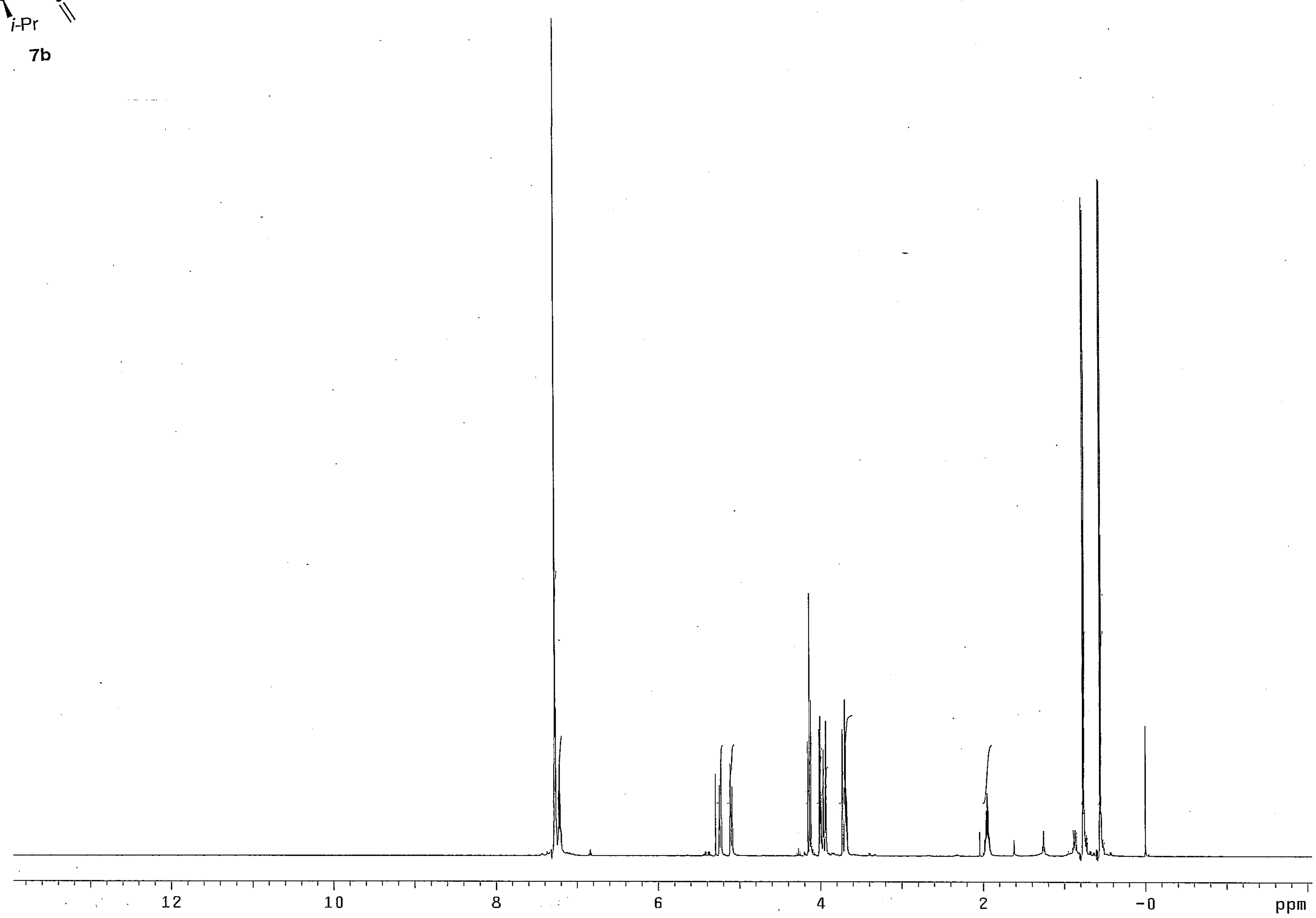

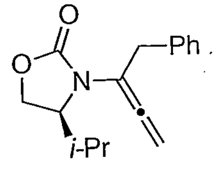

7b

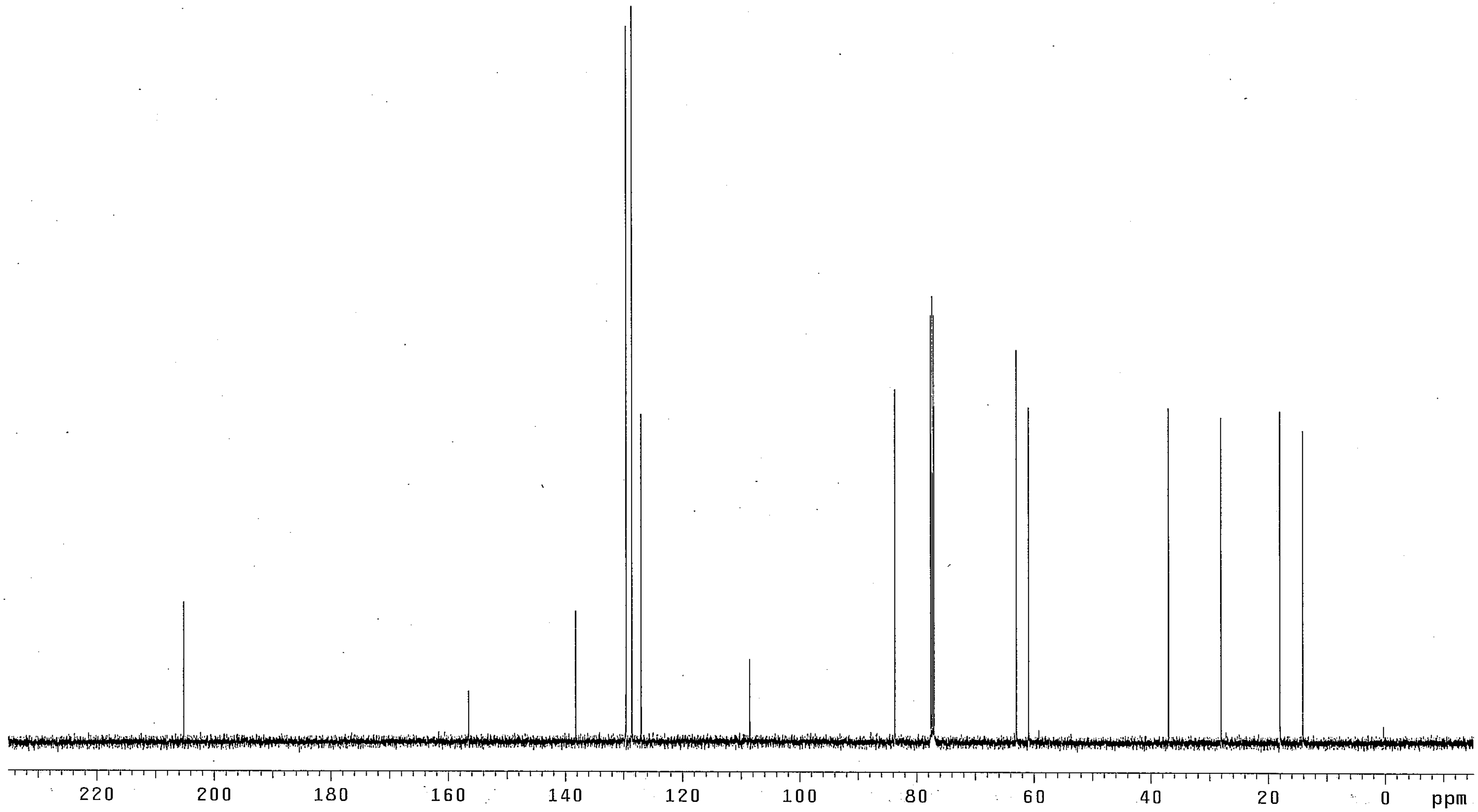

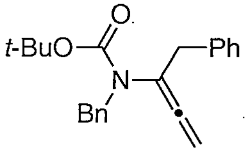

11

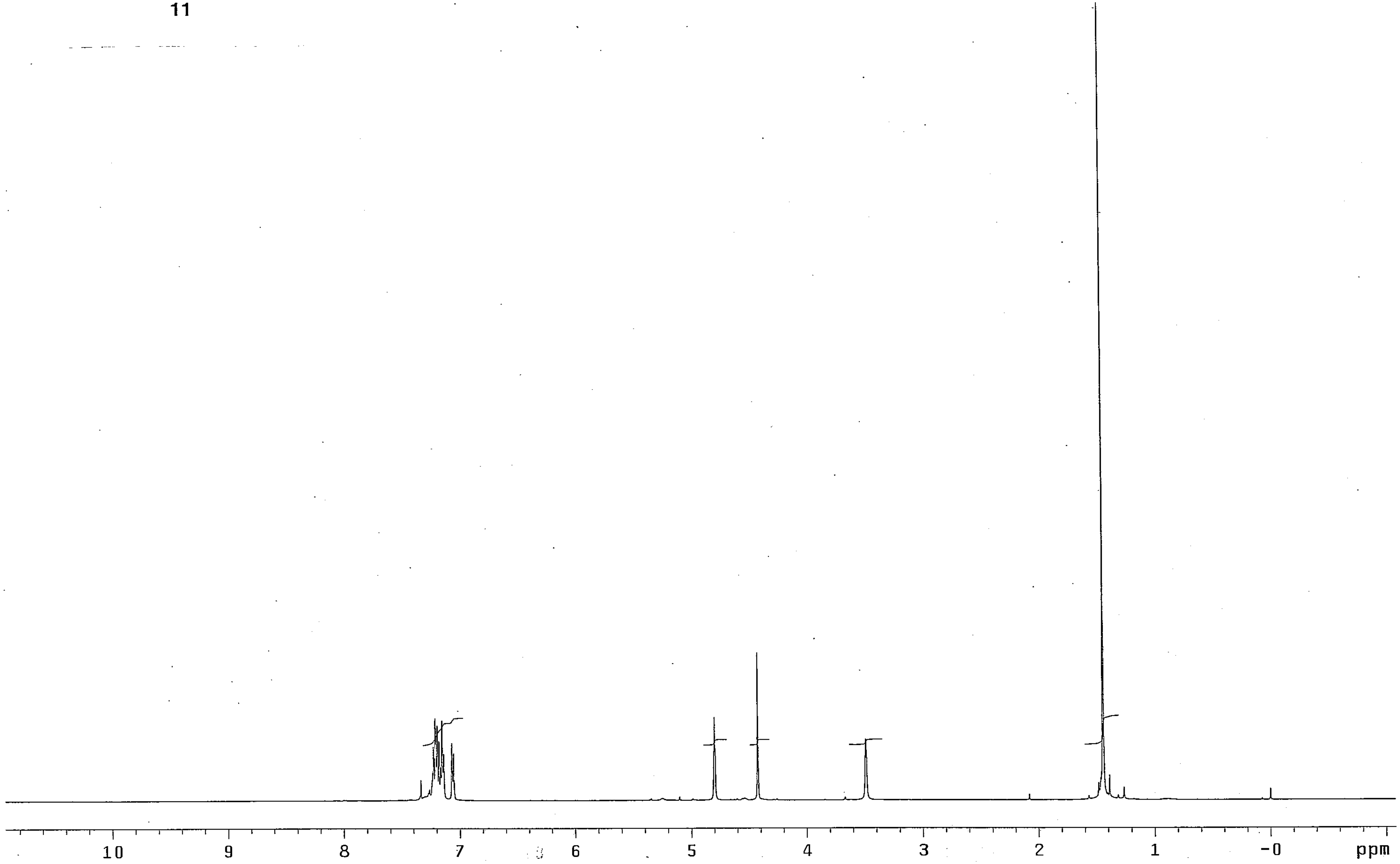

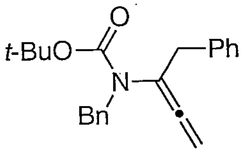

11

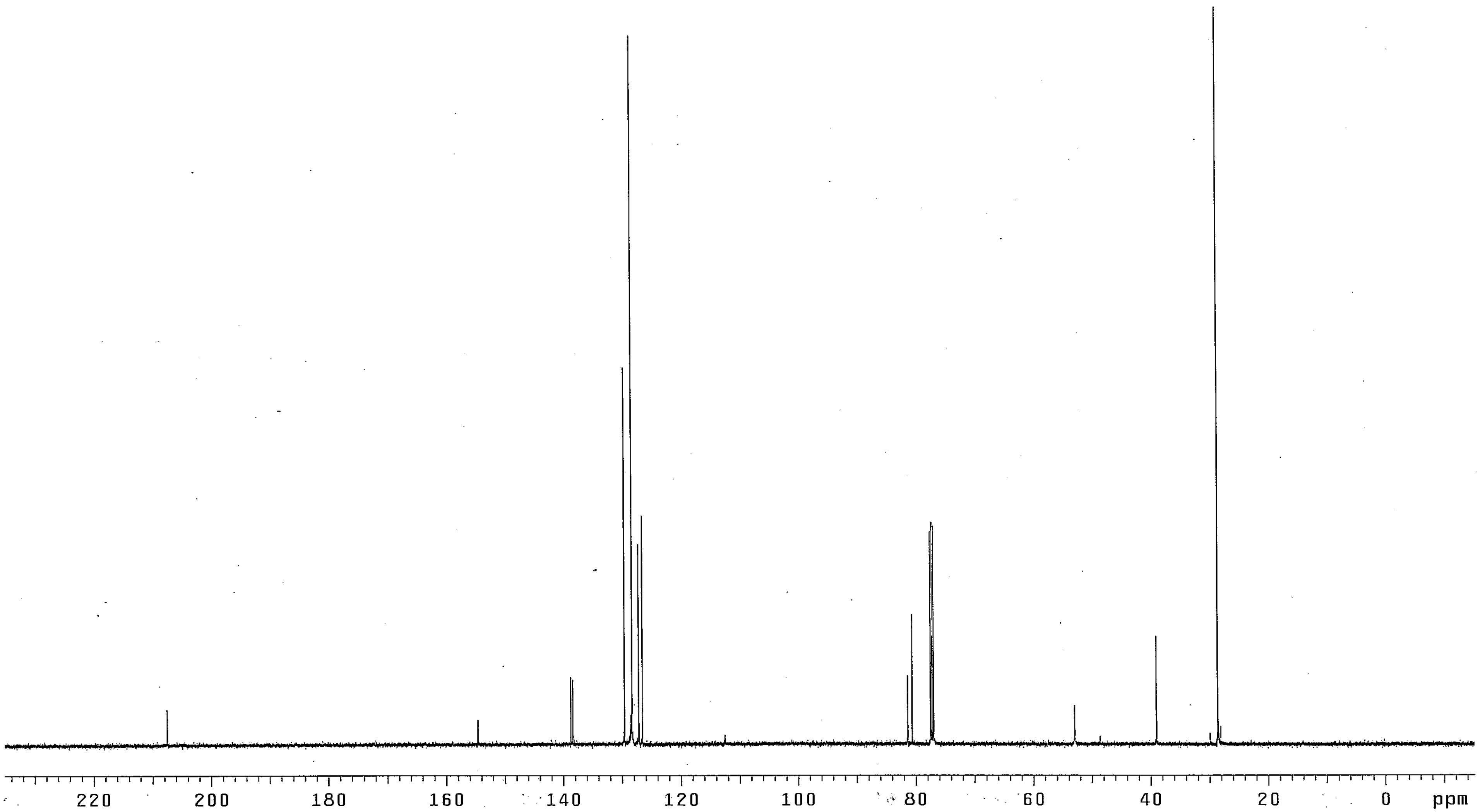

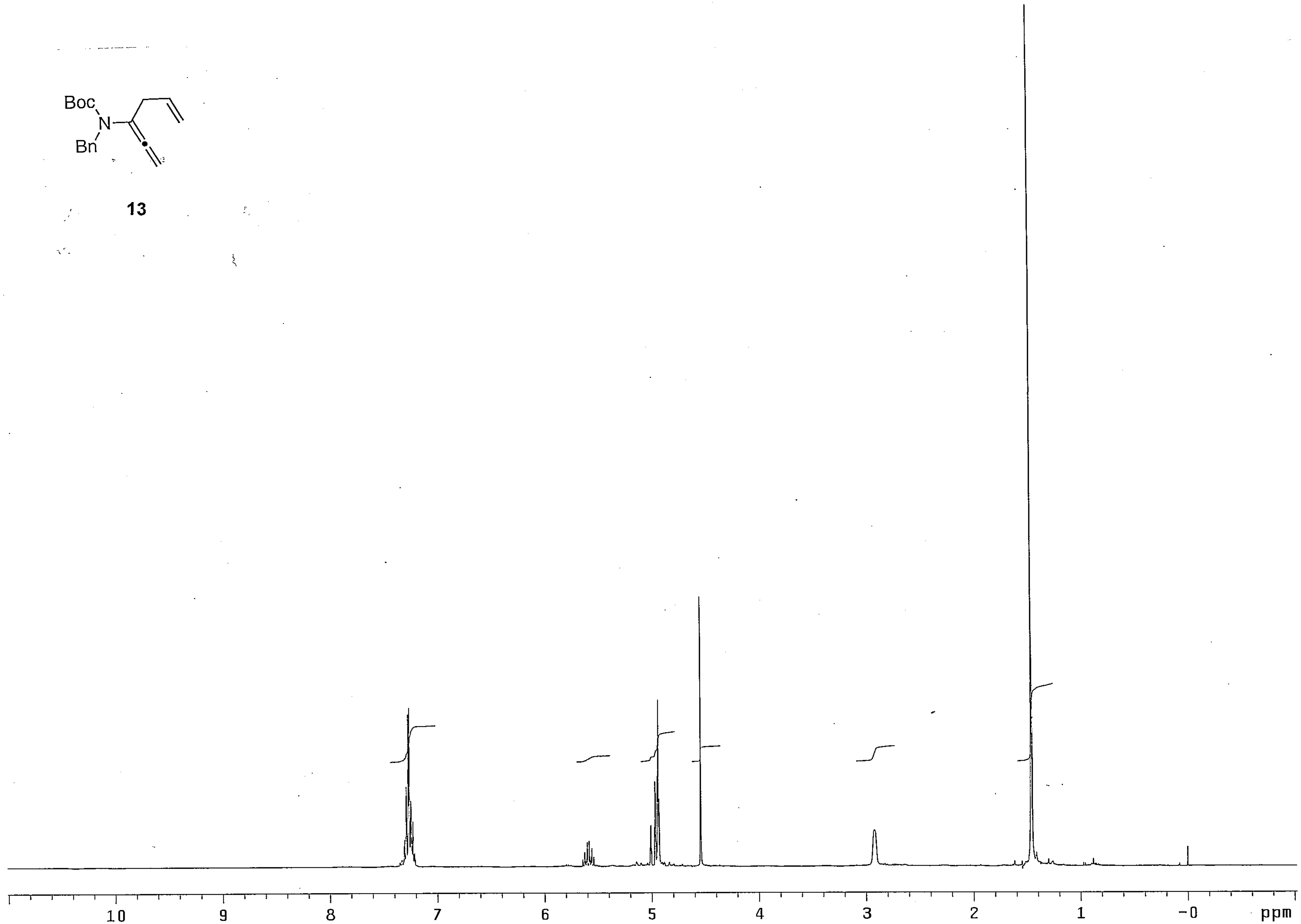

Boc
N
Bn
13
10
9
8
7
6
5
4
3
2
1
-0
ppm

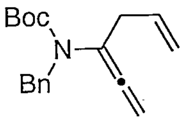

13

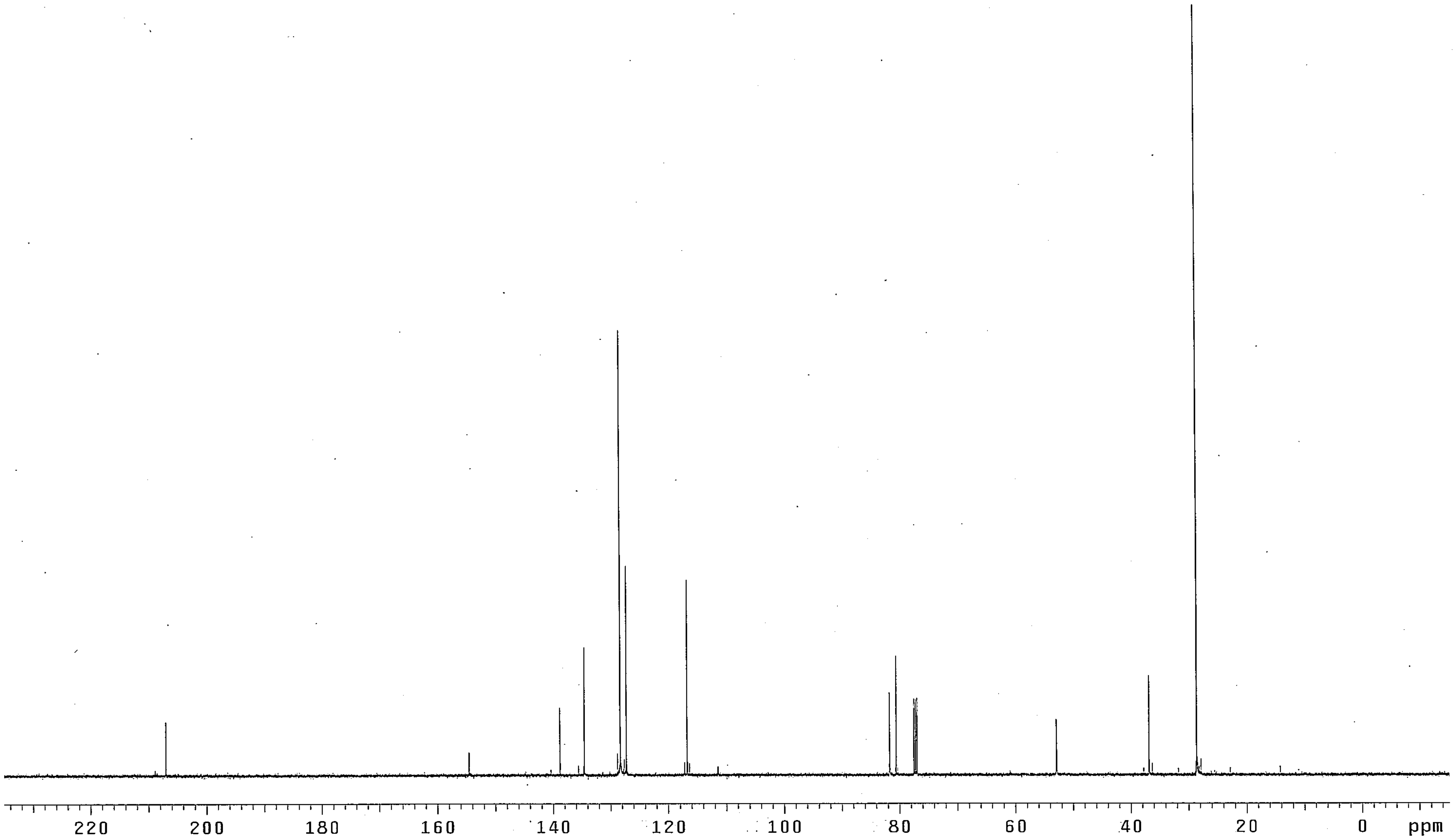

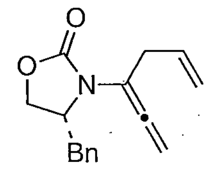

15a

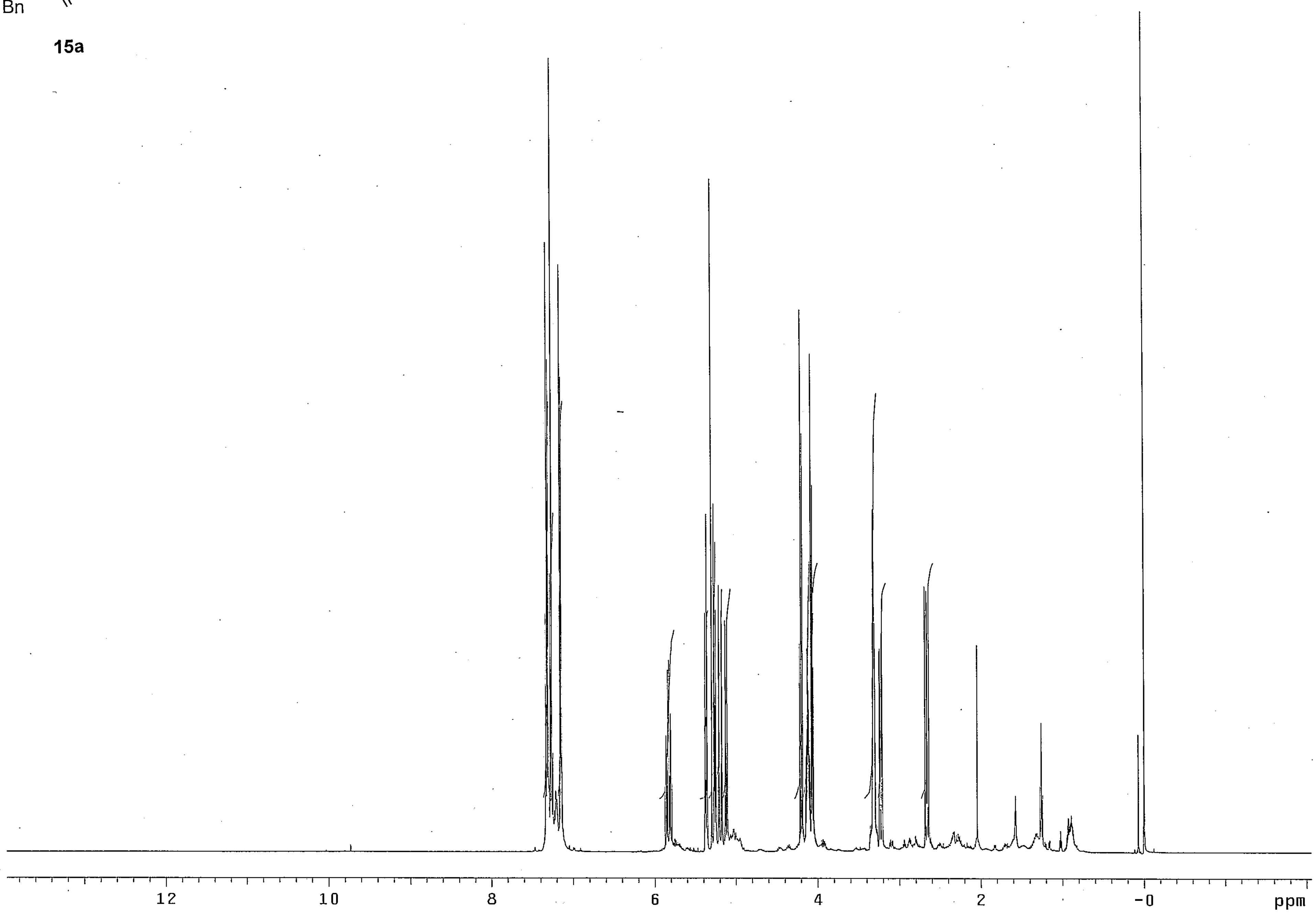

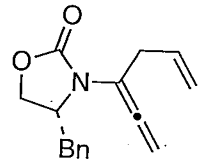

15a

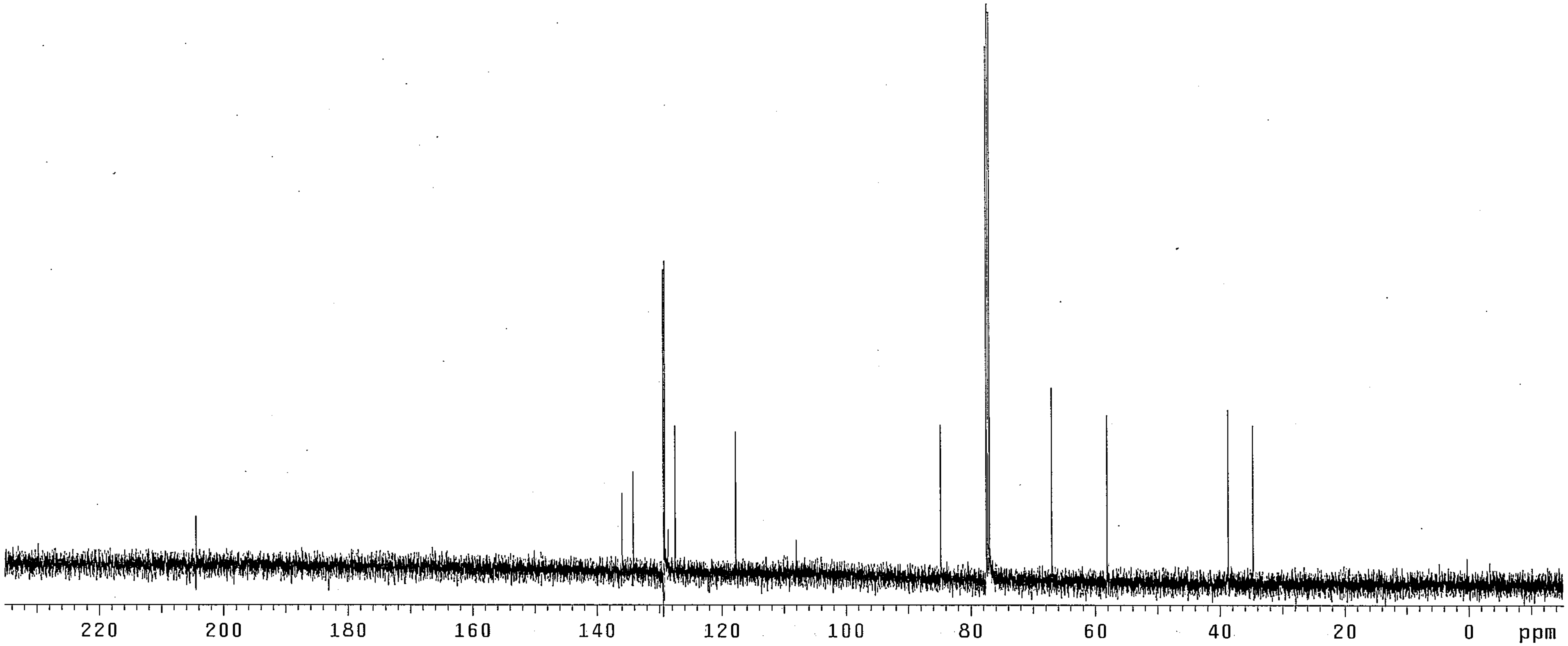

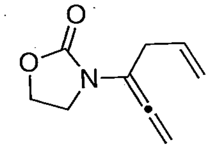

15b

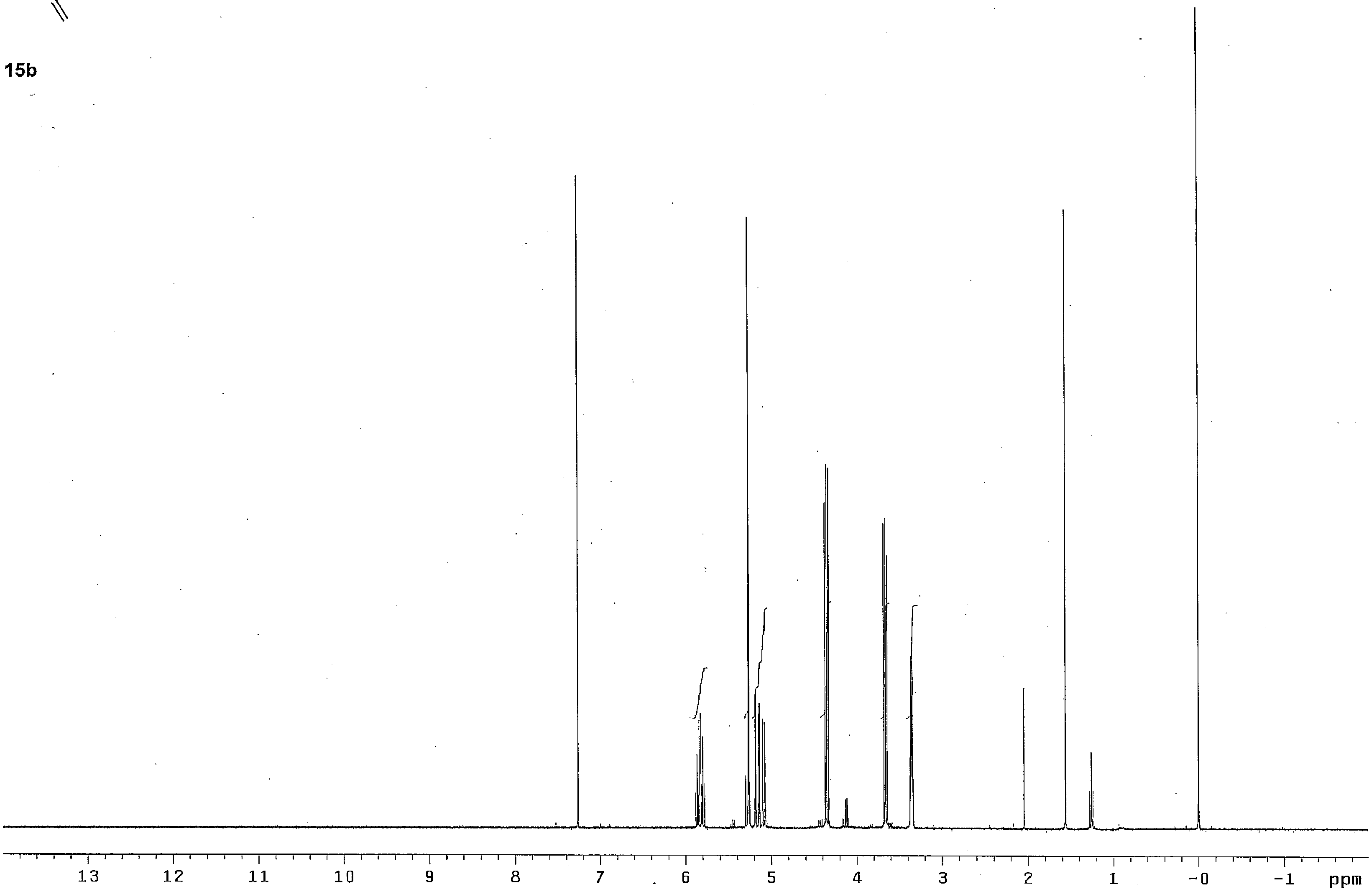

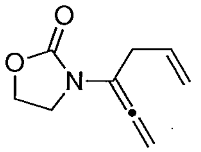

15b

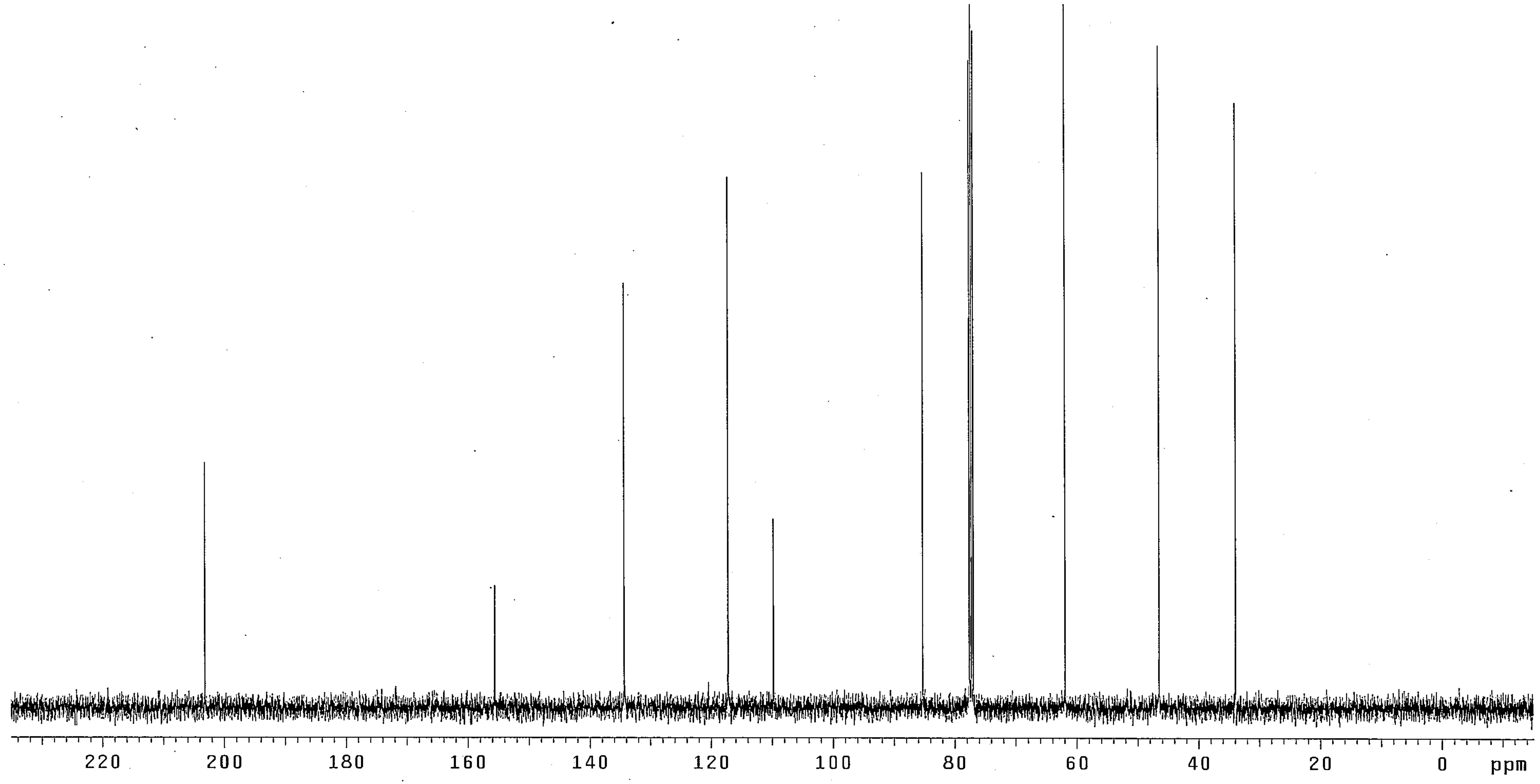

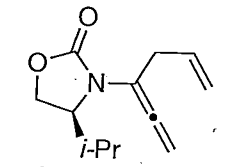

15c

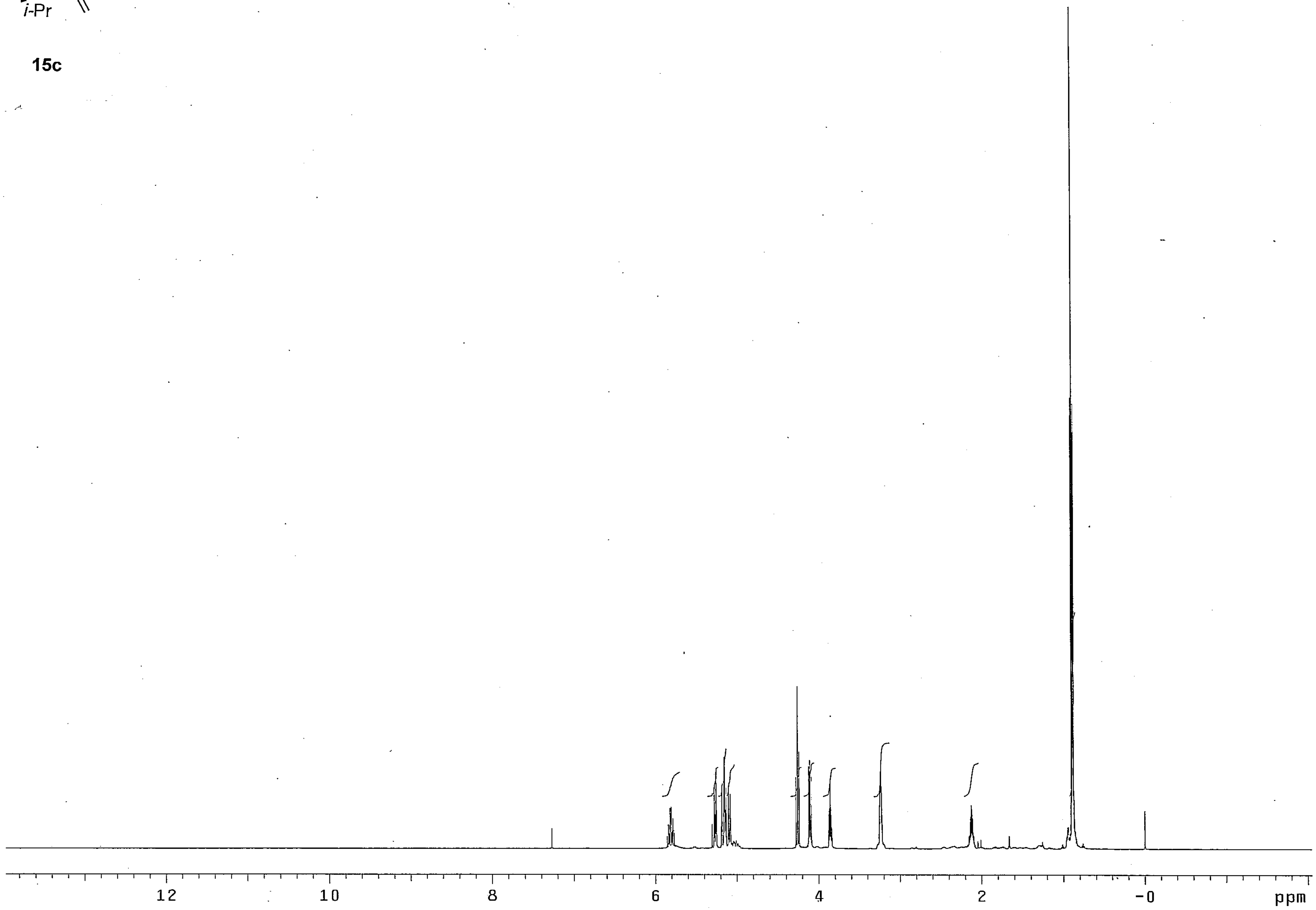

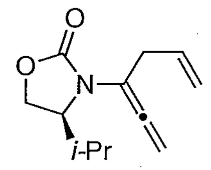

15c

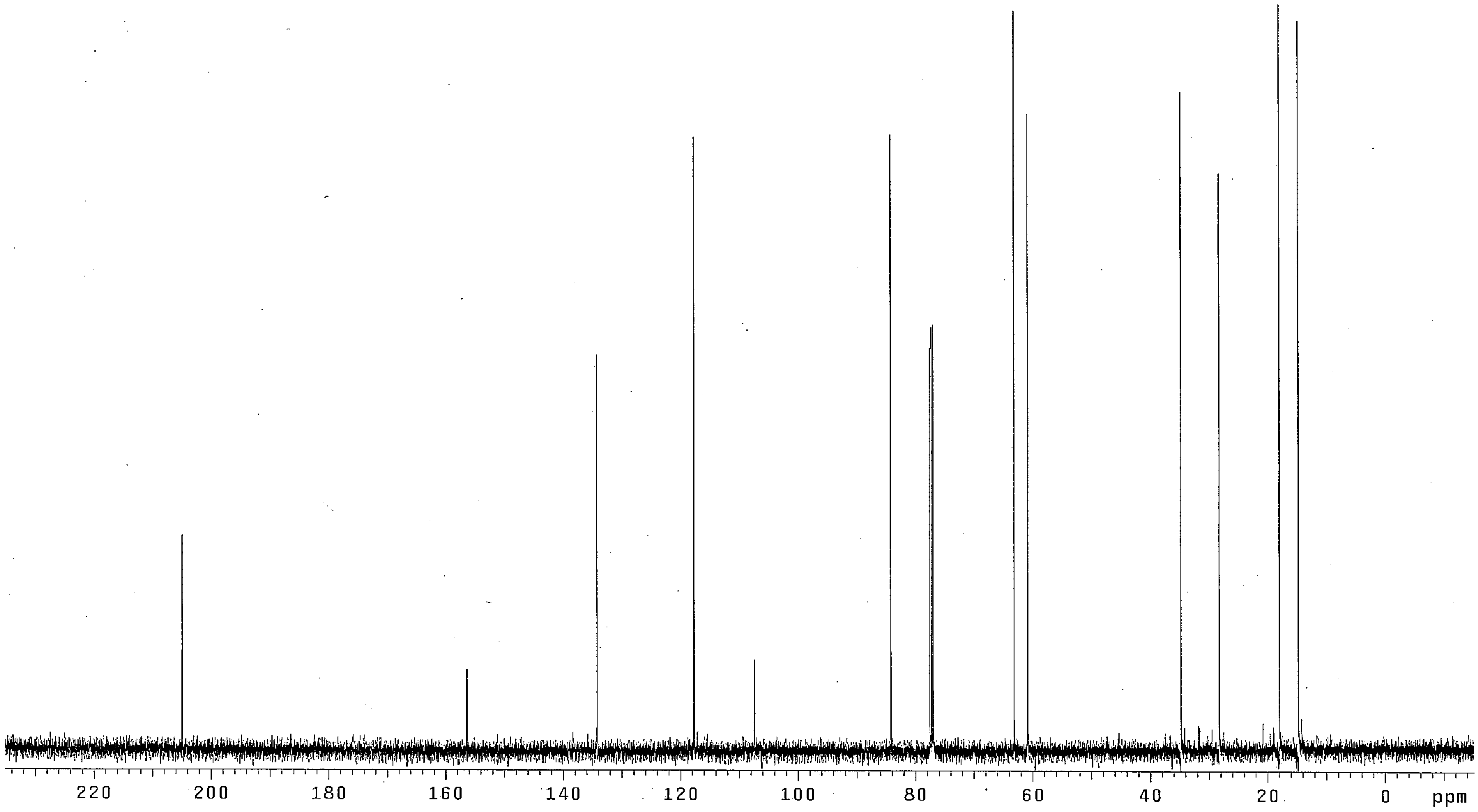

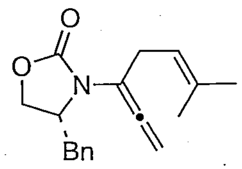

16a

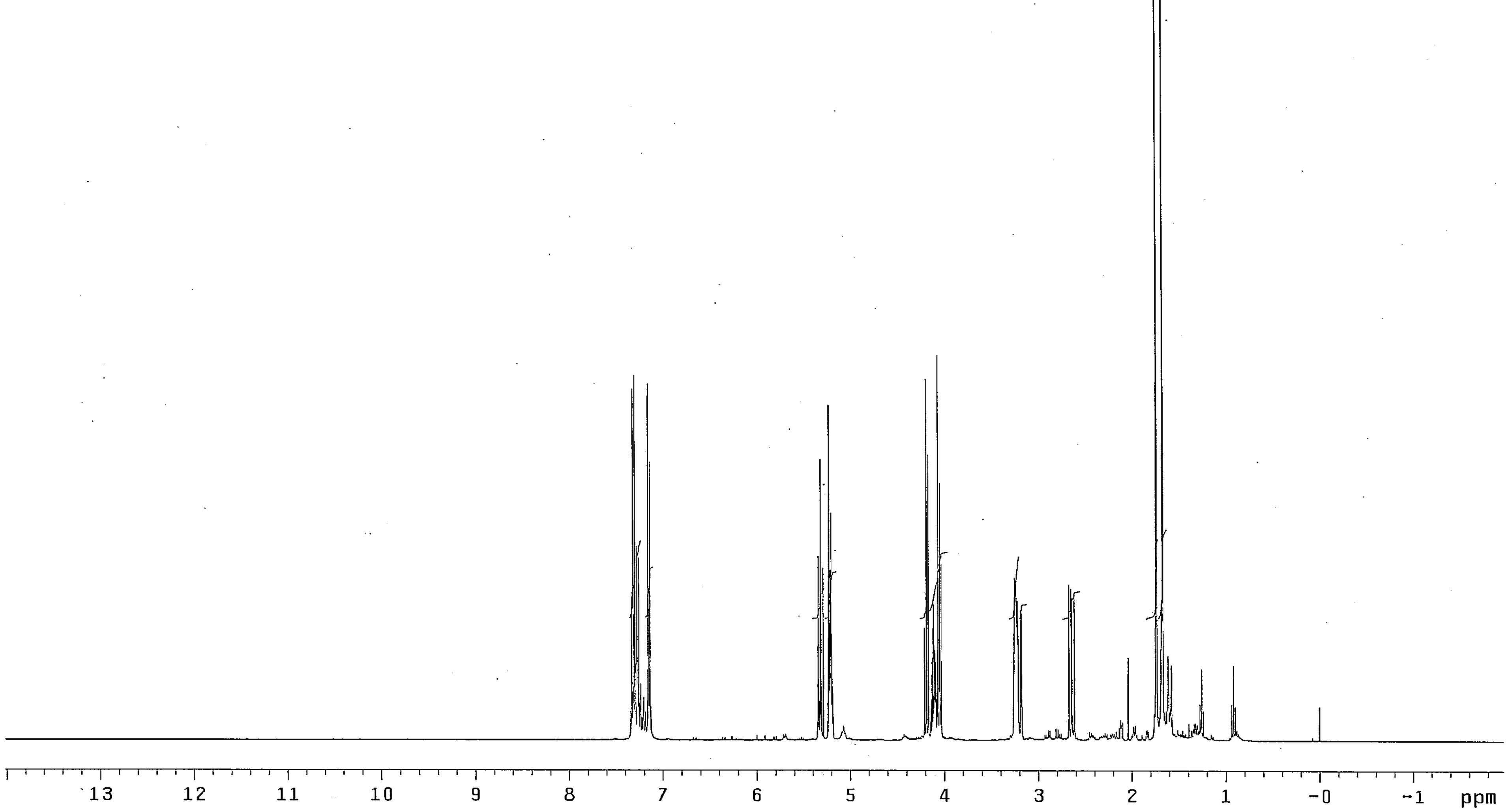

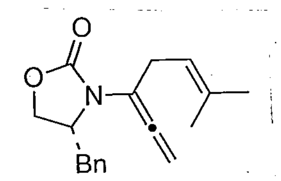

16a

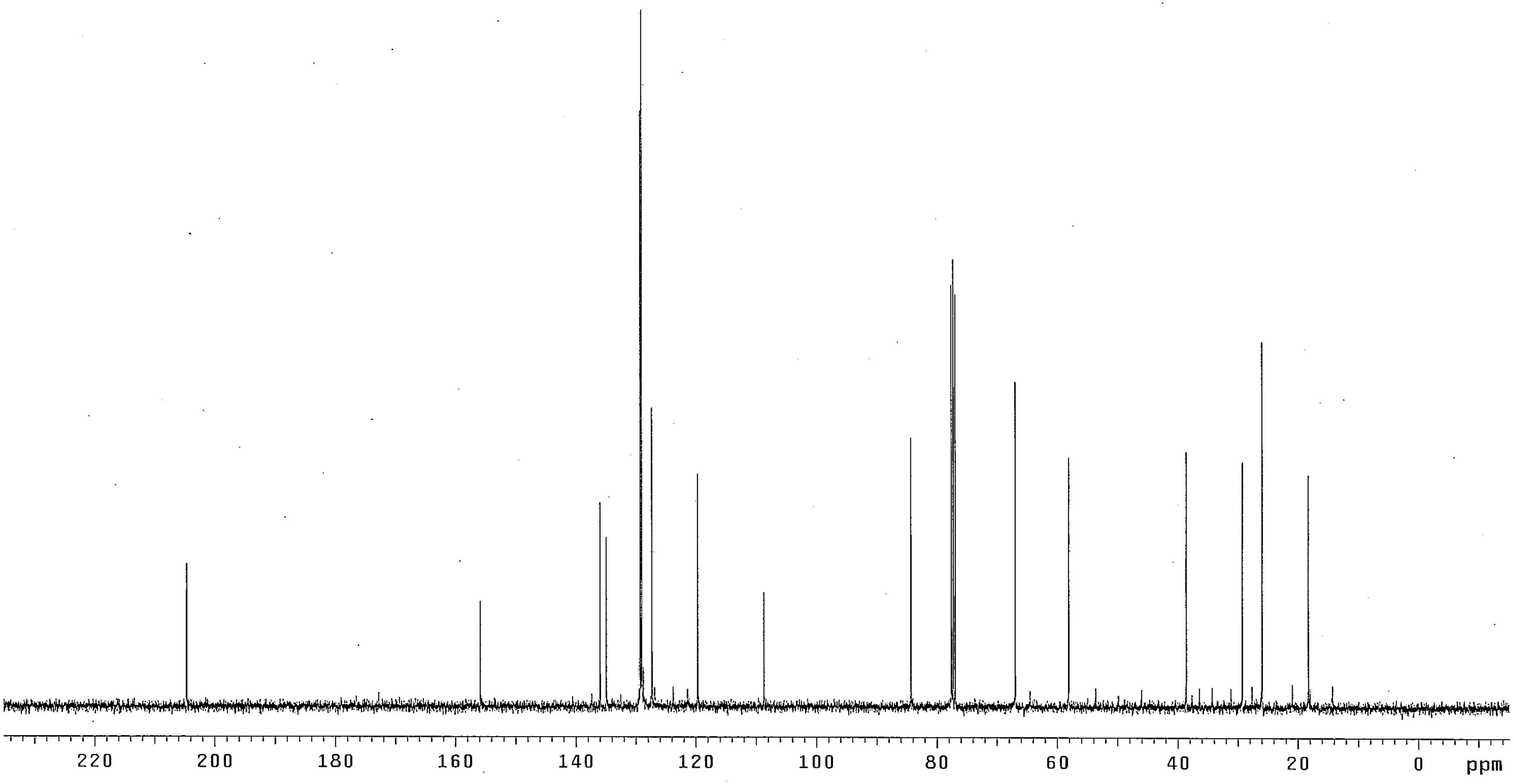

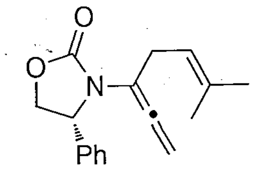

16b

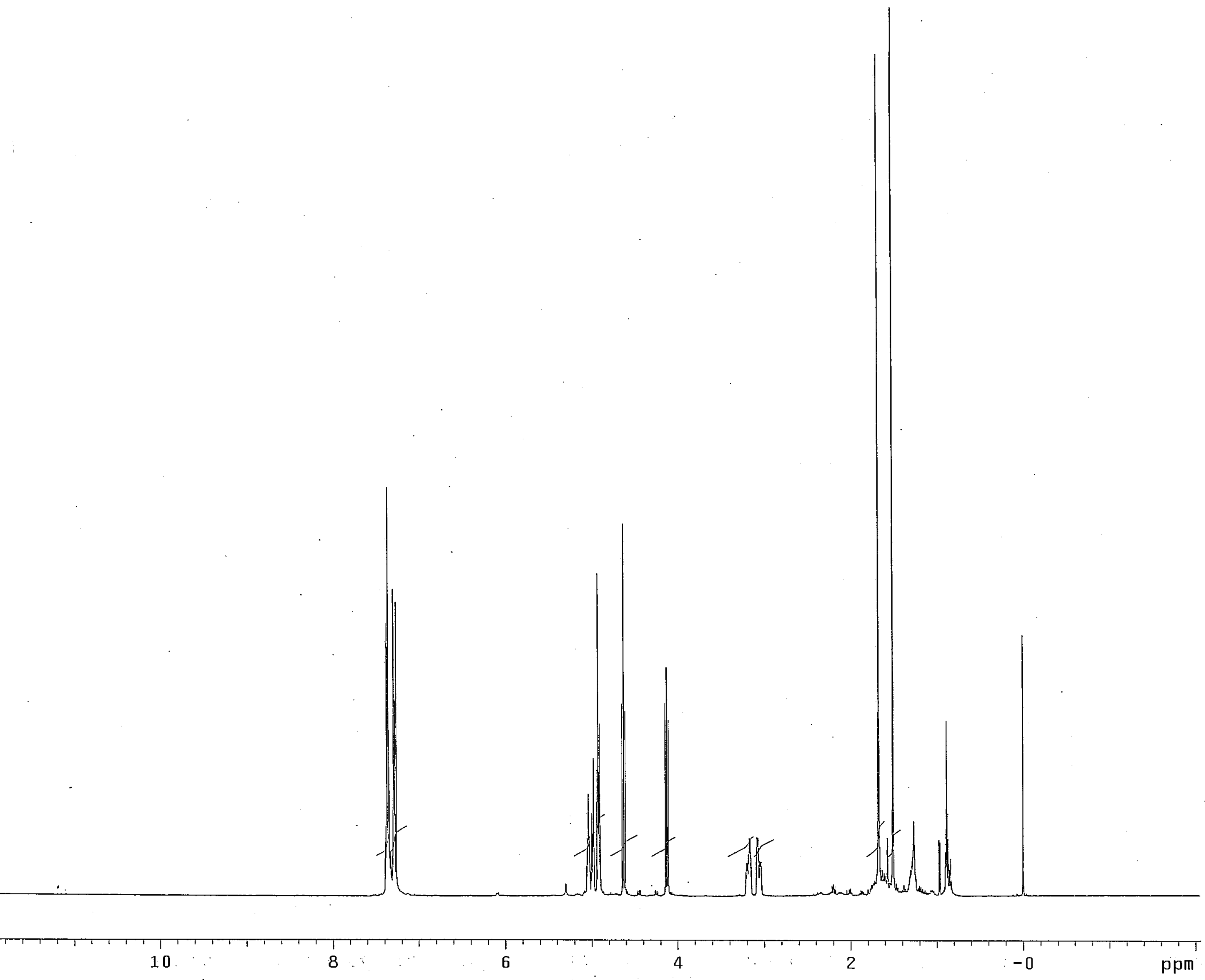

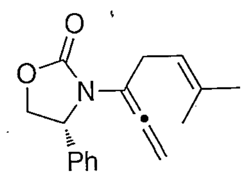

16b

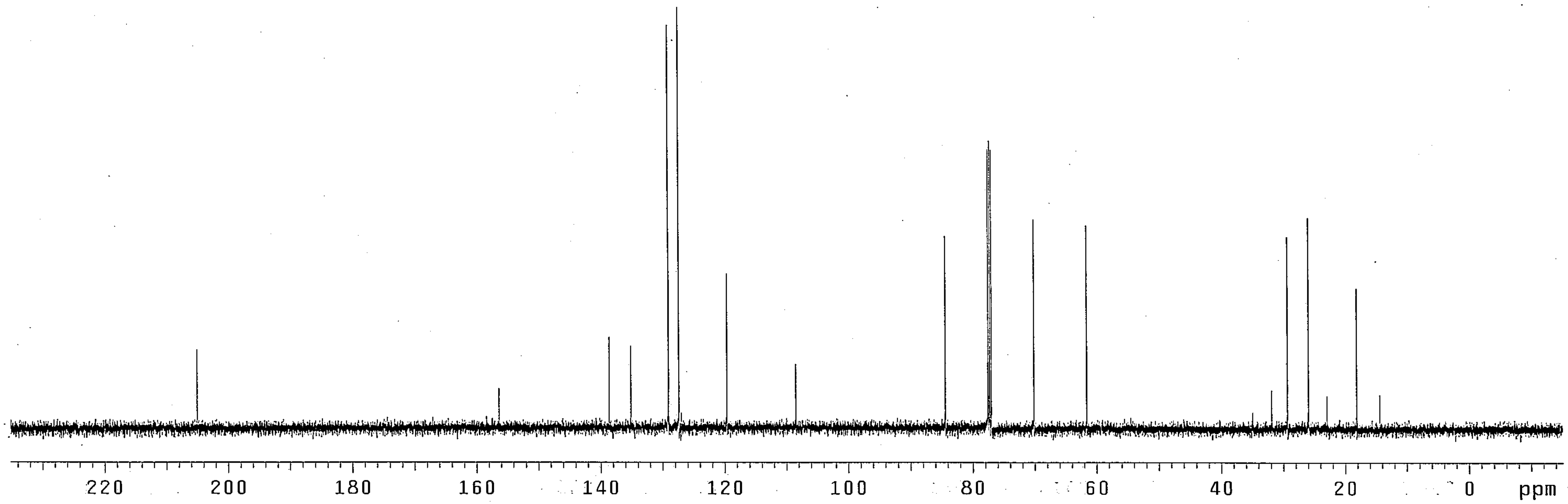

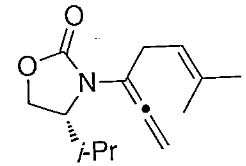

16c

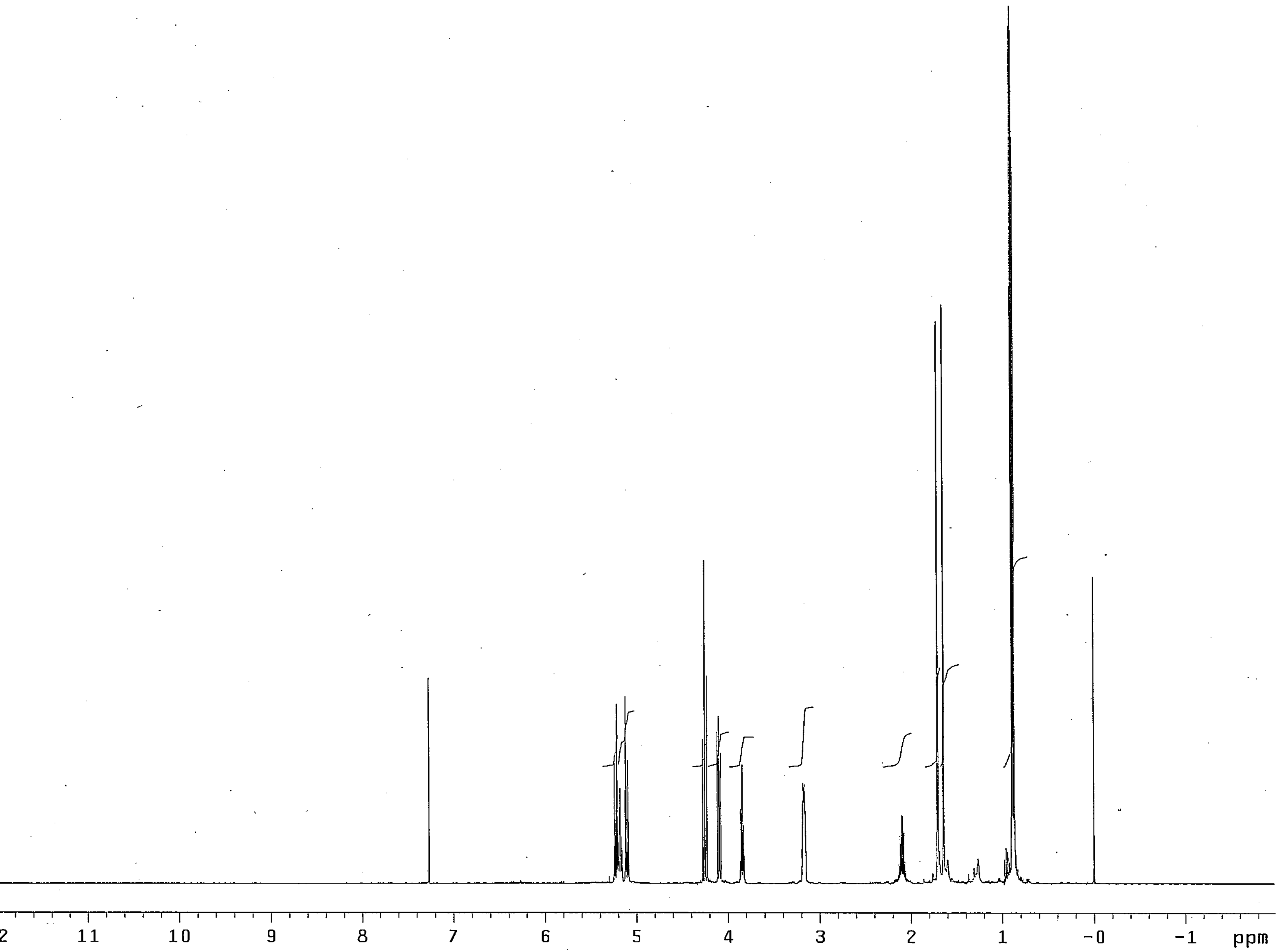

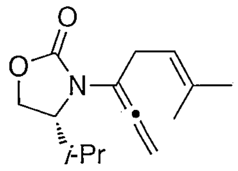

16c

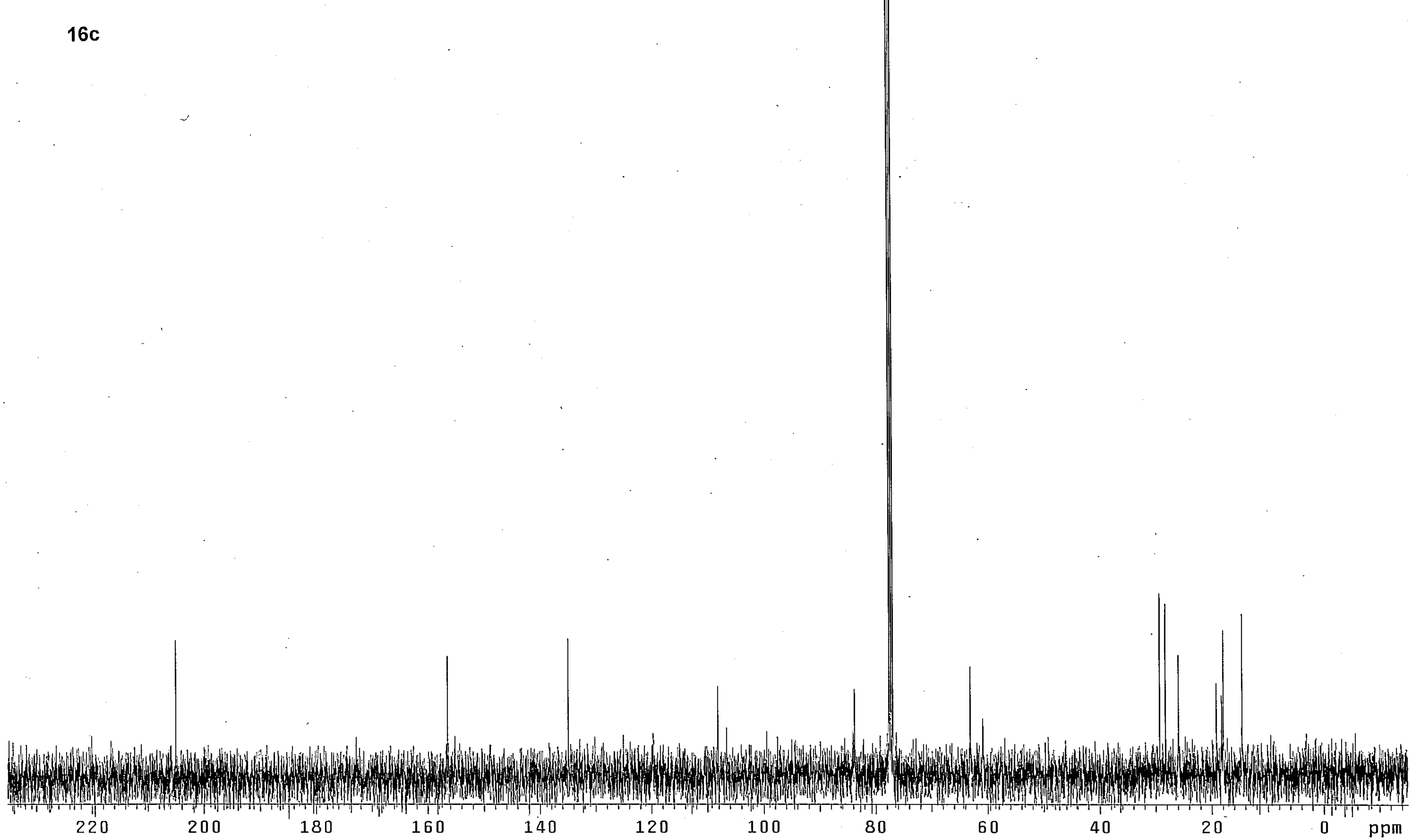

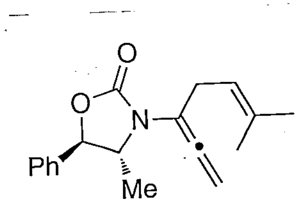

16d

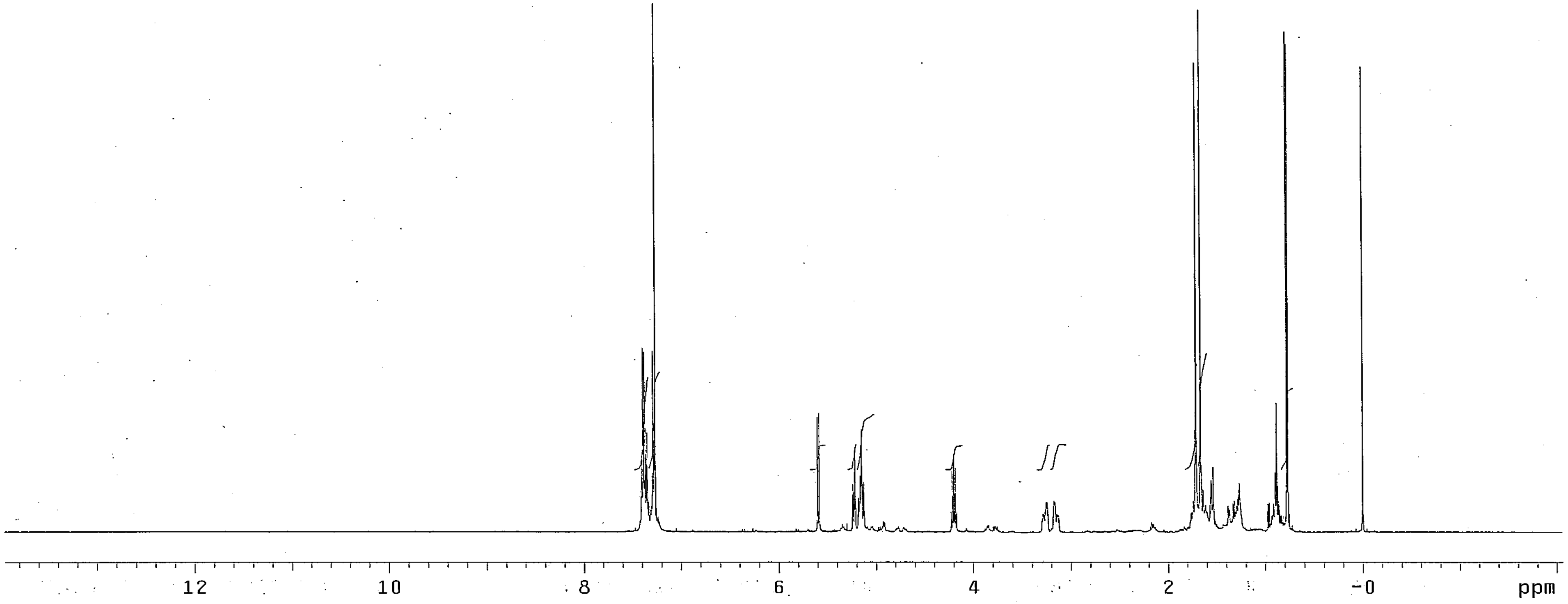

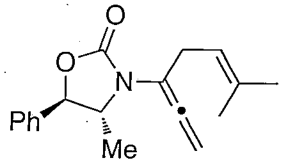

16d

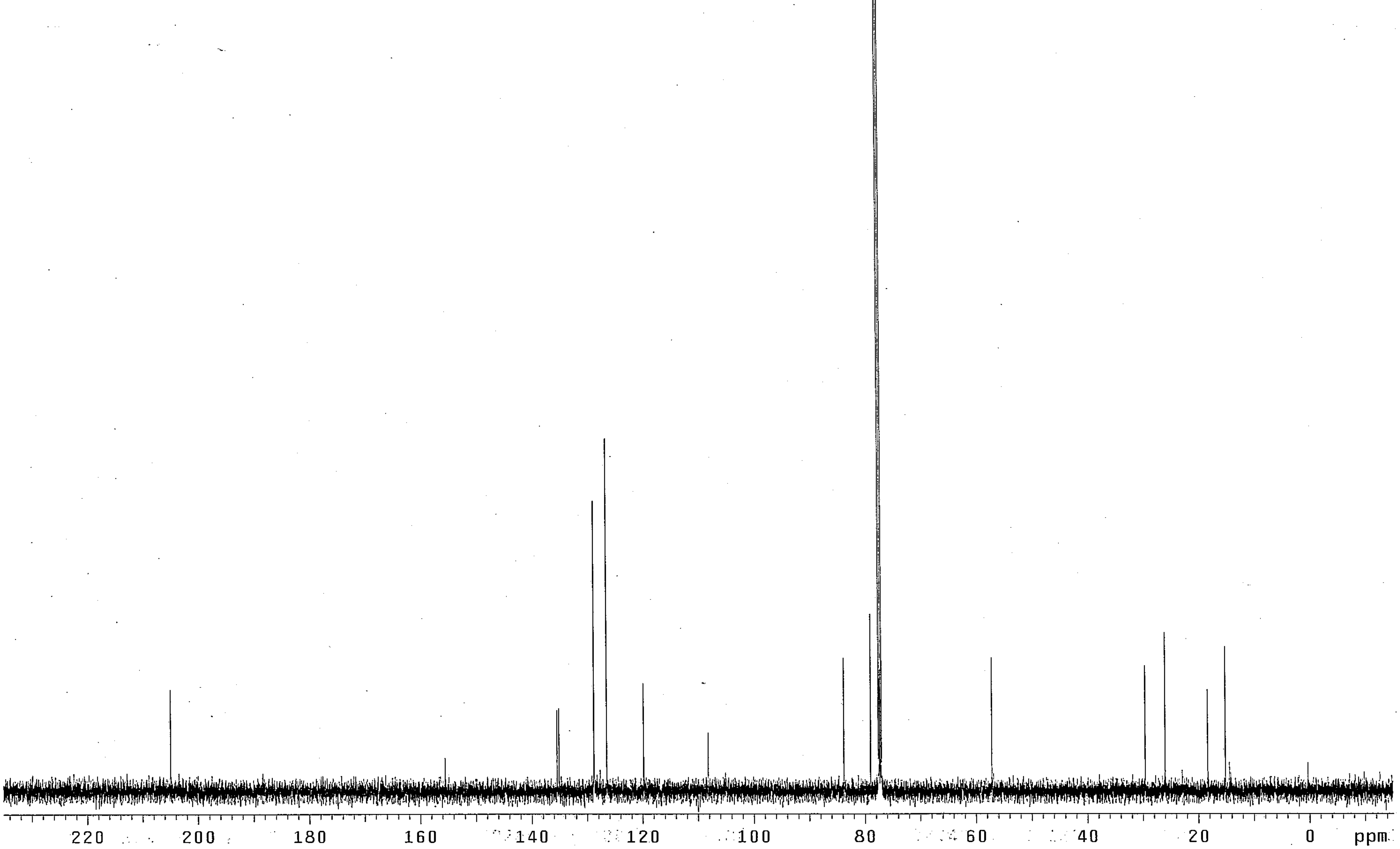

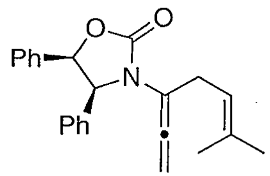

16e

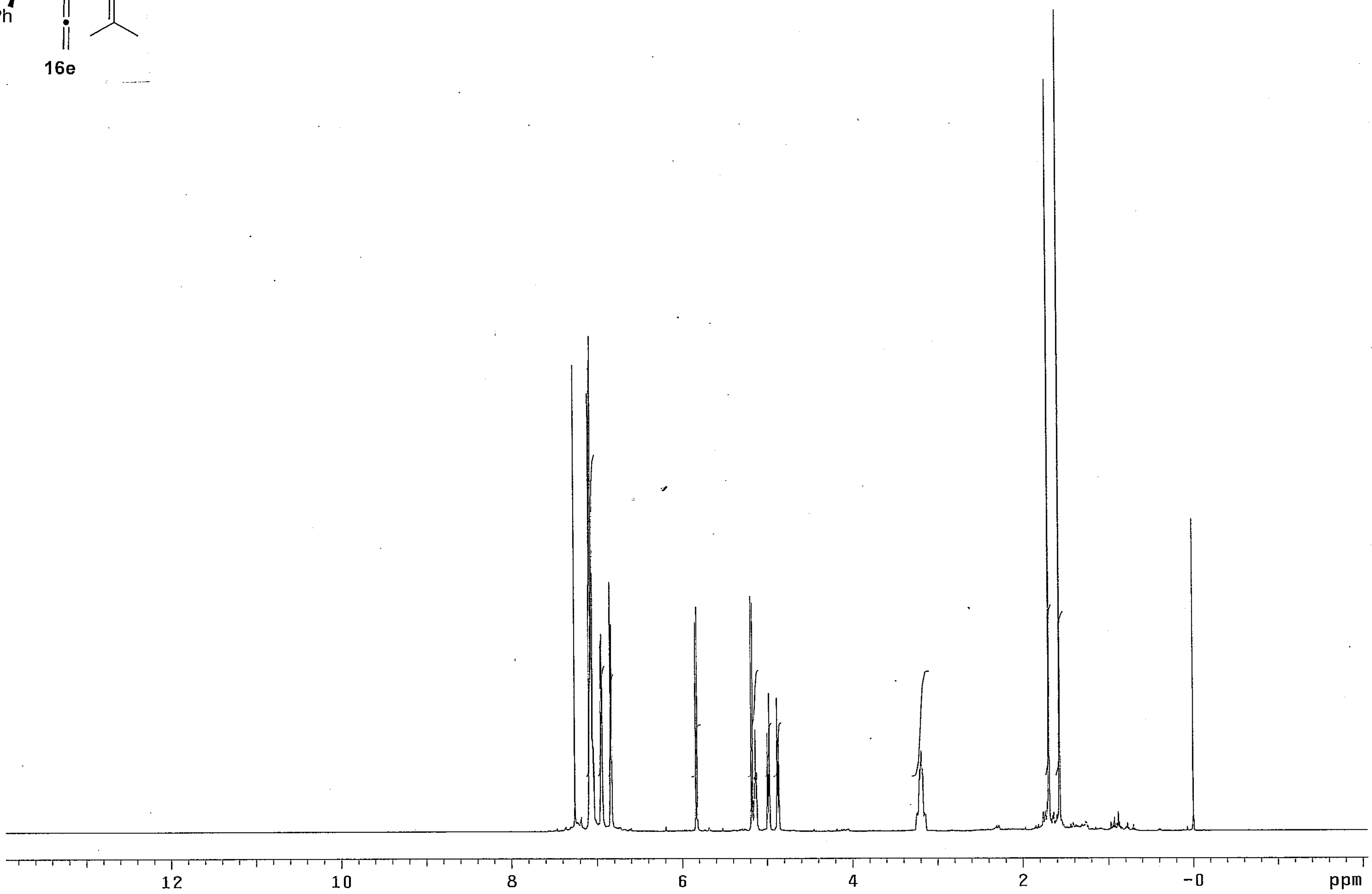

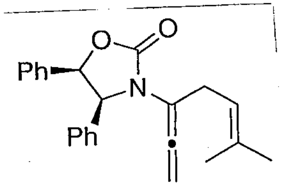

16e

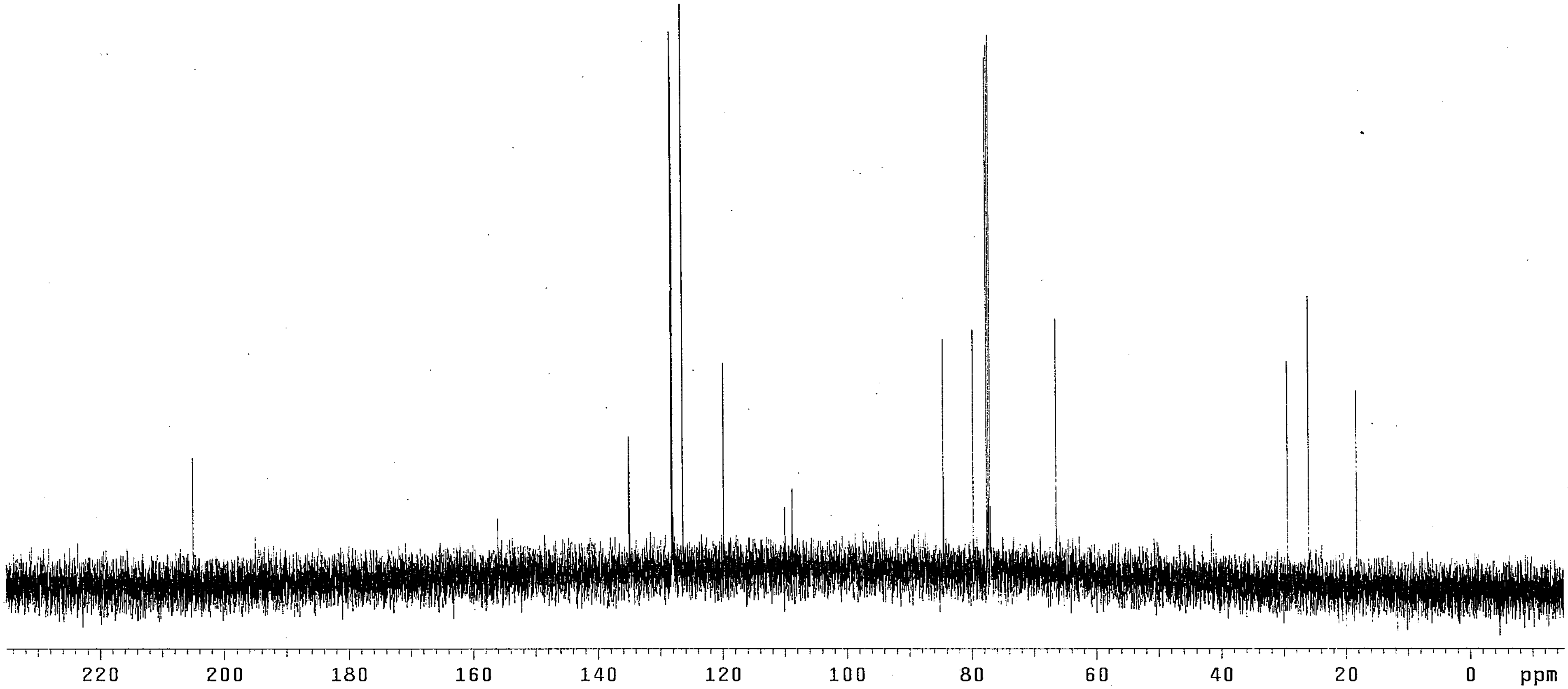

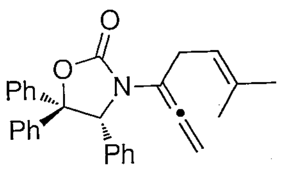

16f

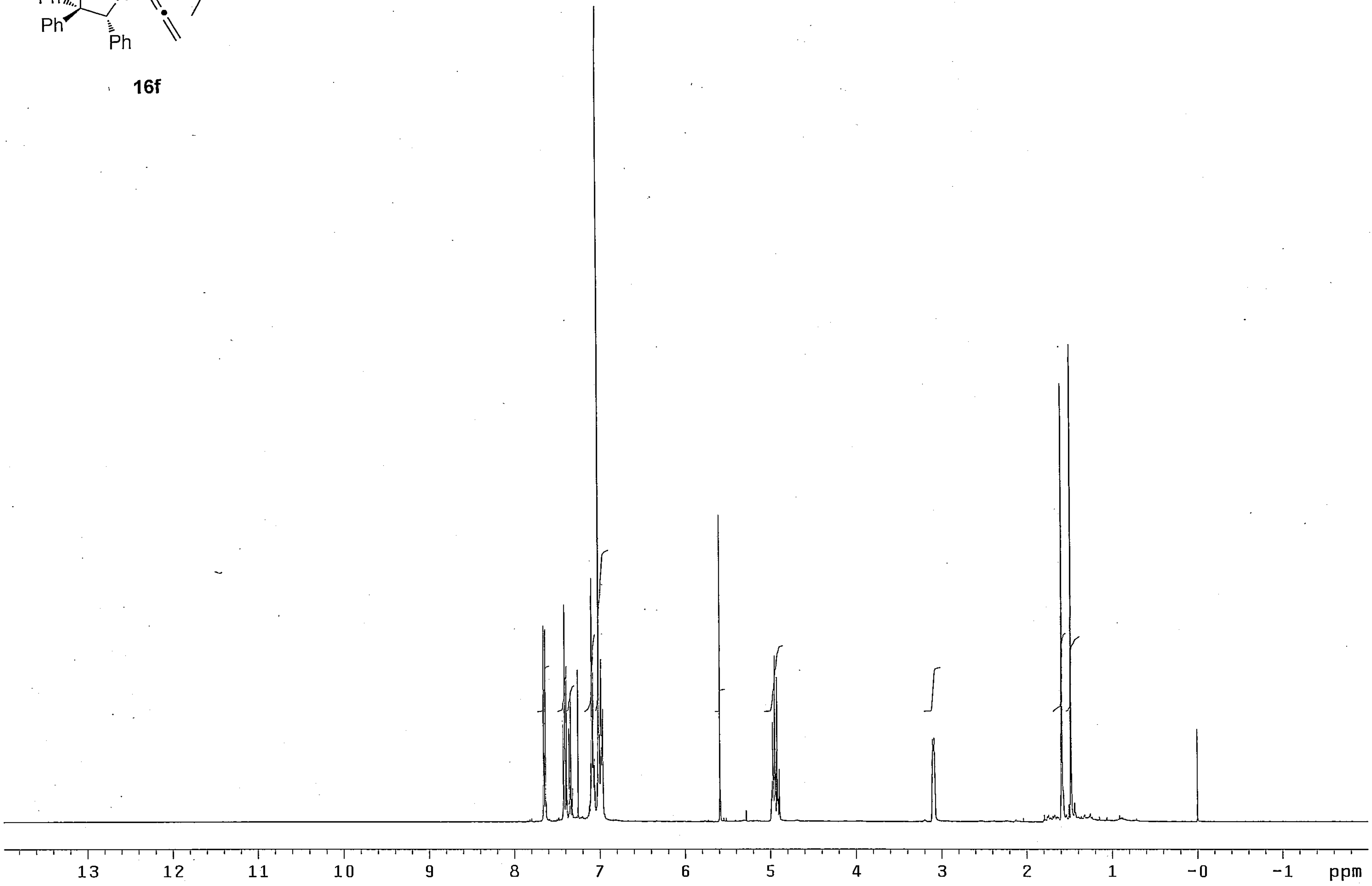

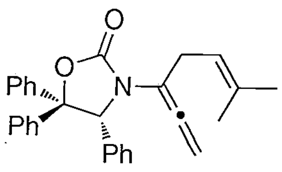

16f

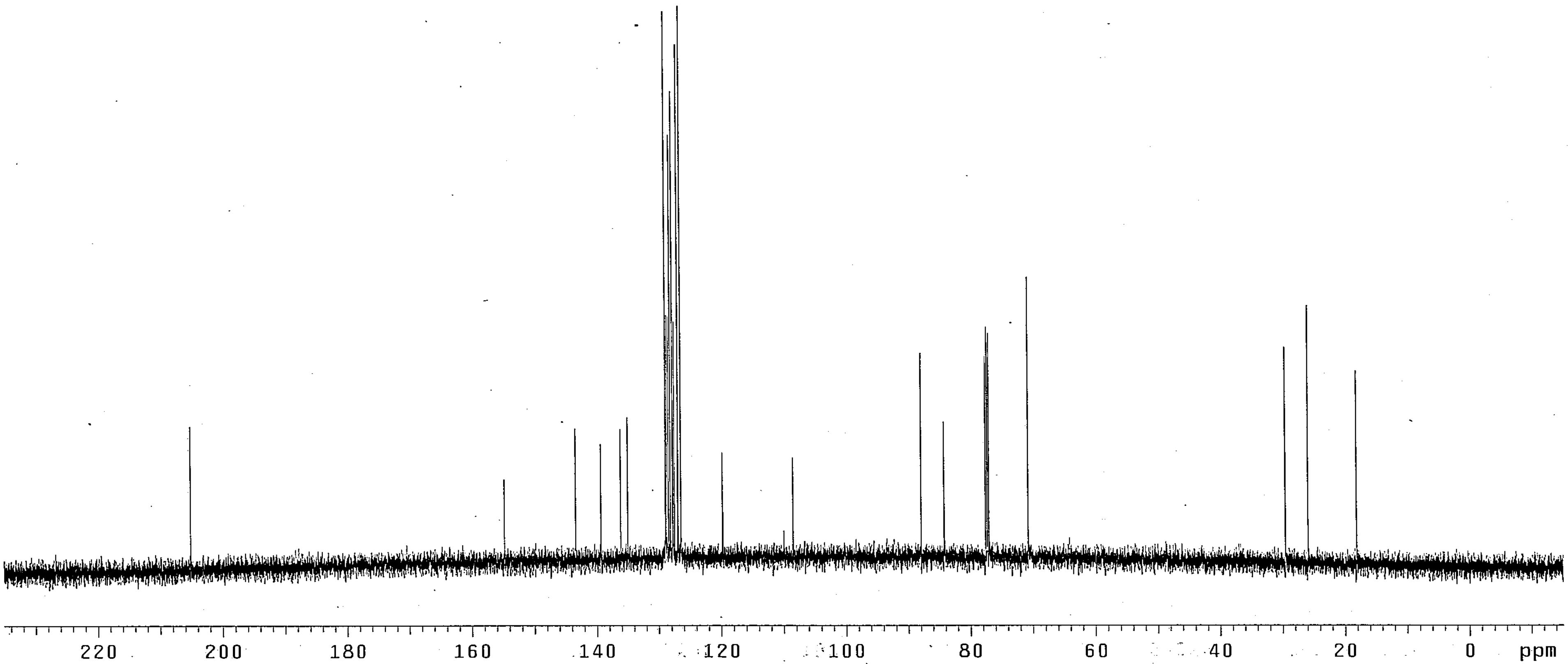

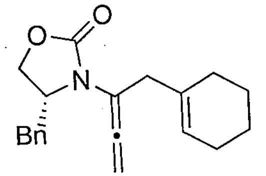

17

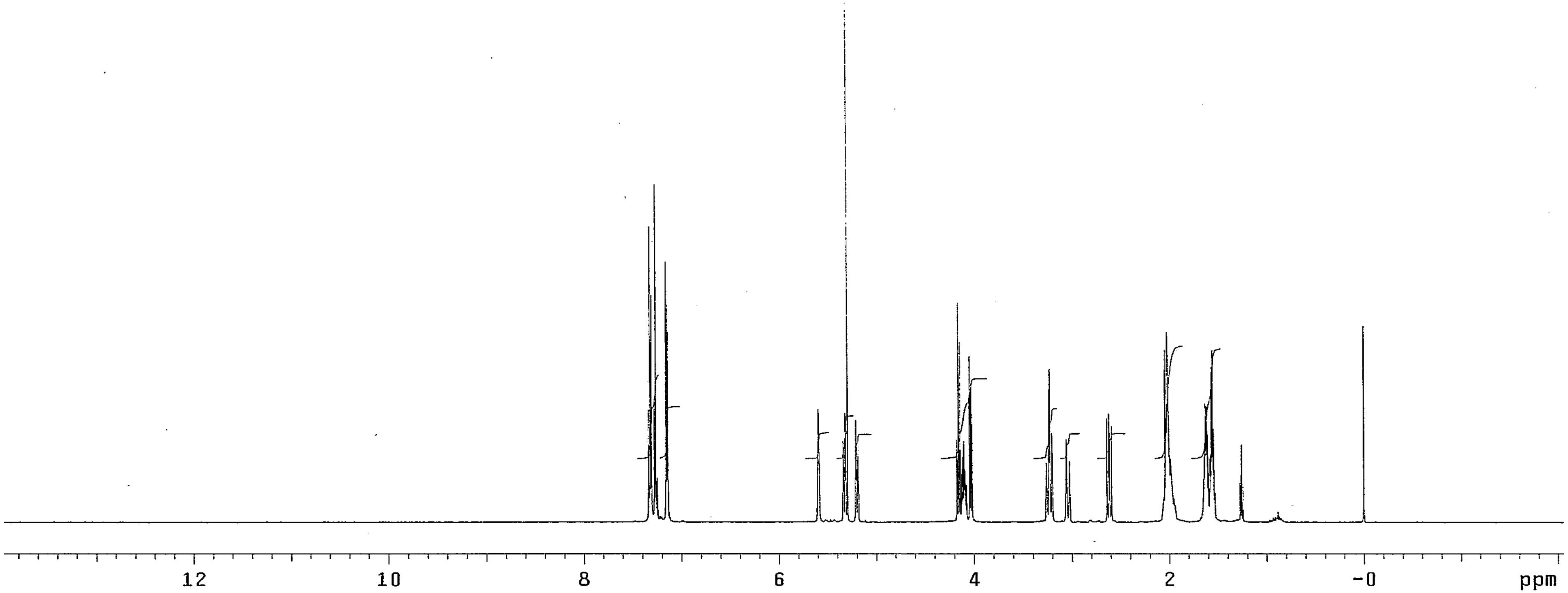

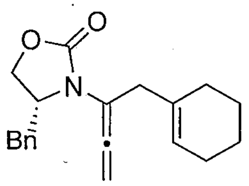

17

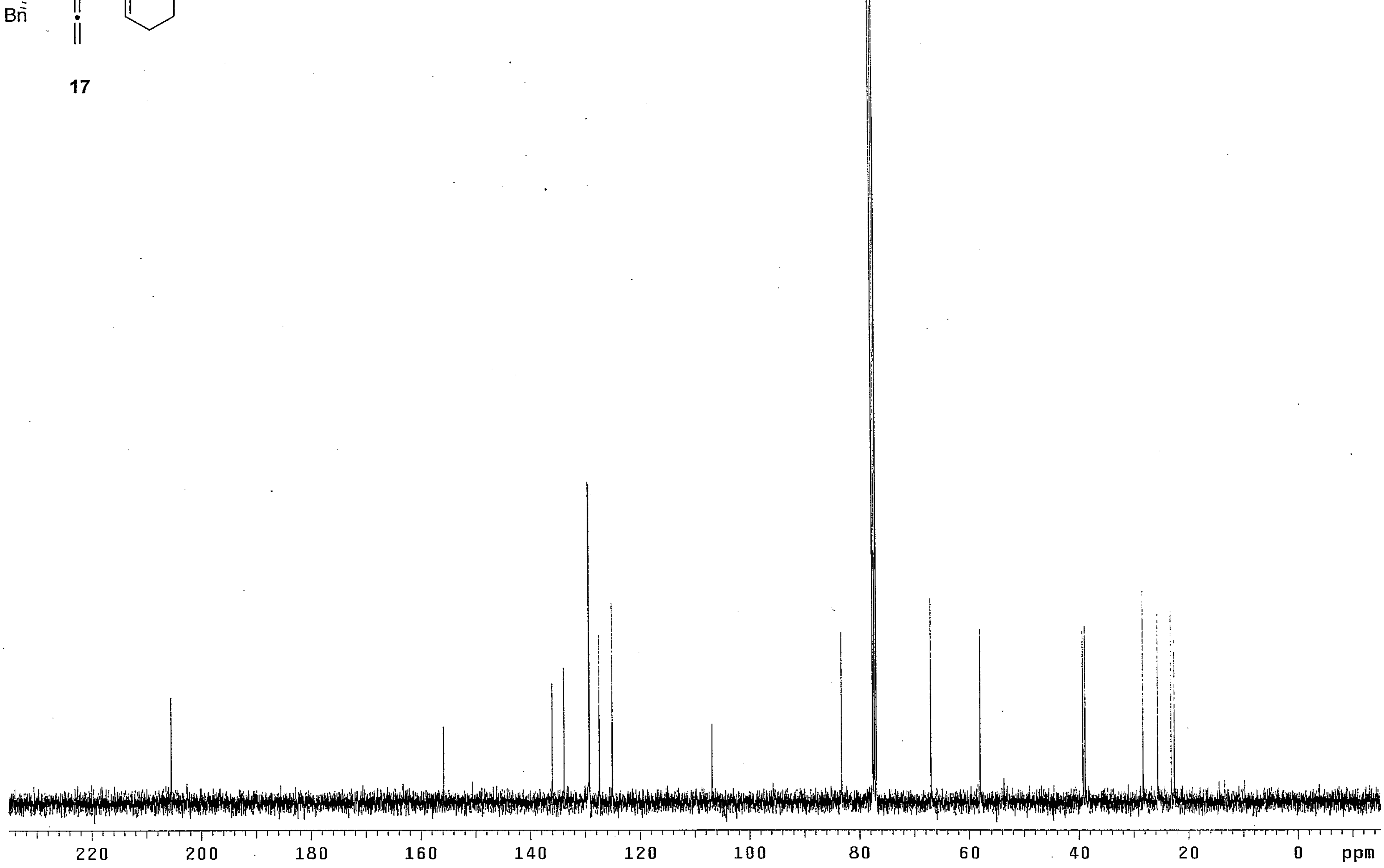

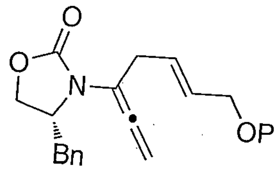

18a: P = TBDPS

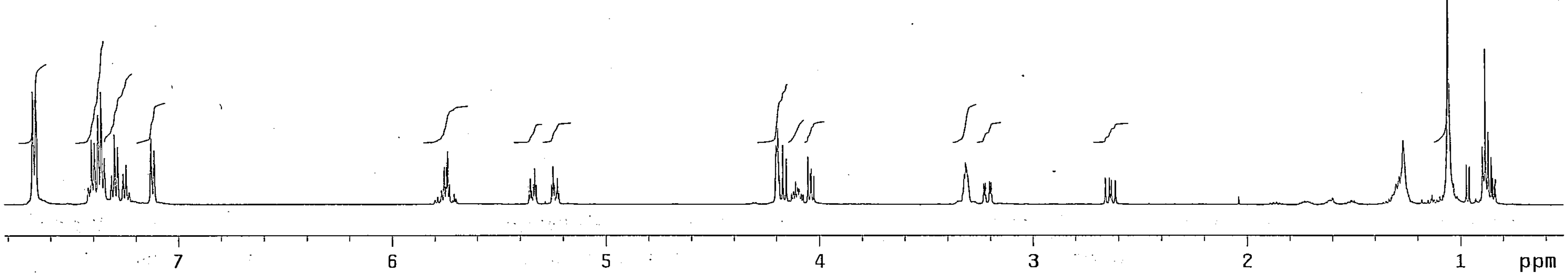

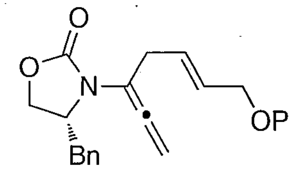

18a: P = TBDPS

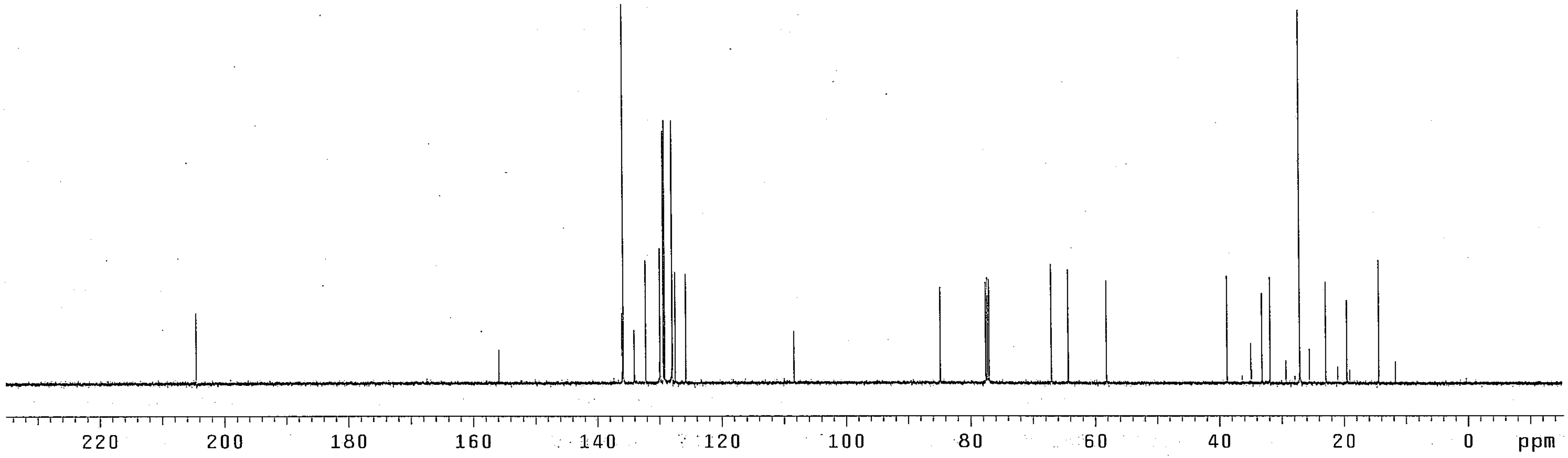

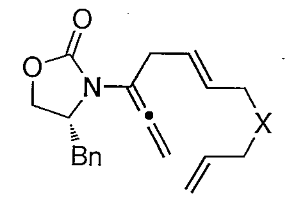

18b: X = O

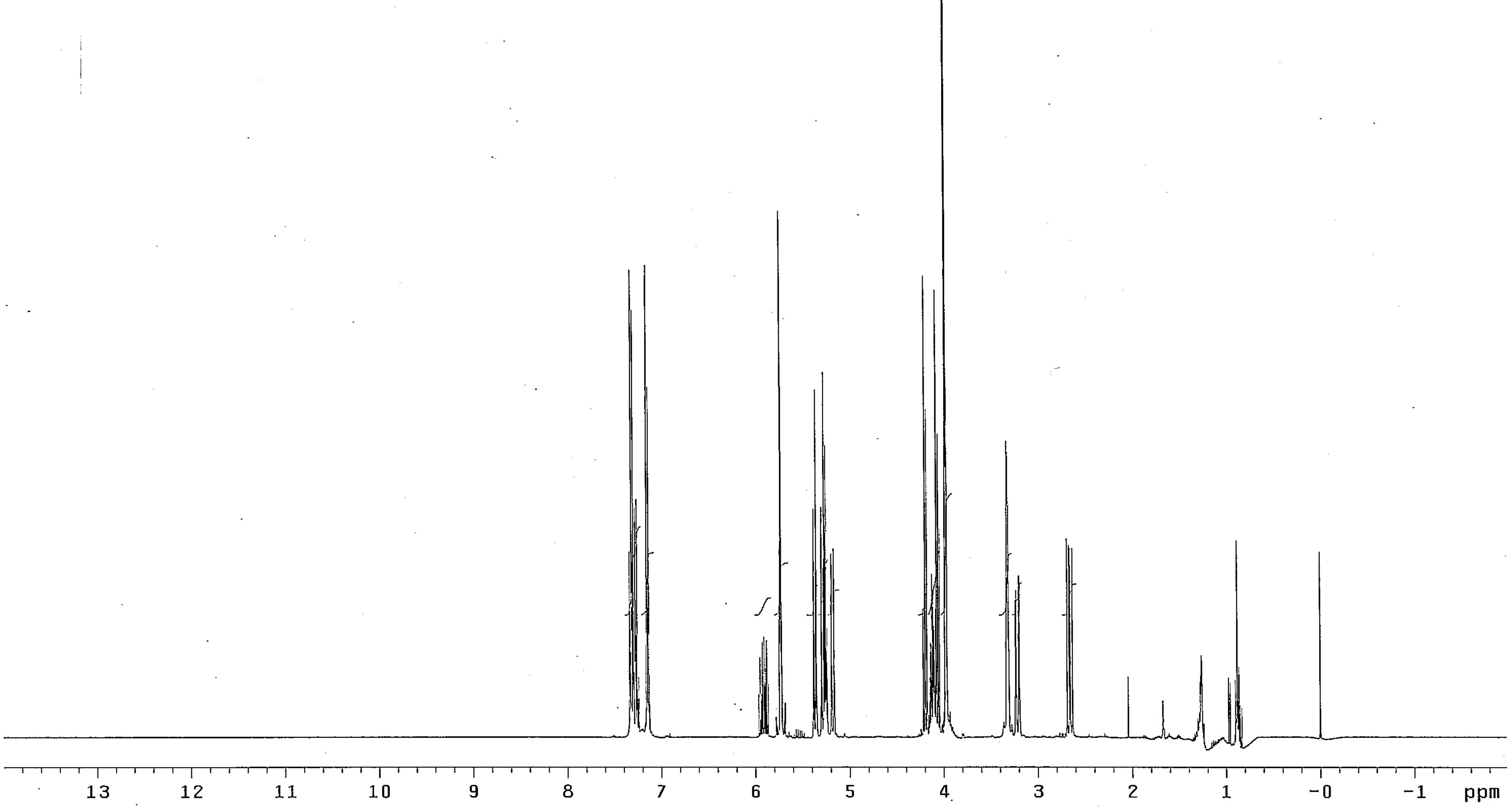

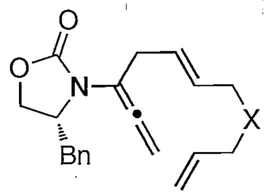

18b: X = O

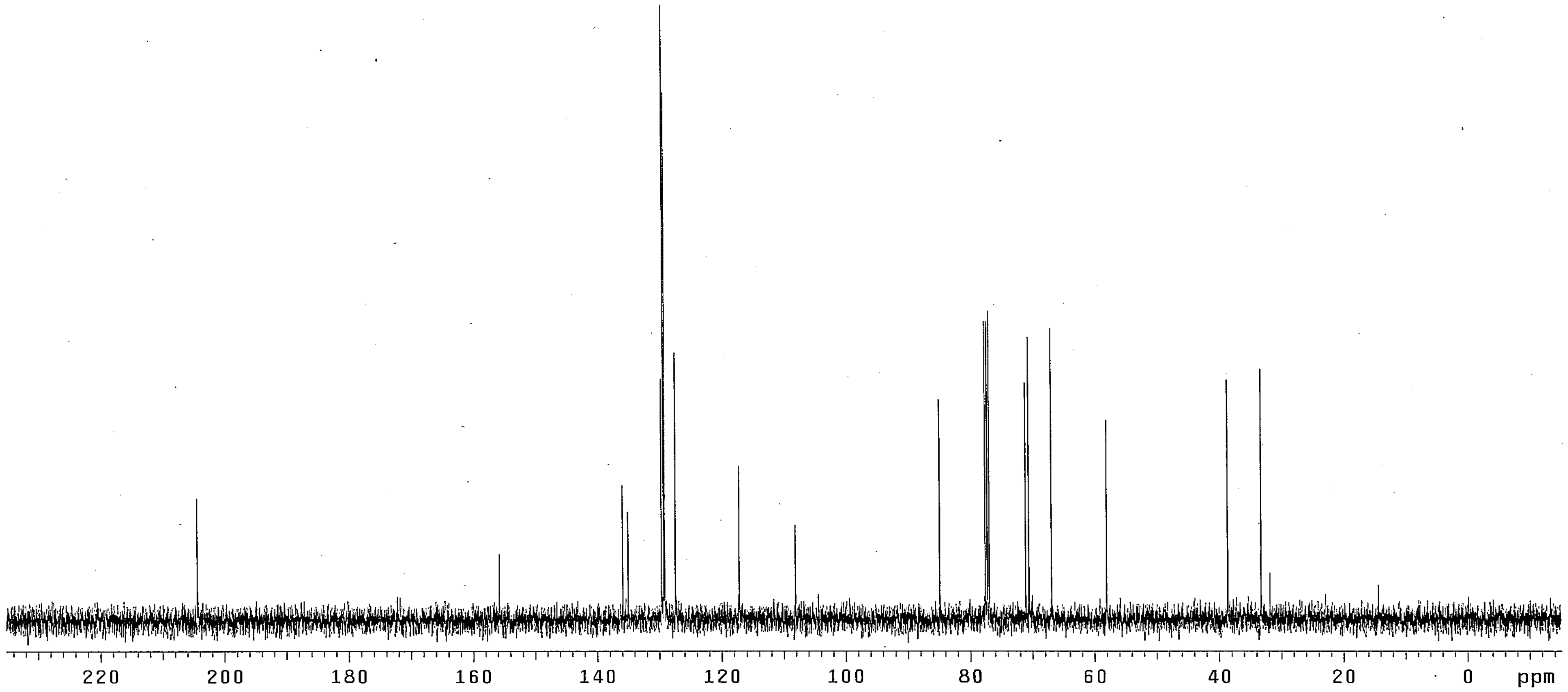

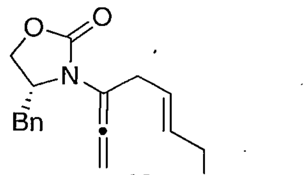

18c O-cinnamyl

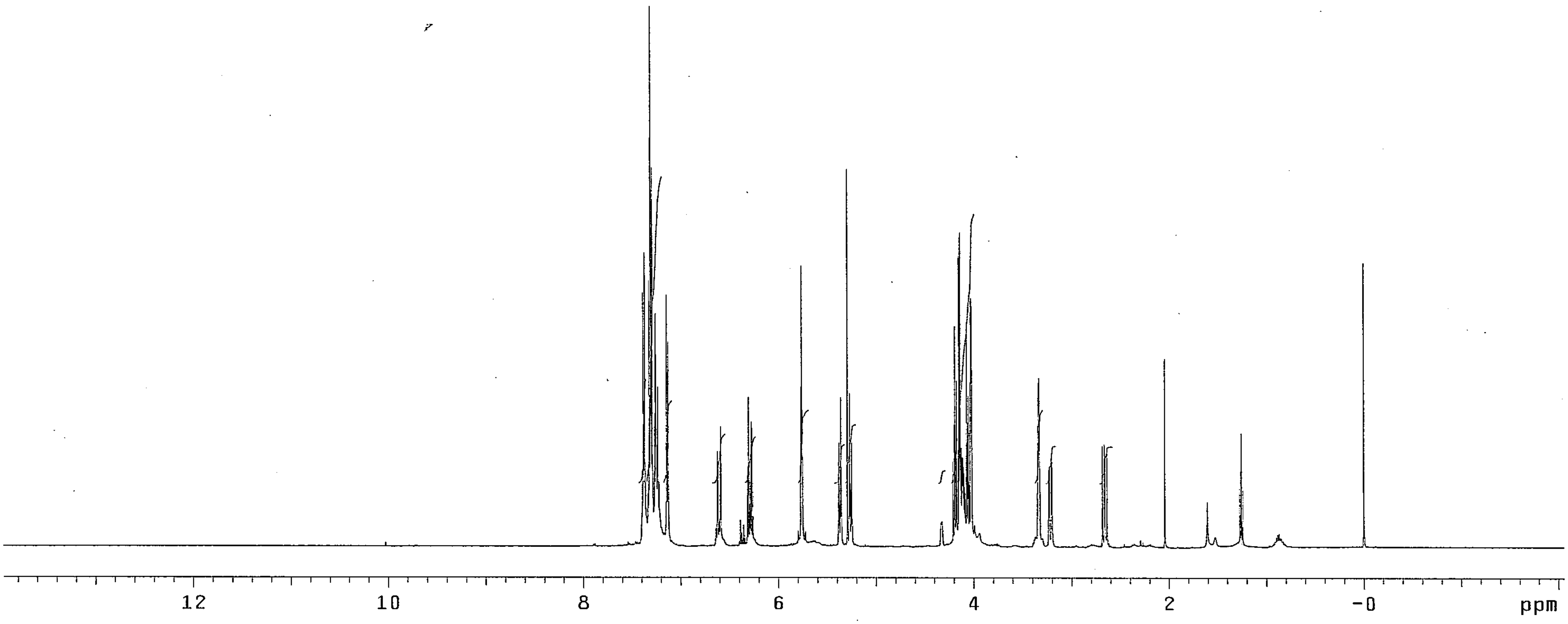

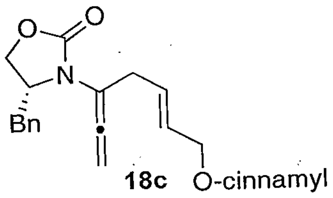

18c

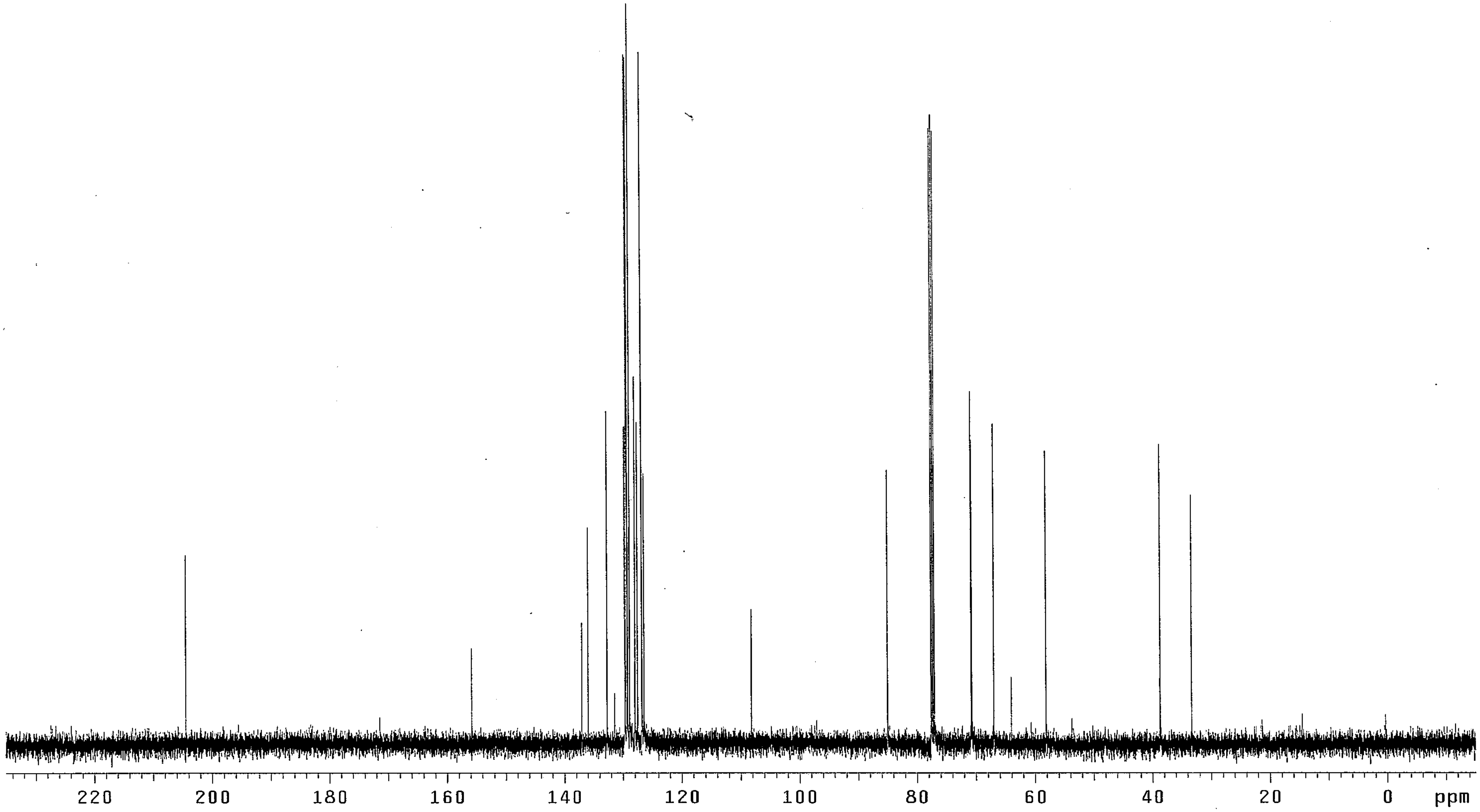

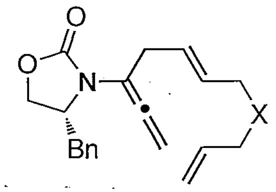

19: X = NTs

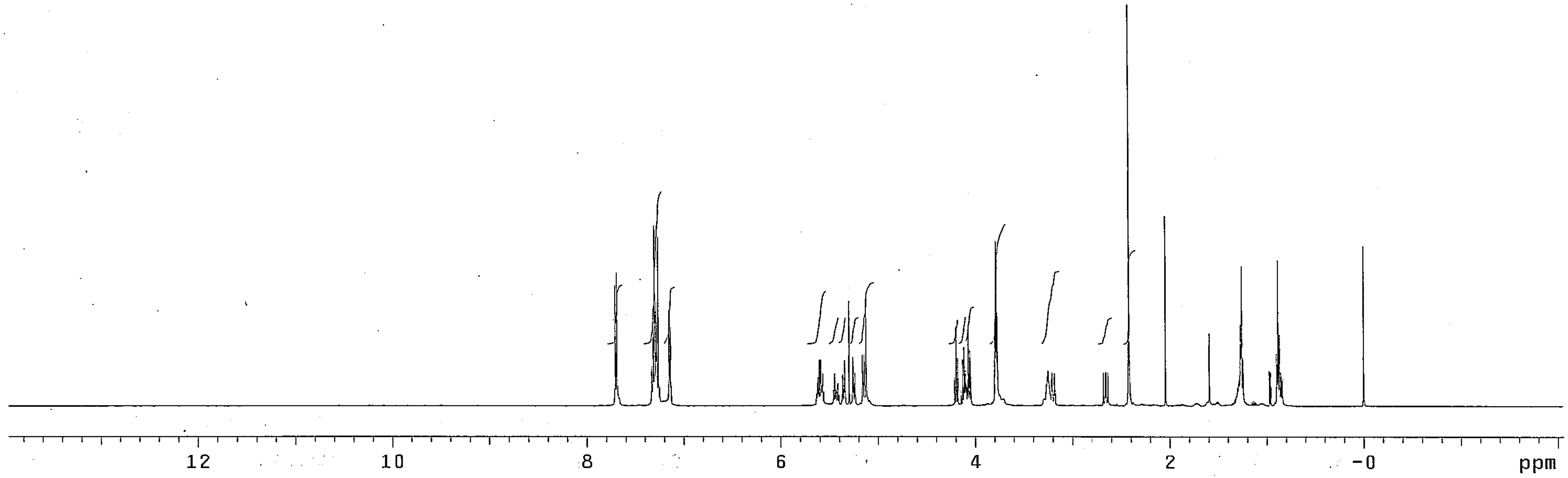

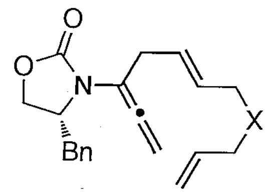

19: X = NTs

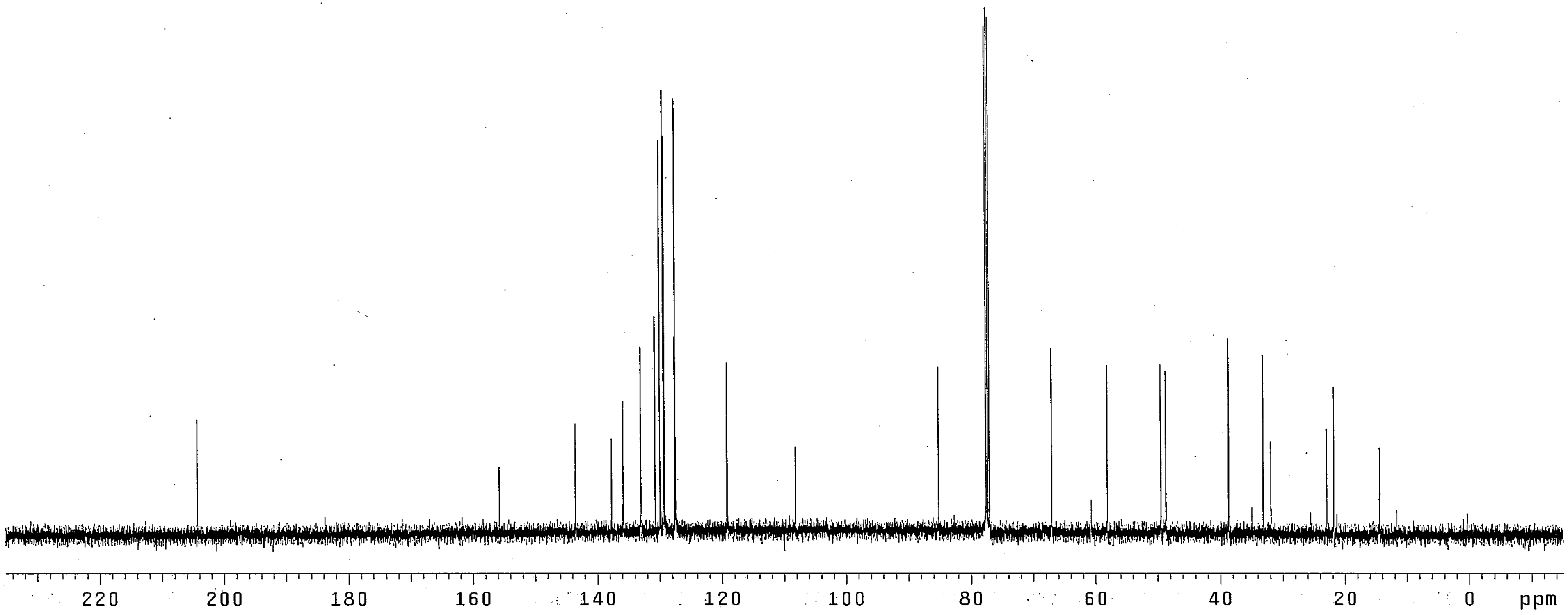

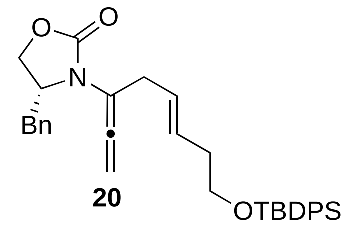

20

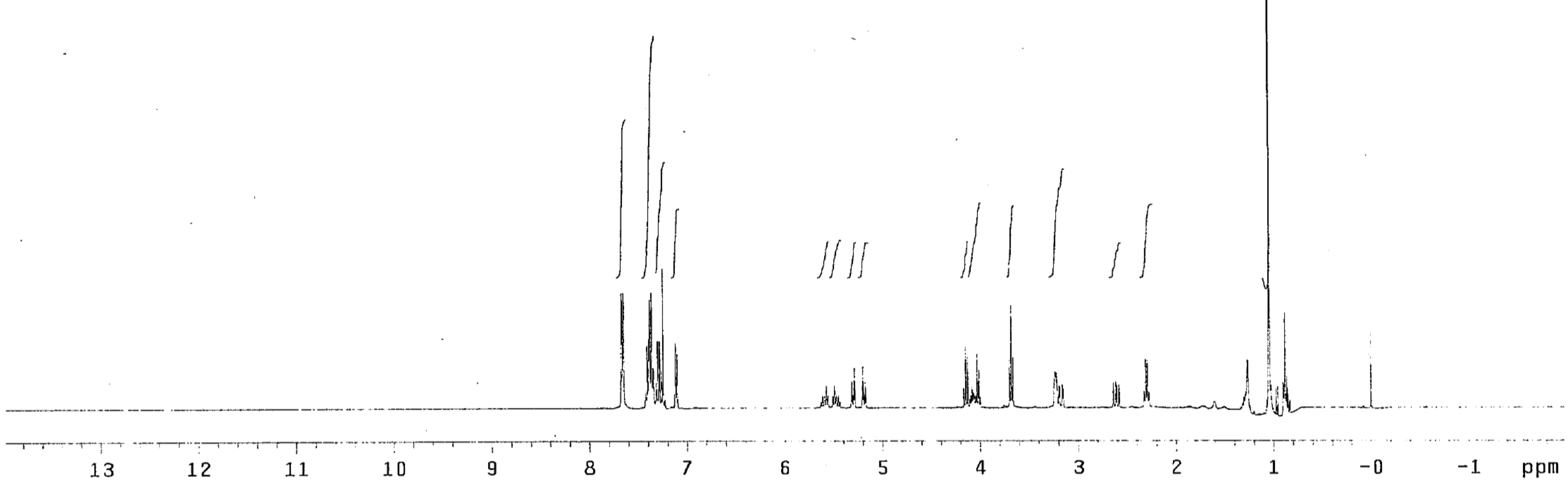

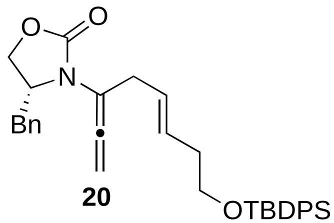

20

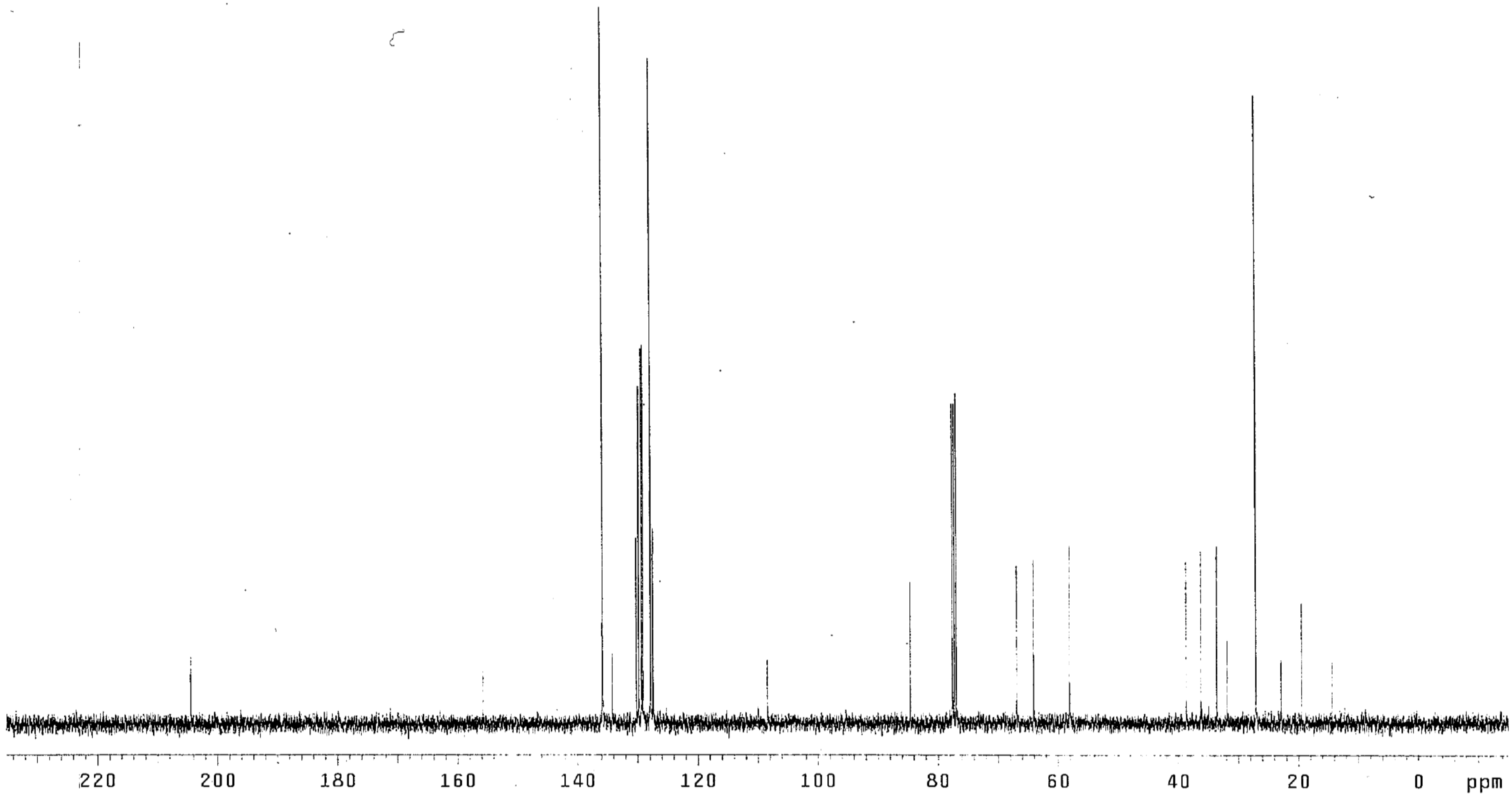

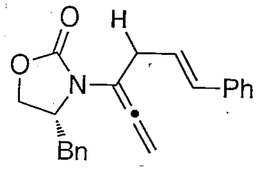

21

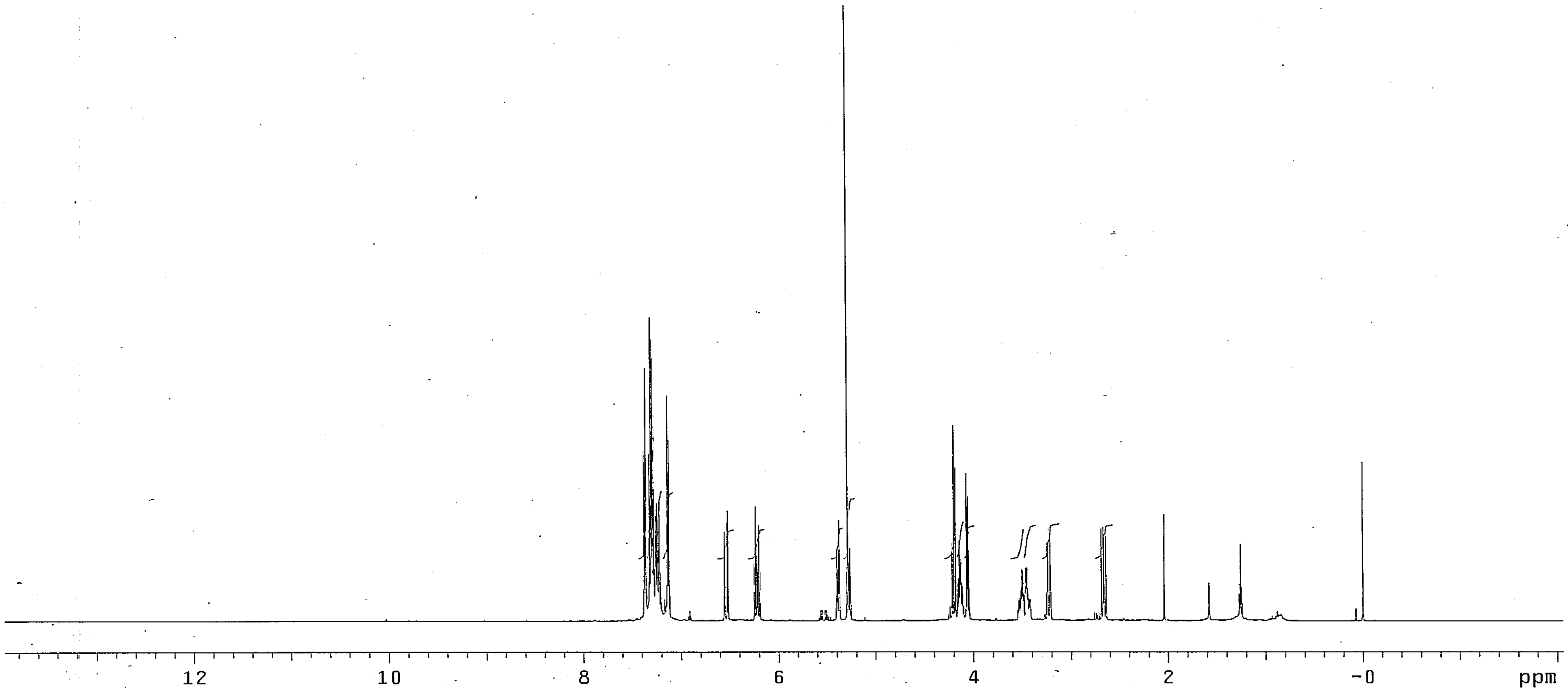

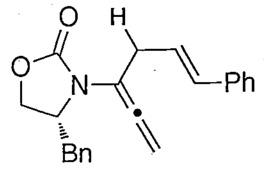

21

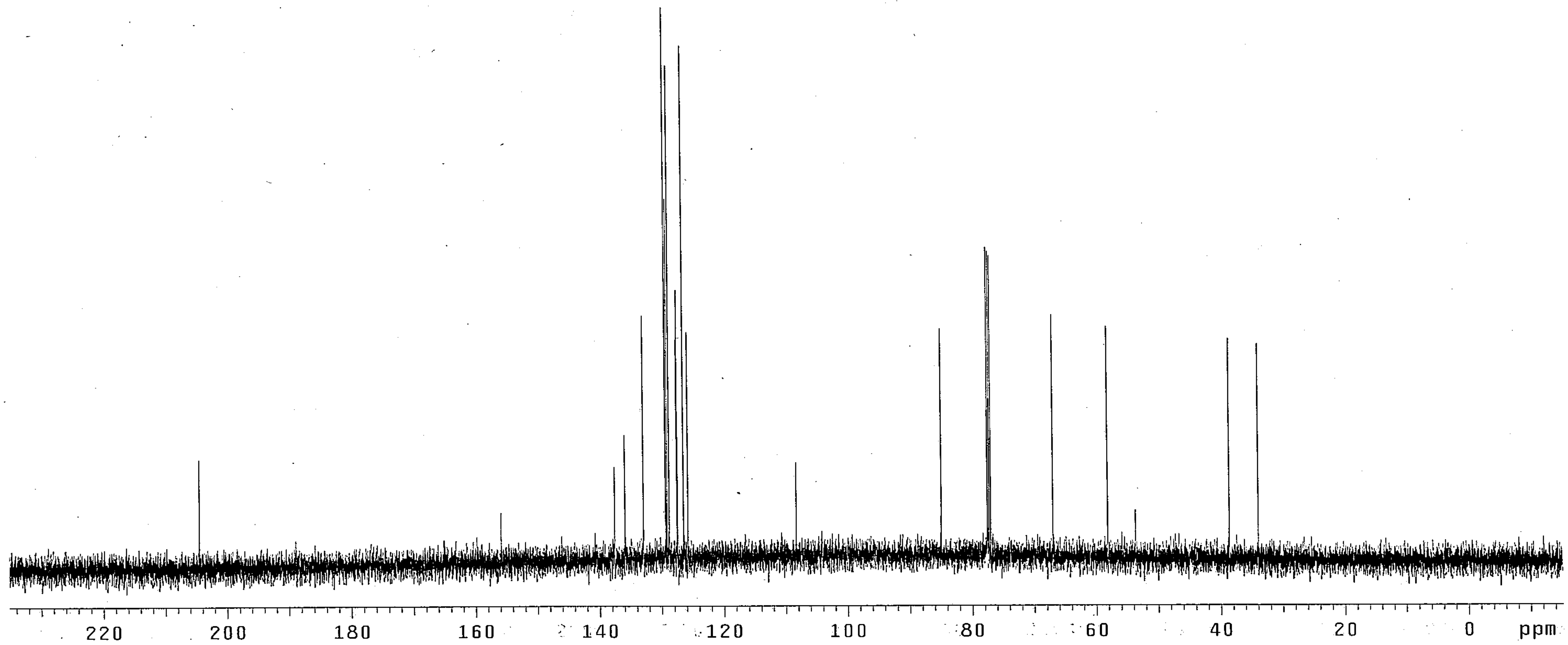

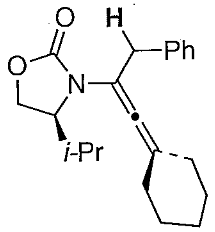

29a

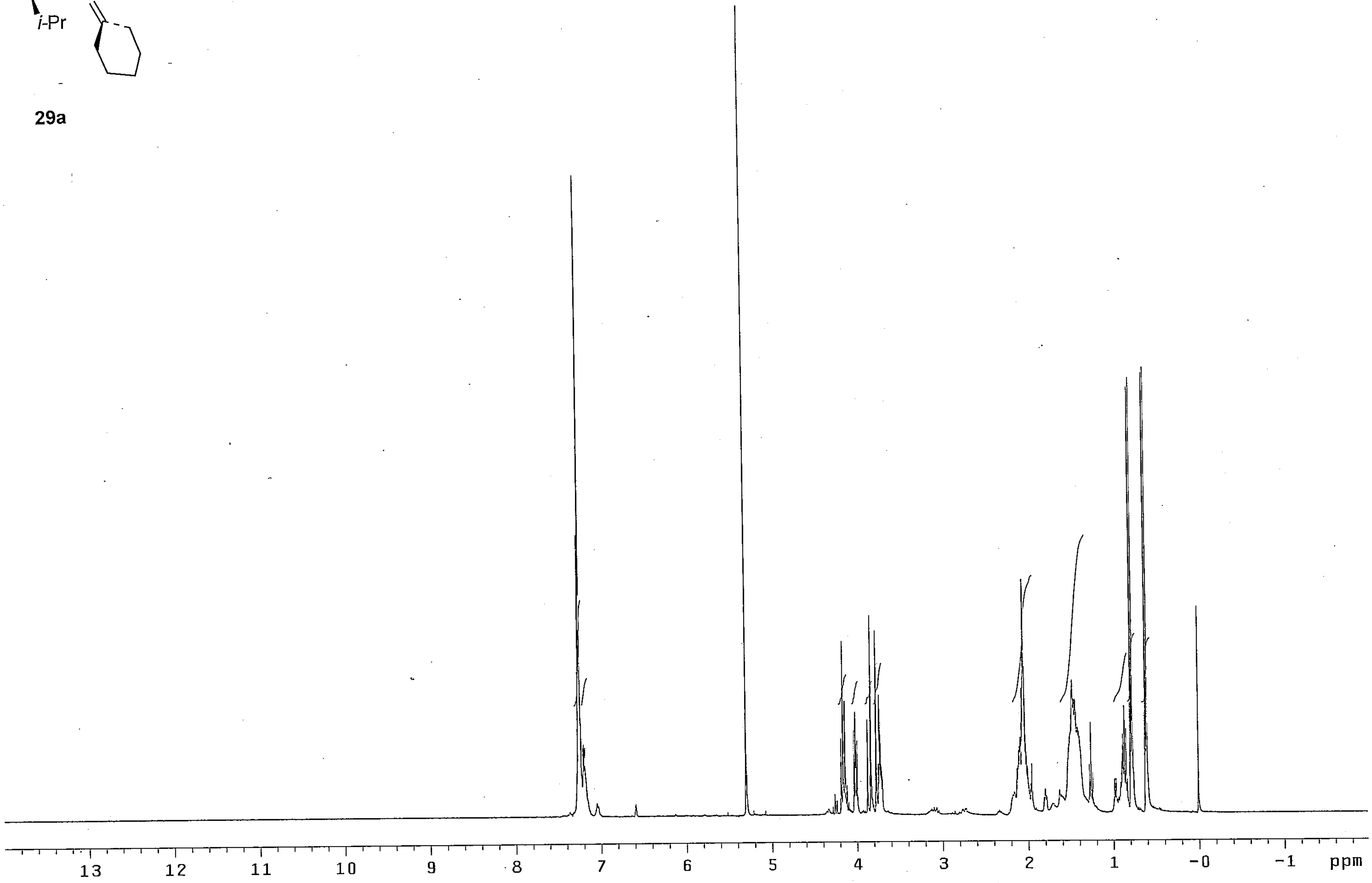

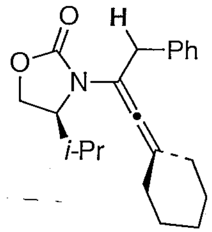

29a

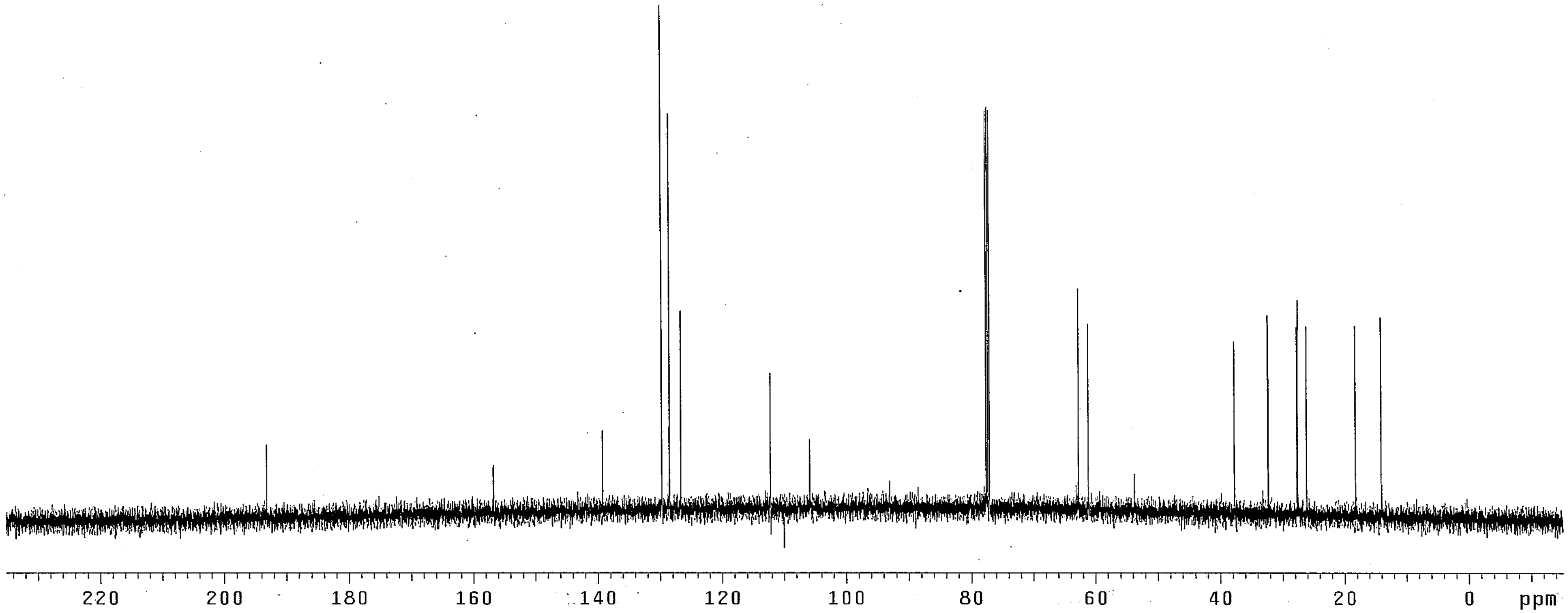

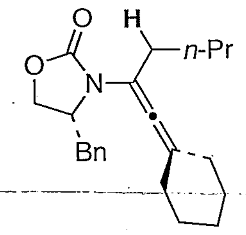

30

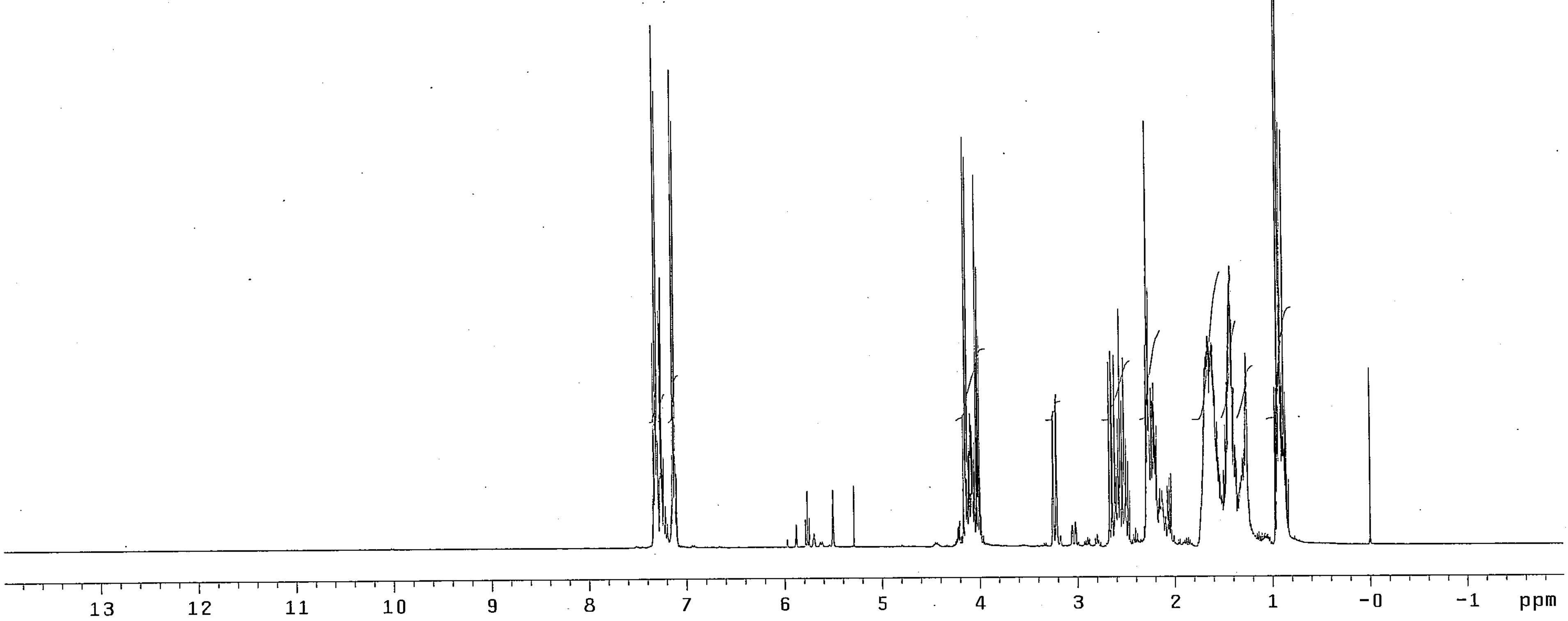

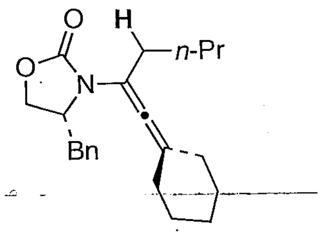

30

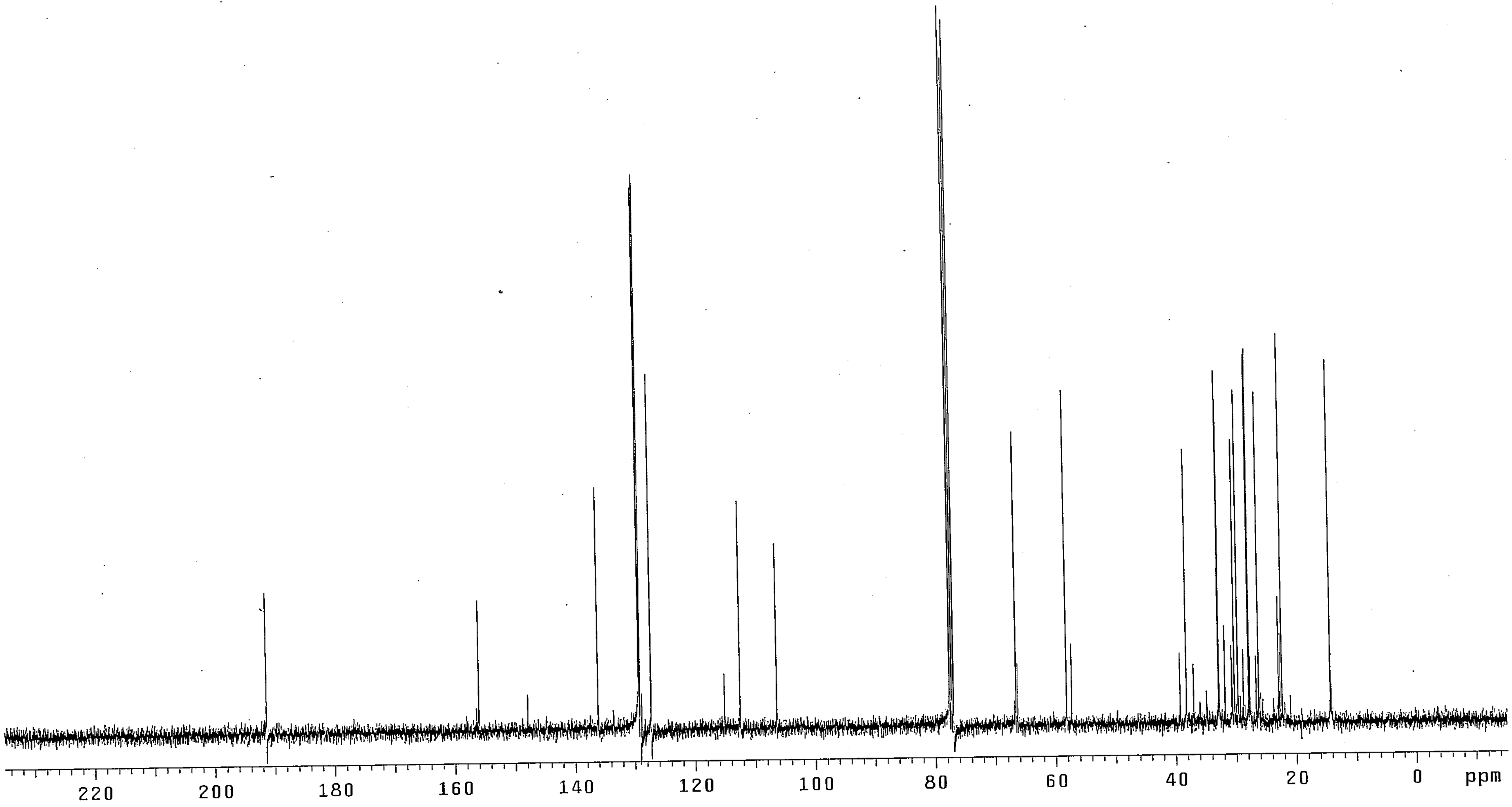

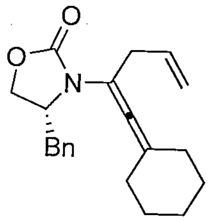

31a

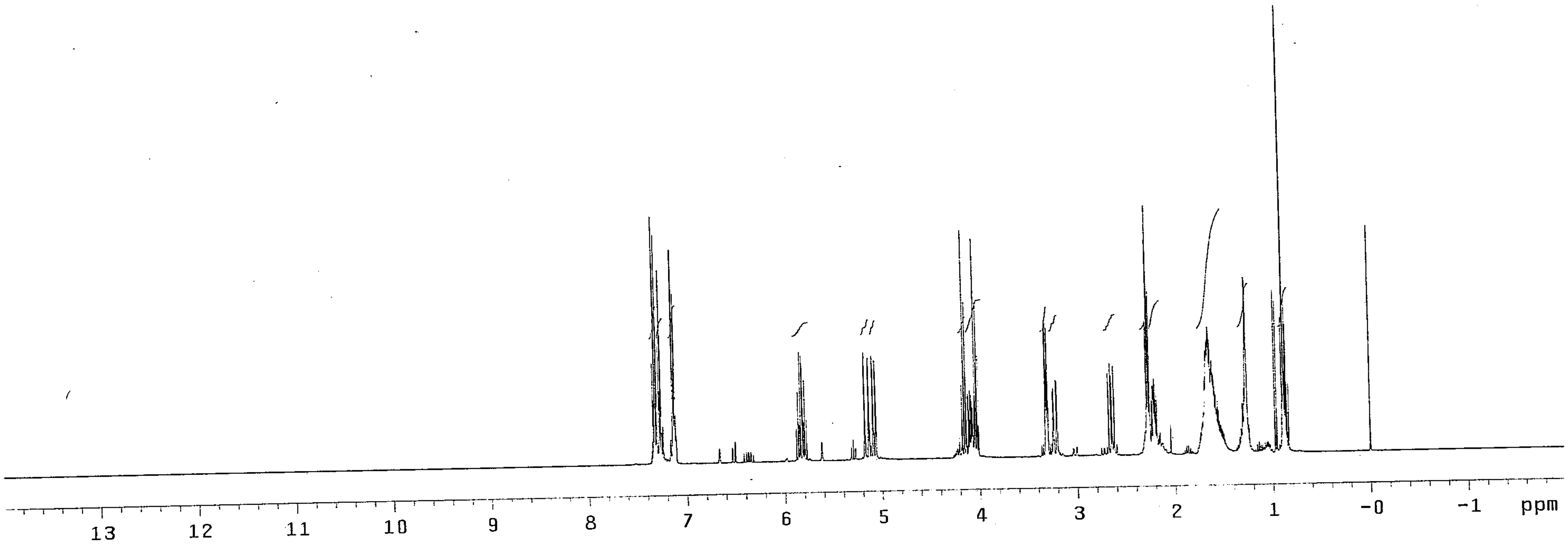

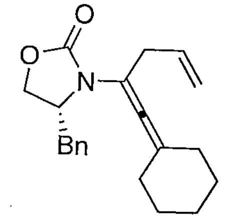

31a

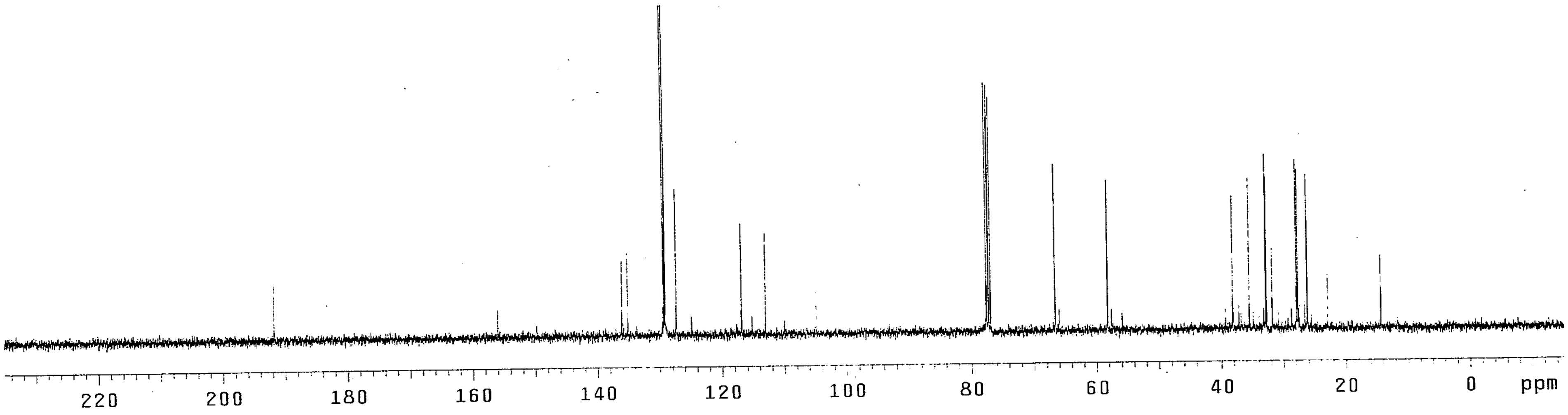

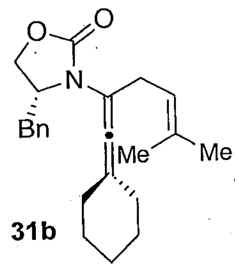

31b

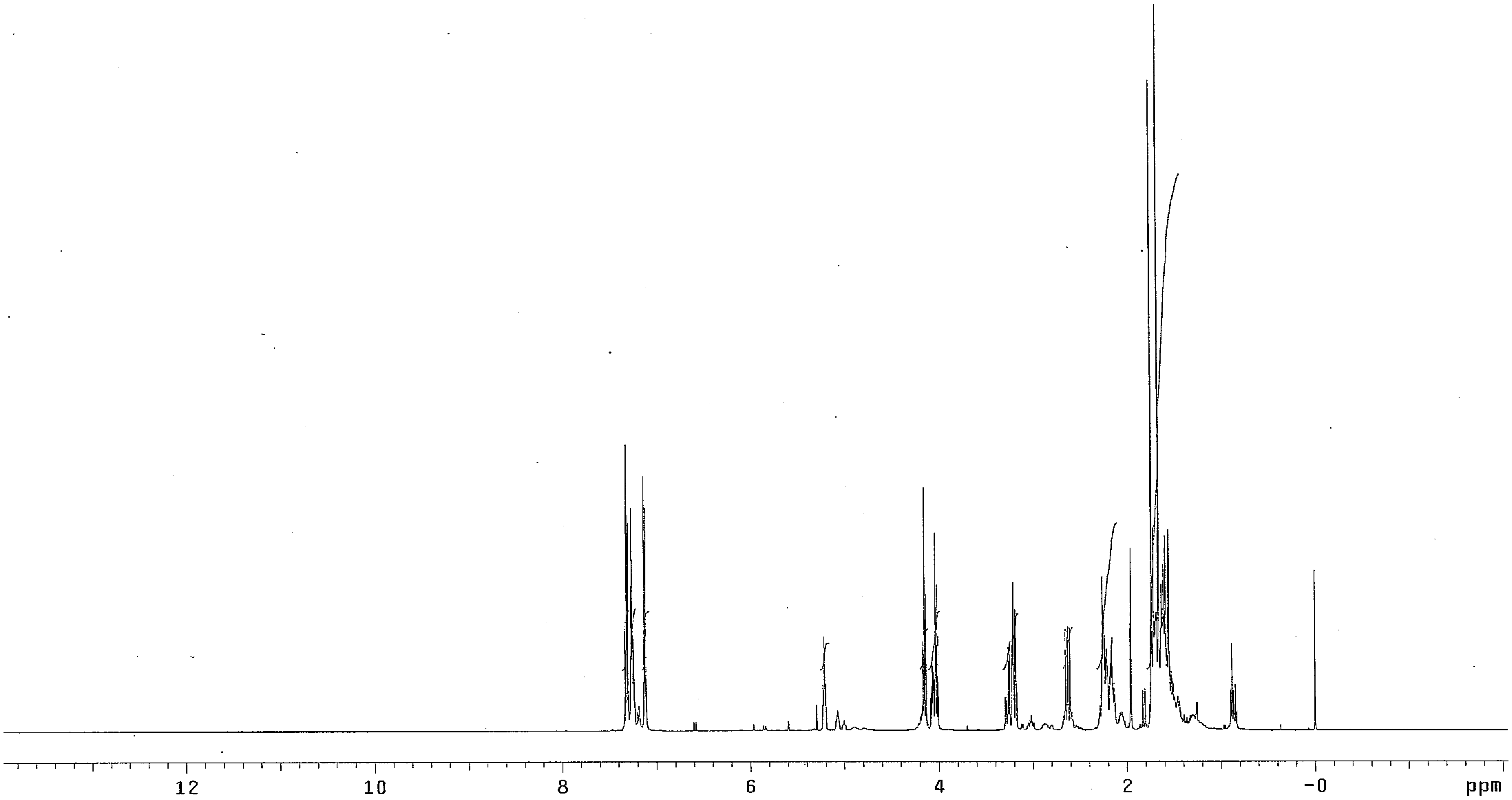

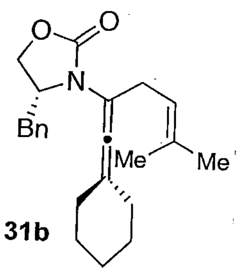

31b

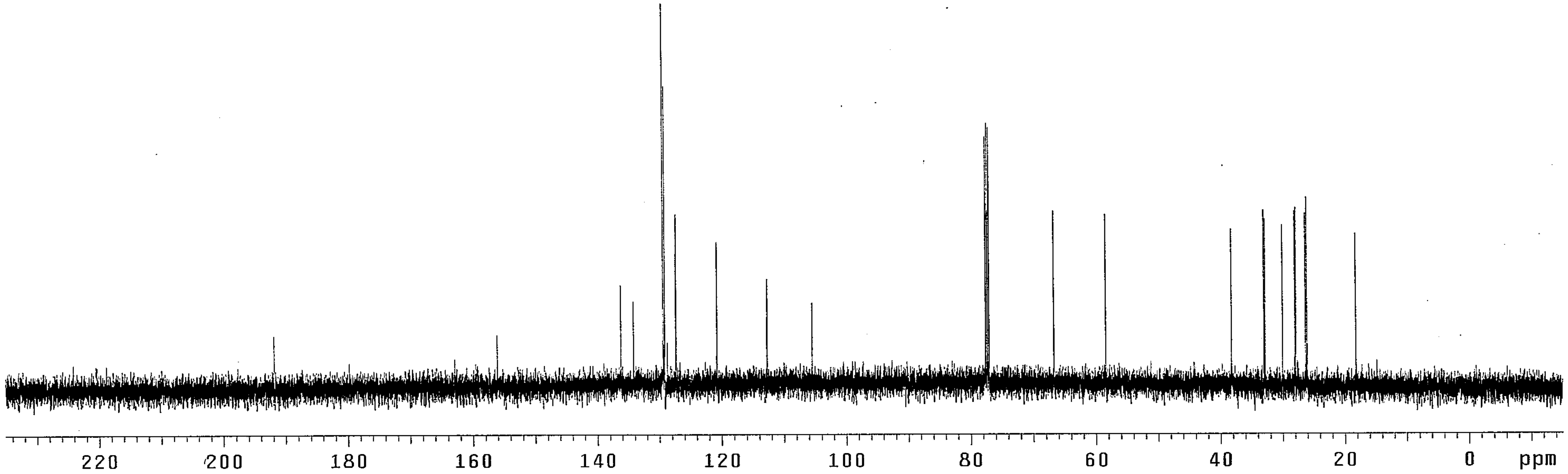

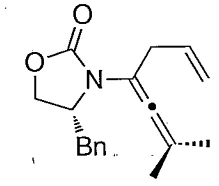

32

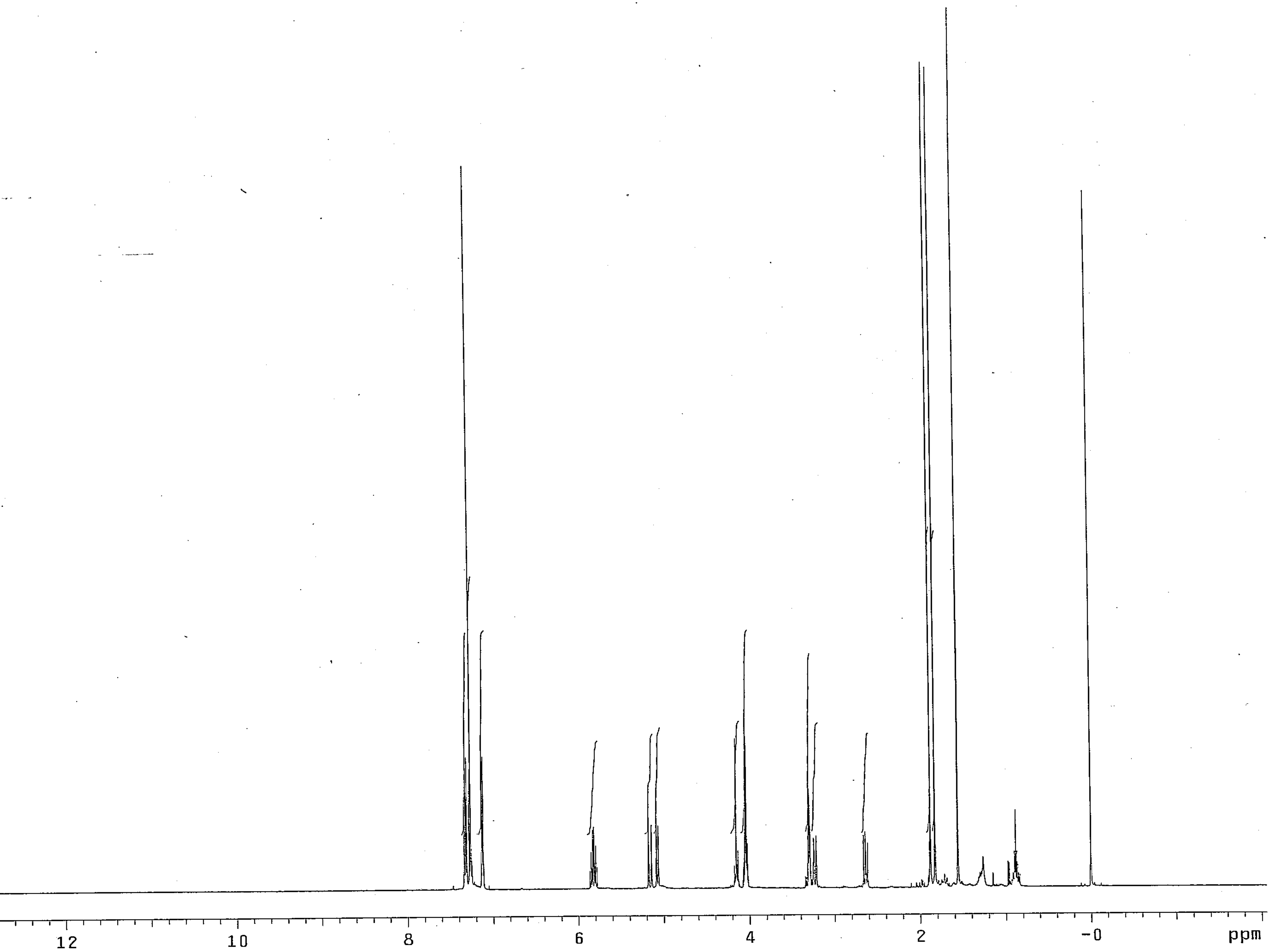

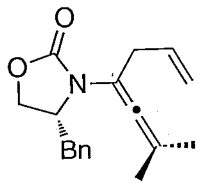

32

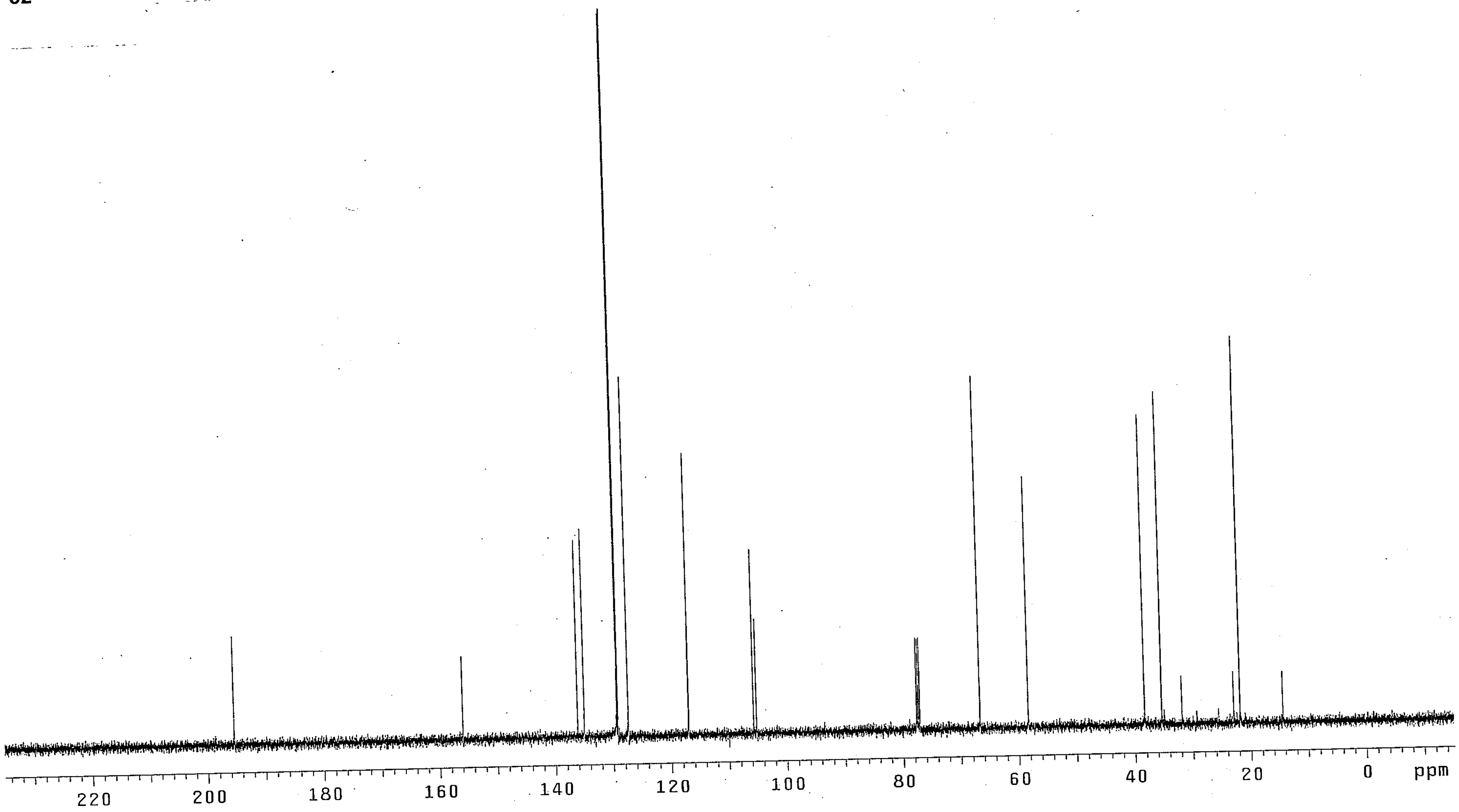

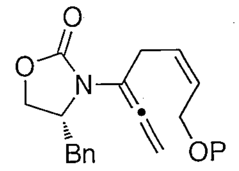

37a: P = TBDPS

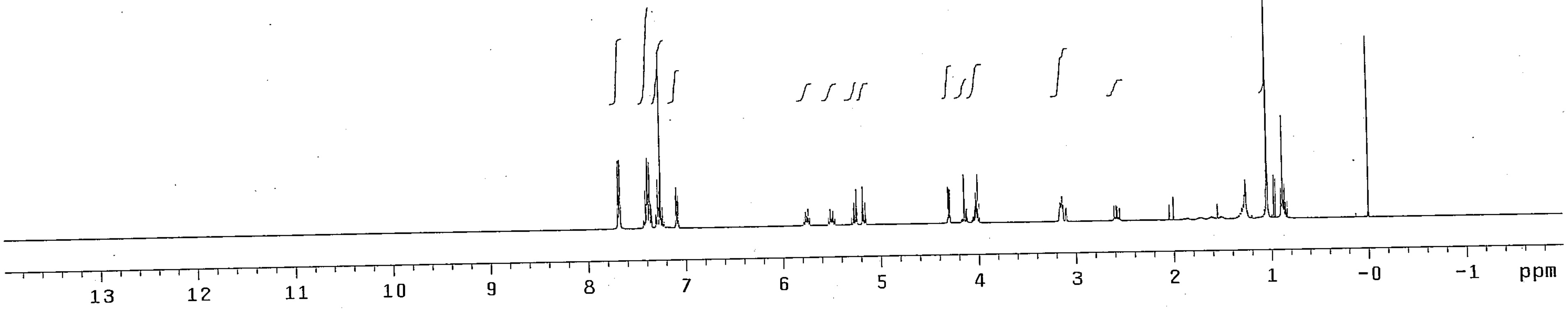

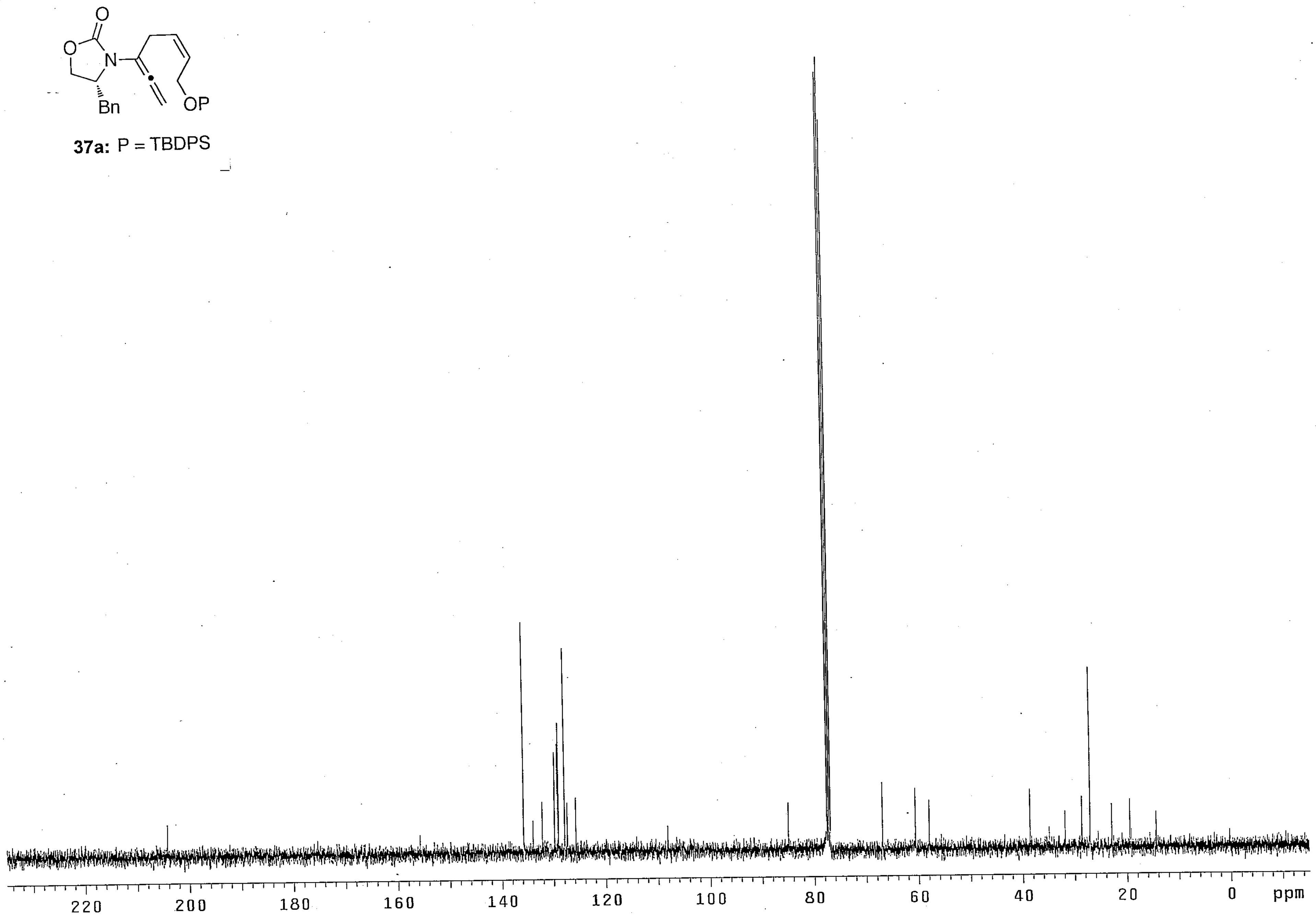

37a: P = TBDPS

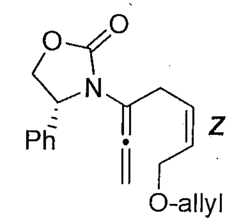

37b

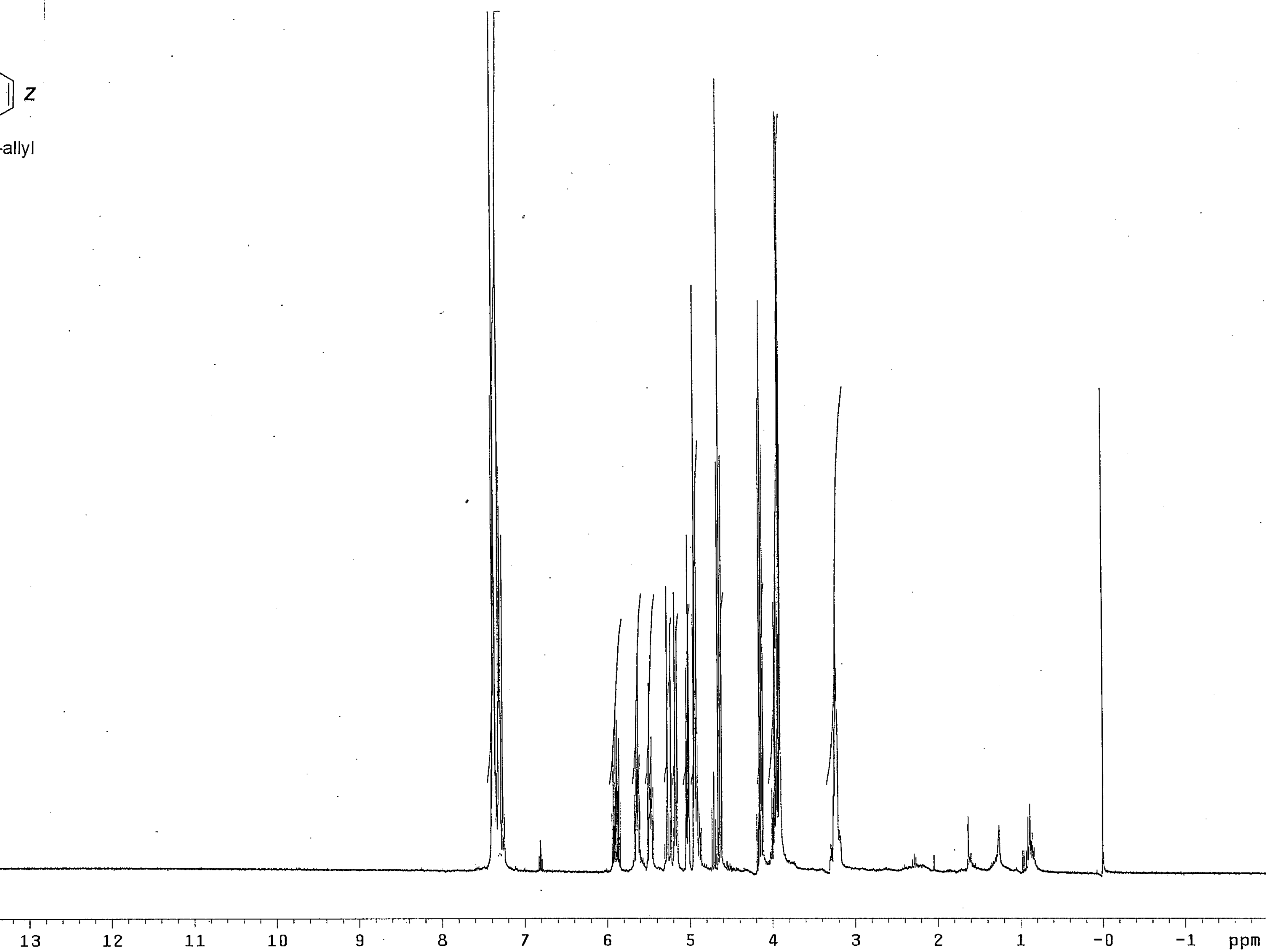

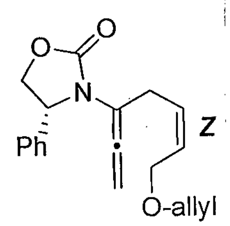

37b

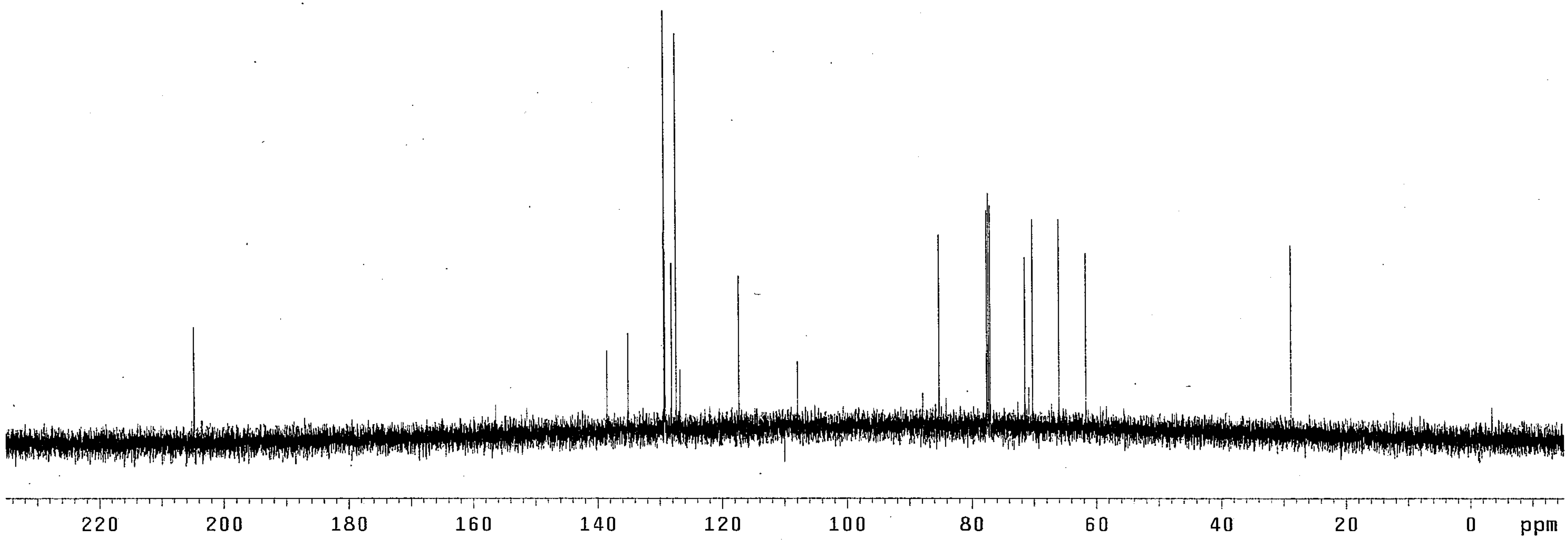

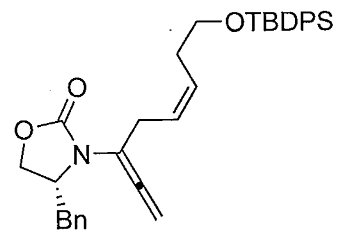

40

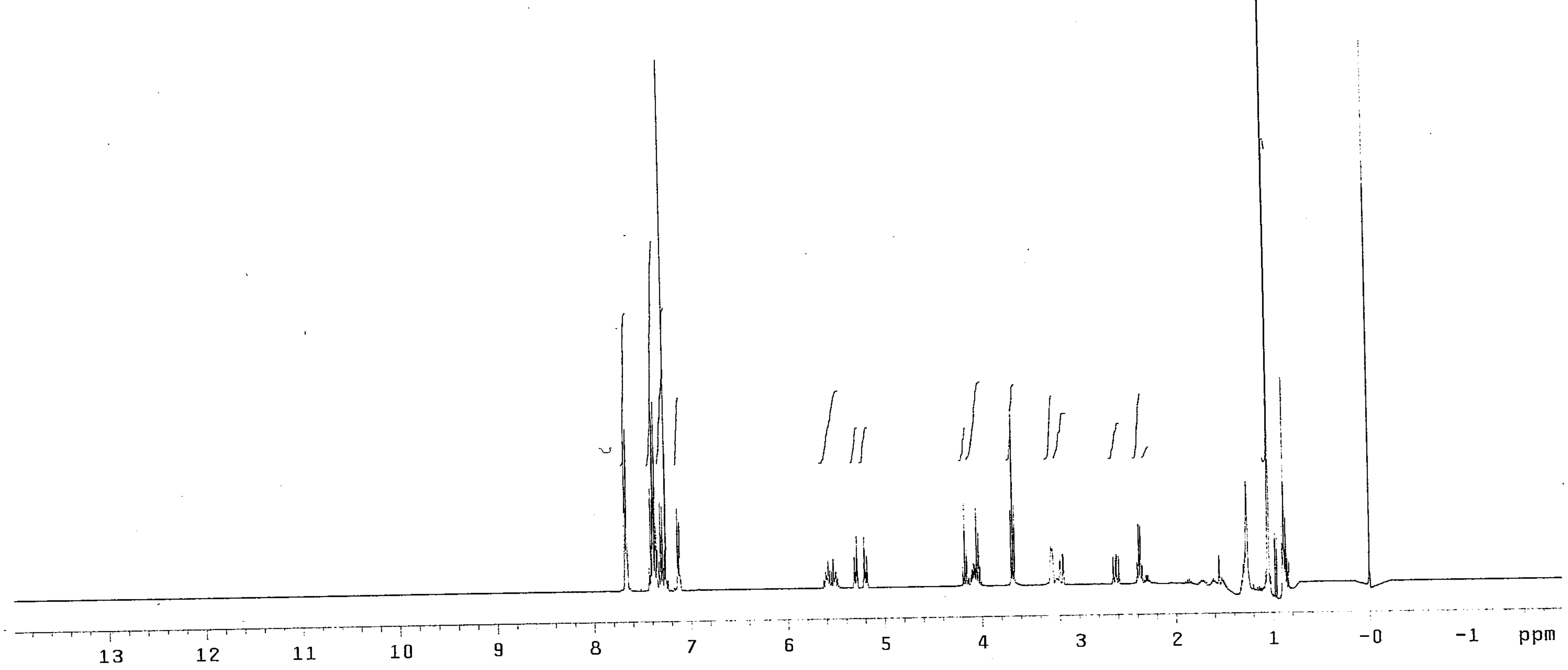

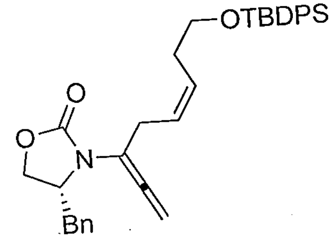

40

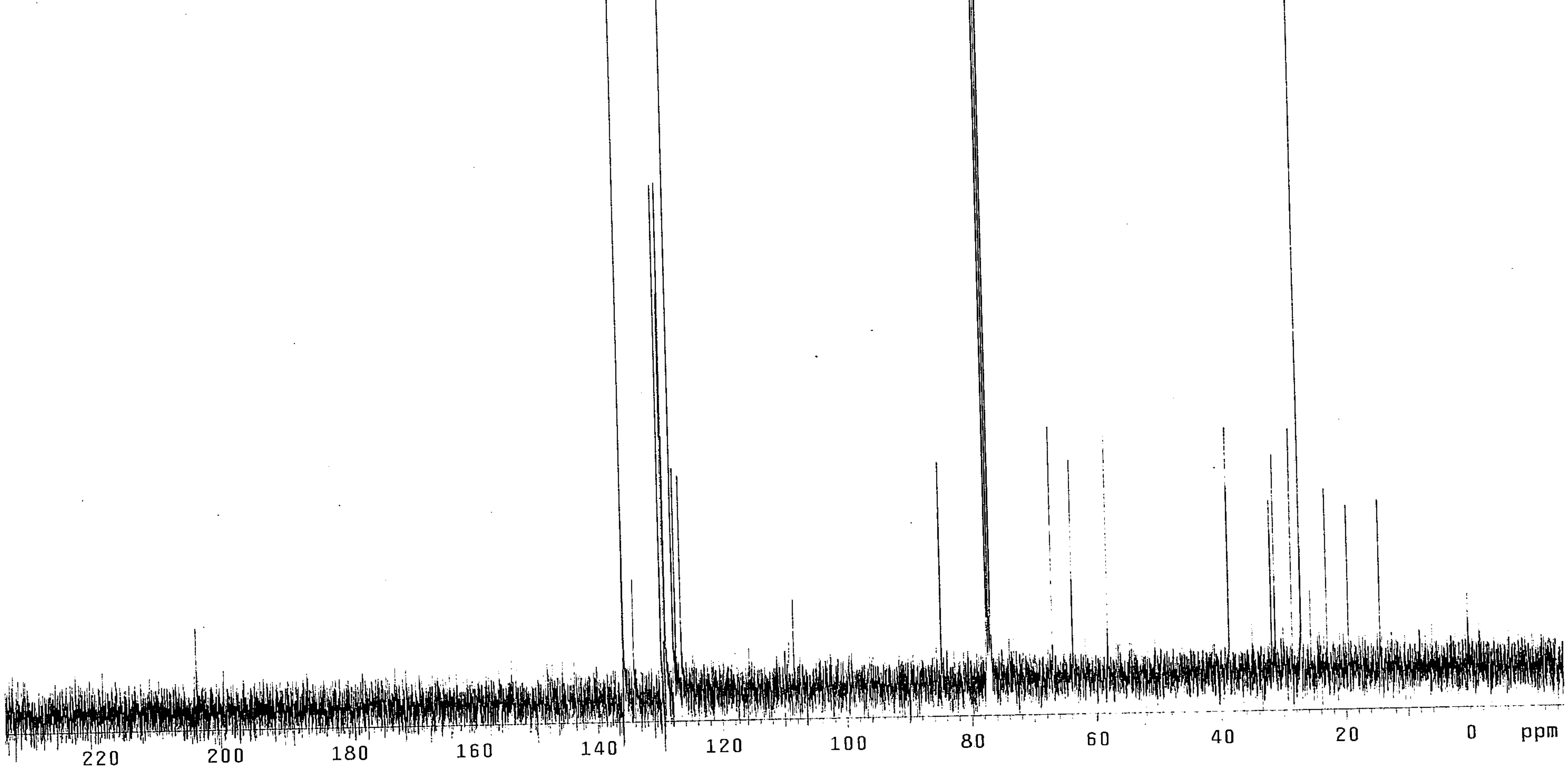

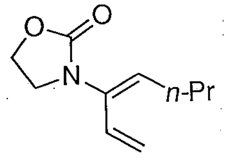

2

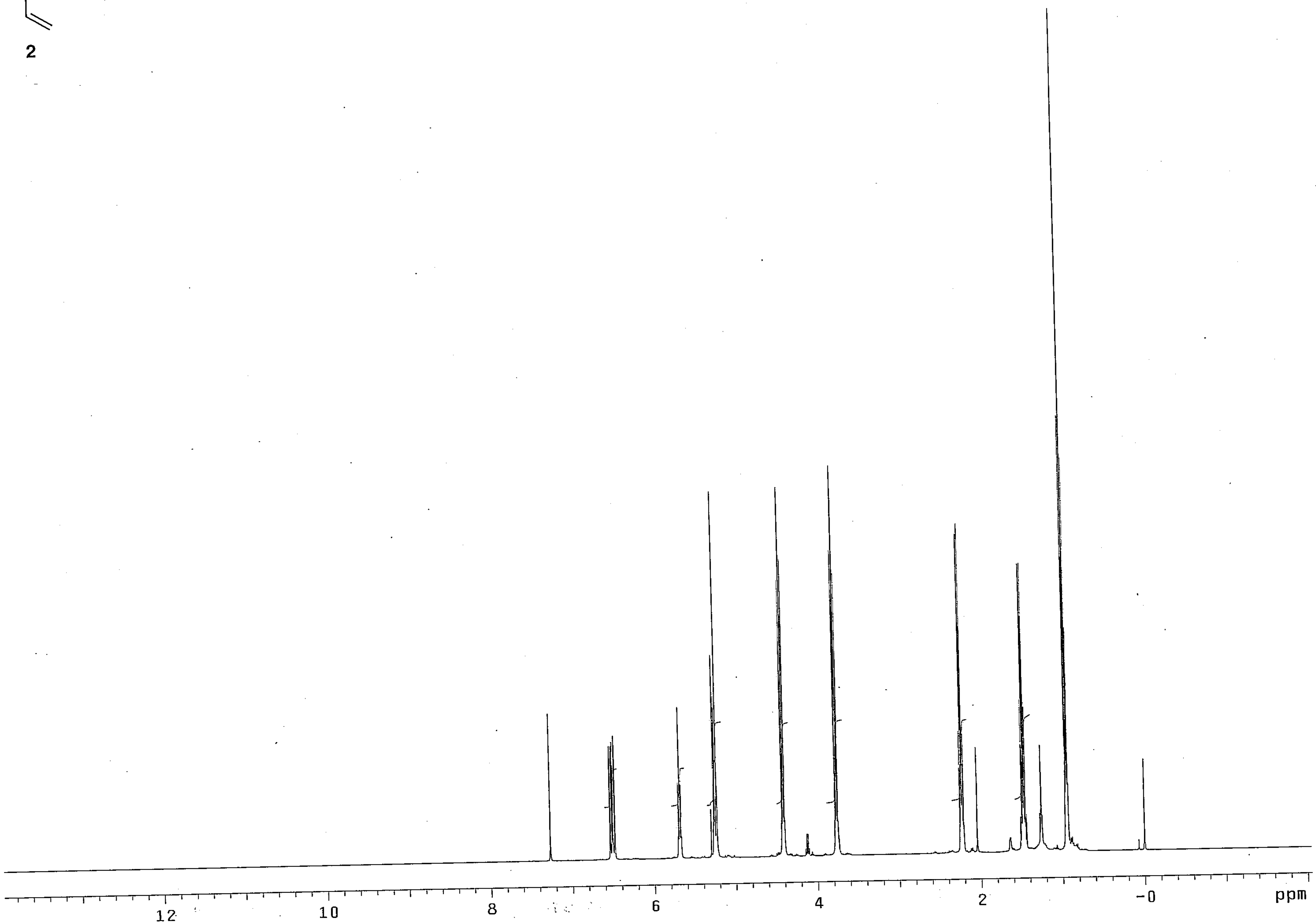

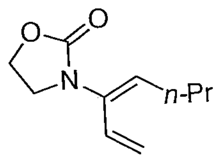

2

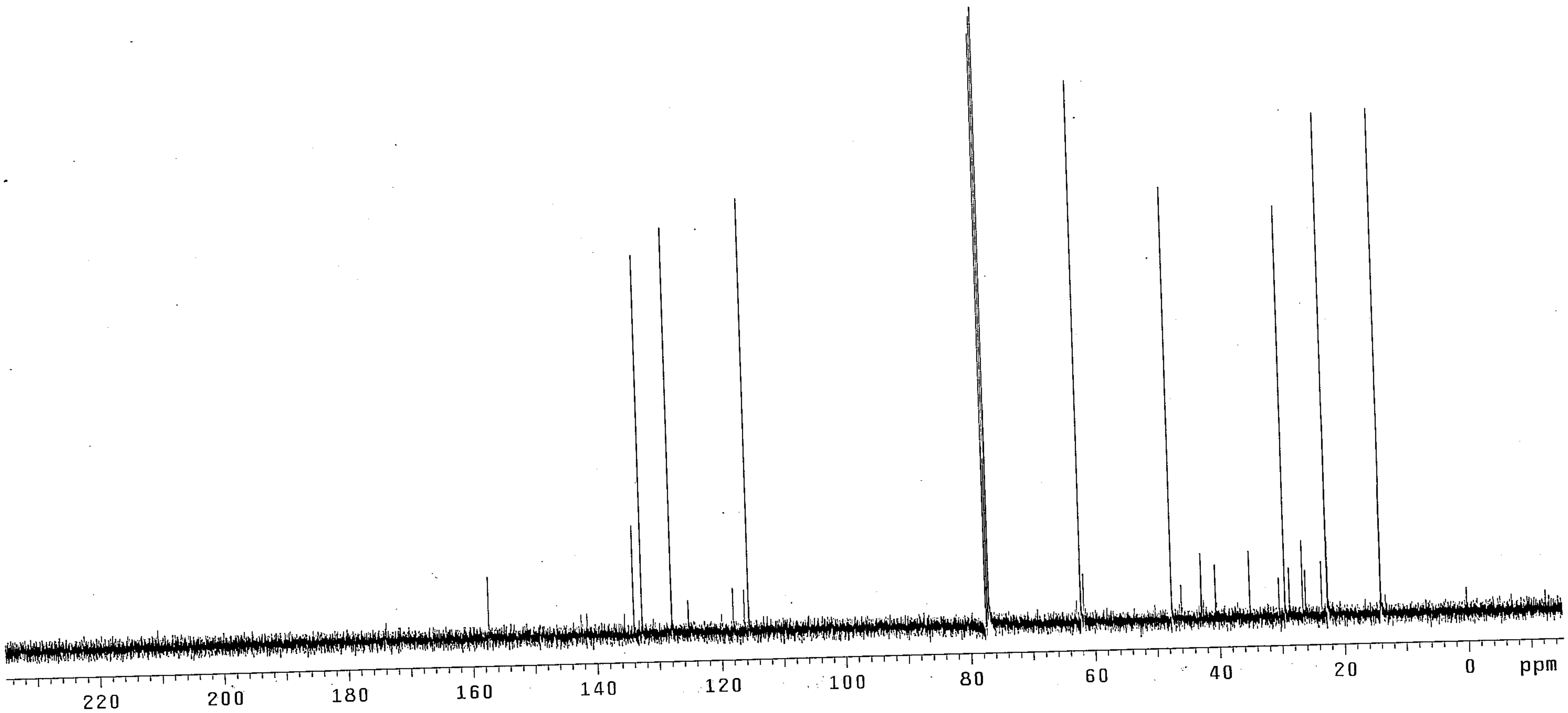

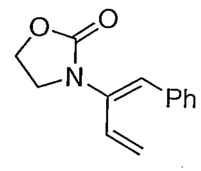

4

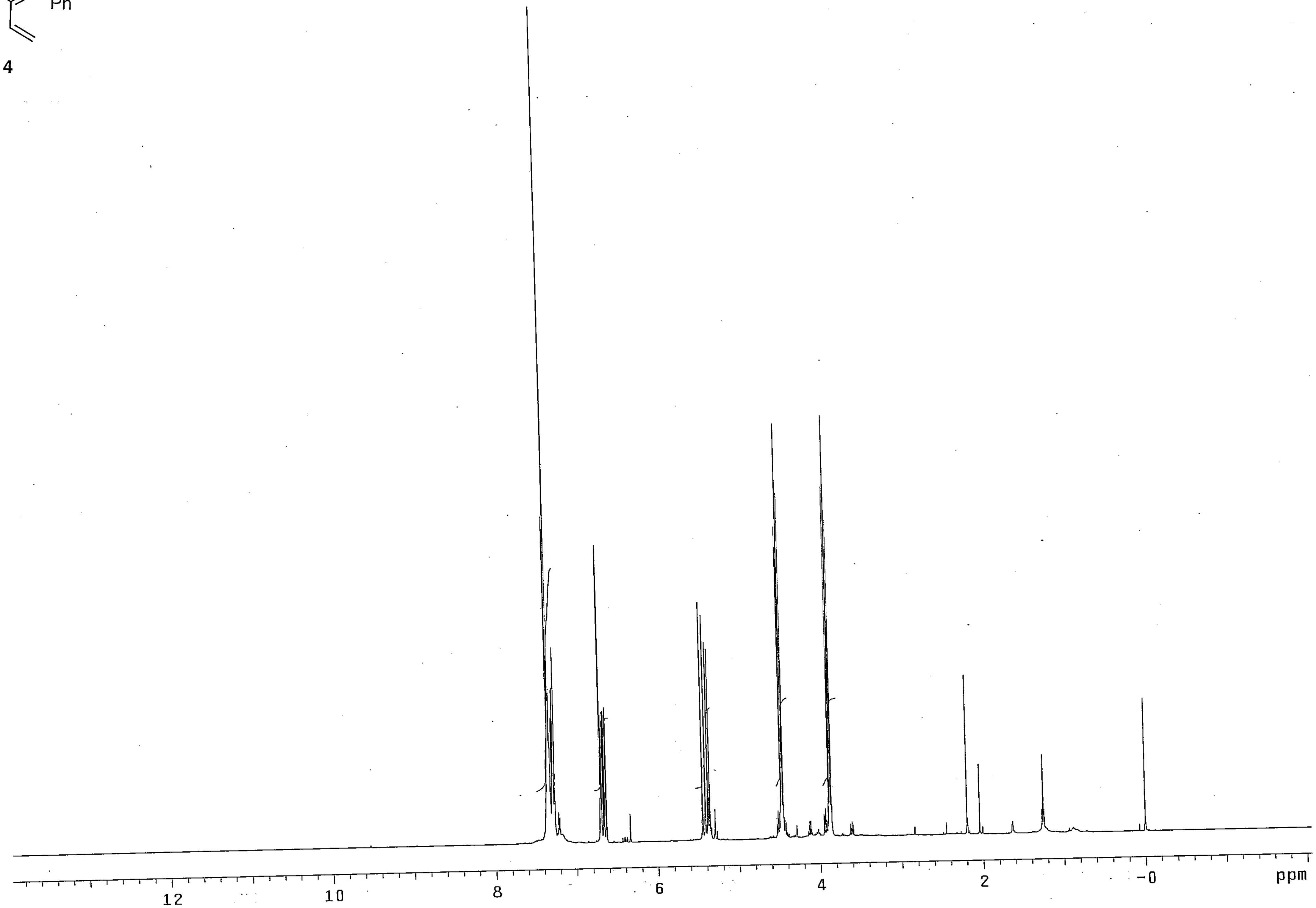

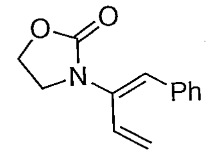

4

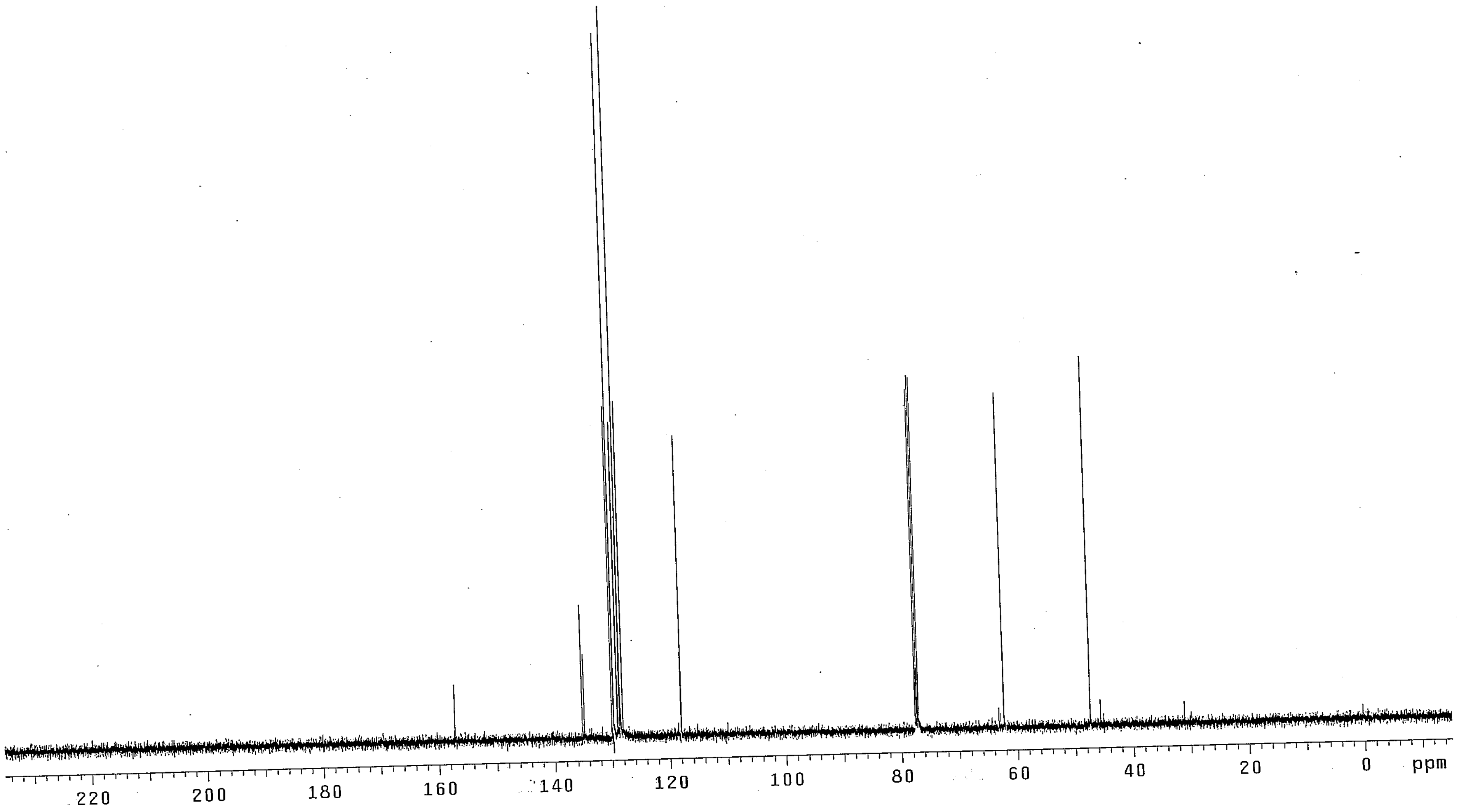

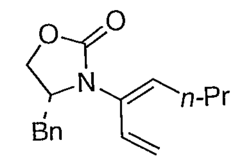

8a

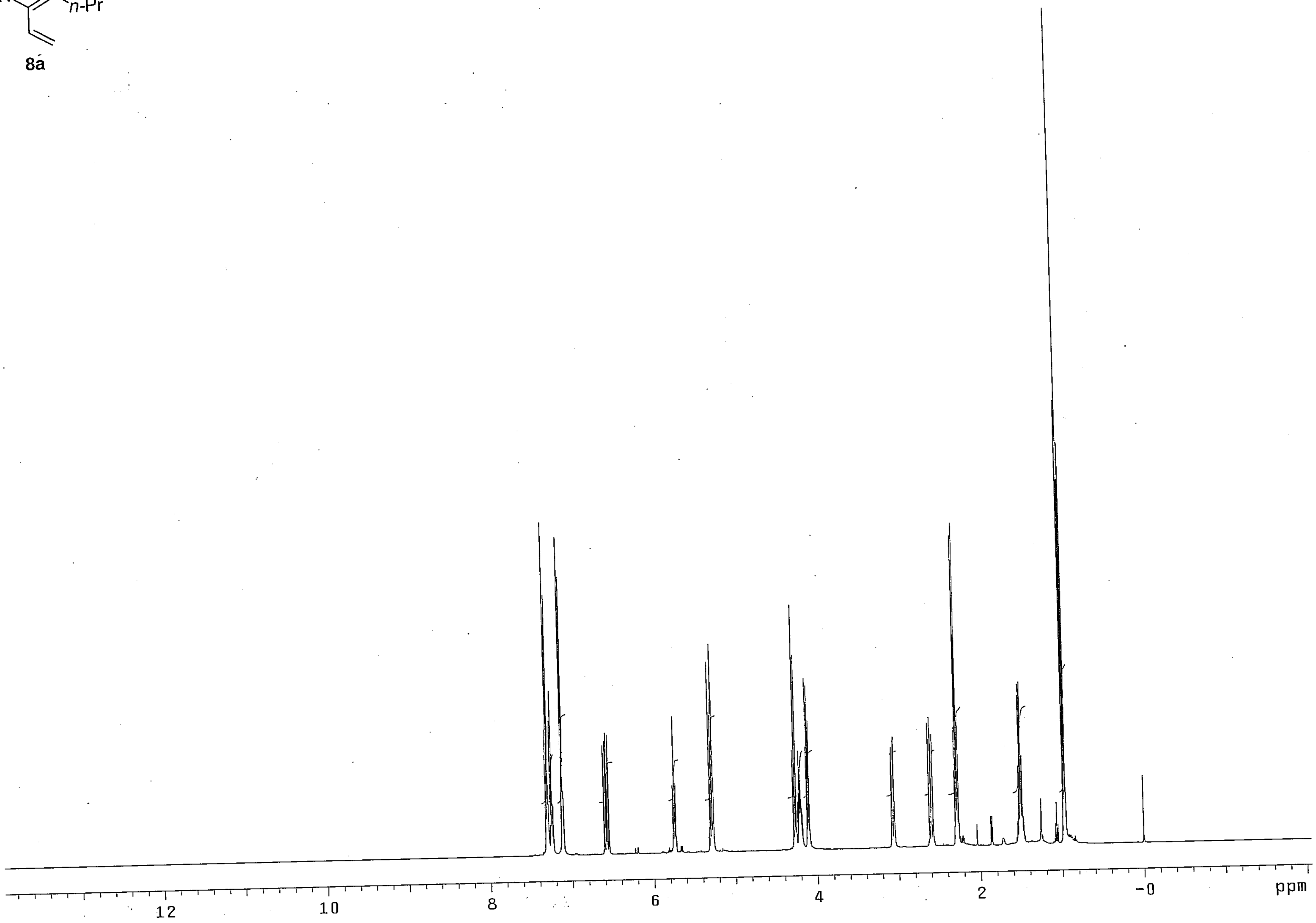

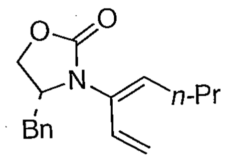

8a

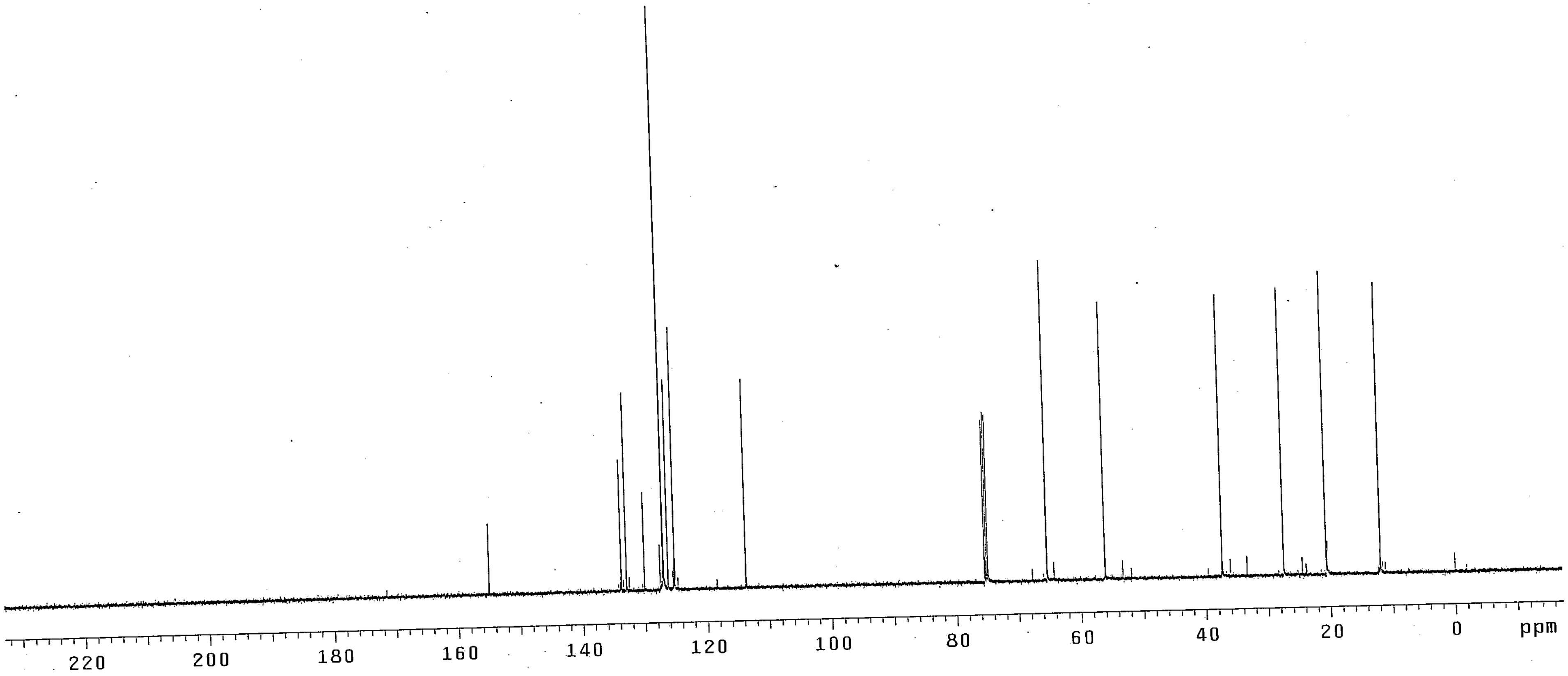

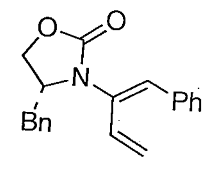

8b

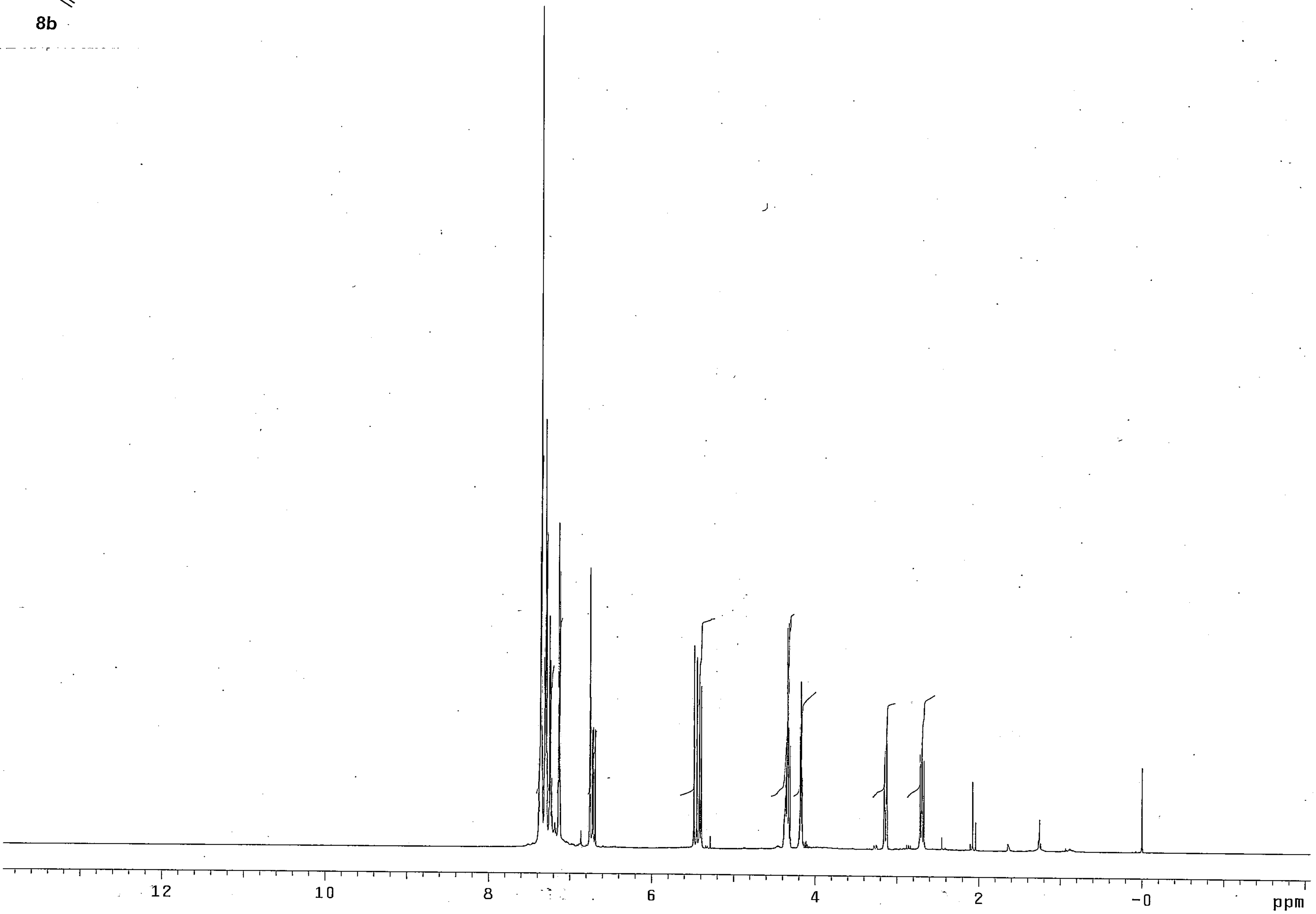

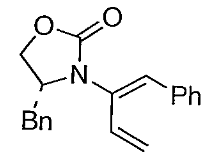

8b

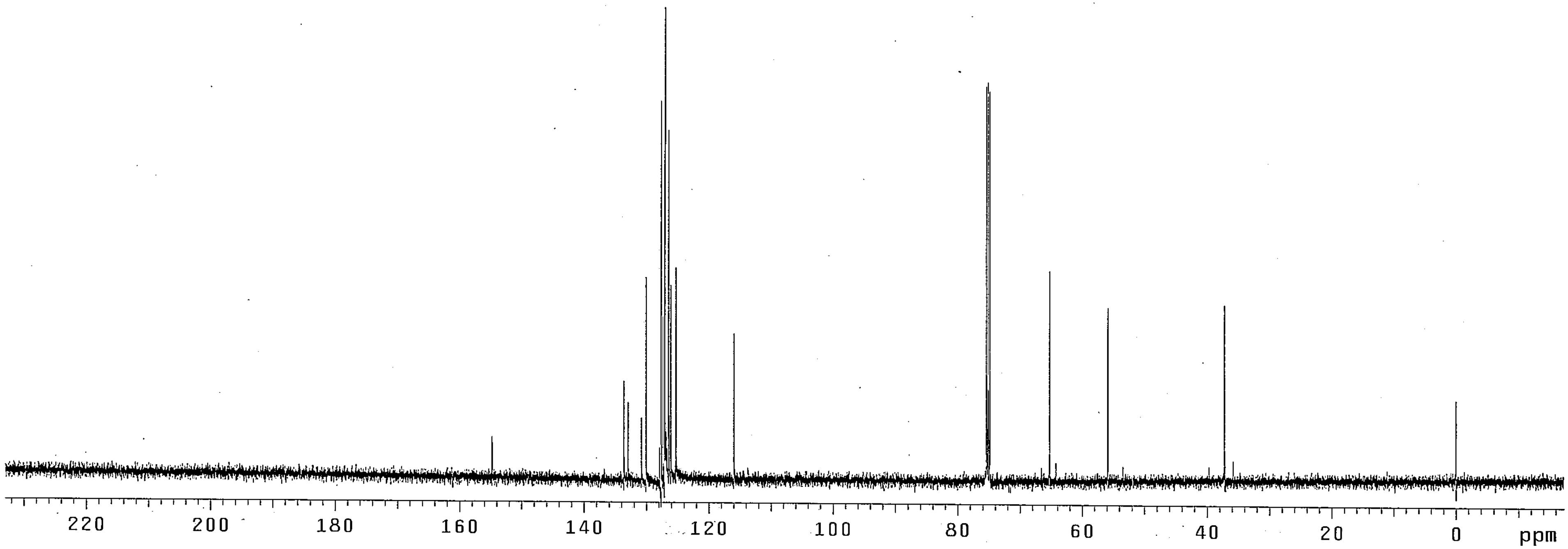

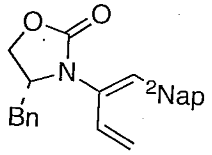

8c

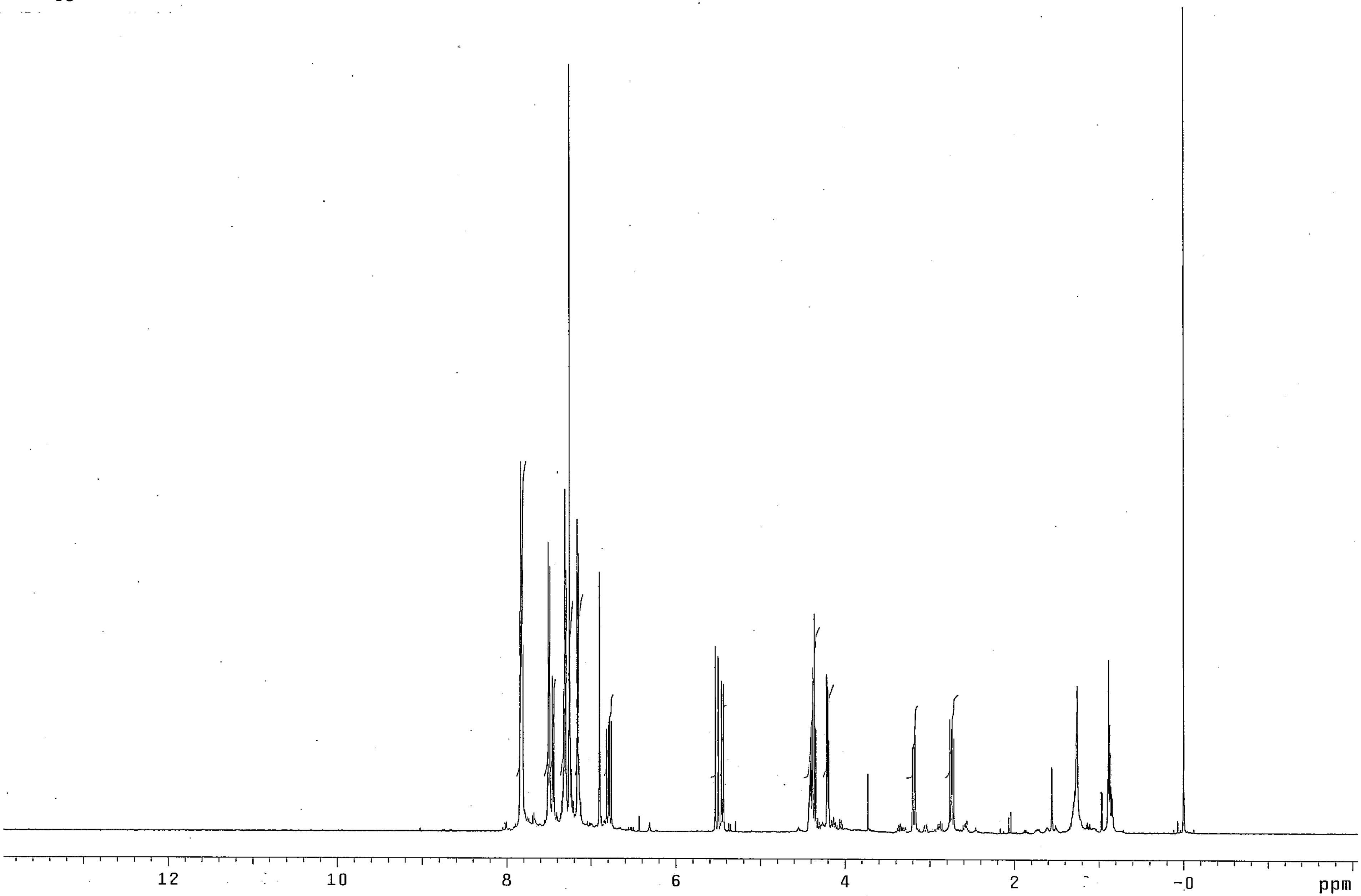

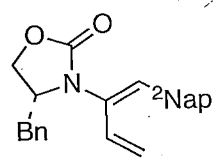

8c

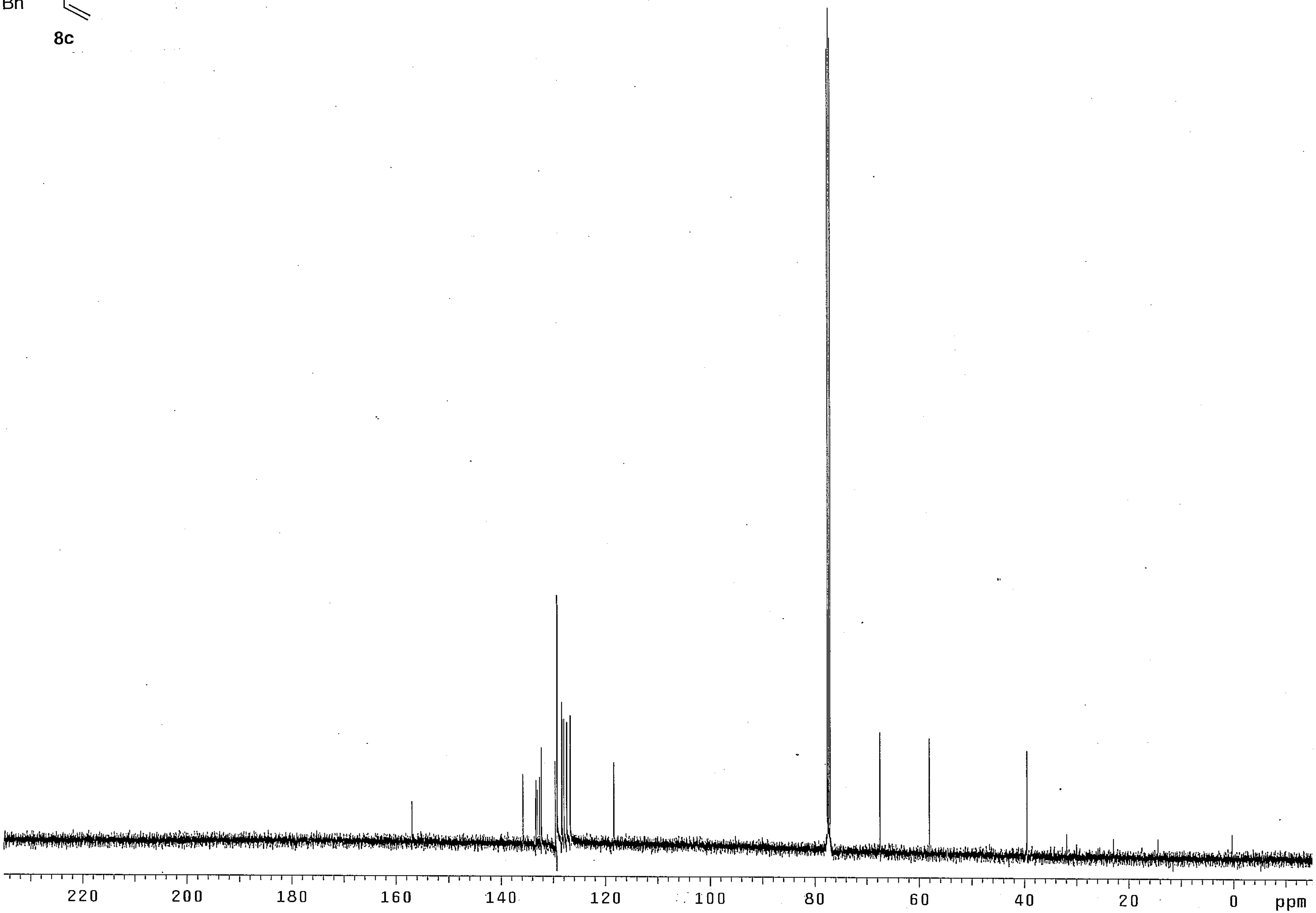

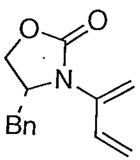

8d

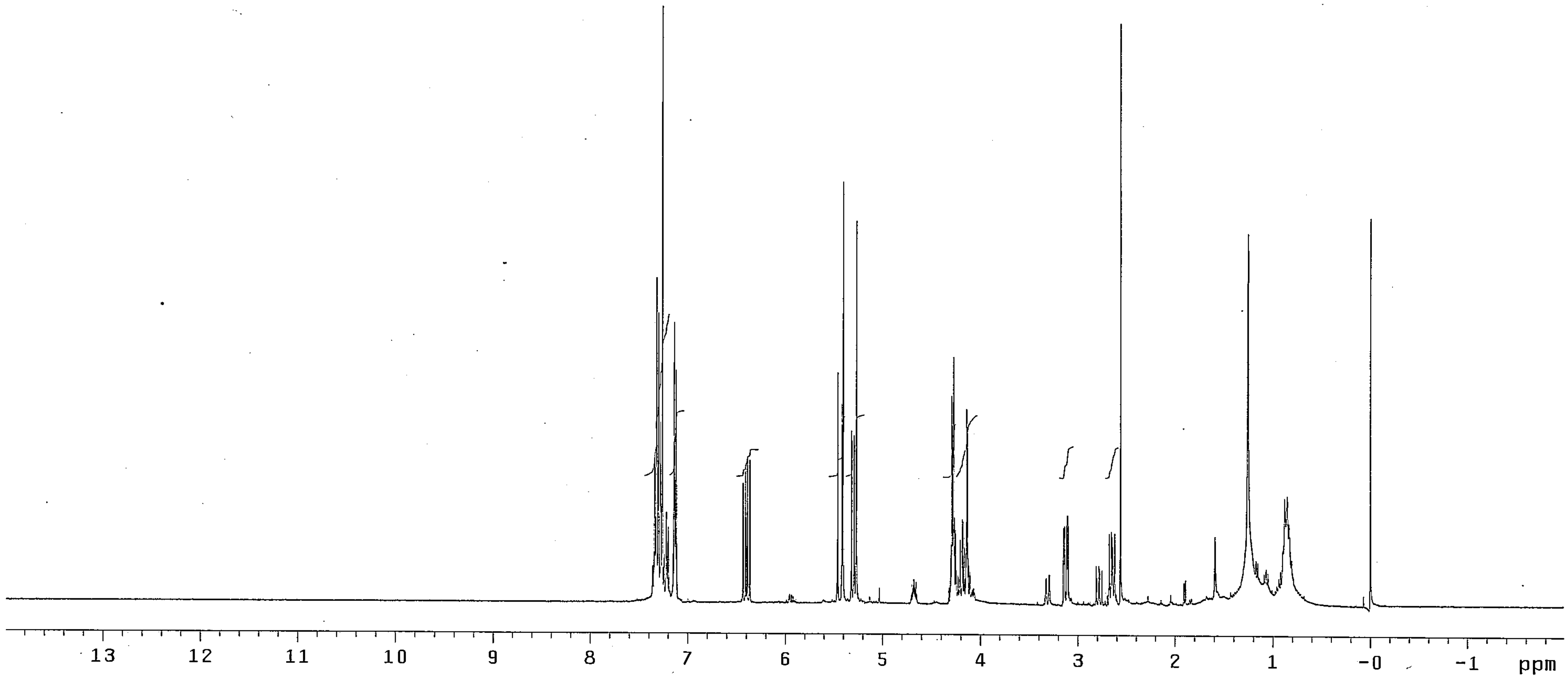

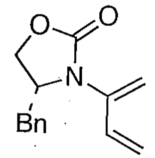

8d

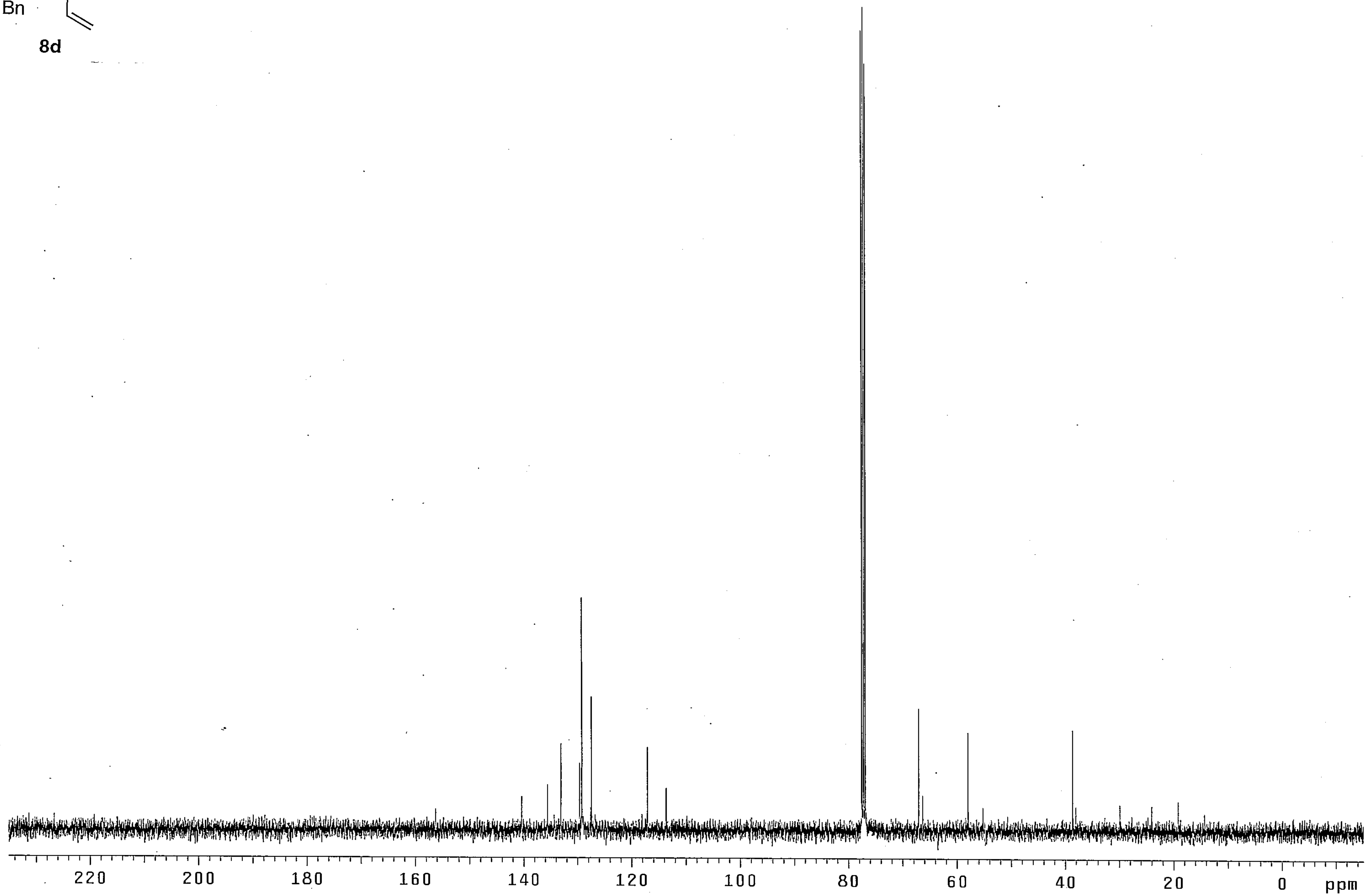

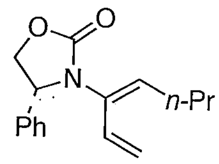

9a

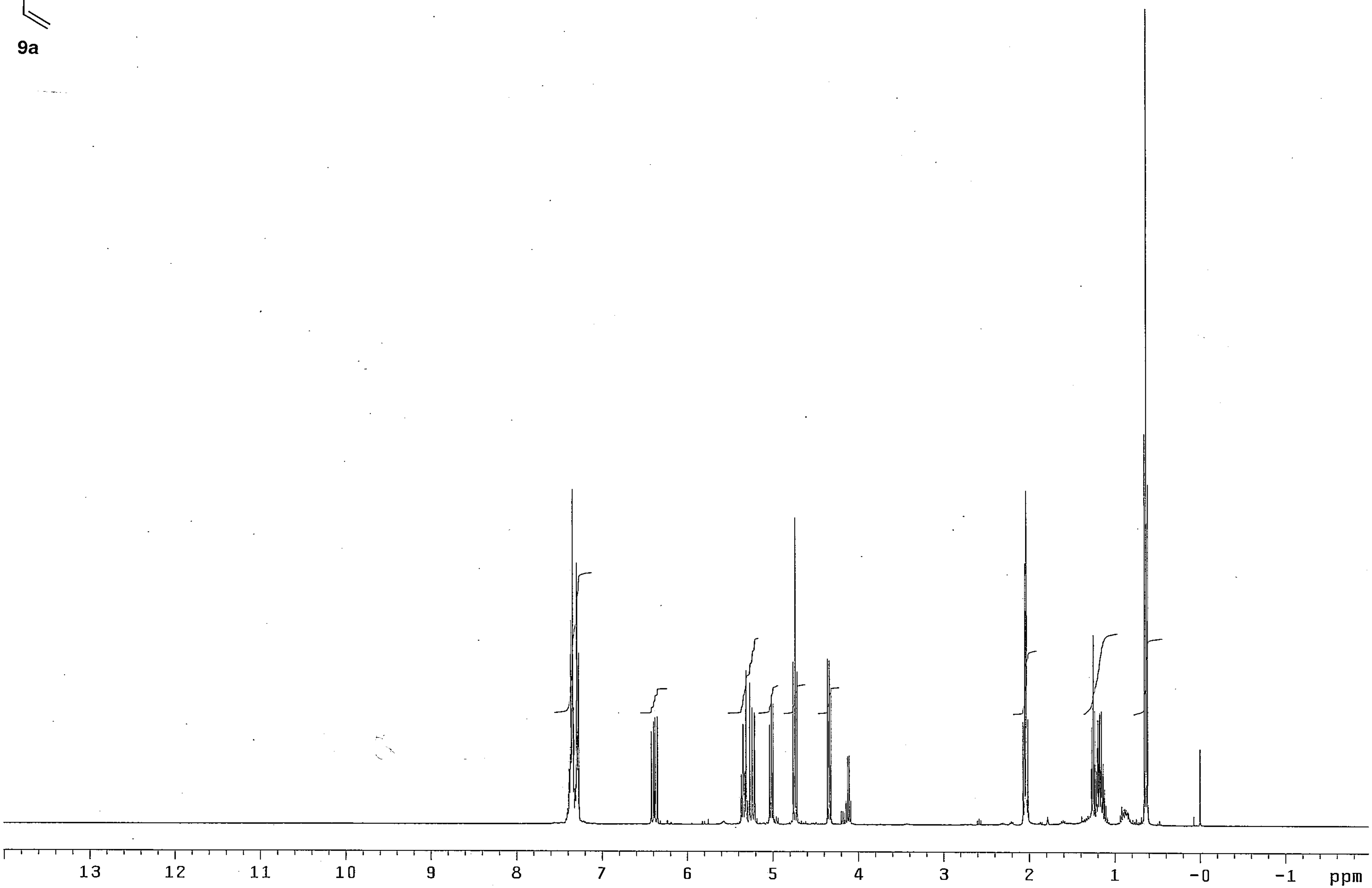

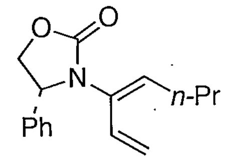

9a

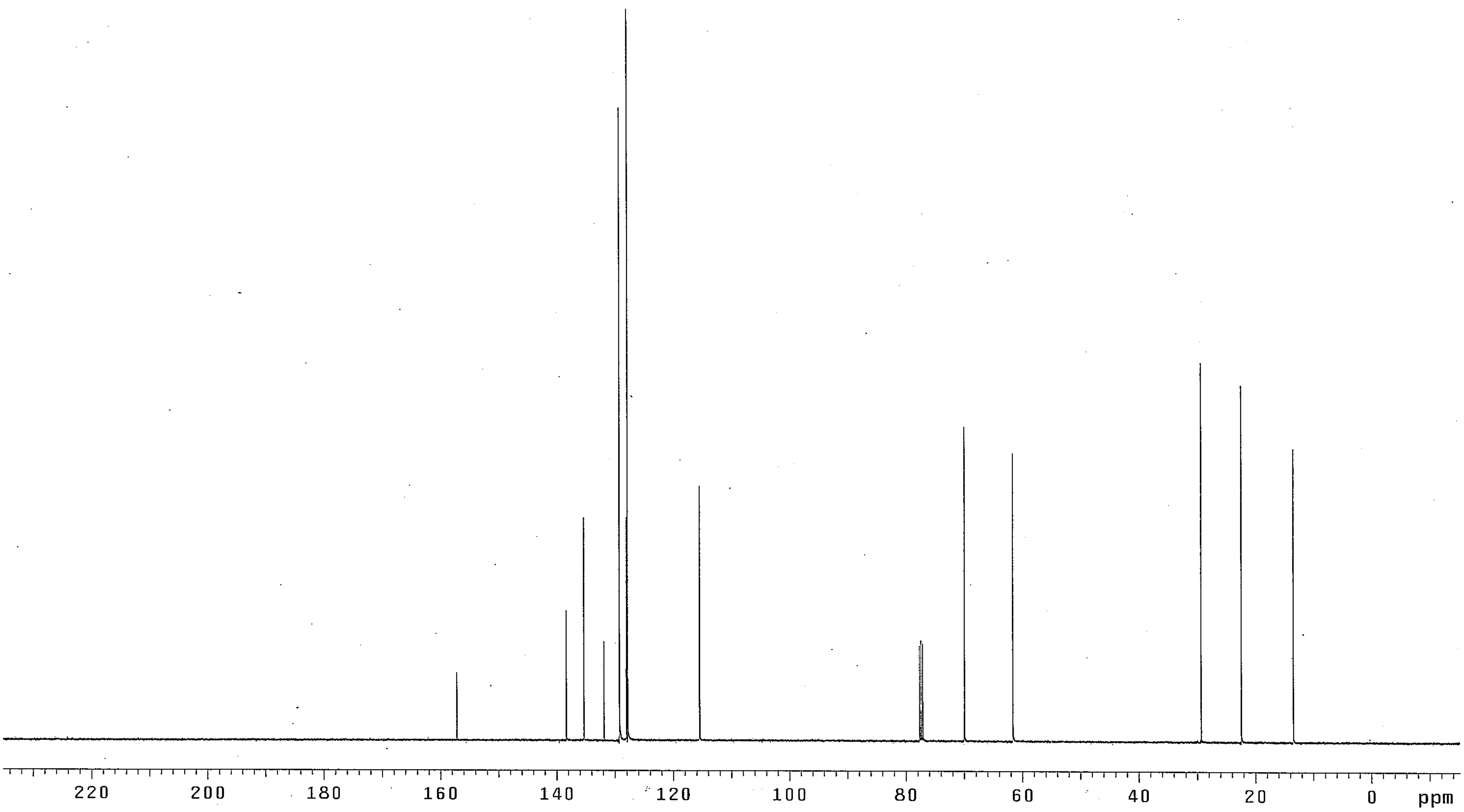

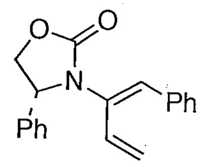

9b

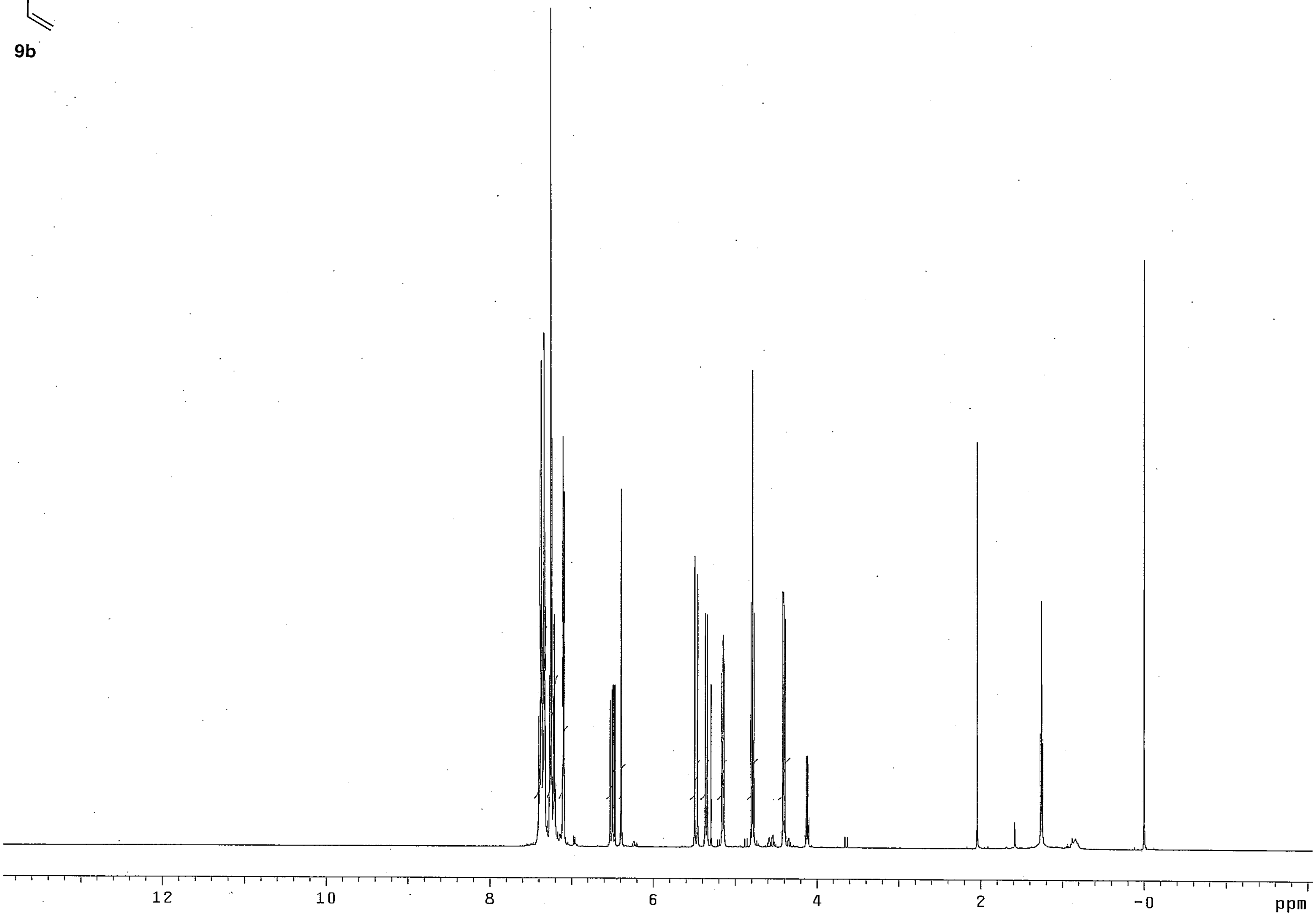

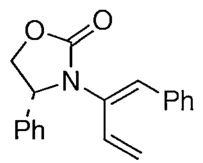

9b

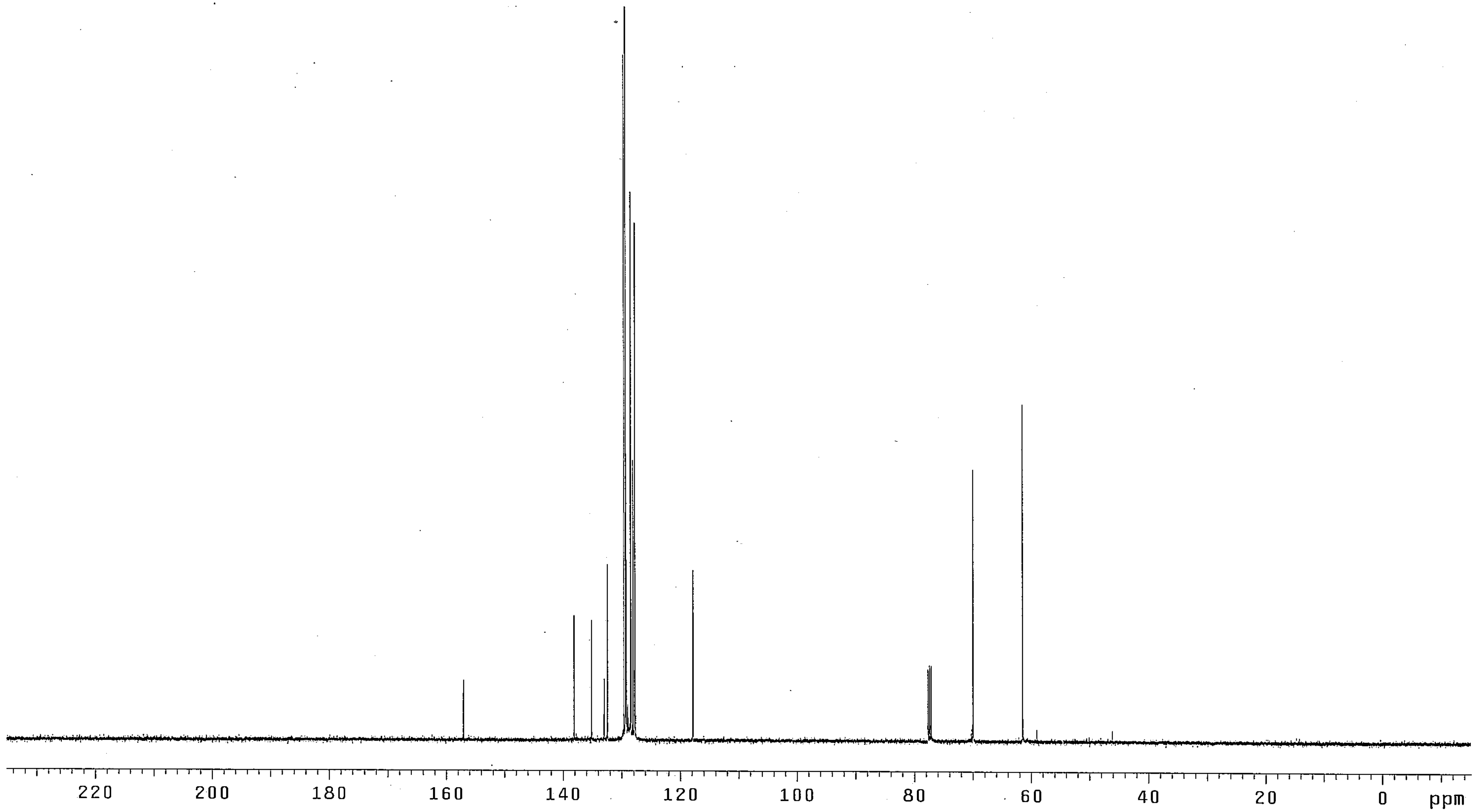

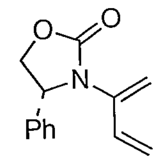

9c

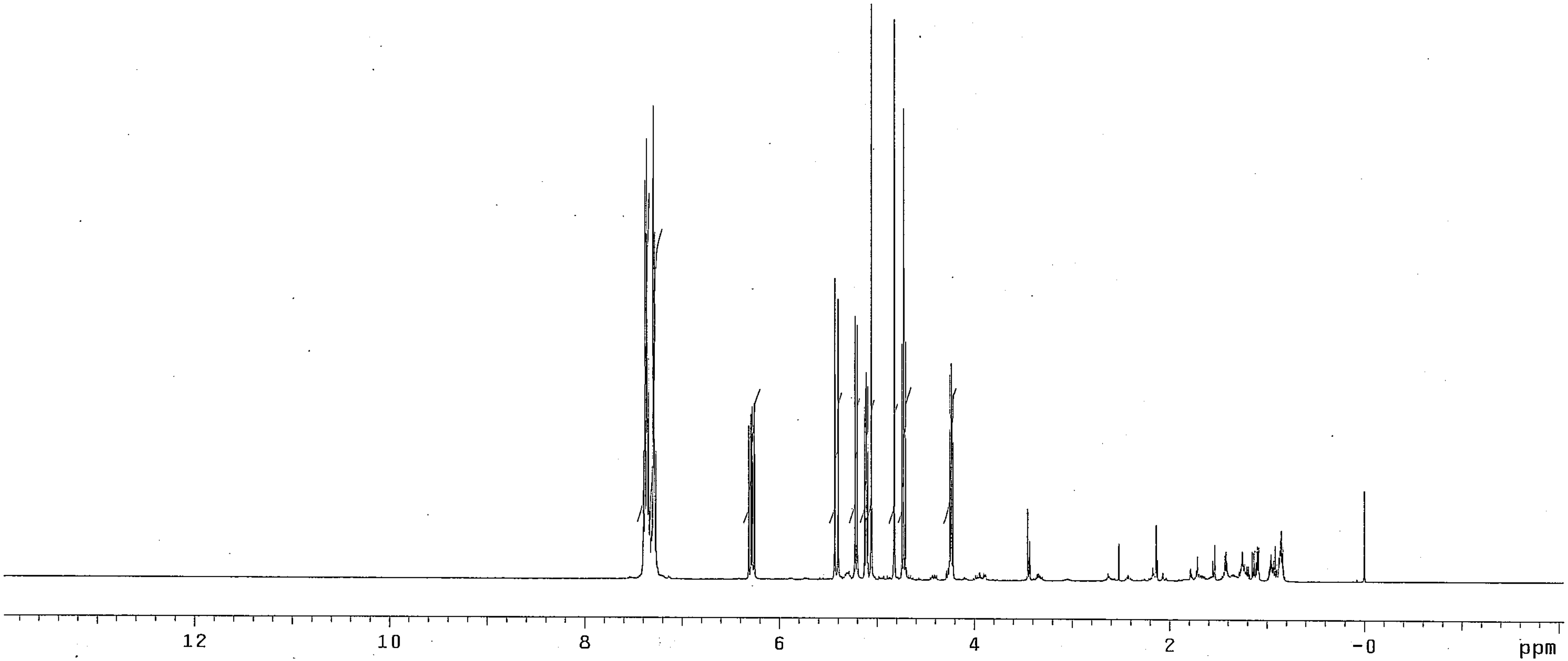

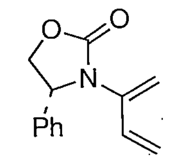

9c

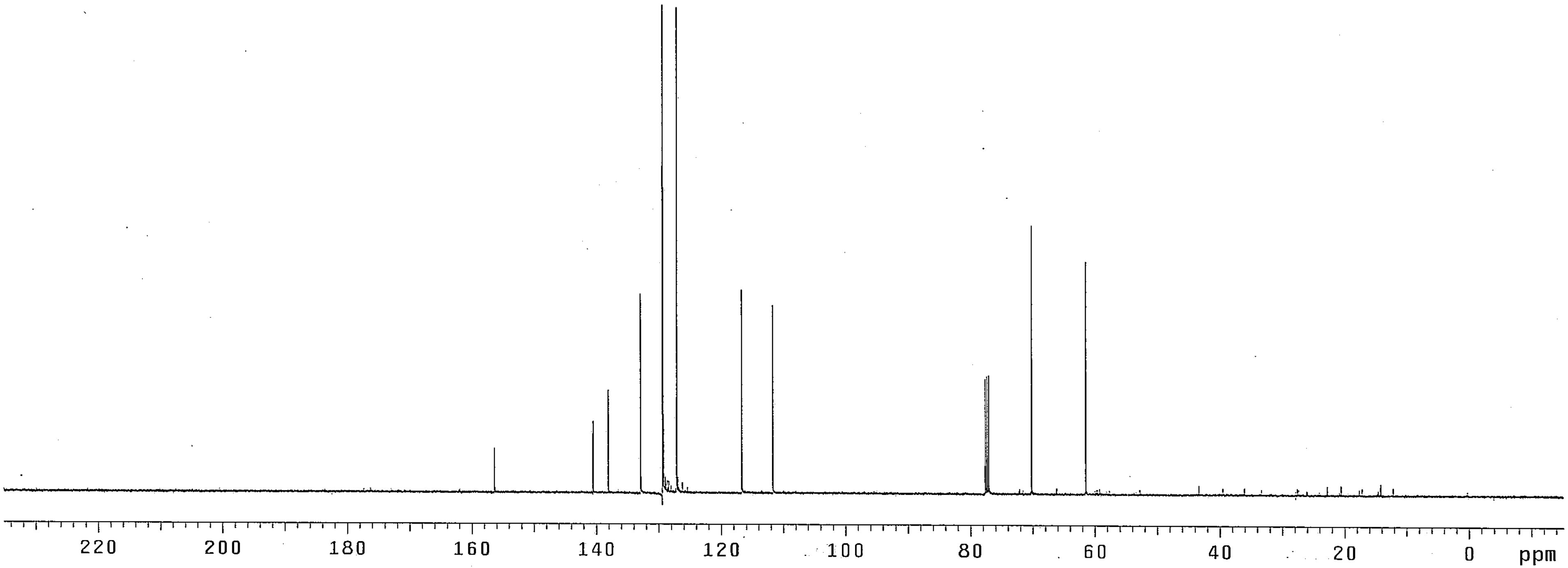

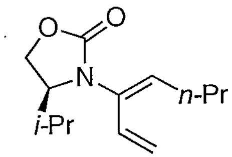

10a

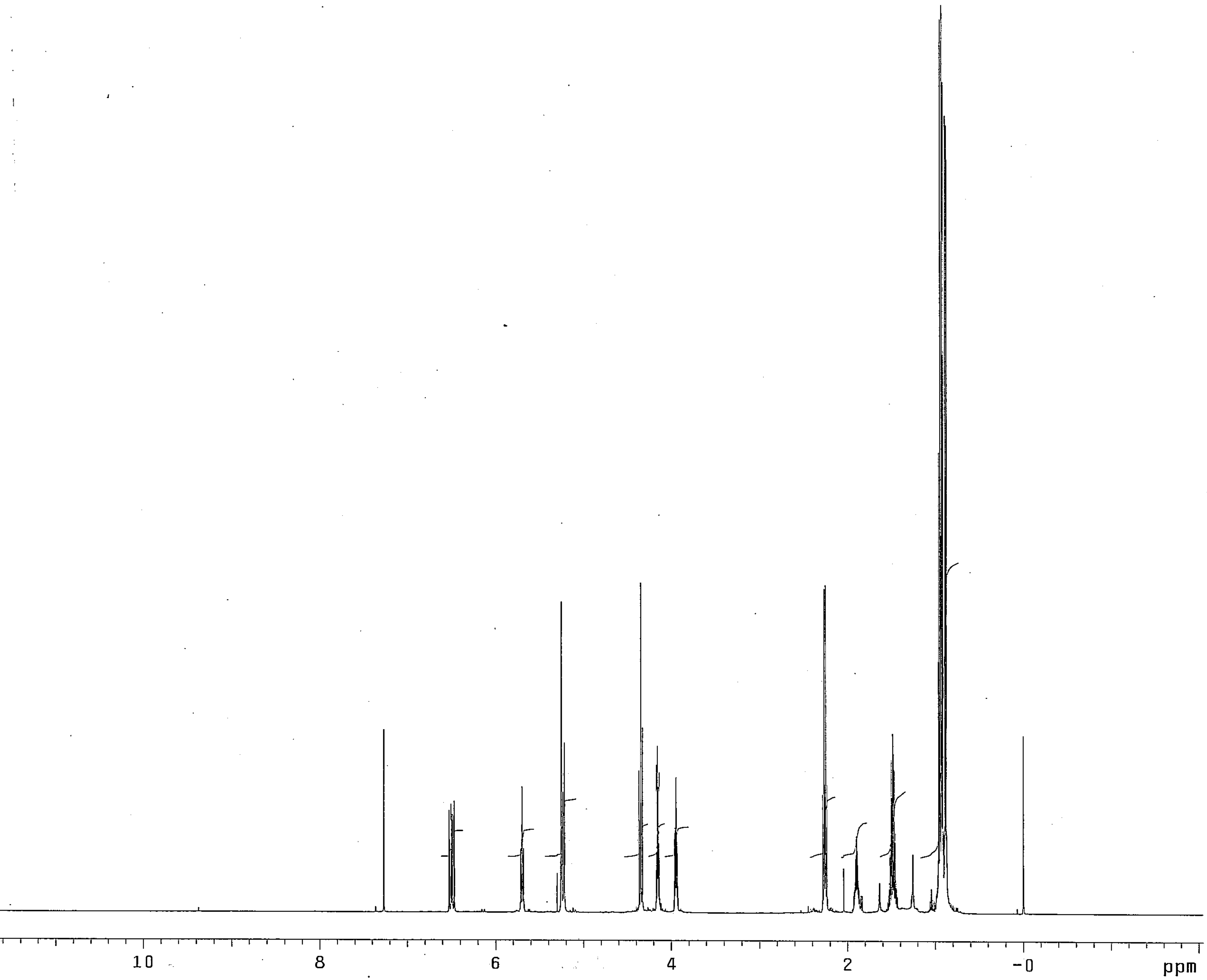

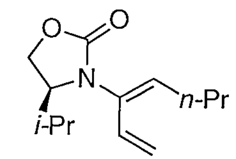

10a

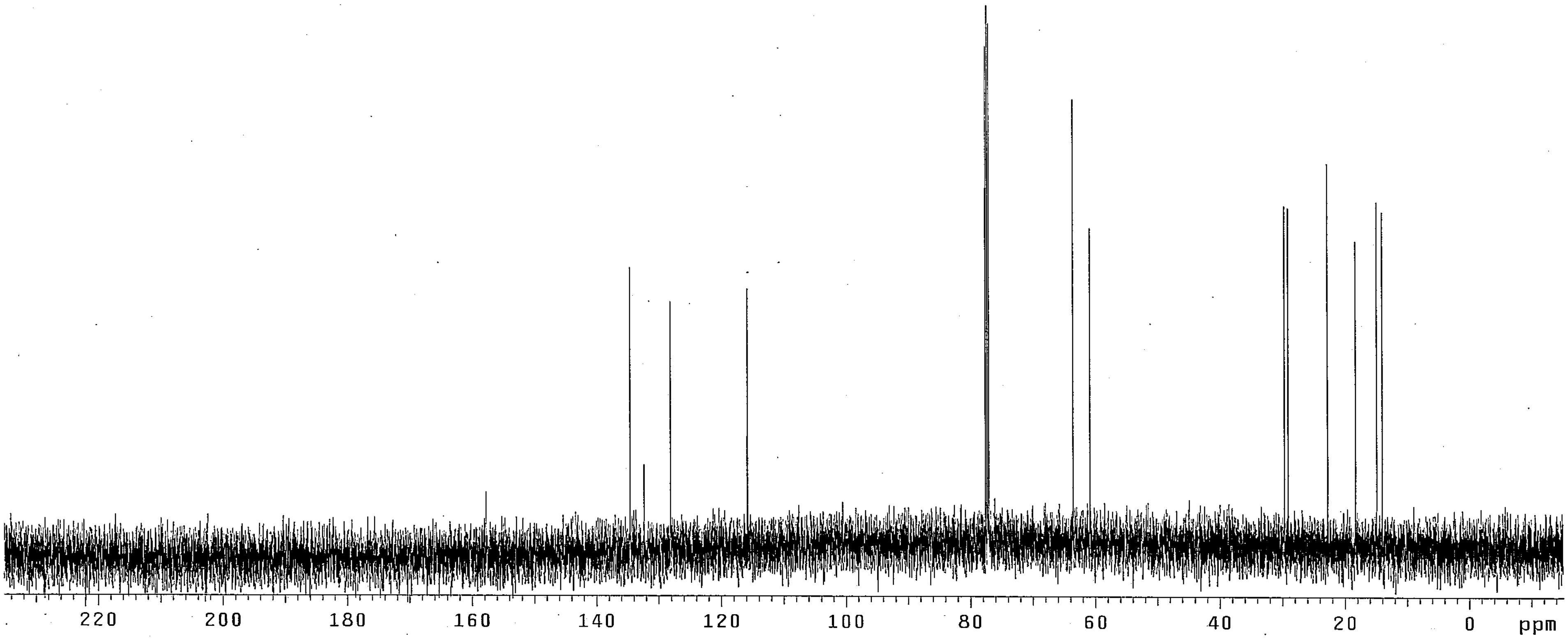

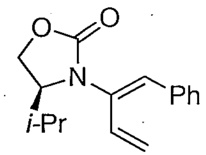

10b

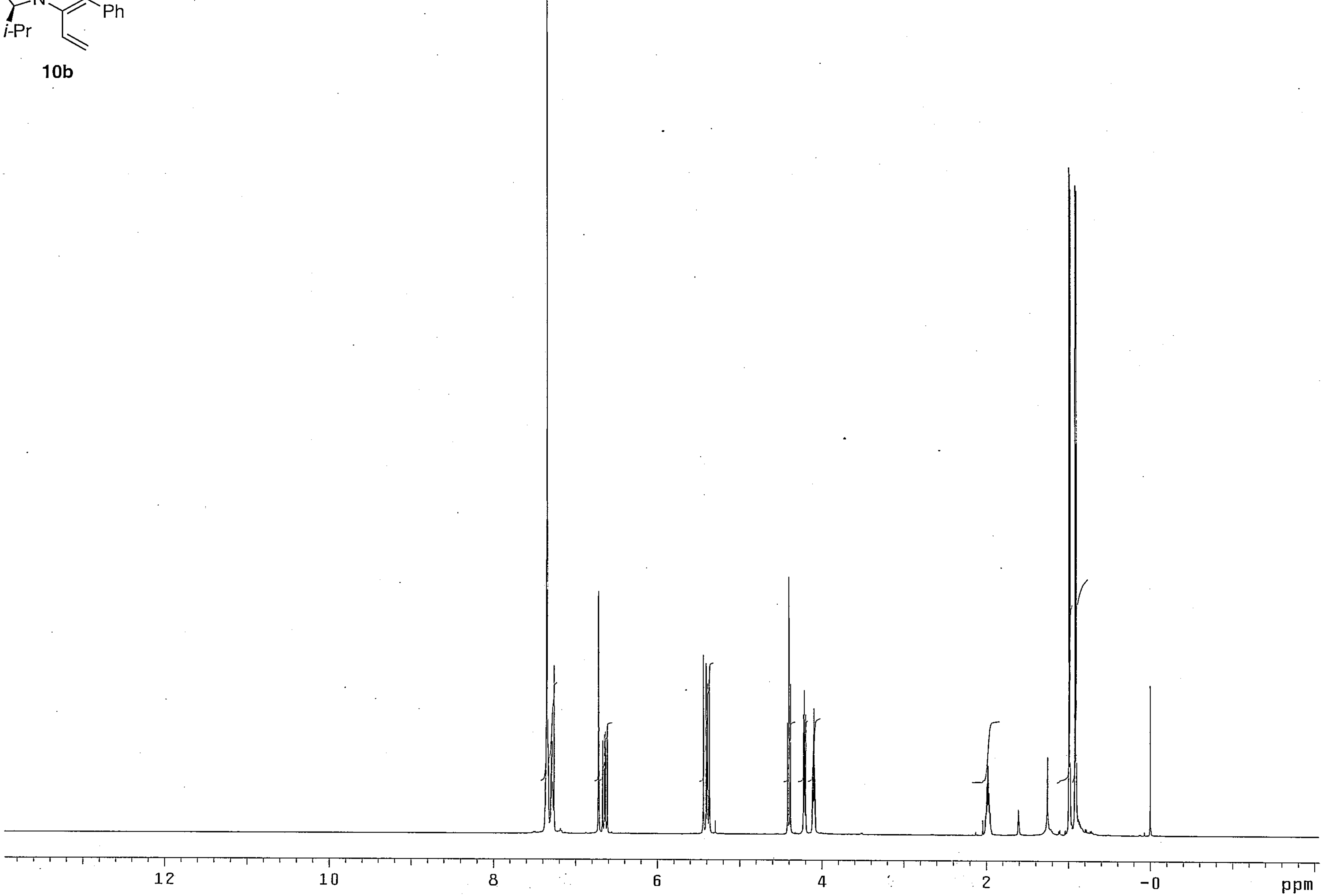

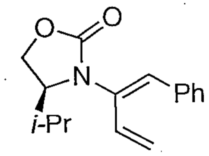

10b

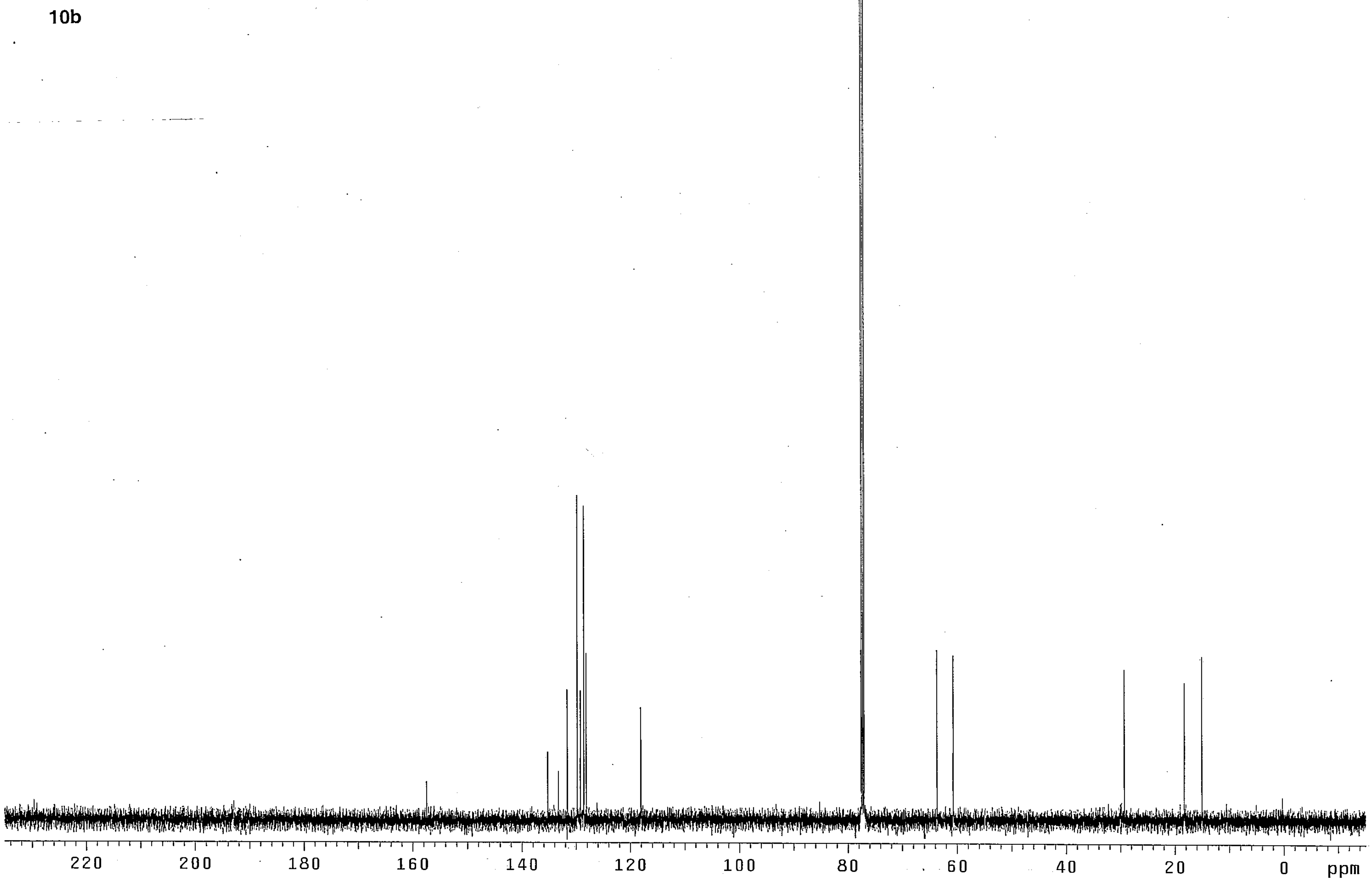

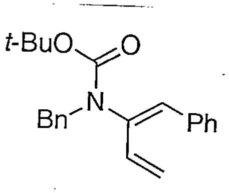

12

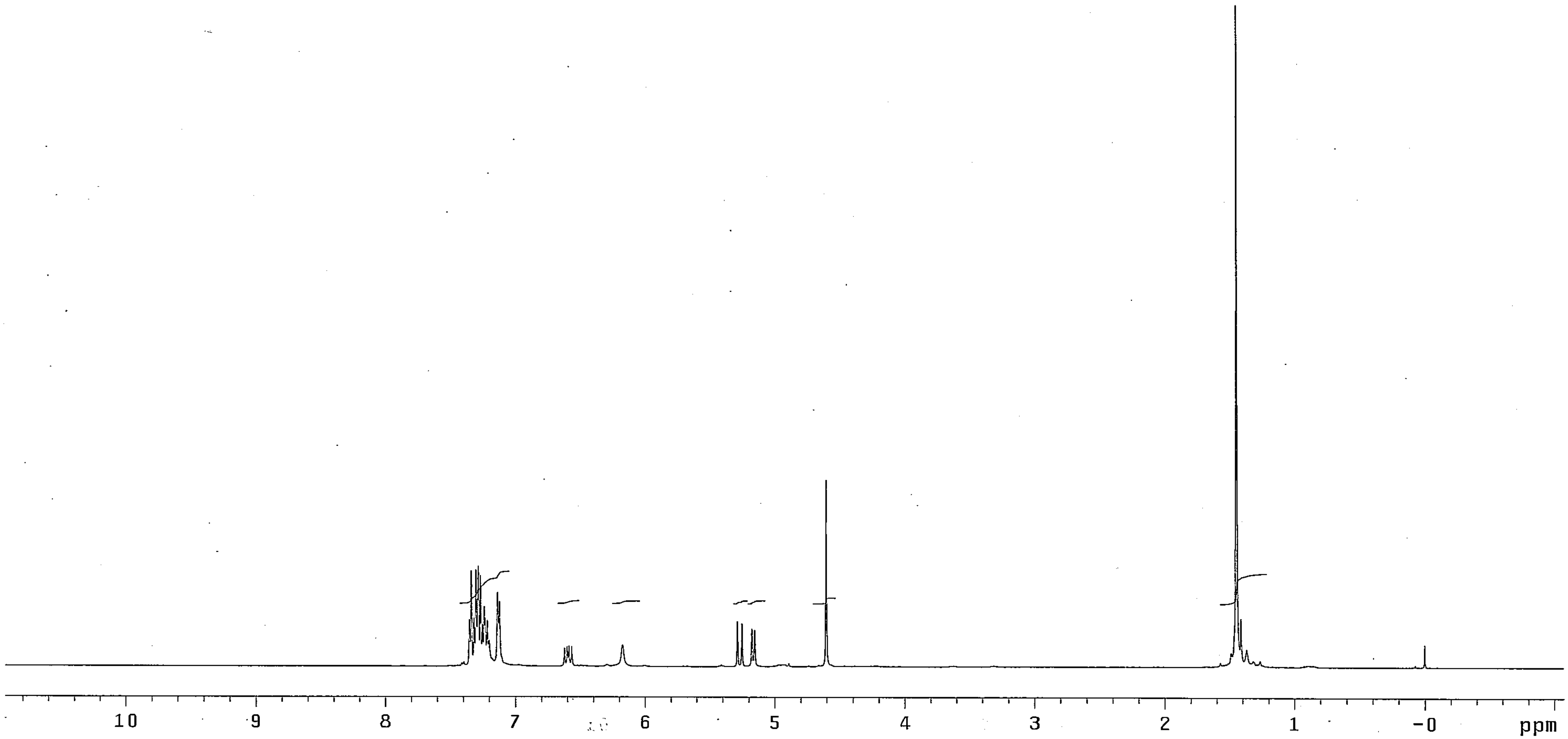

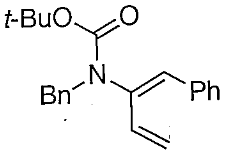

12

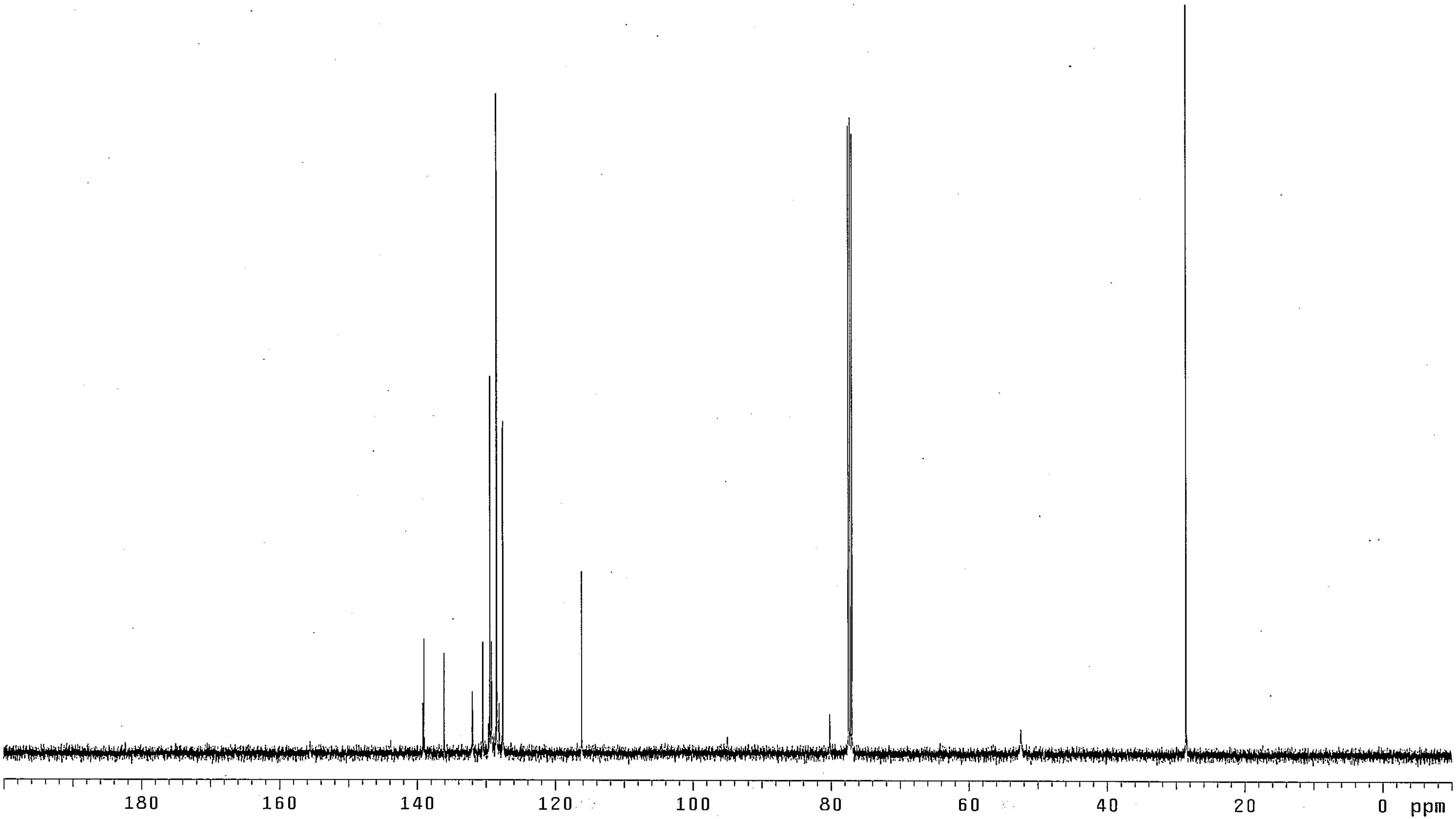

55

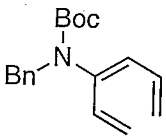

14

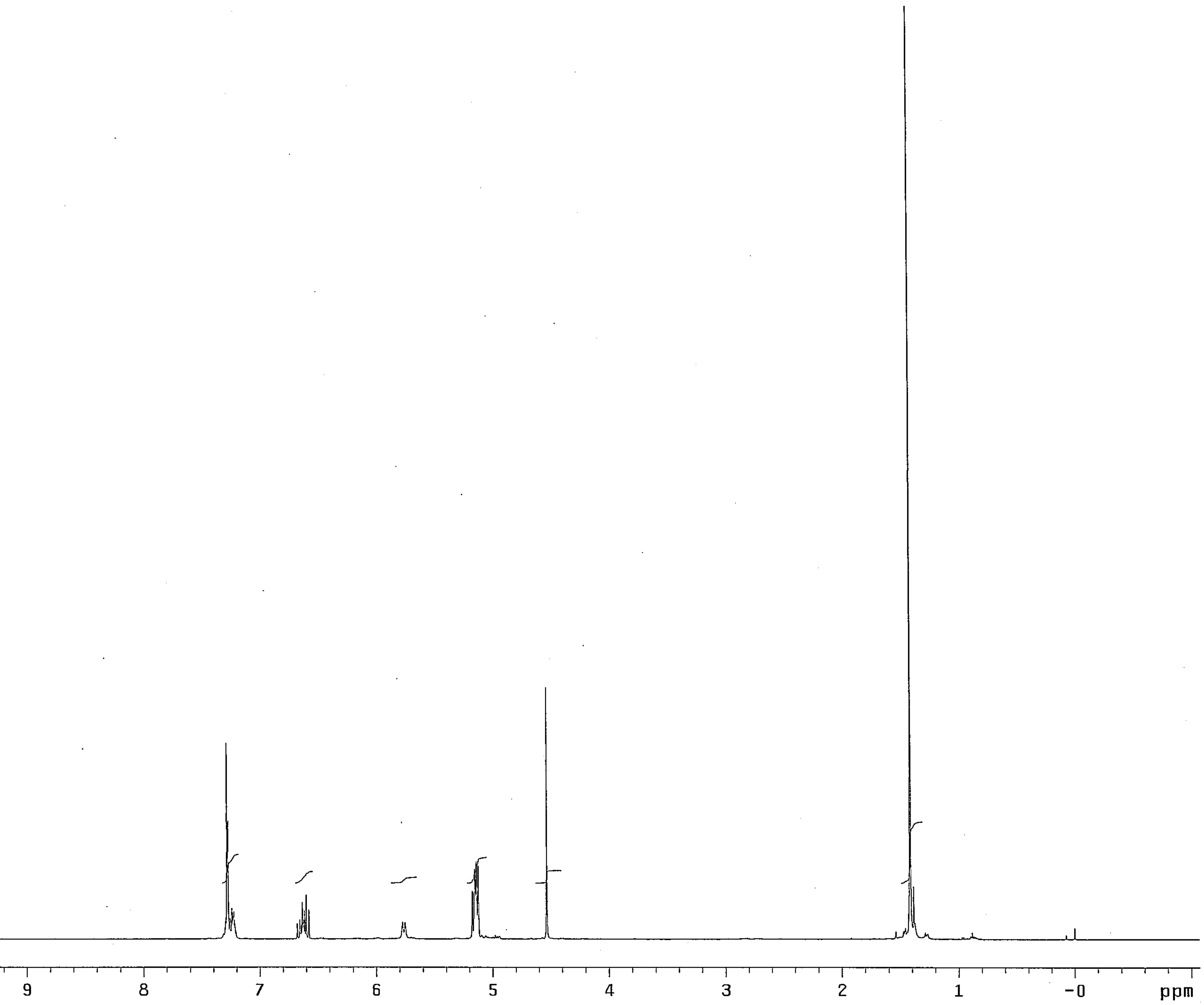

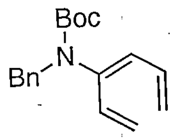

14

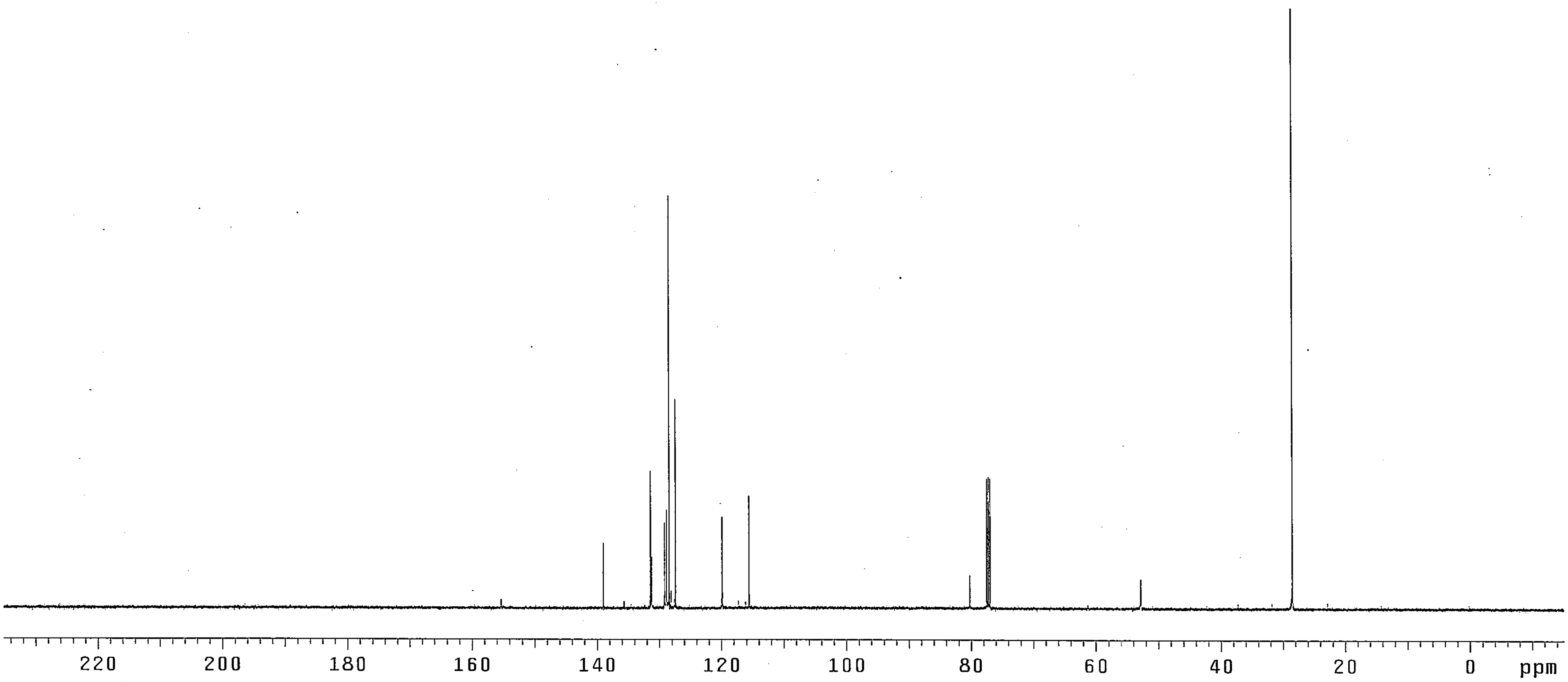

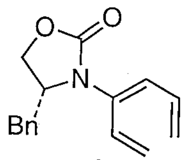

22

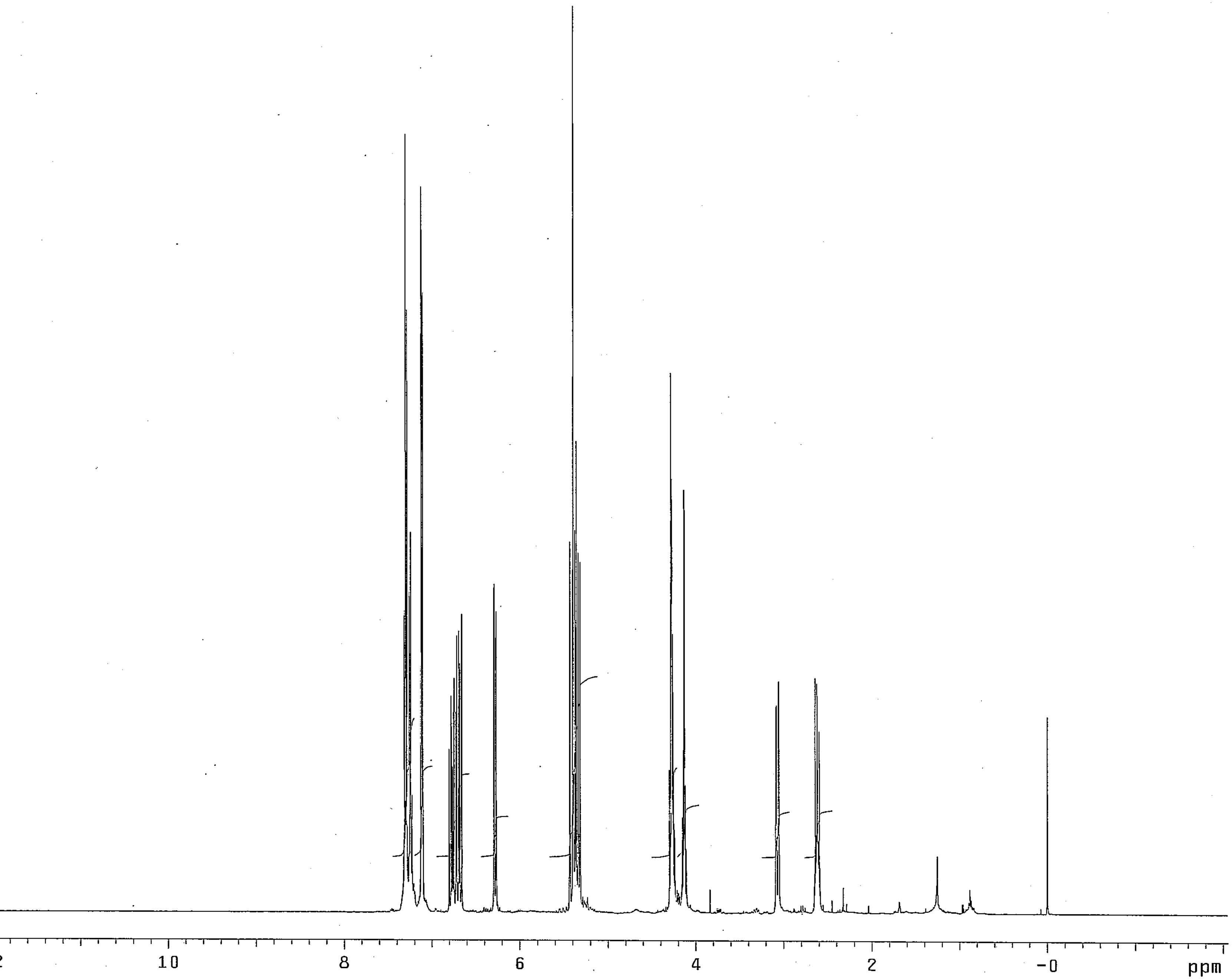

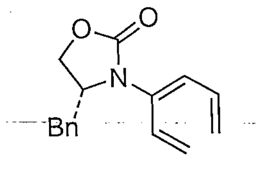

22

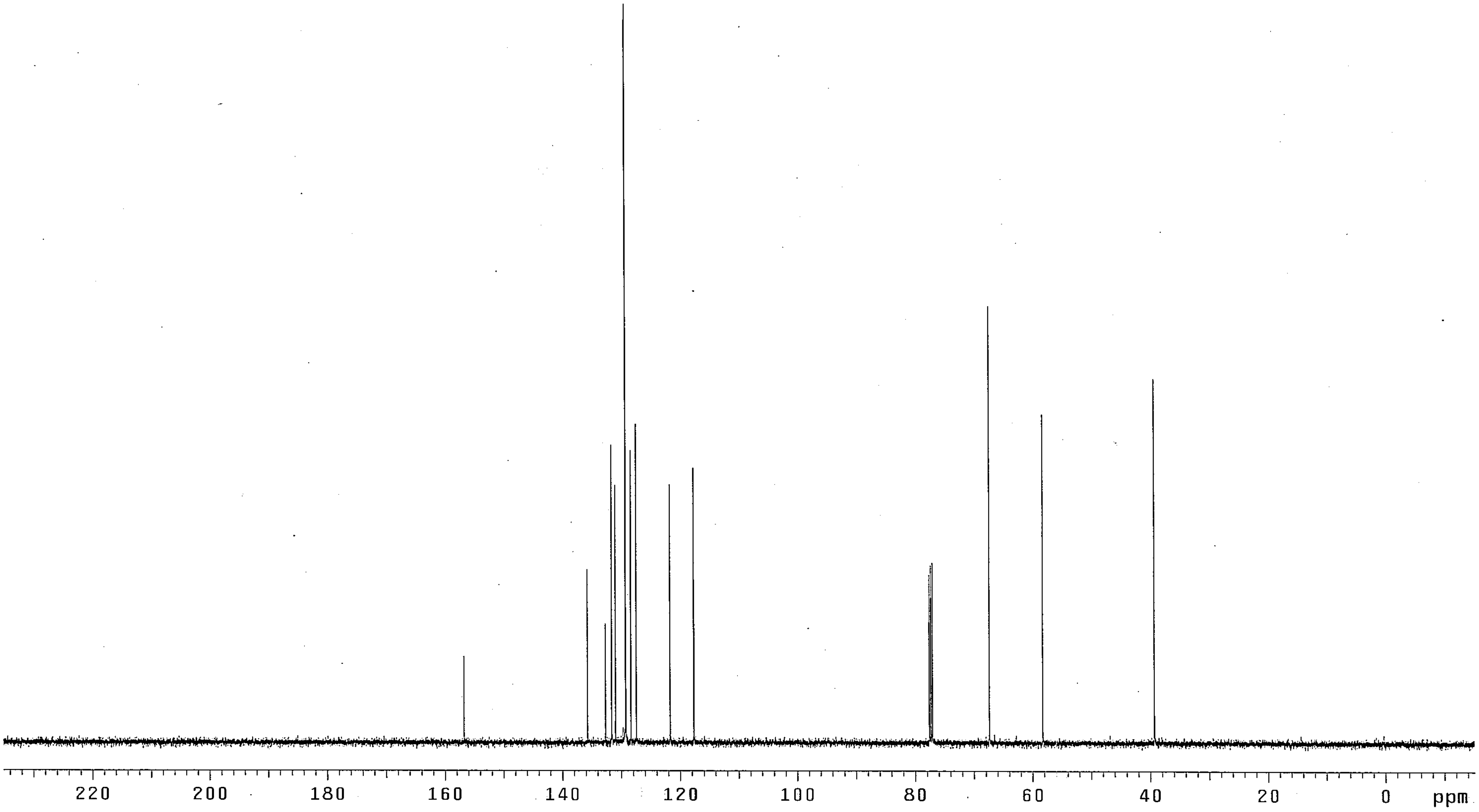

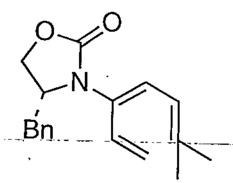

23a

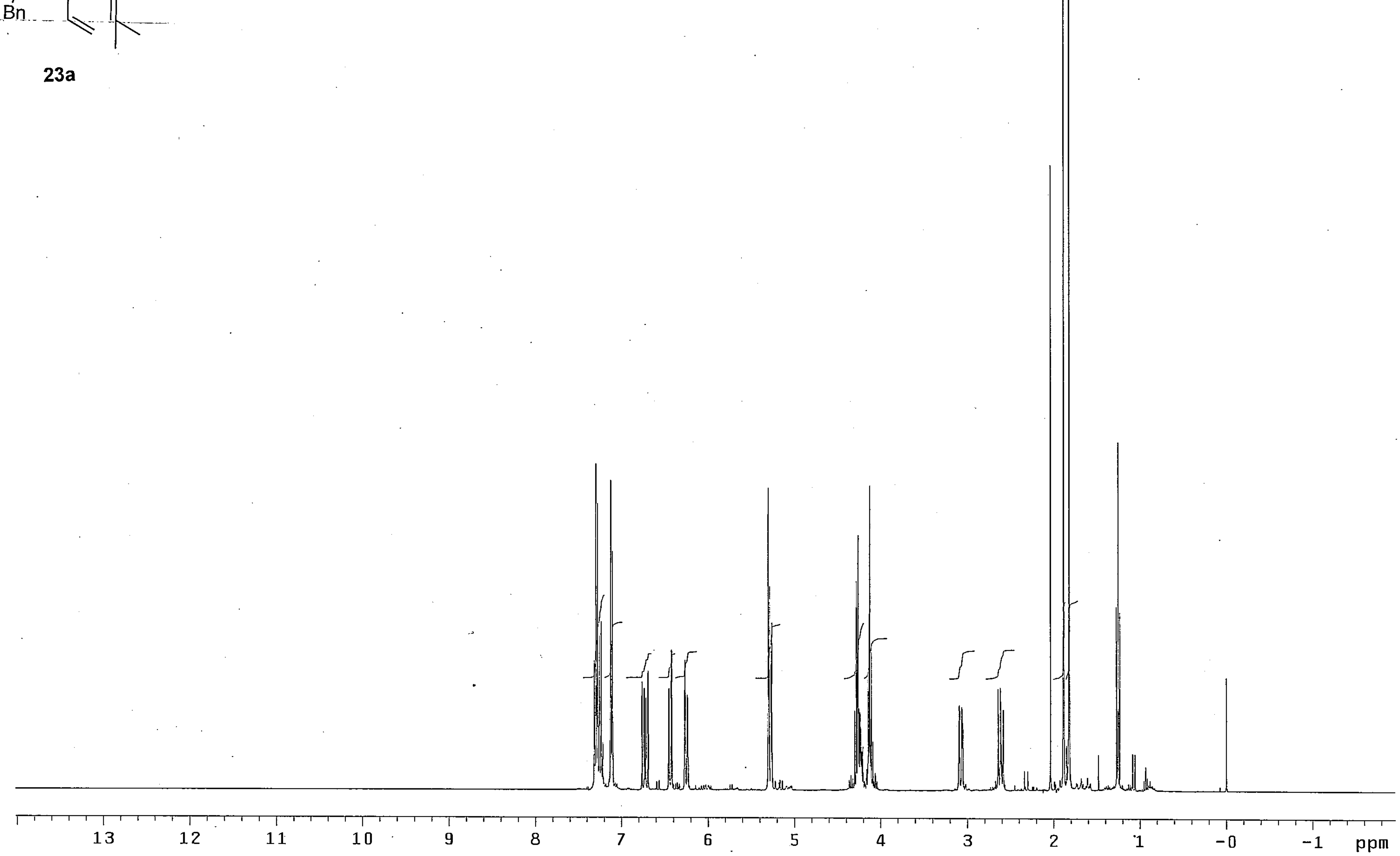

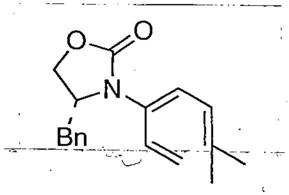

23a

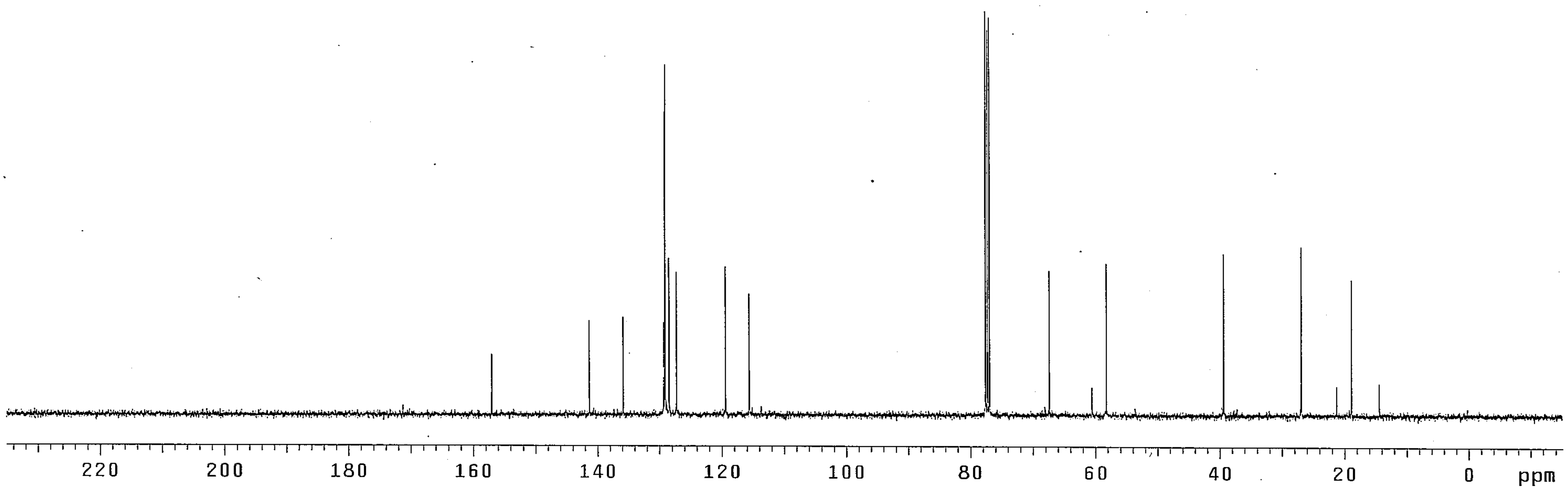

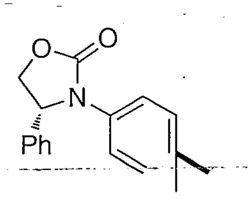

23b

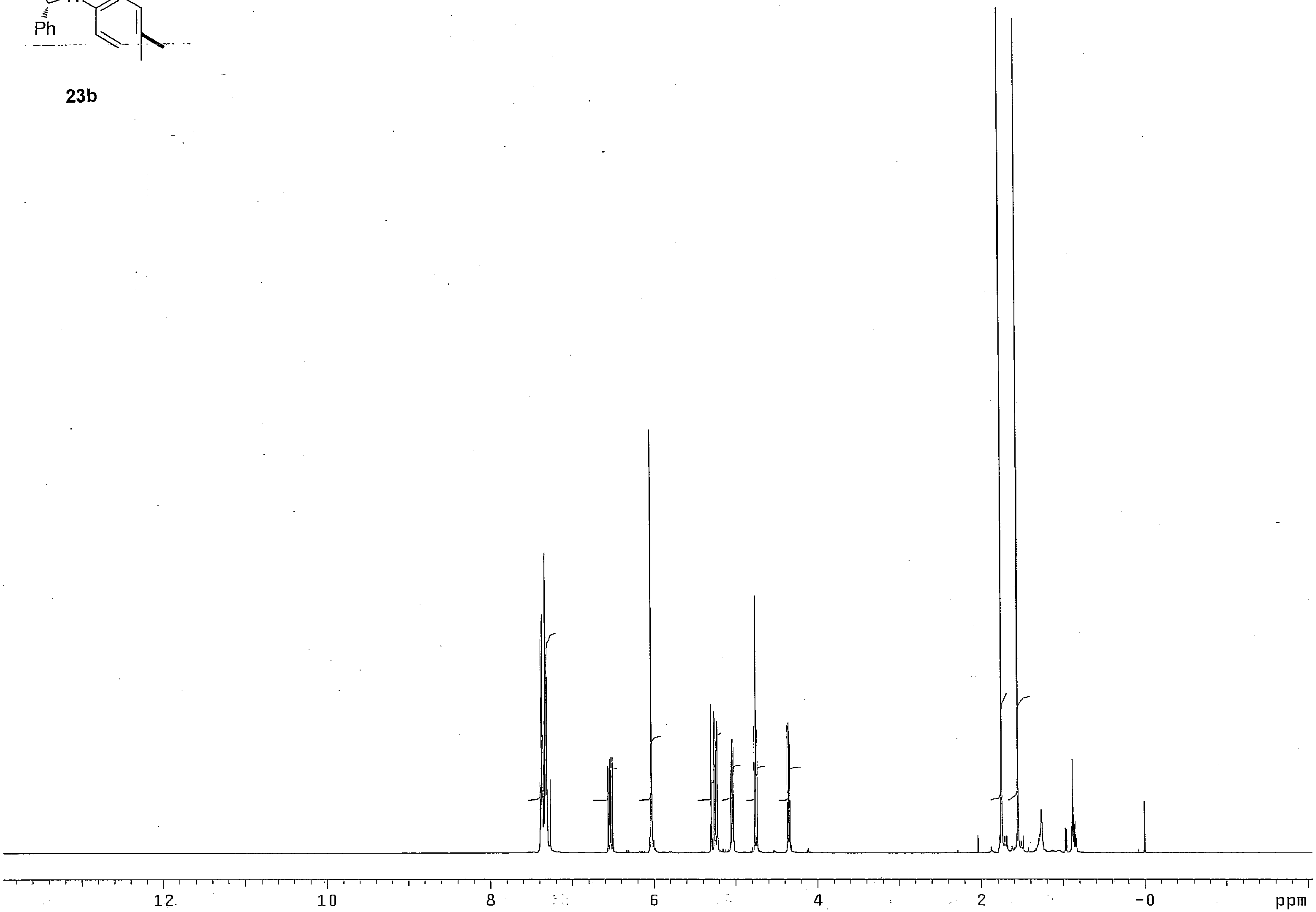

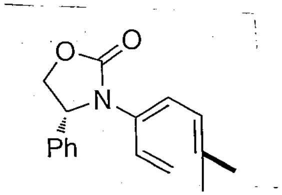

23b

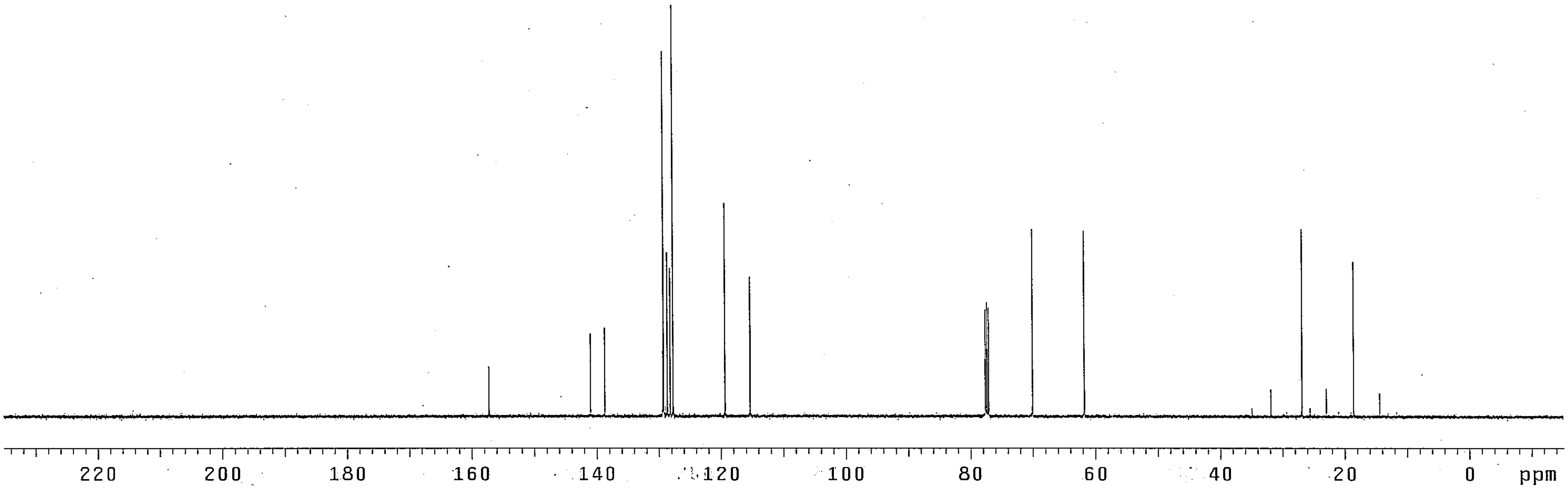

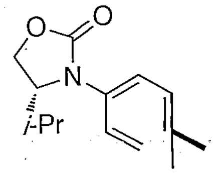

23c

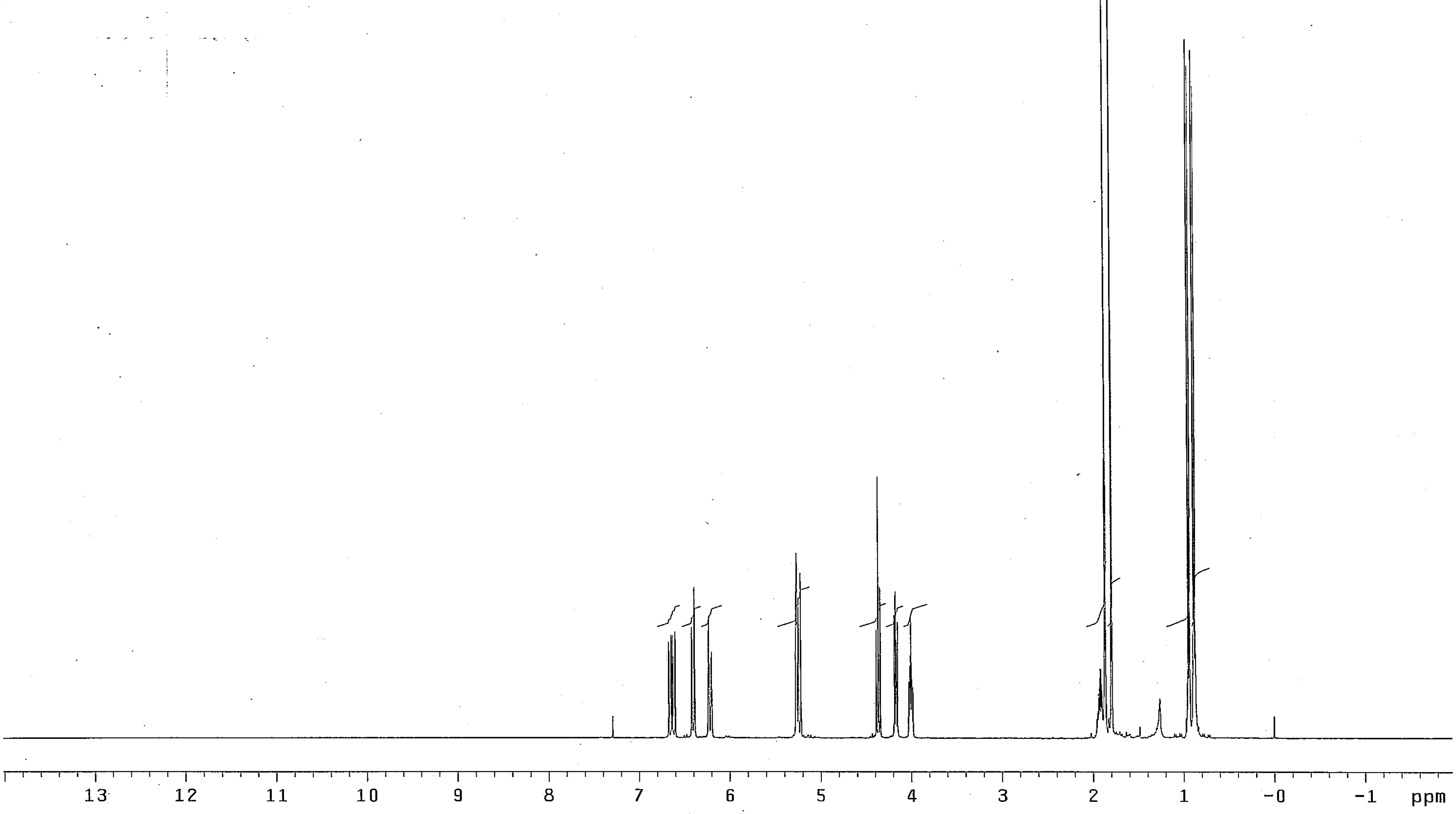

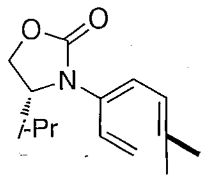

23c

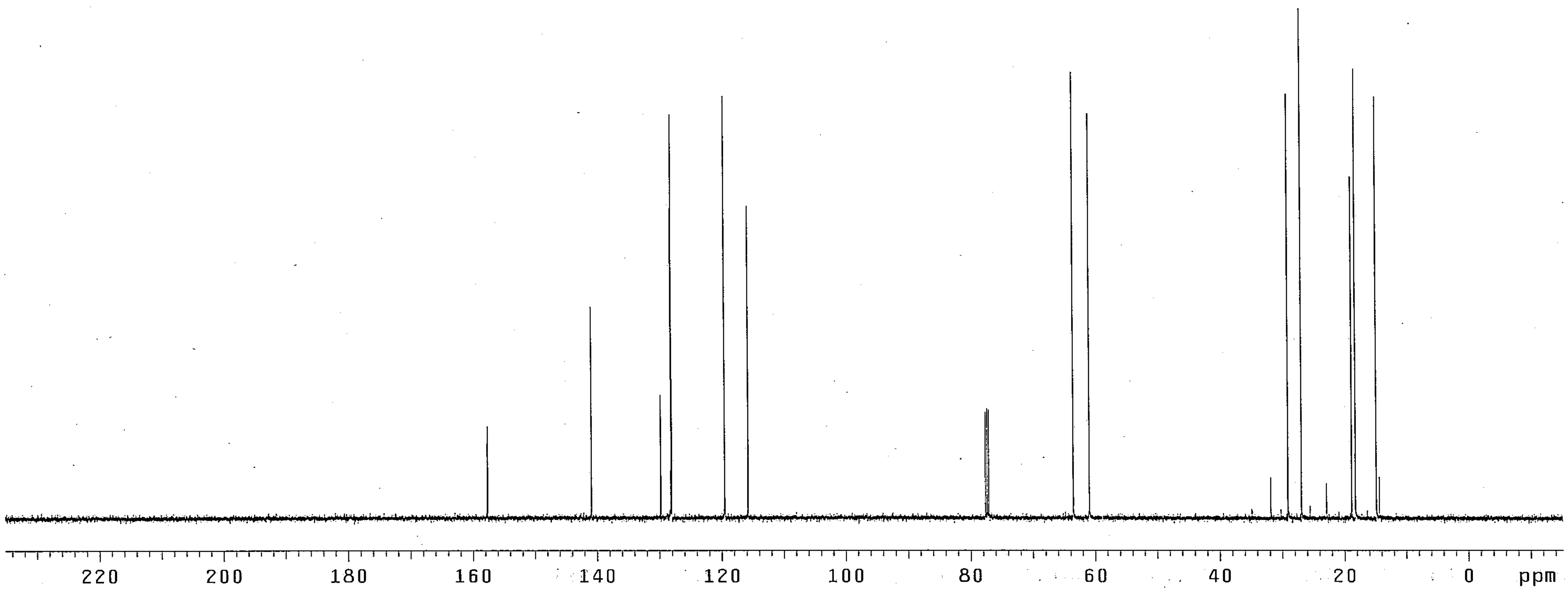

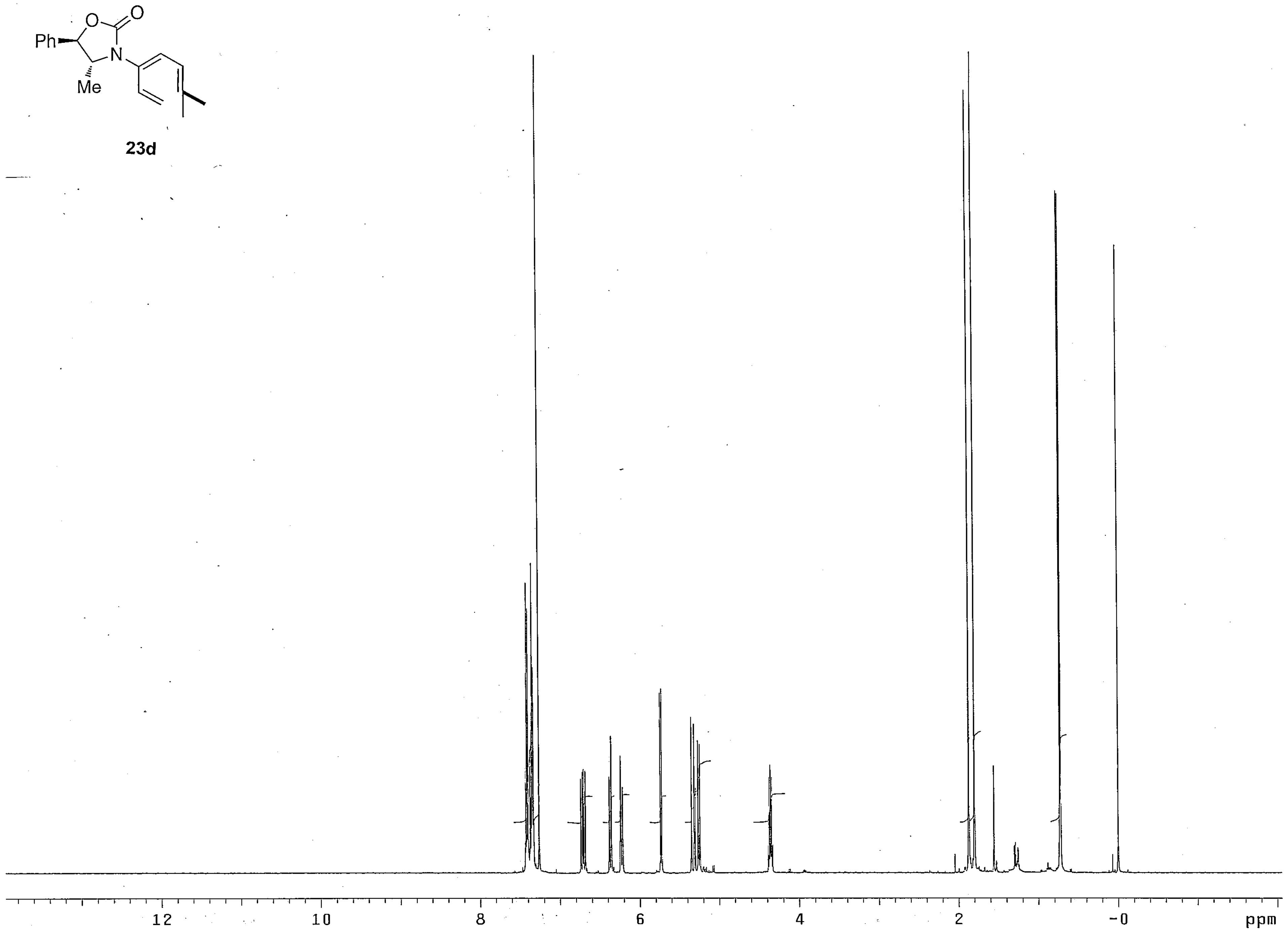

23d

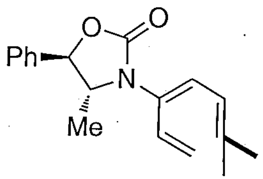

23d

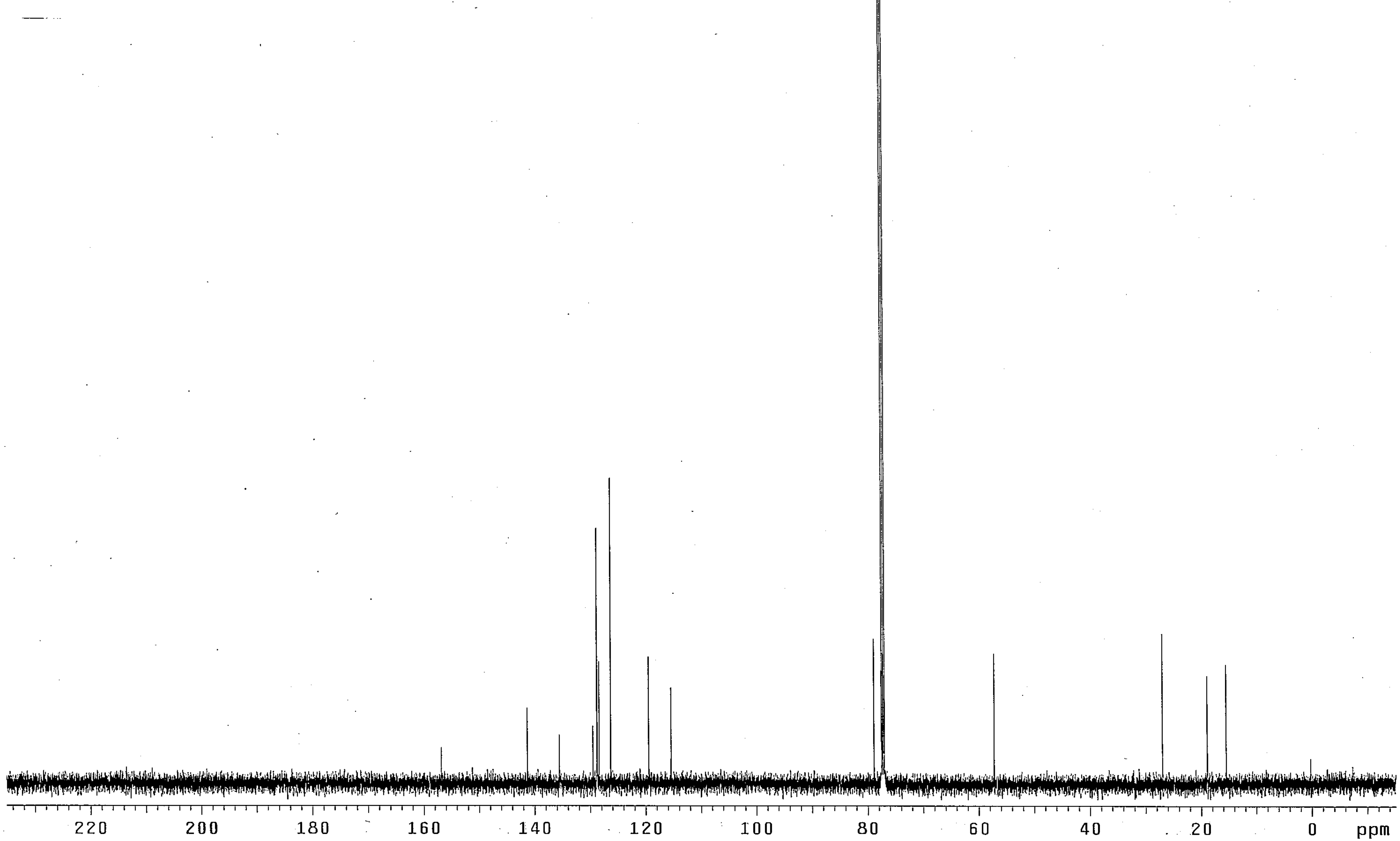

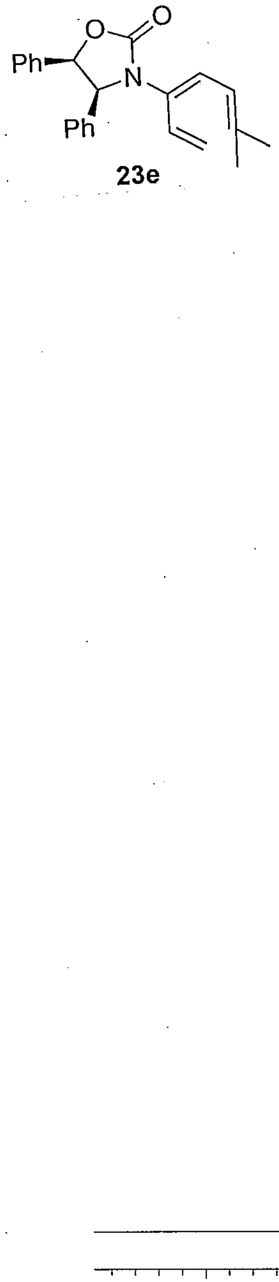

23e

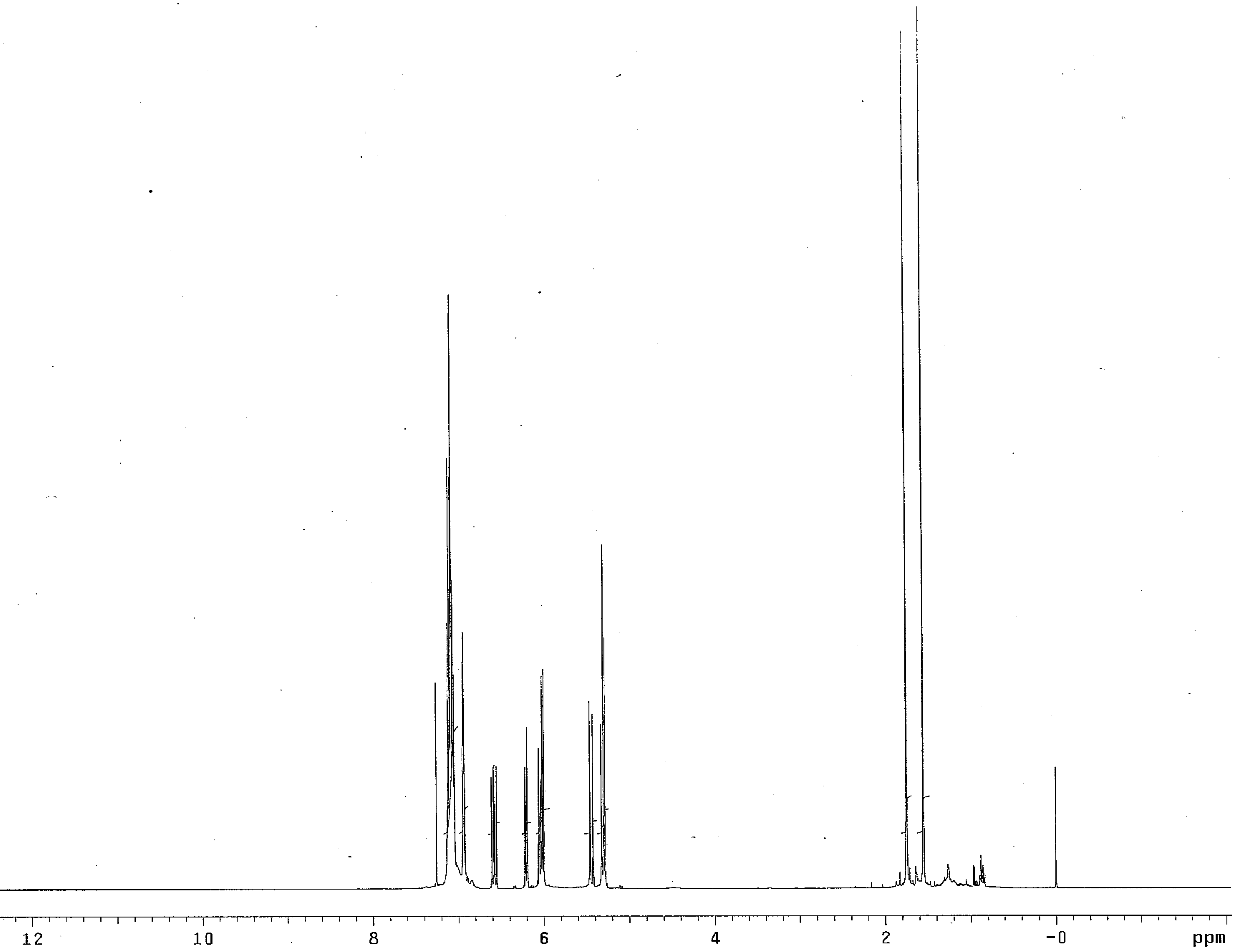

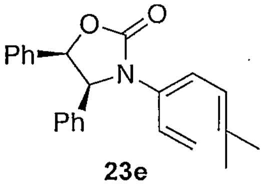

23e

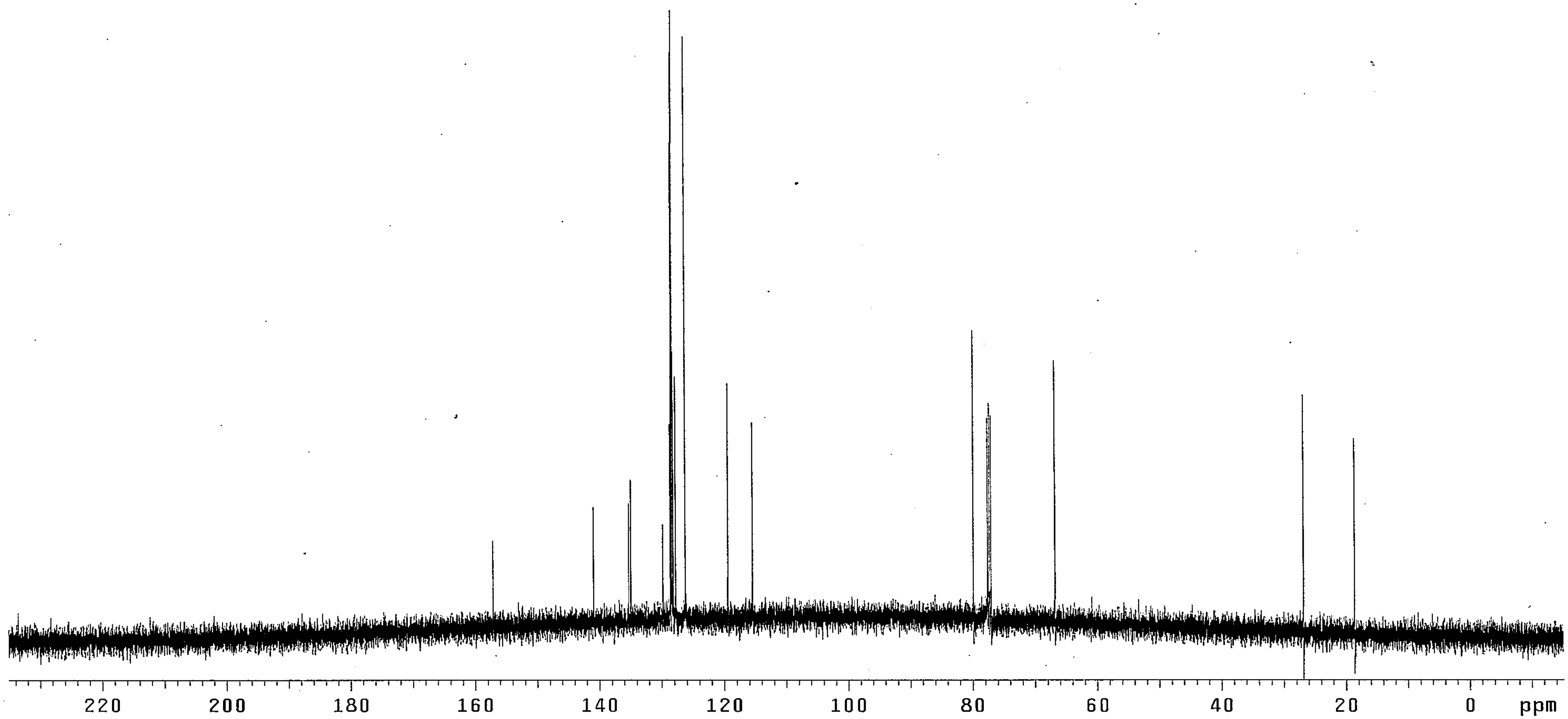

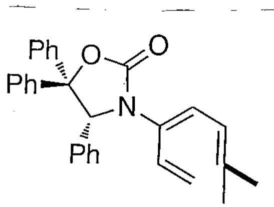

23f

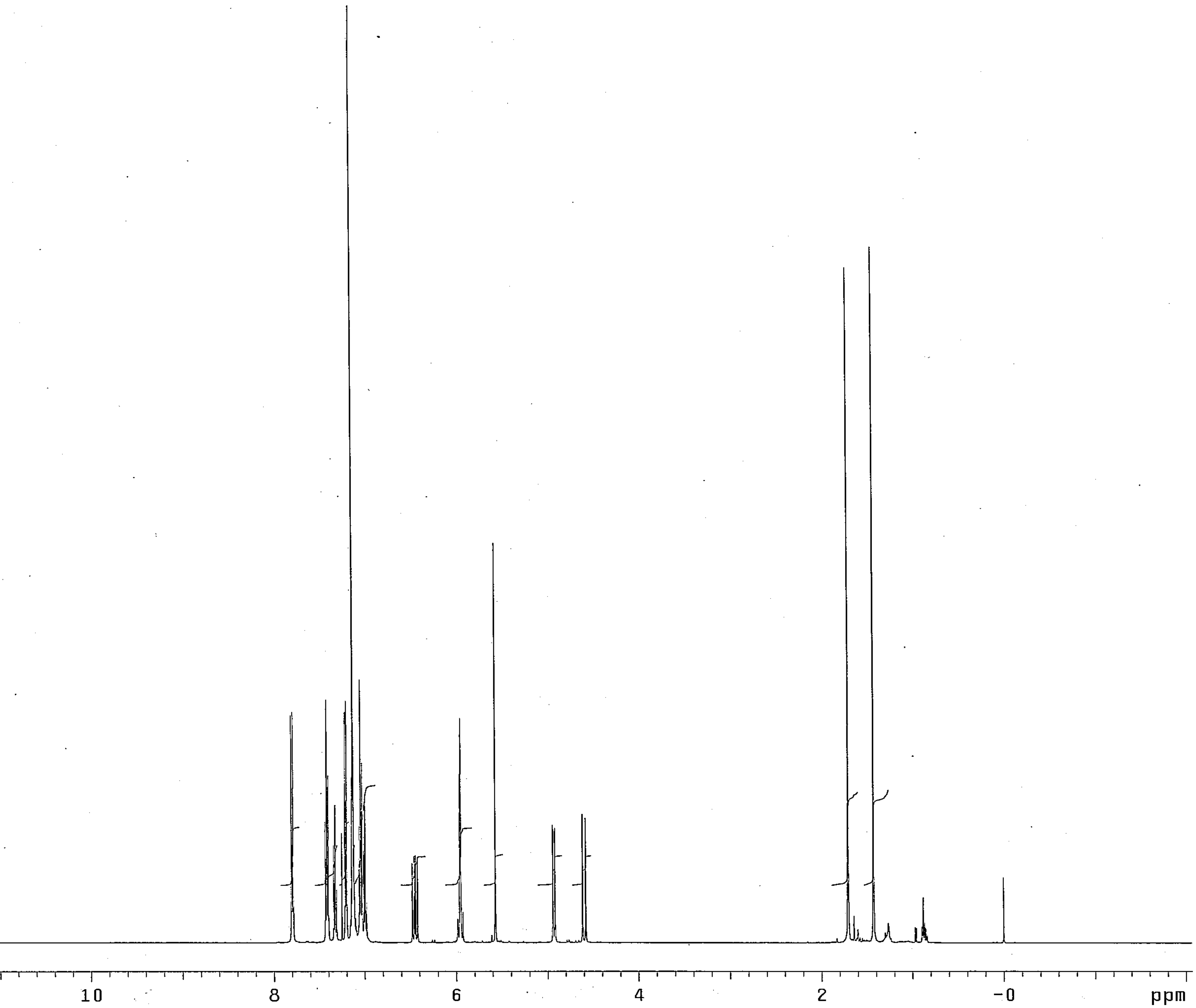

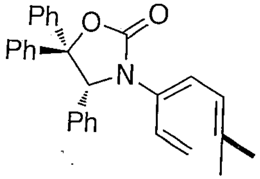

23f

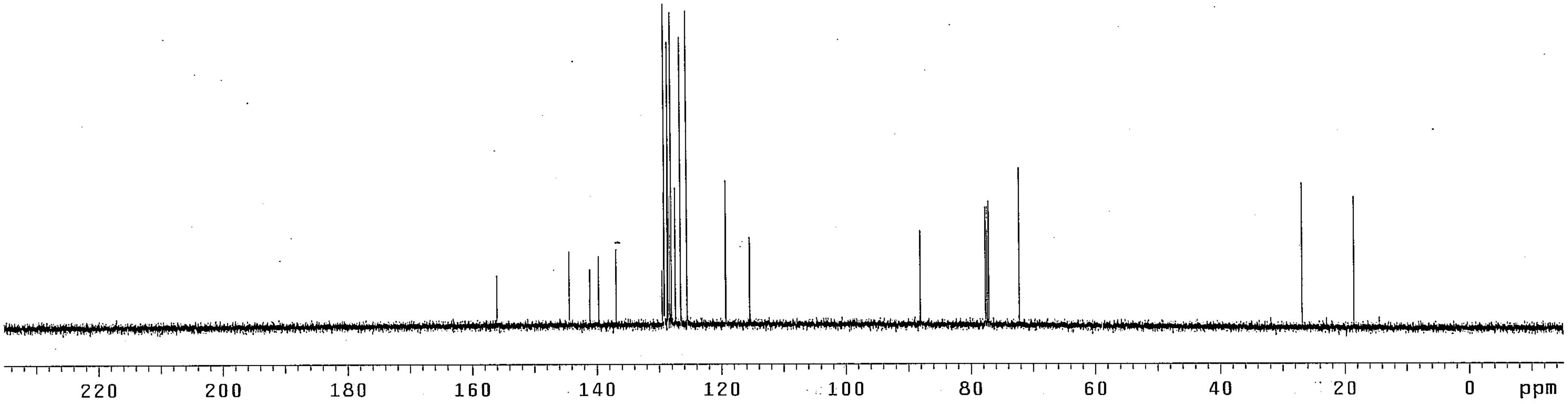

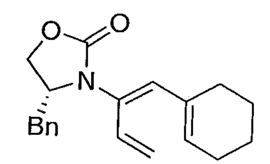

24

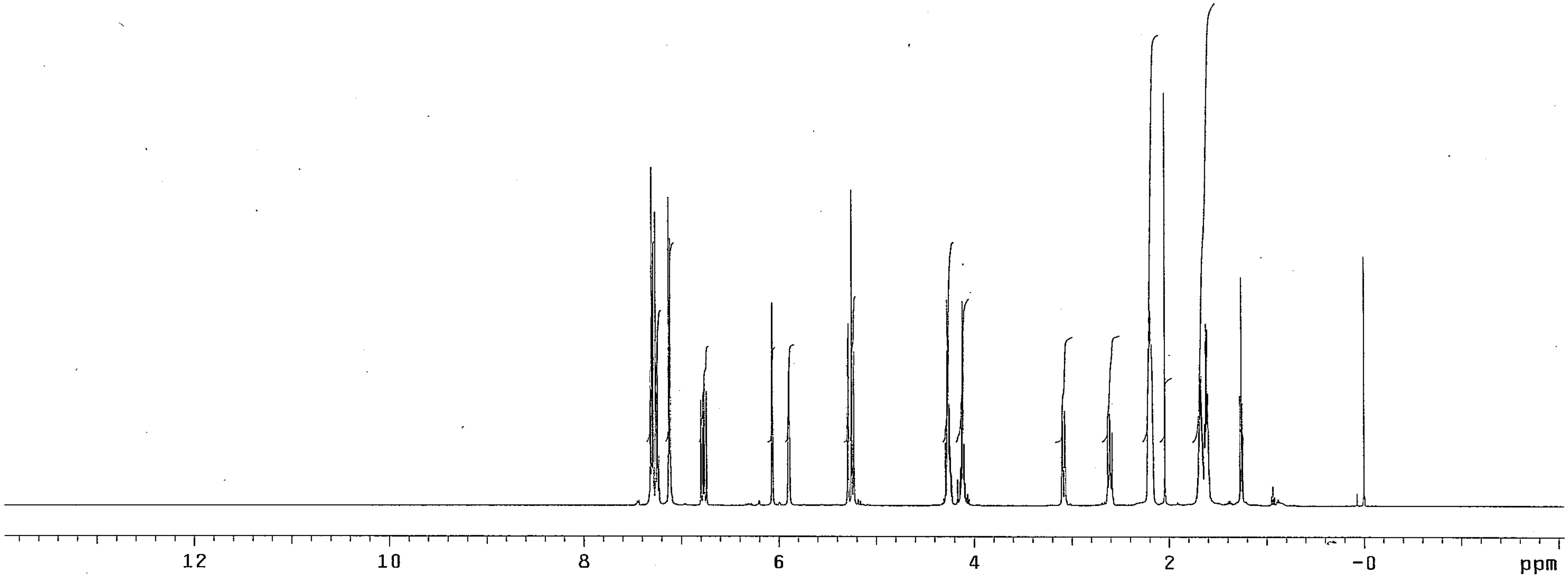

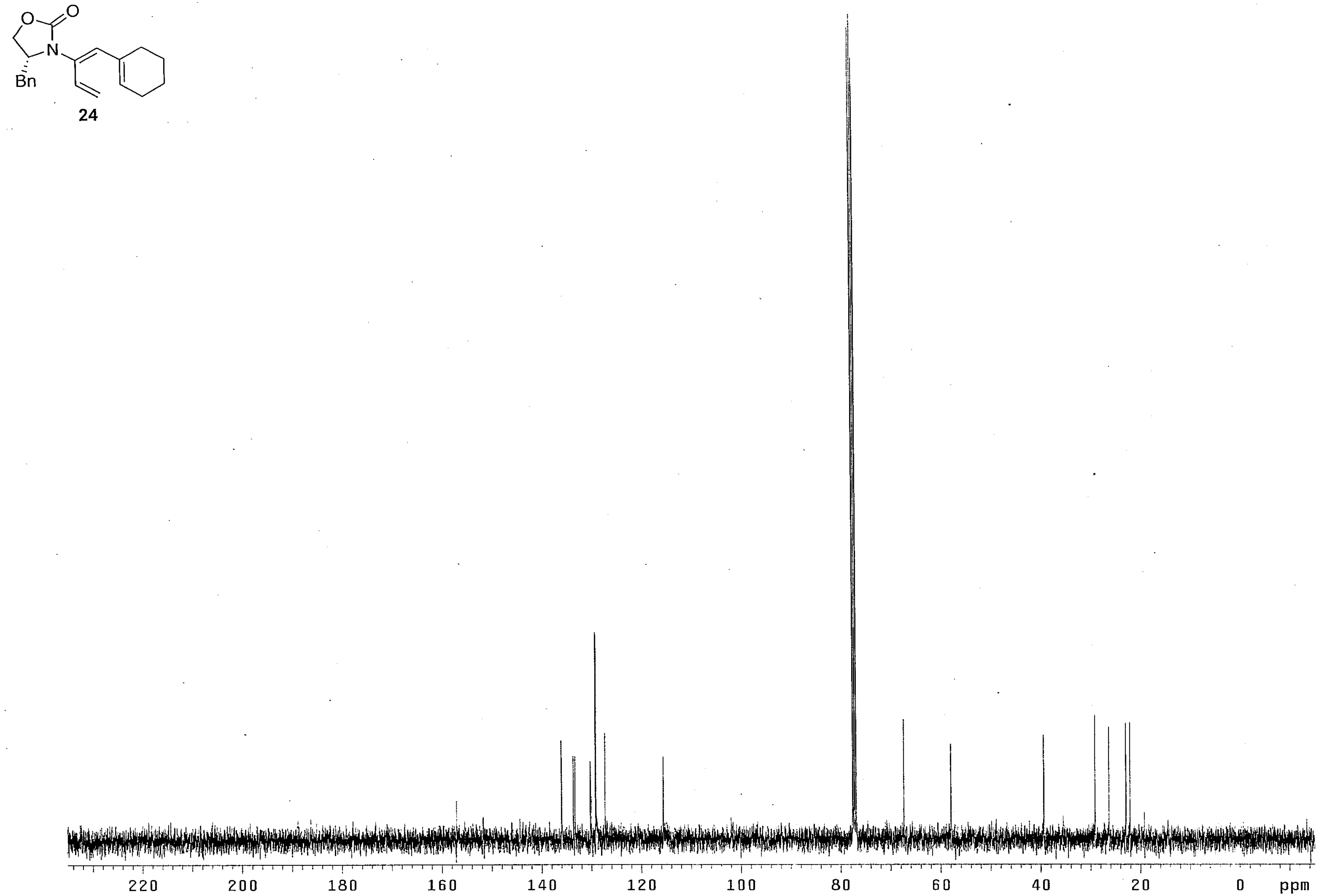
O
N
Bn
24
220 200 180 160 140 120 100 80 60 40 20 0 ppm

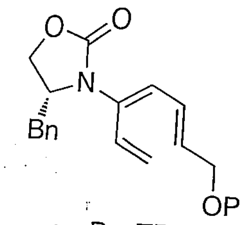

25a: P = TBDPS

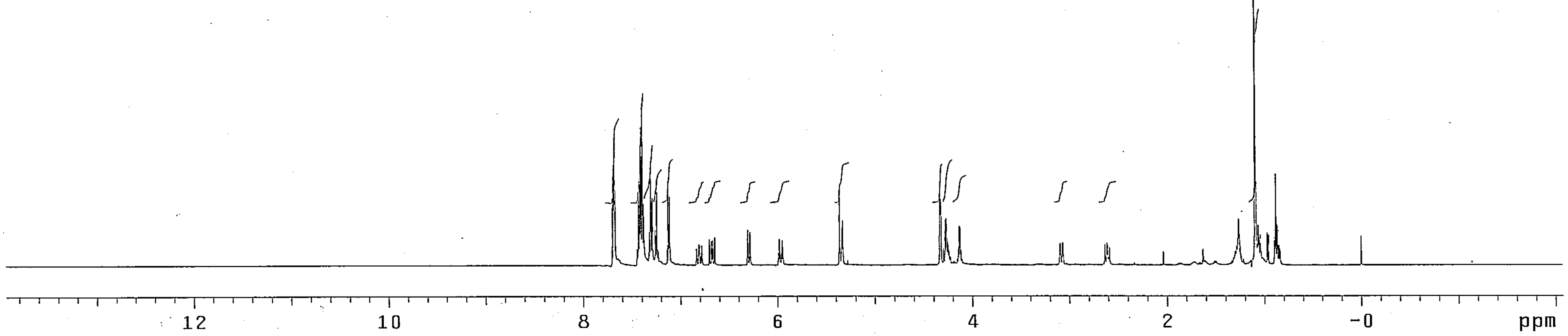

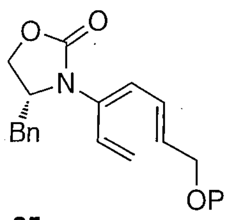

25a: P = TBDPS

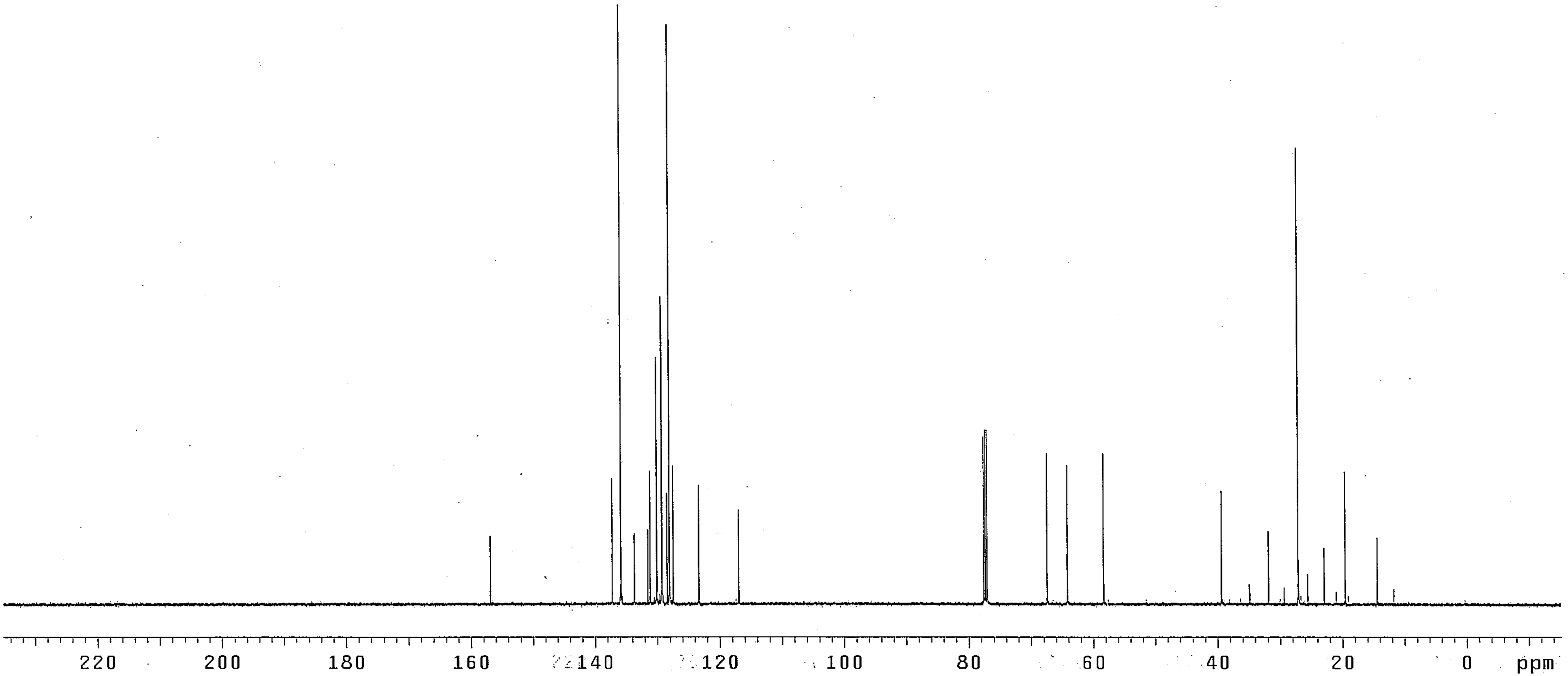

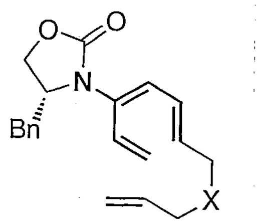

25b: X = O

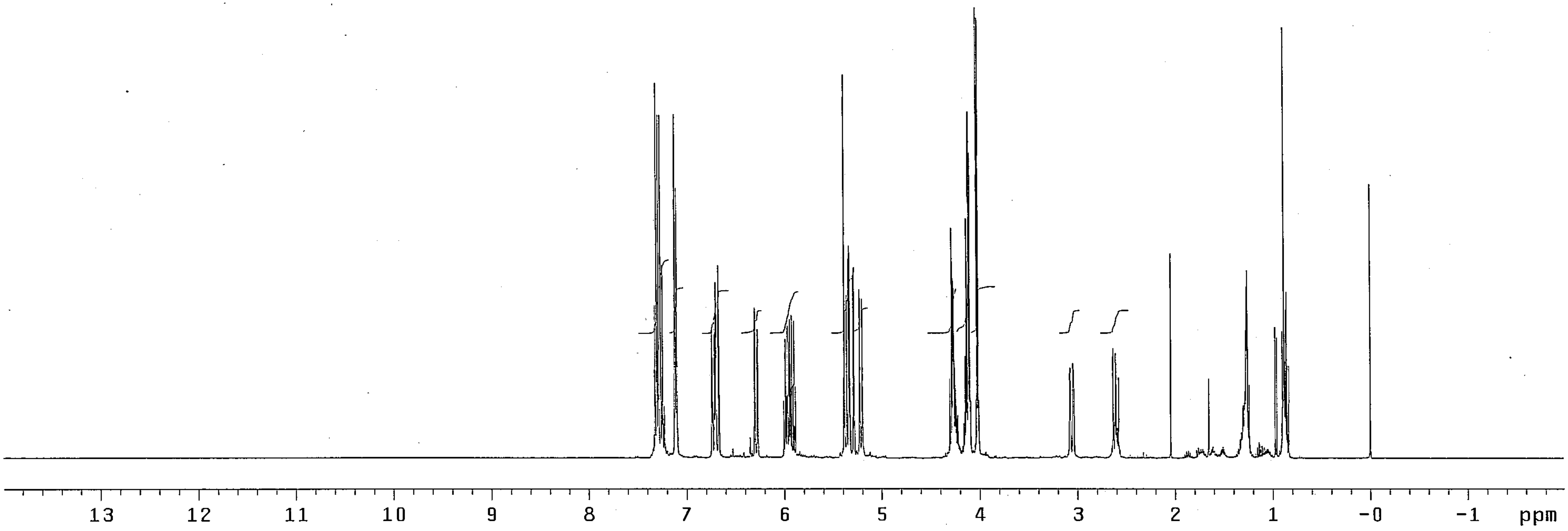

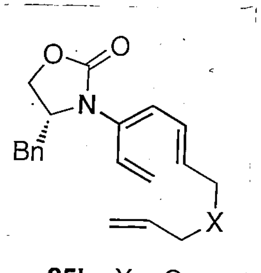

25b: X = O

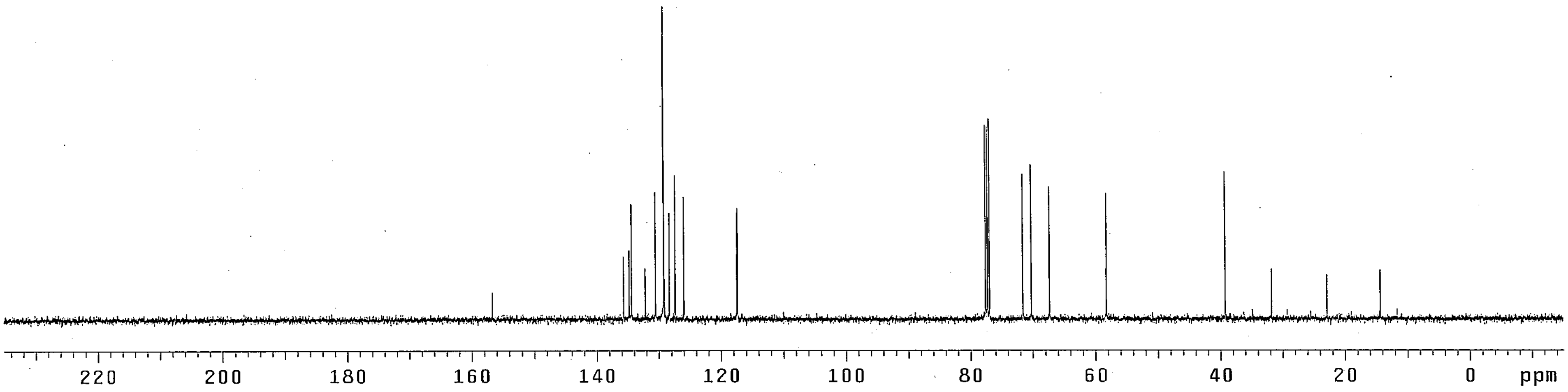

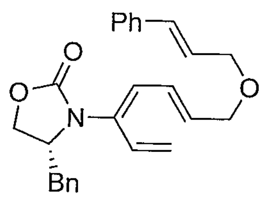

25c

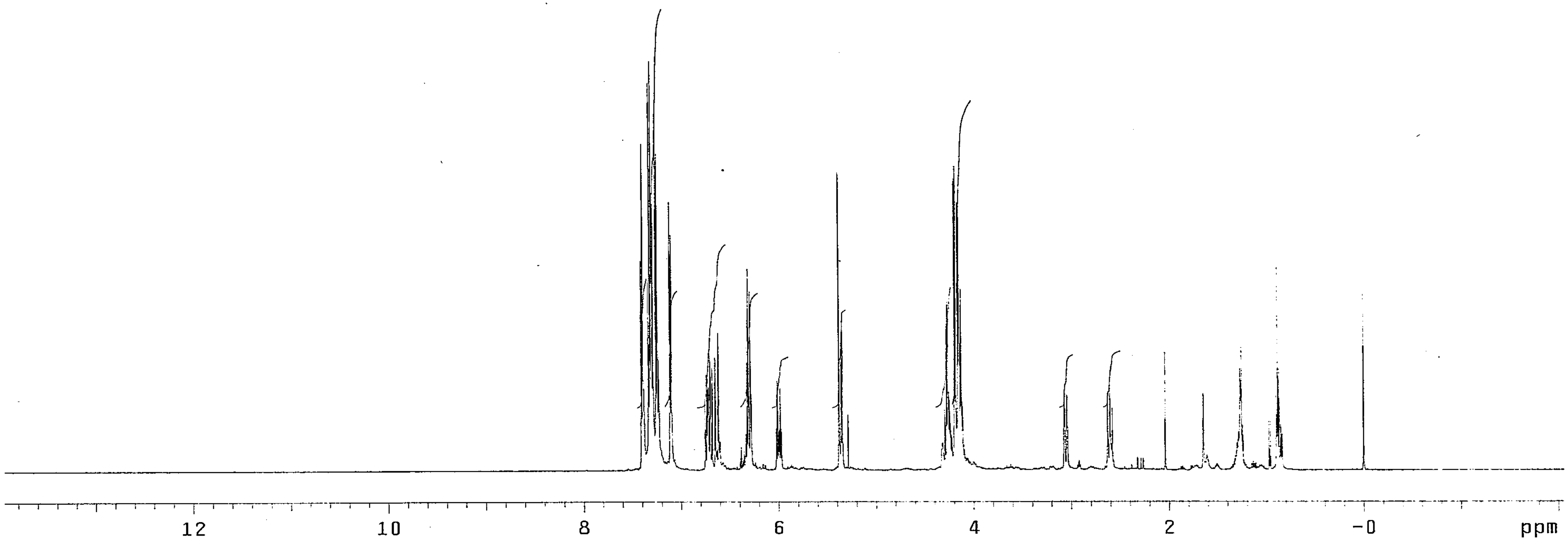

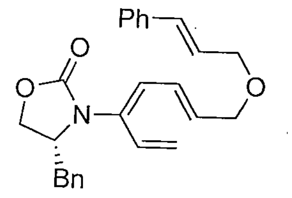

25c

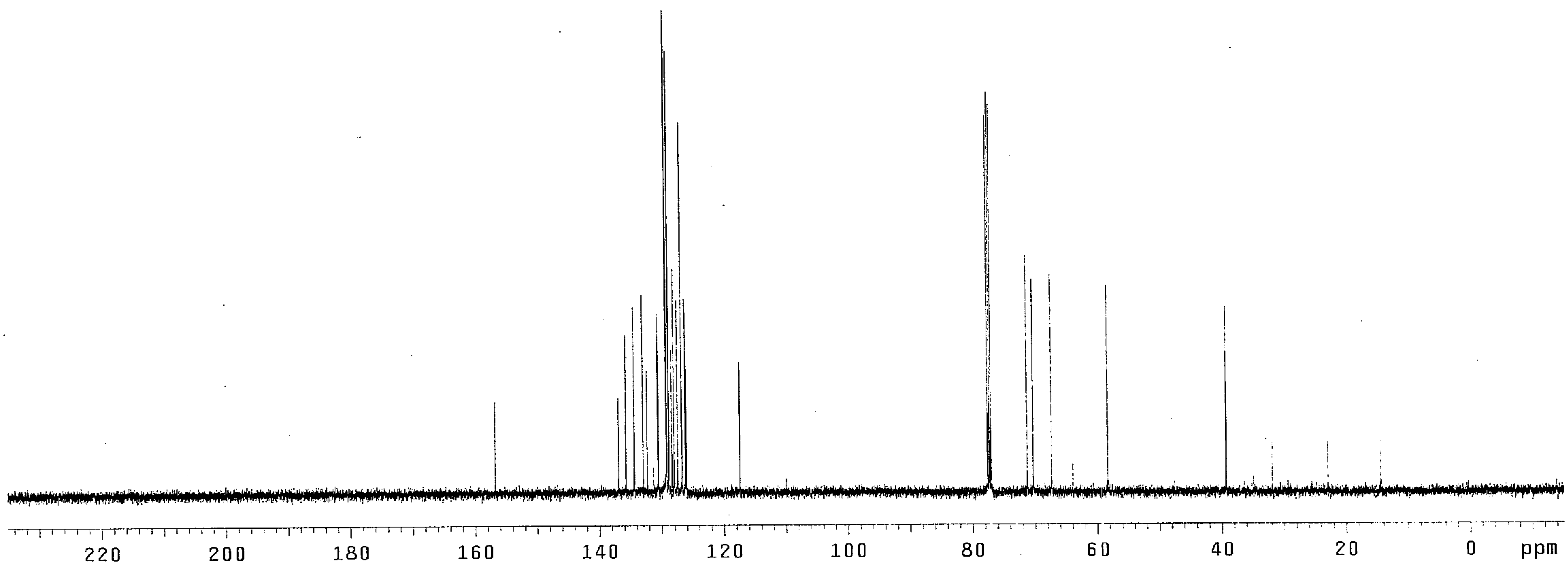

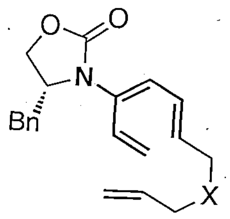

26 X = NTs

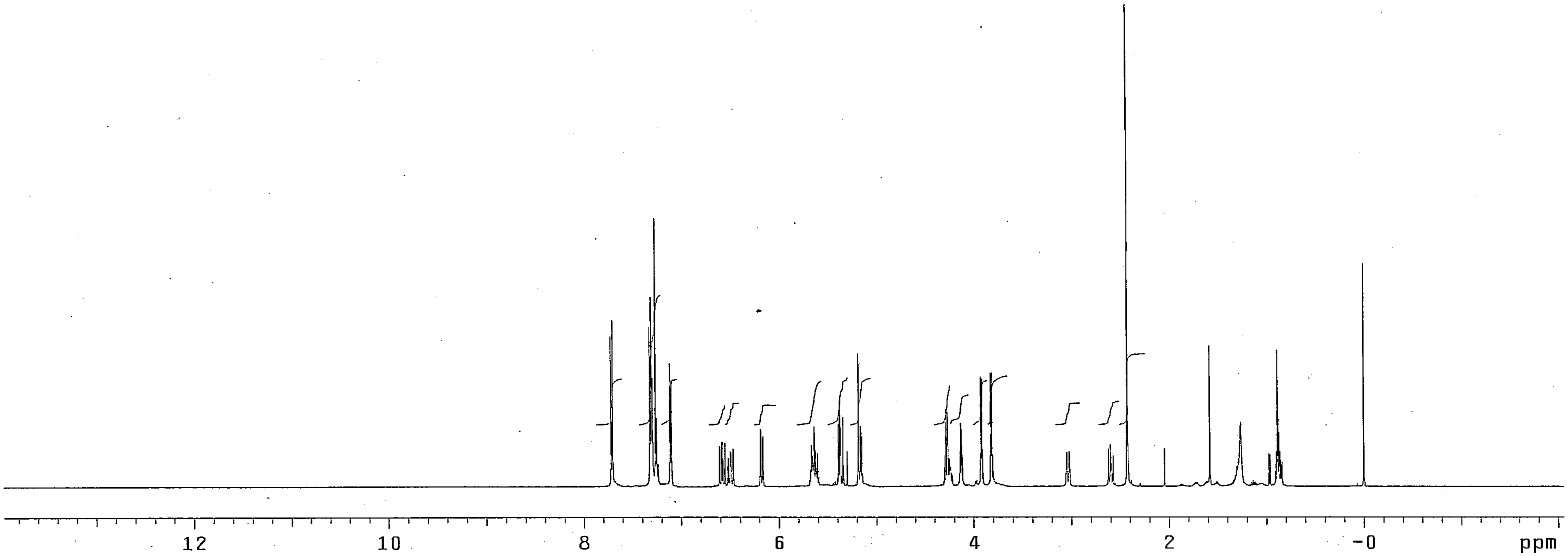

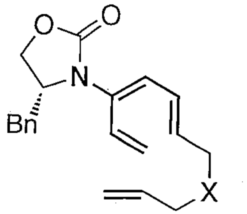

26 X = NTs

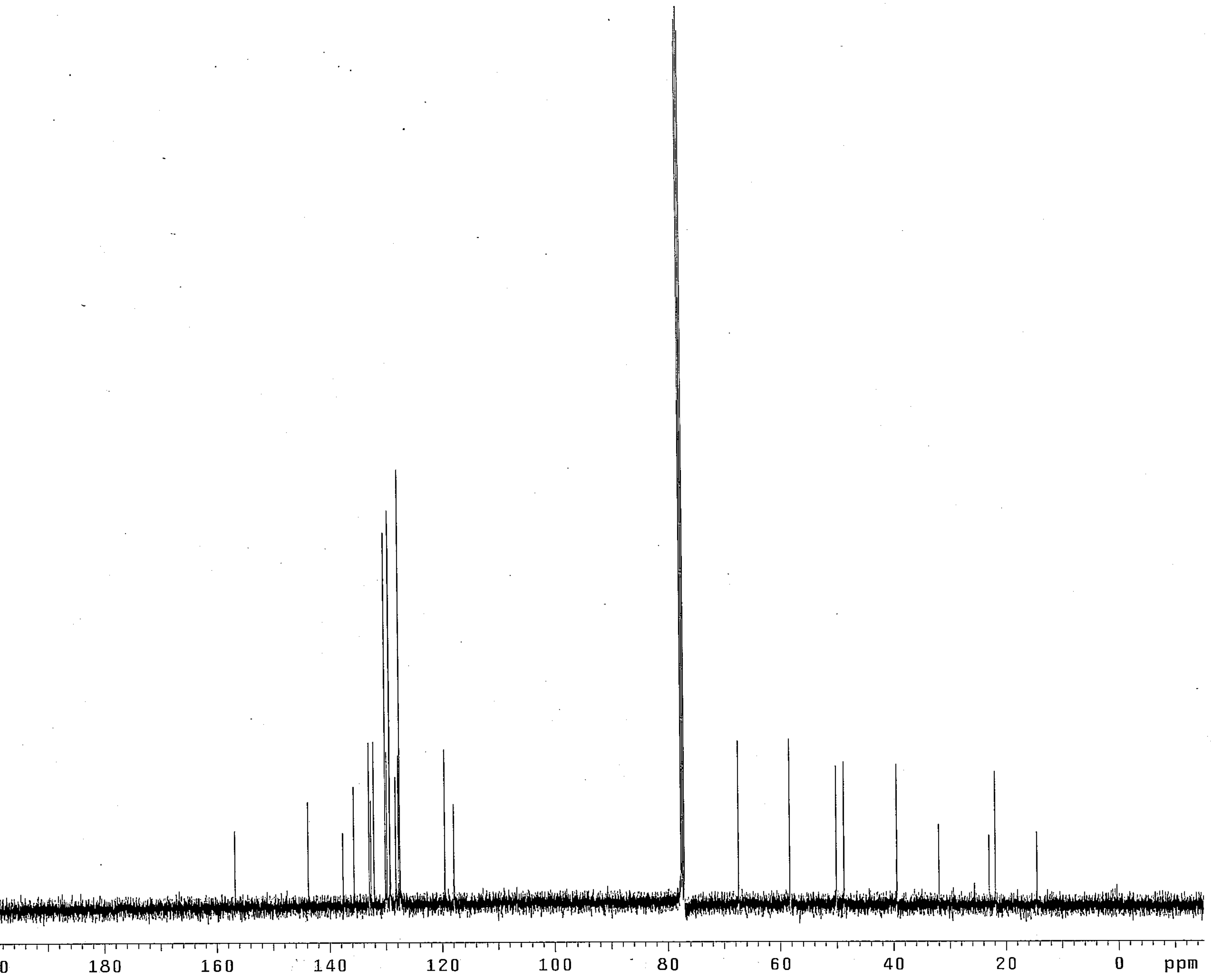

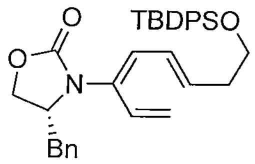

27

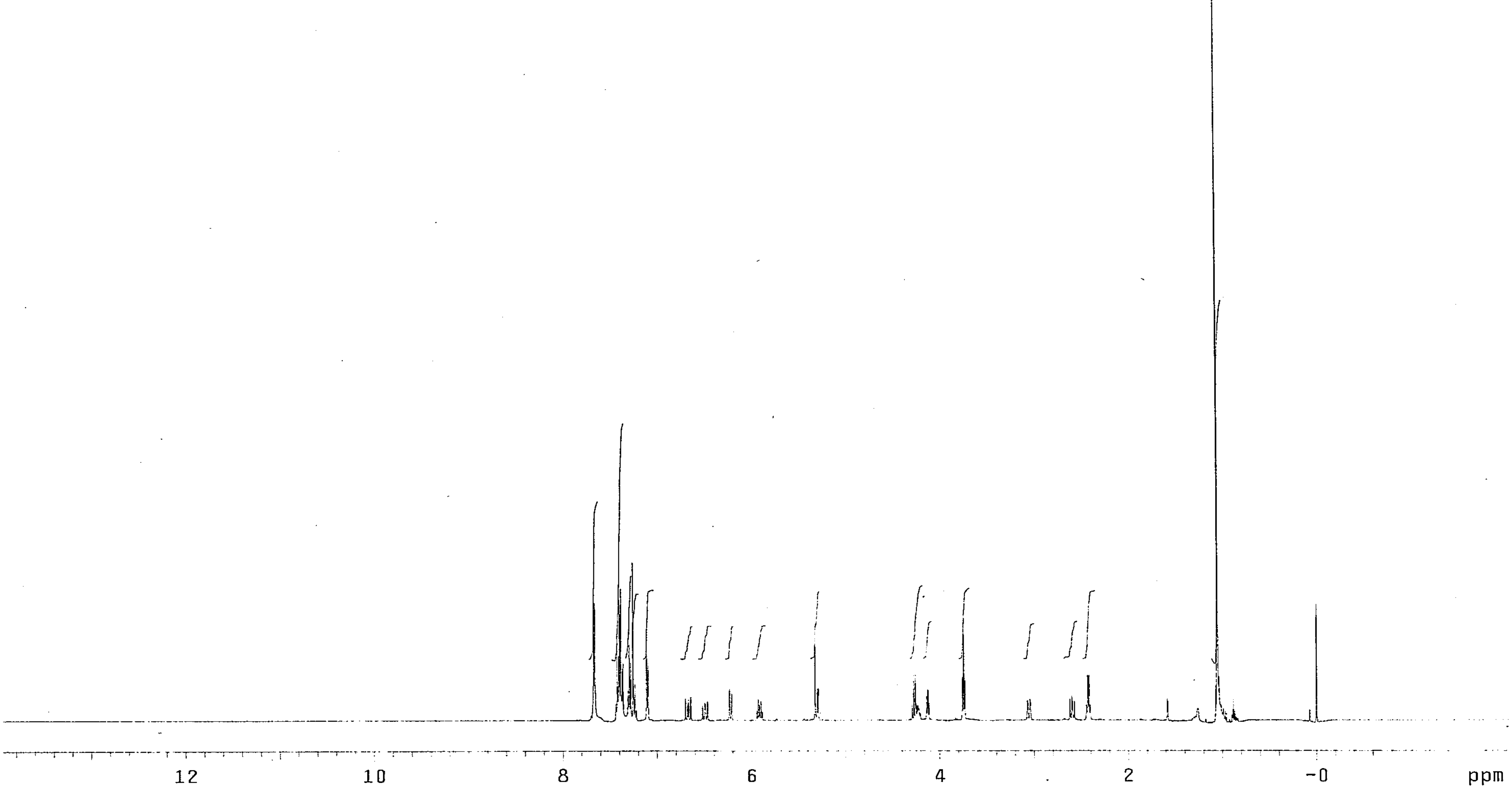

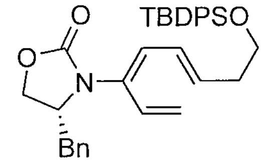

27

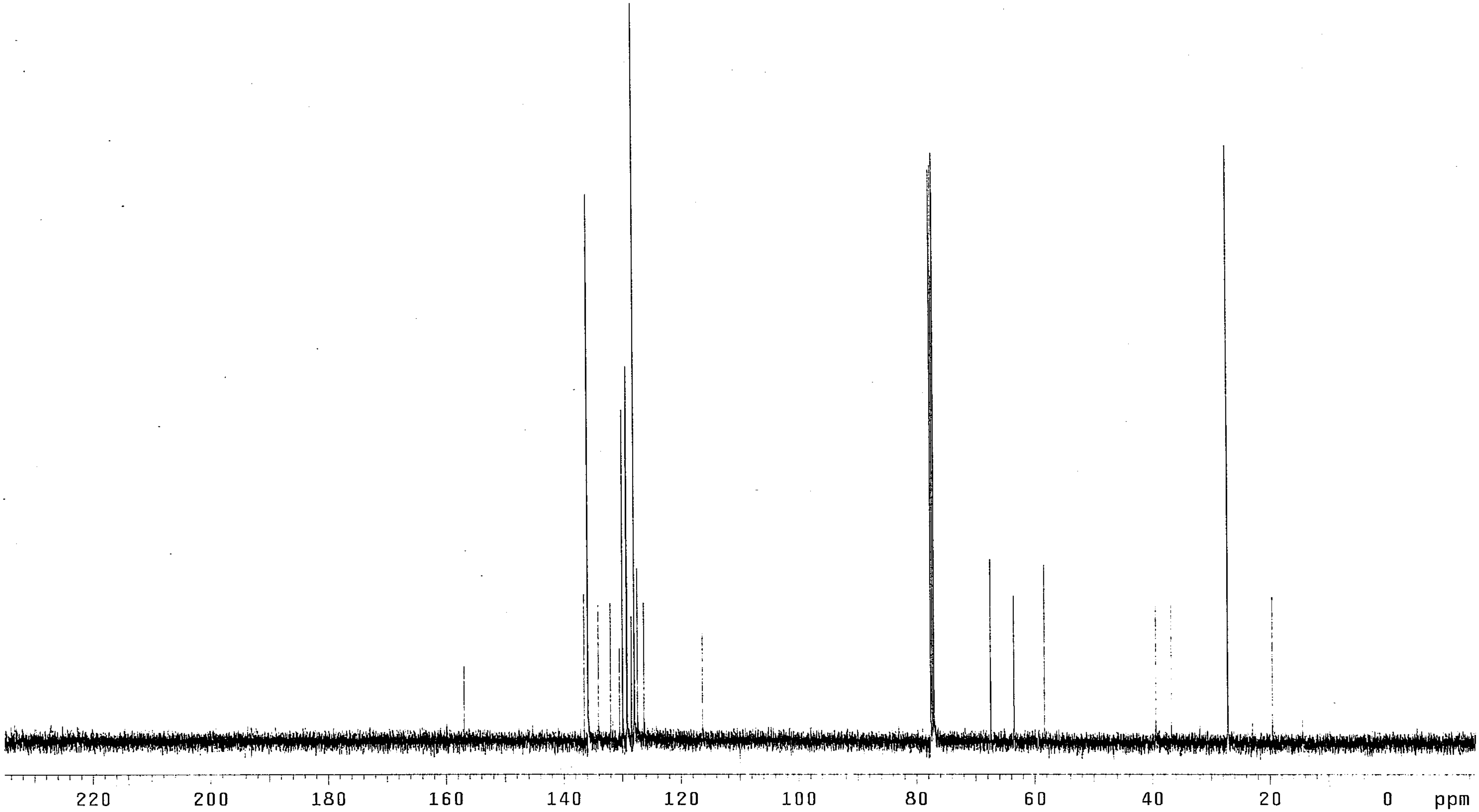

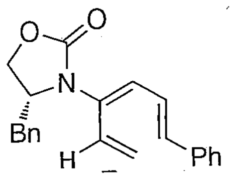

28

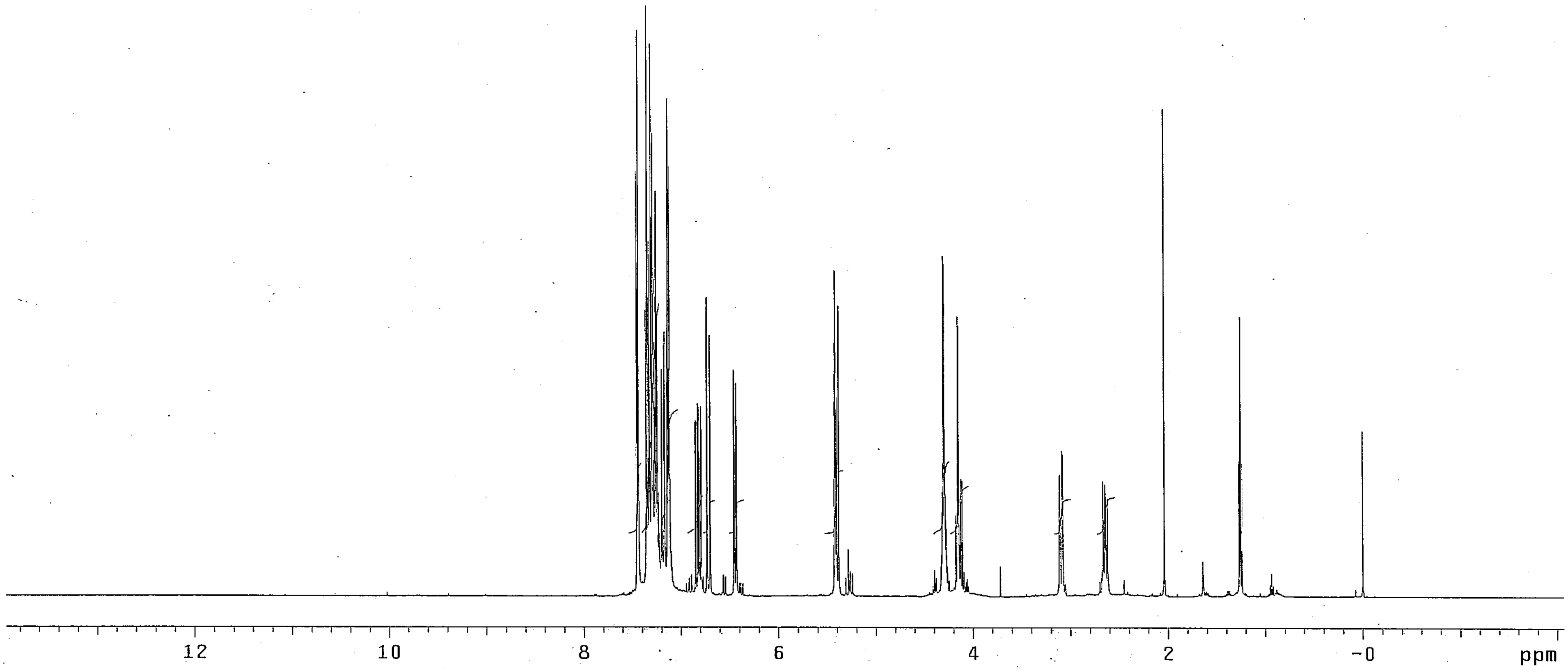

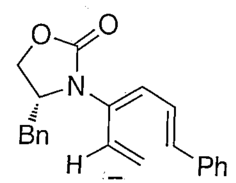

28

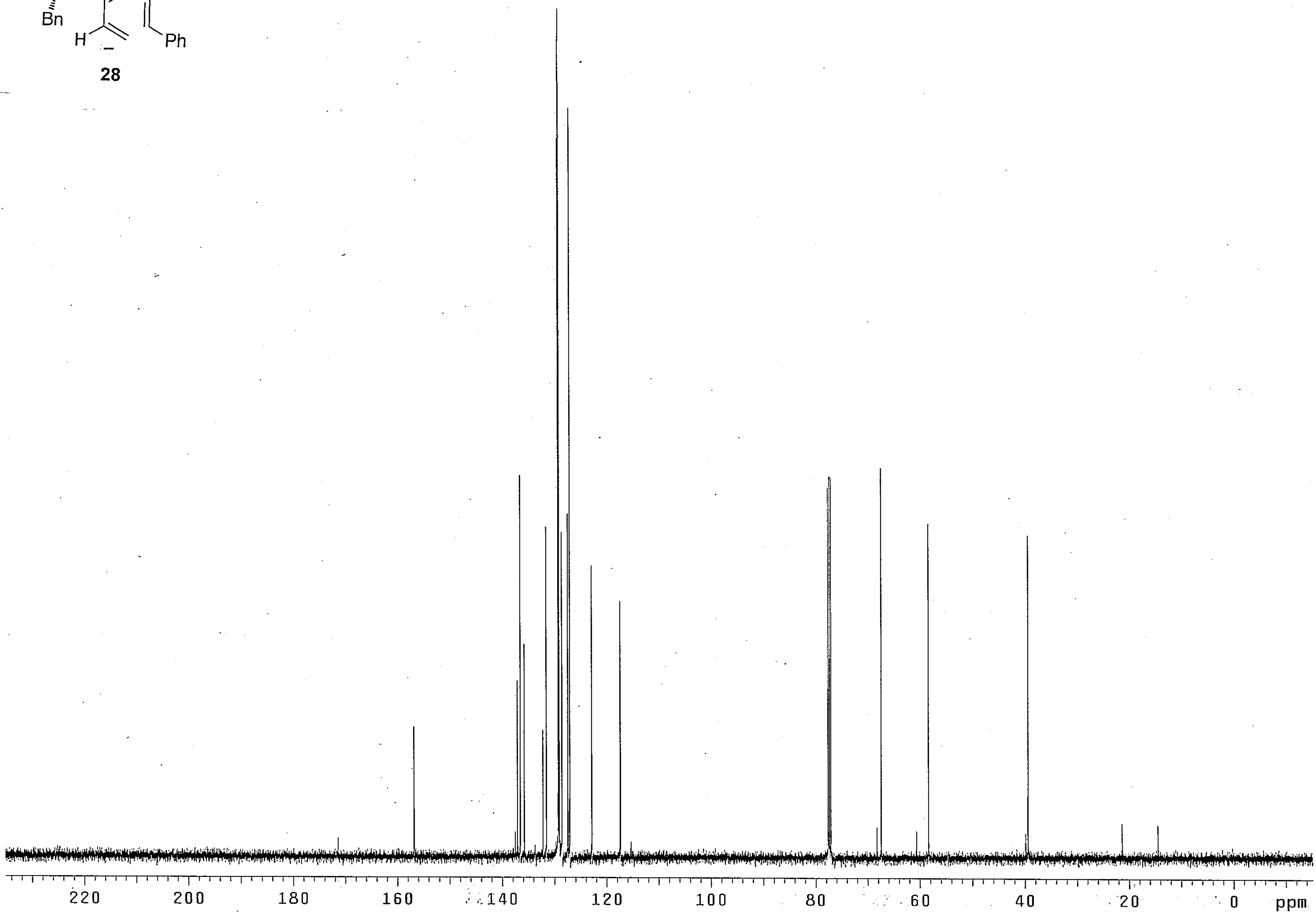

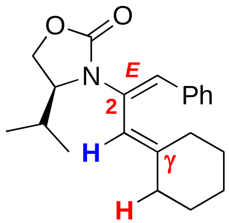

33a

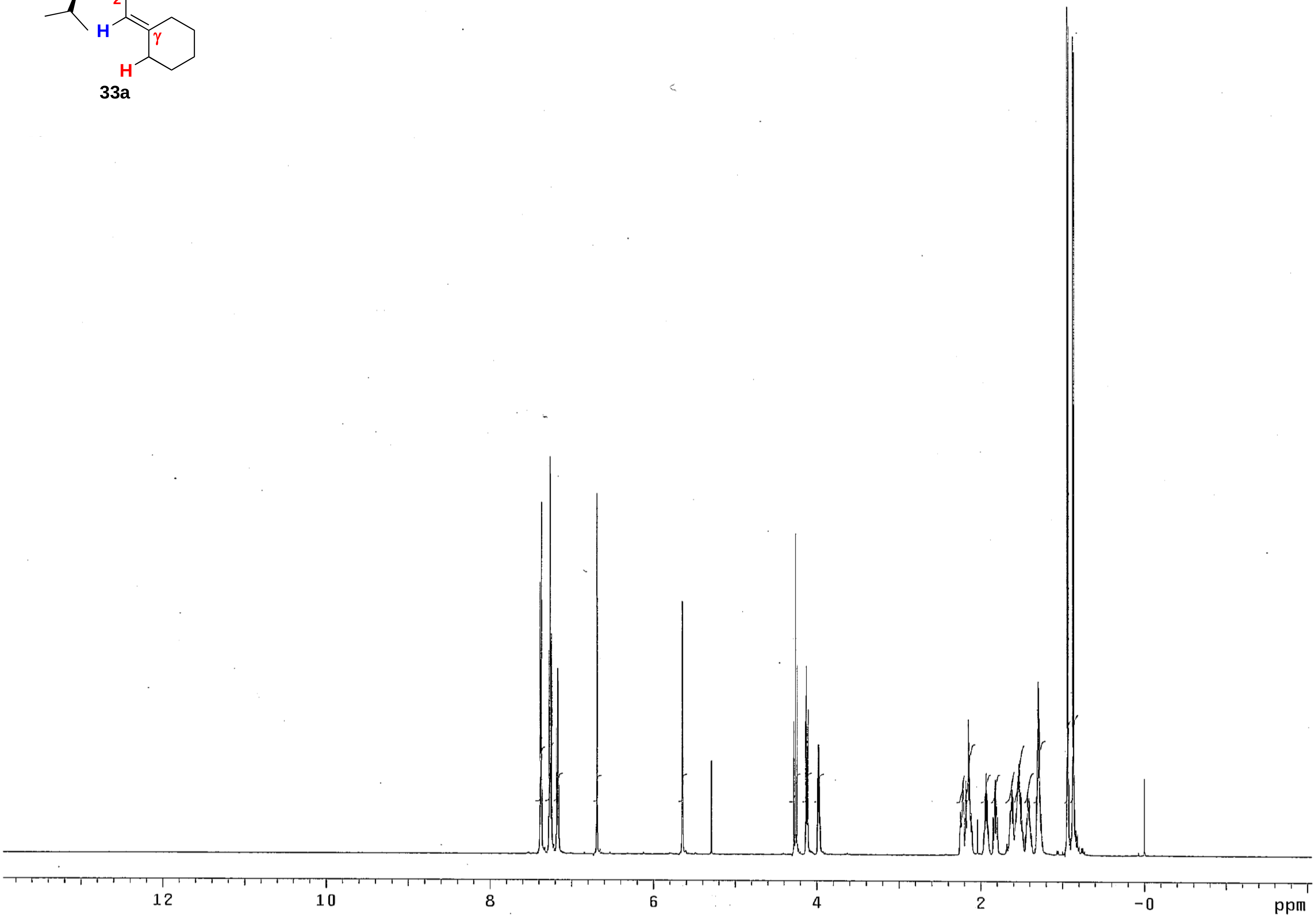

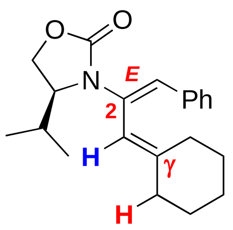

33a

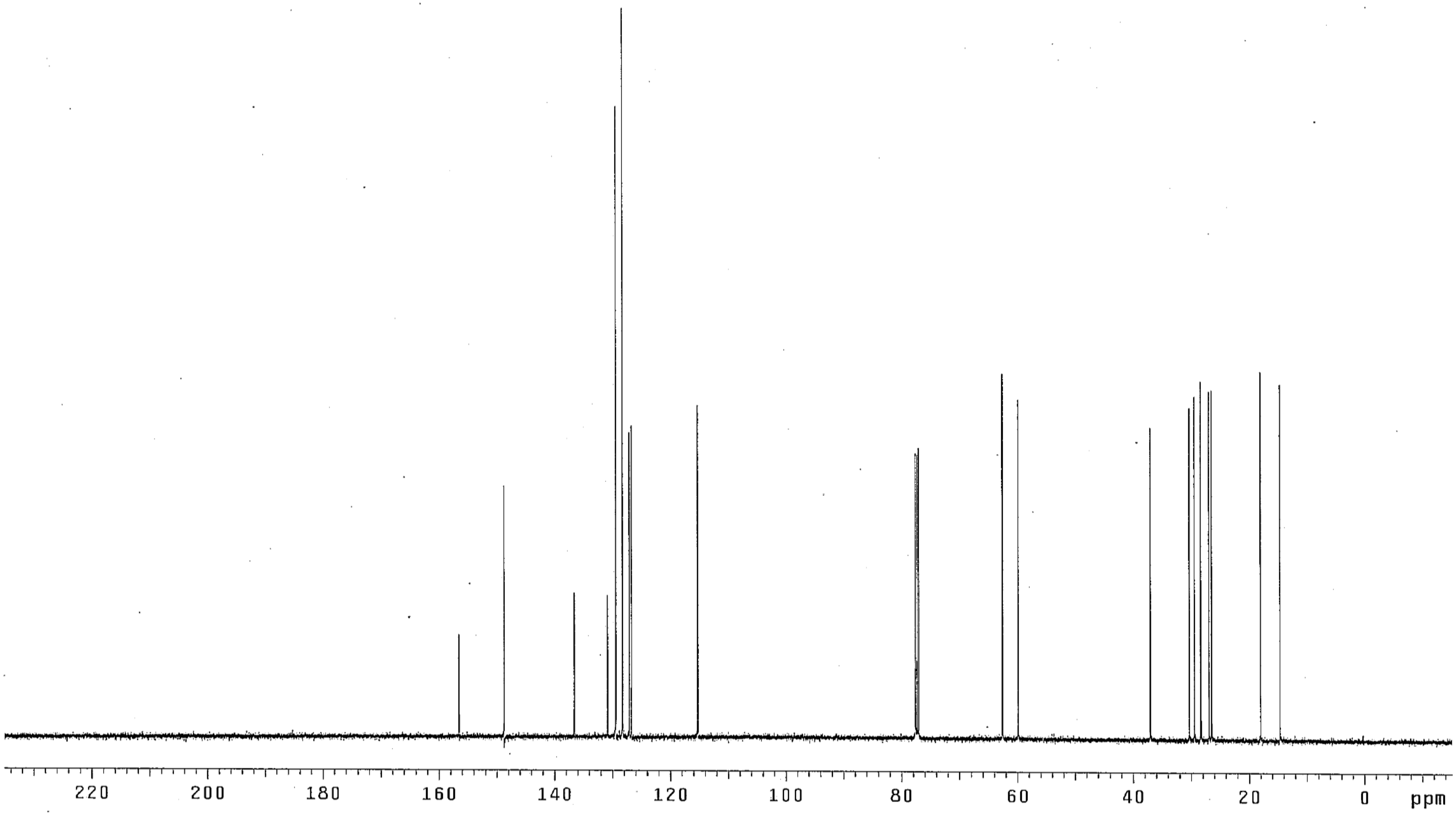

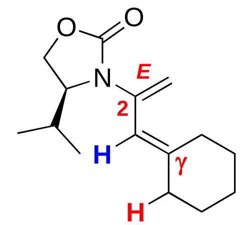

33b

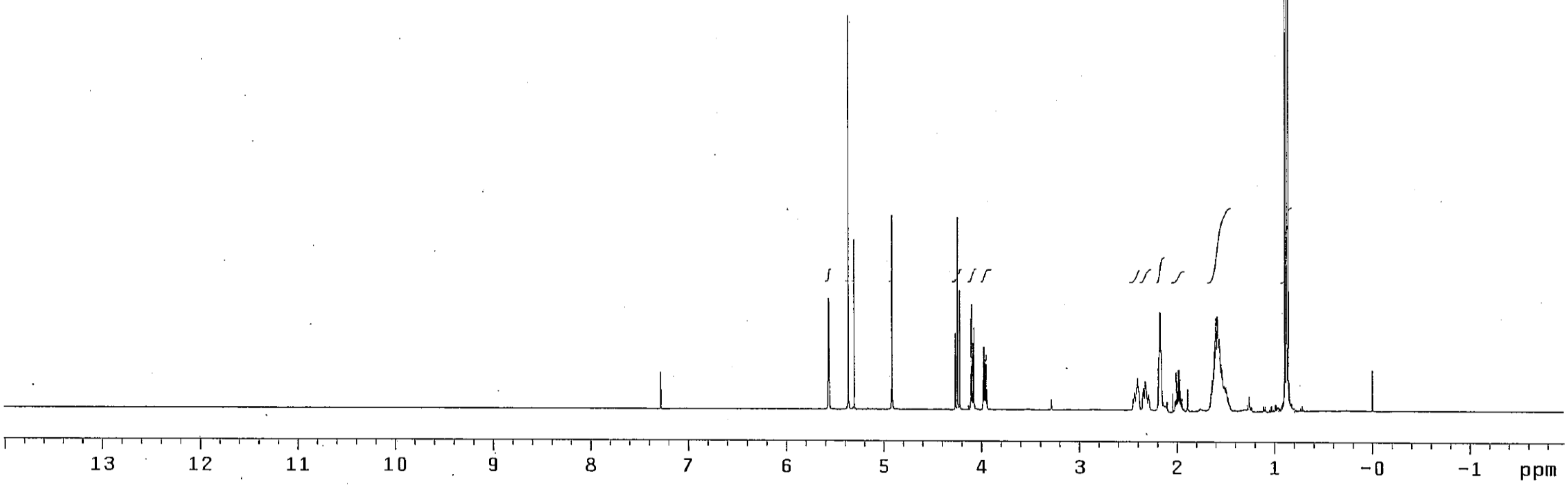

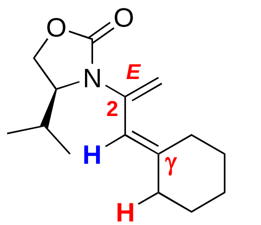

33b

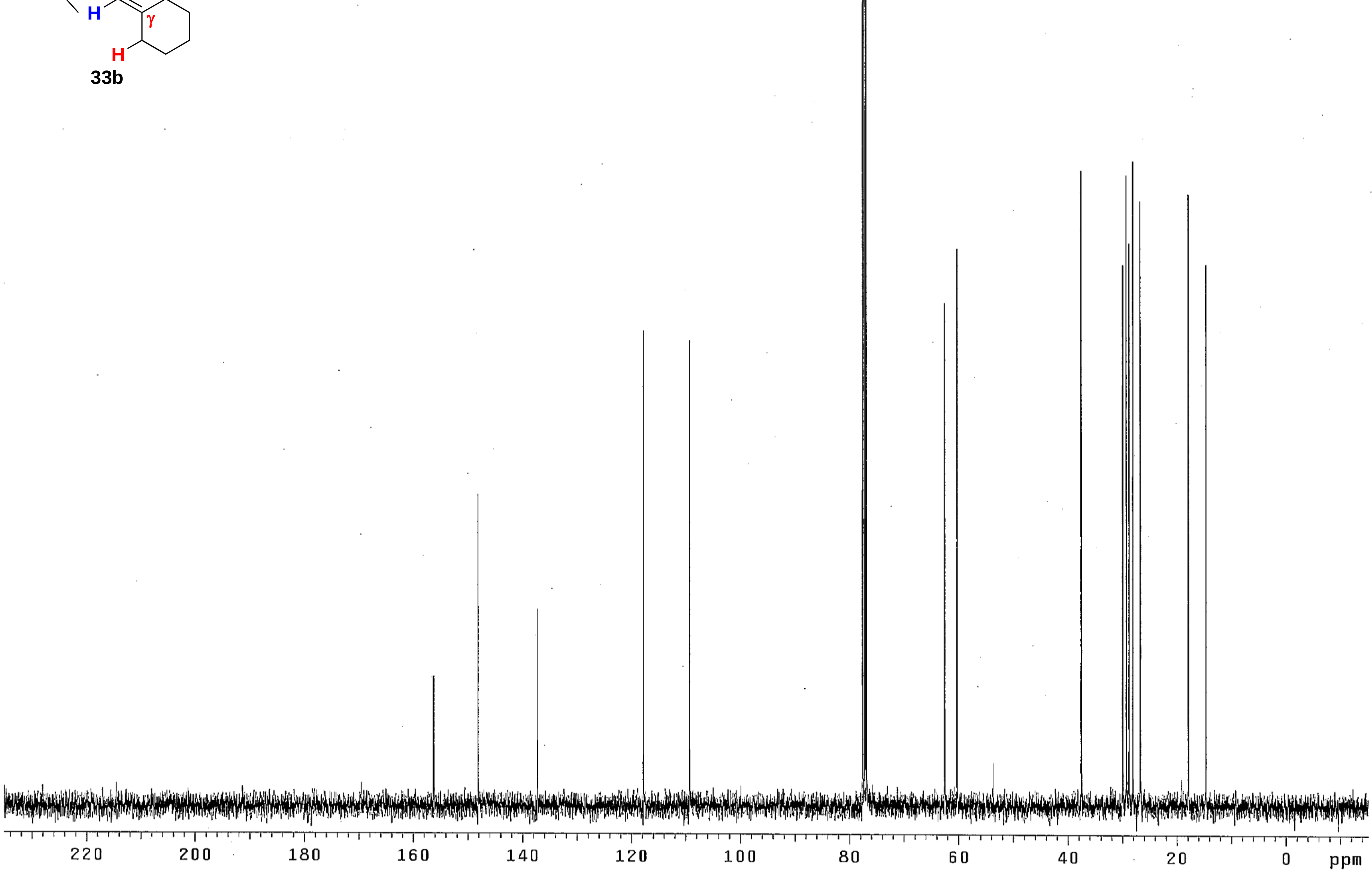

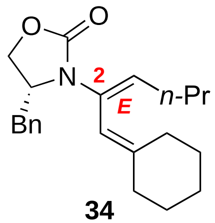

34

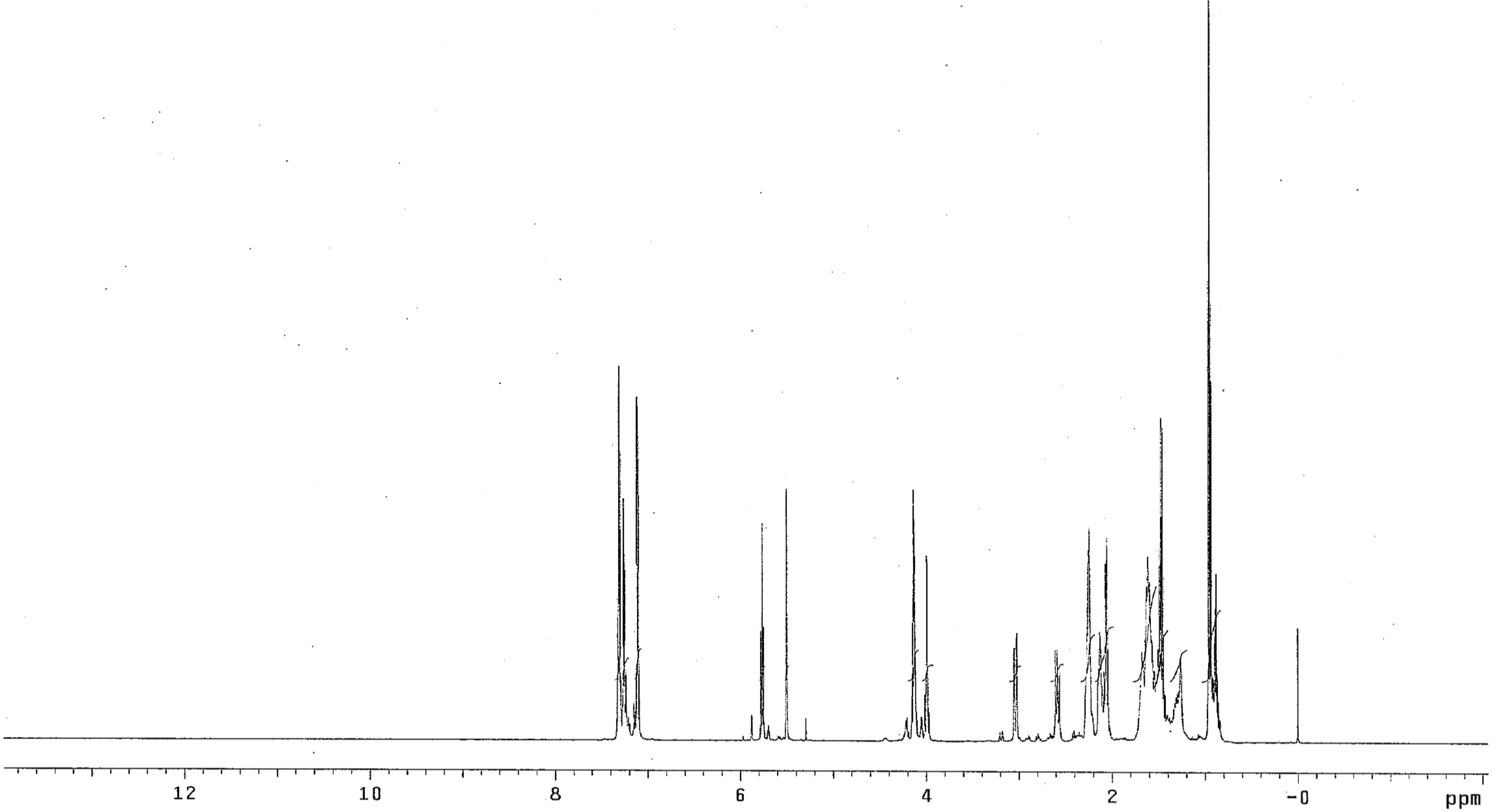

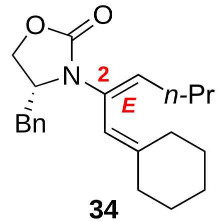

34

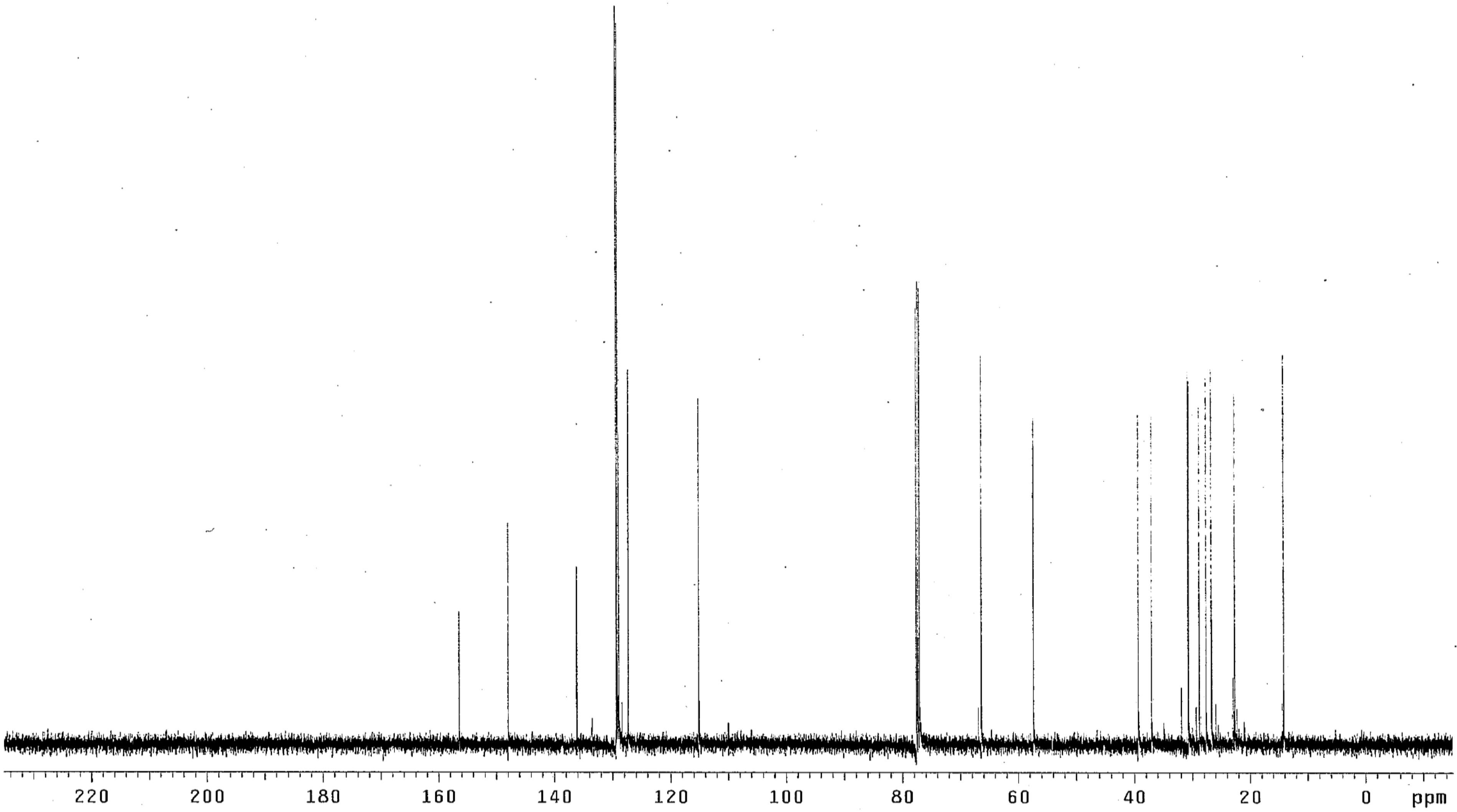

RH-2-140cosy_28Mar2009

Archive directory: /export/home/ryuji/vnmrsys/data
Sample directory: RH-2-140cosy_28Mar2009

Pulse Sequence: gCOSY

Solvent: CDCl3
Temp. 25.0 C / 298.1 K
File: gCOSY
INOVA-500 "nmr03"

Relax. delay 1.000 sec
Acq. time 0.128 sec
Width 8000.0 Hz
2D Width 8000.0 Hz
Single scan
128 increments
OBSERVE H1, 499.7288165 MHz
DATA PROCESSING
Sq. sine bell 0.064 sec
F1 DATA PROCESSING
Sq. sine bell 0.016 sec
FT size 2048 x 2048
Total time 2 min, 45 sec

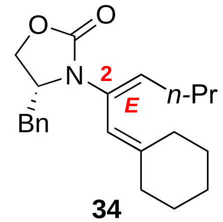

34

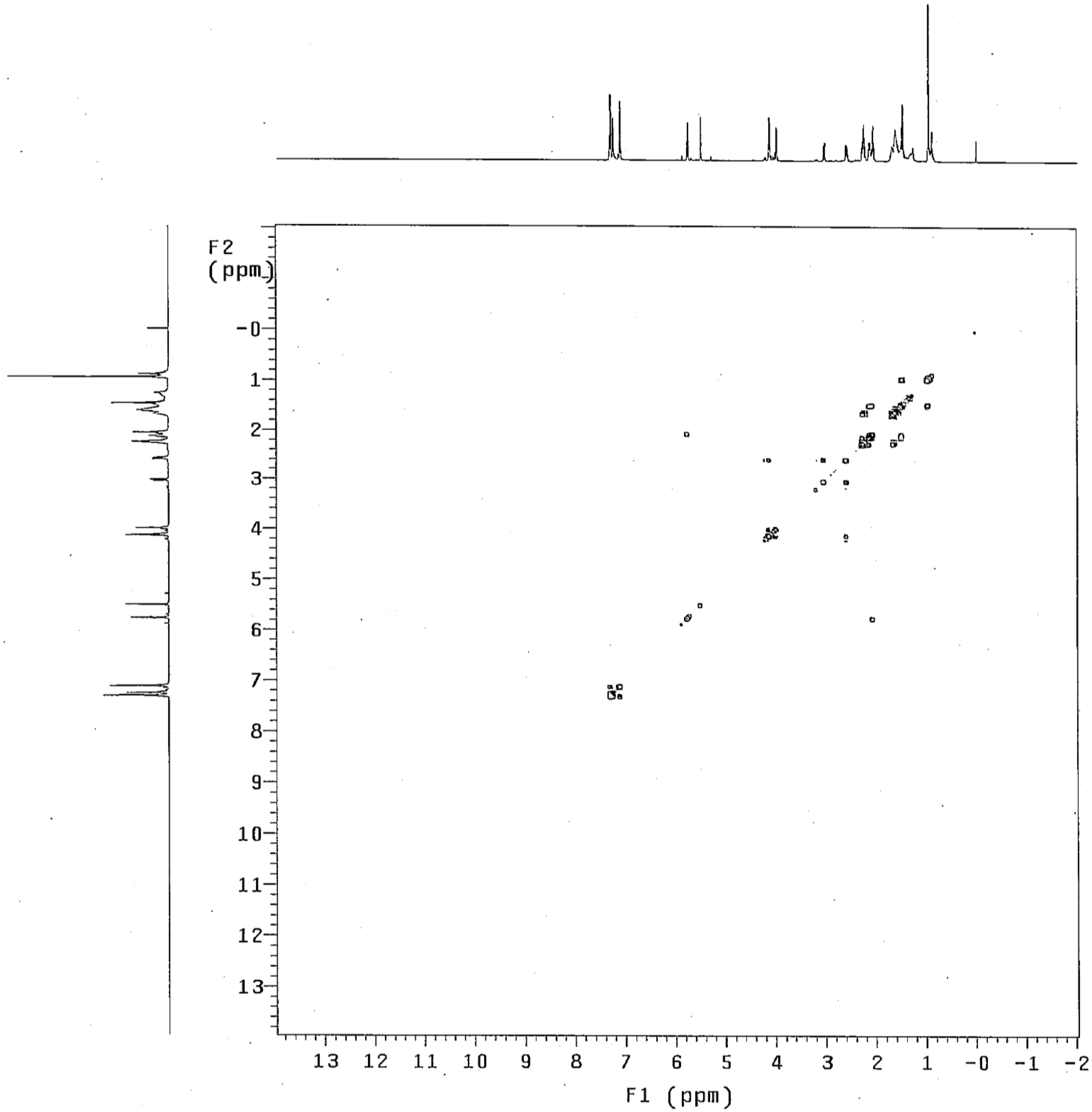

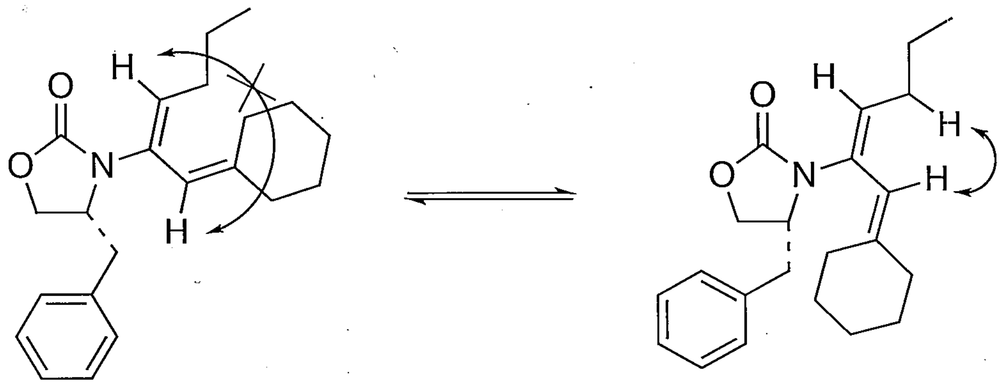

The Key nOe for the 2-Amido-Diene

```
RH-2-140NOESY_29Mar2009

 Archive directory: /export/home/ryuji/vnmrsys/data
 Sample directory: RH-2-140NOESY_29Mar2009

Pulse Sequence: NOESY

 Solvent: CDCl3
 Temp. 25.0 C / 298.1 K
File: NOESY
INOVA-500  "nmr03"

 Relax. delay 1.000 sec
 Mixing 0.200 sec
 Acq. time 0.160 sec
 Width    6387.7 Hz
 2D Width 6387.7 Hz
 4 repetitions
 2 x 128 increments
OBSERVE   H1, 399.7865261 MHz
DATA PROCESSING
 Gauss apodization 0.074 sec
F1 DATA PROCESSING
 Gauss apodization 0.018 sec
FT size 2048 x 2048
Total time 24 min, 21 sec
```

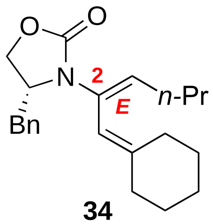

34

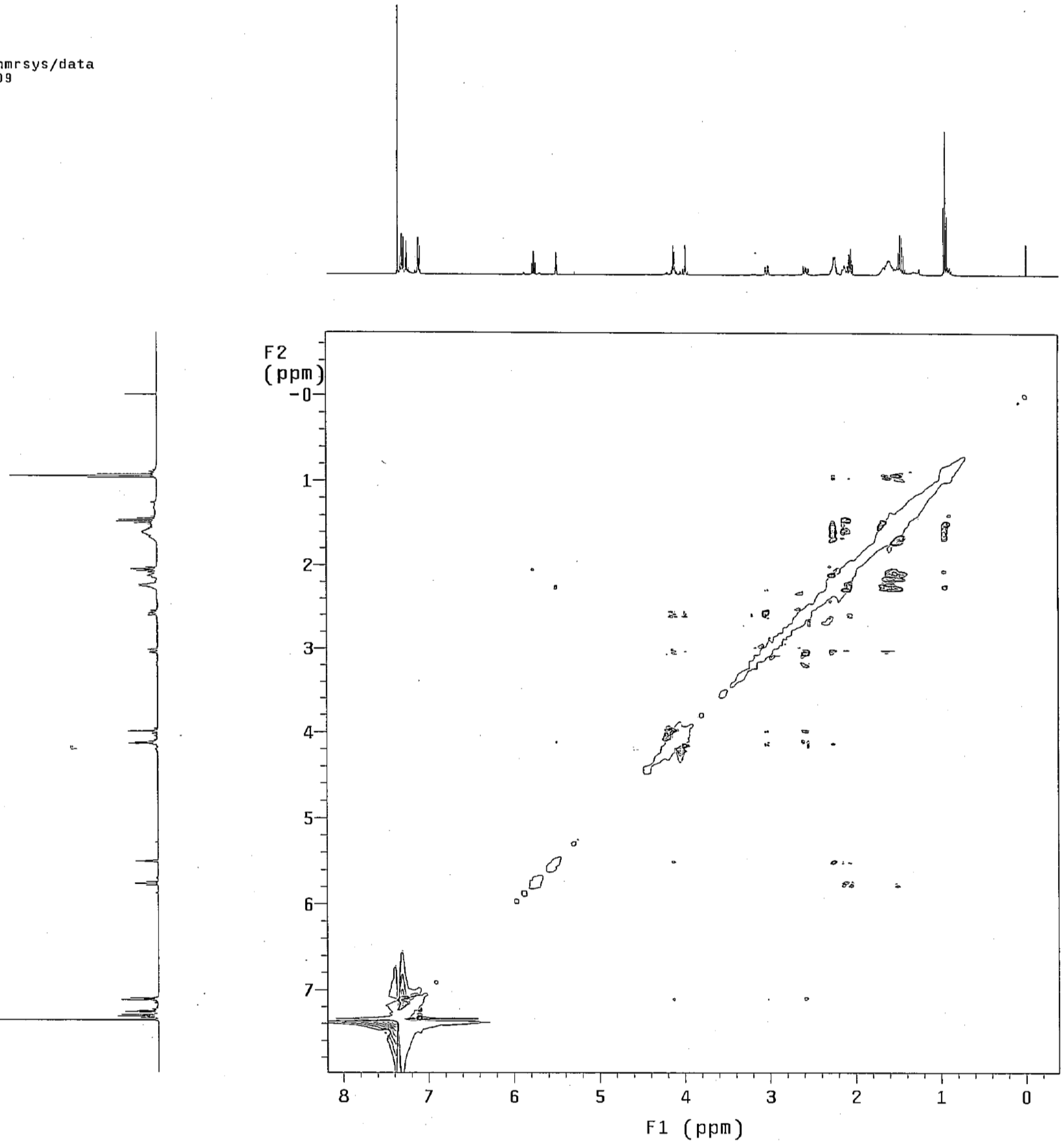

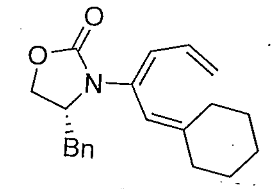

35a

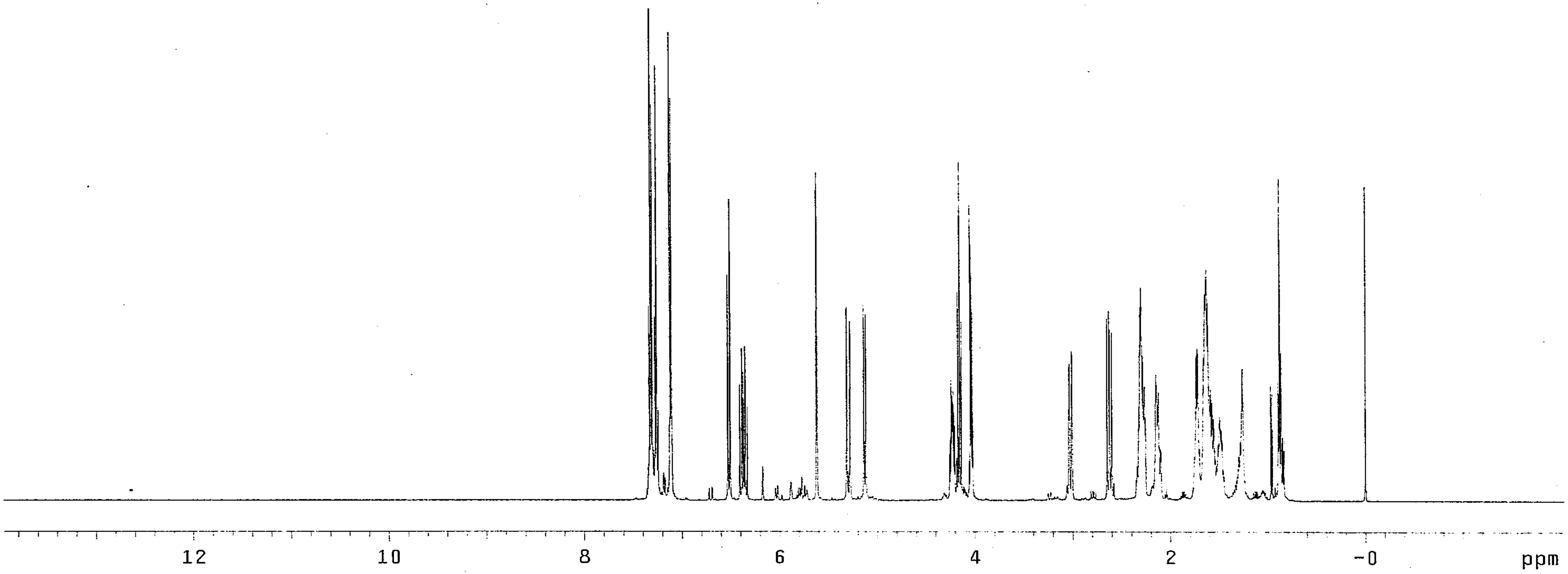

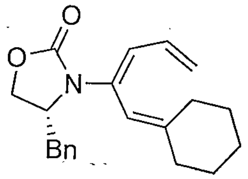

35a

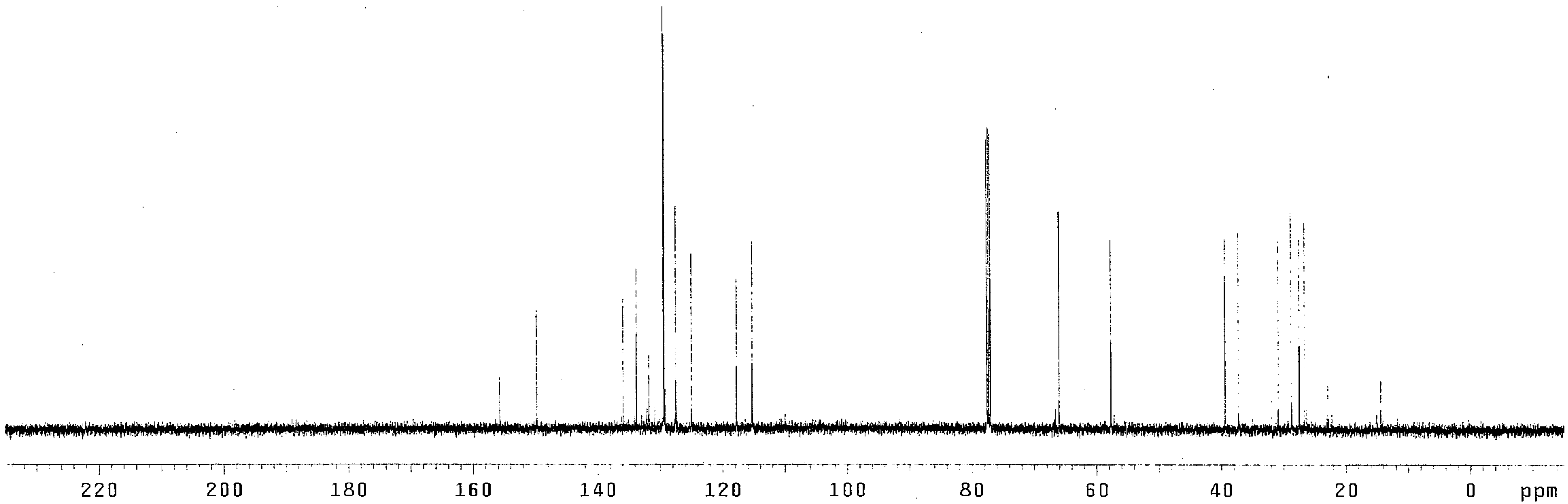

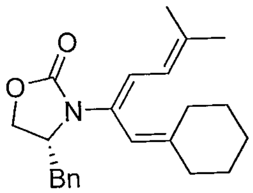

35b

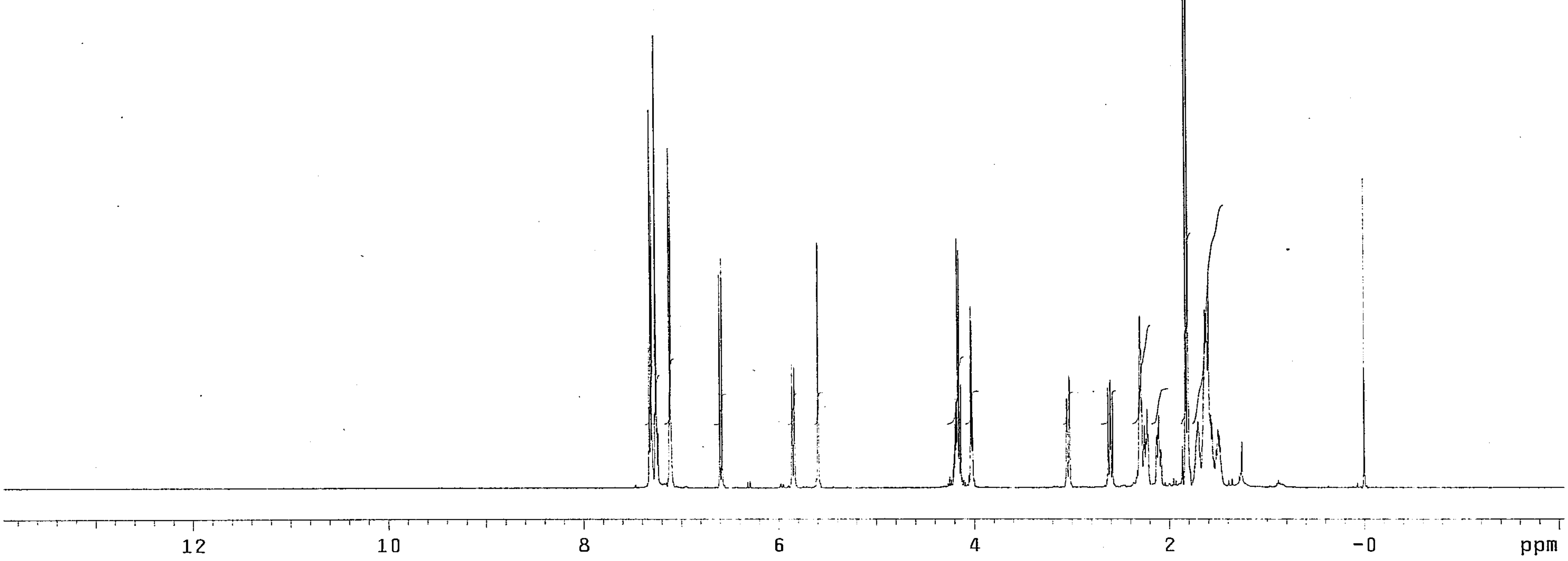

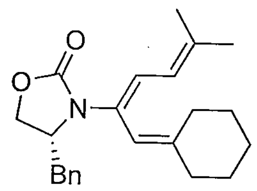

35b

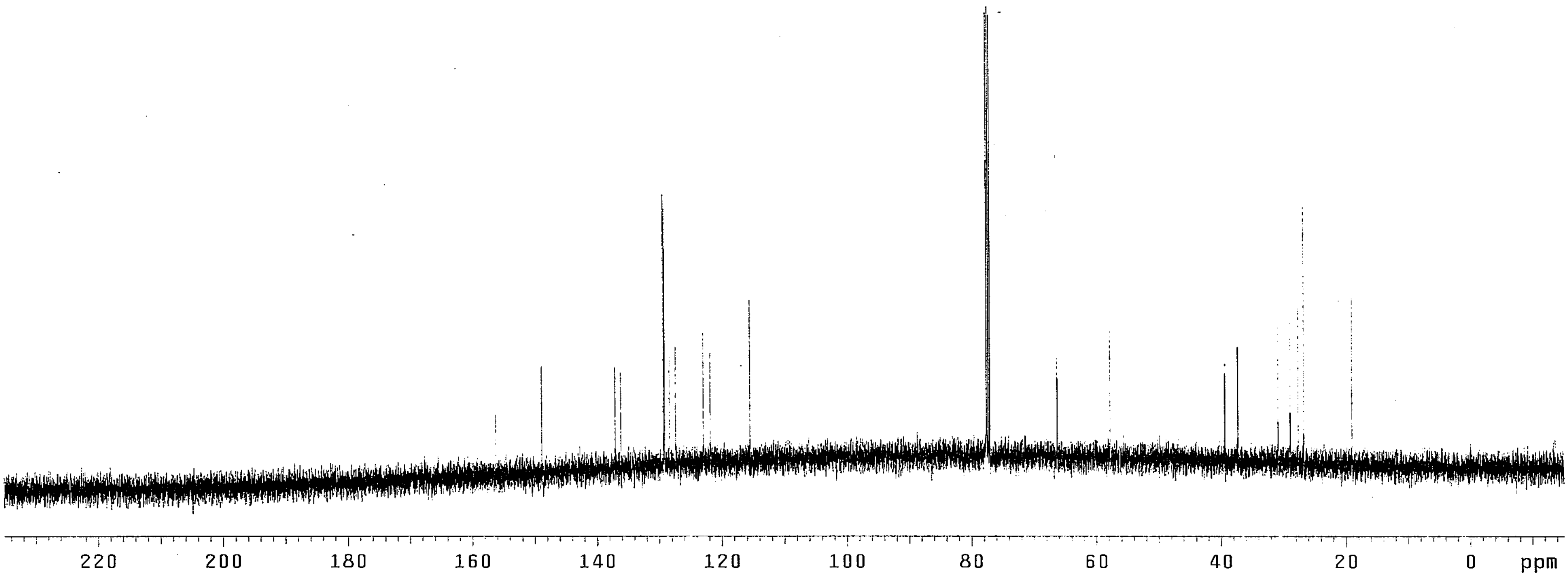

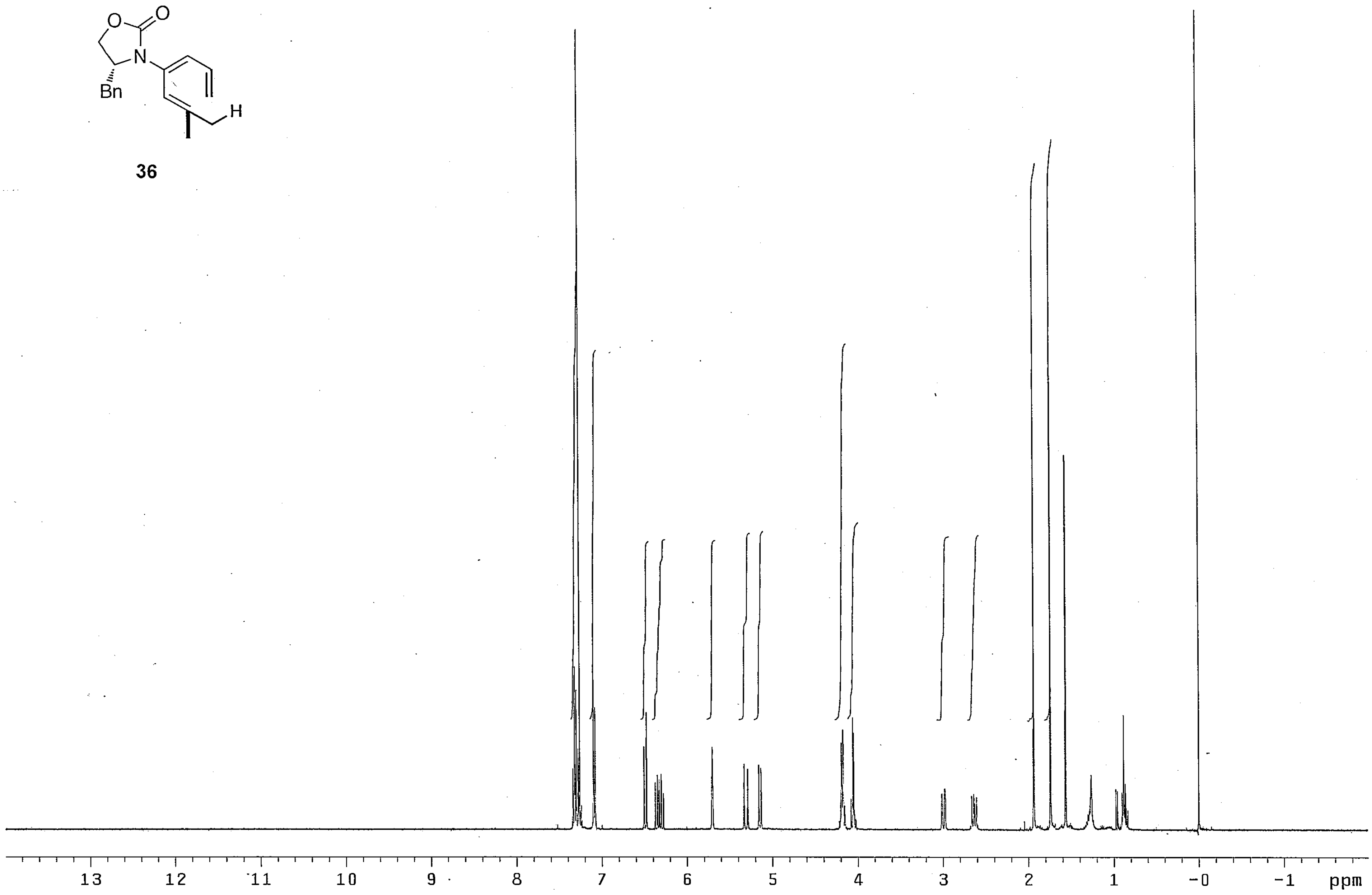
O
O
N
Bn
H
36
13
12
11
10
9
8
7
6
5
4
3
2
1
-0
-1
ppm

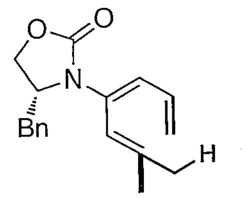

36

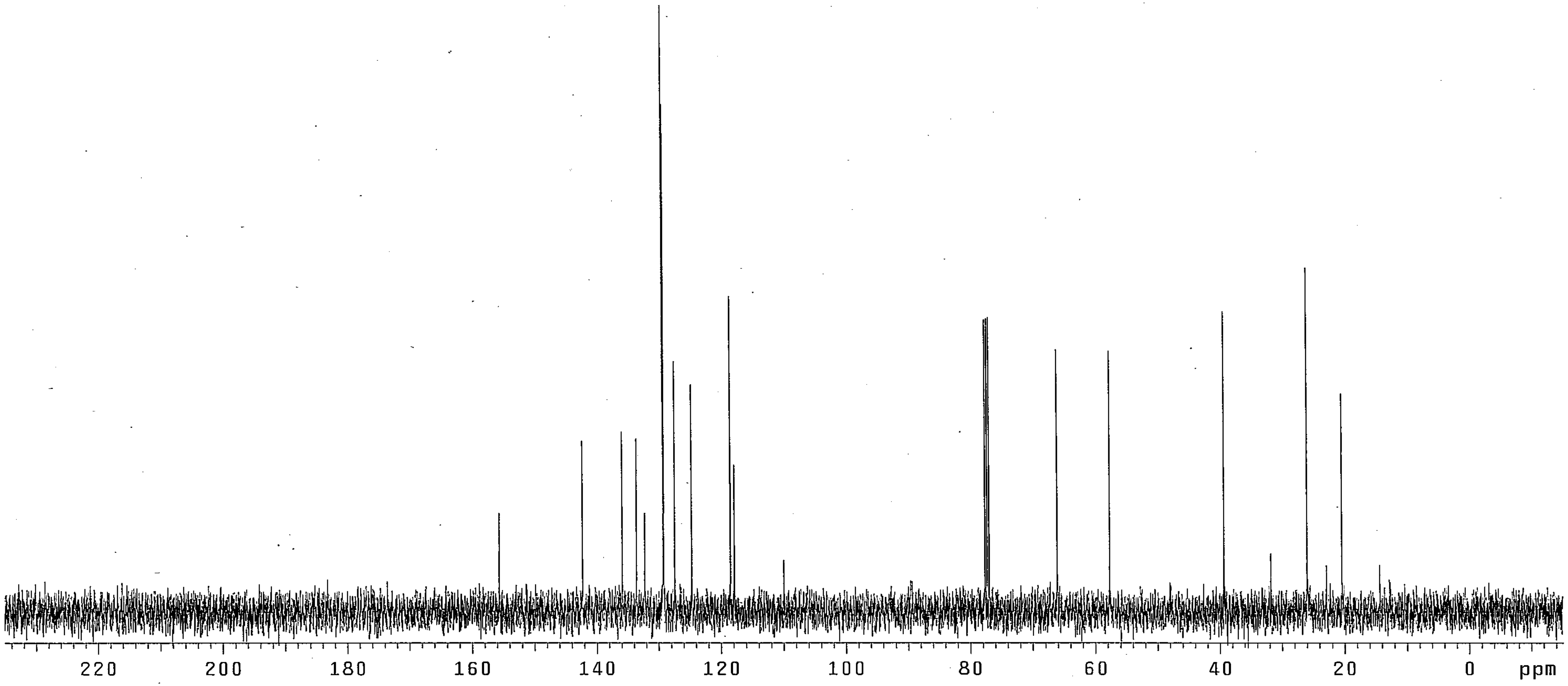

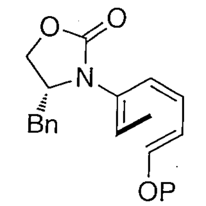

39a: P = TBDPS

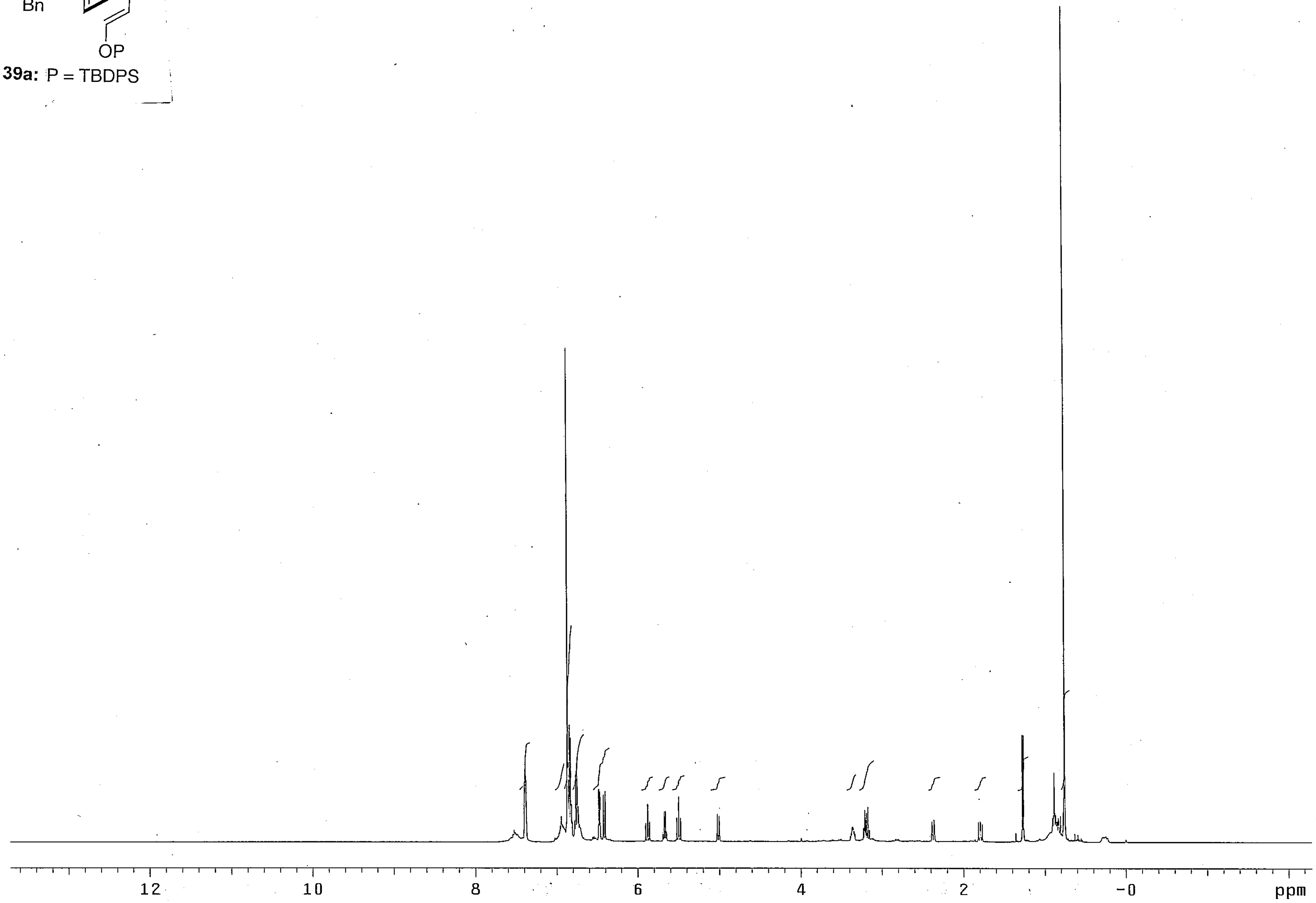

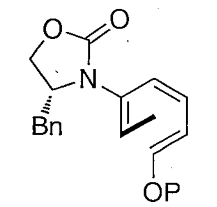

39a: P = TBDPS

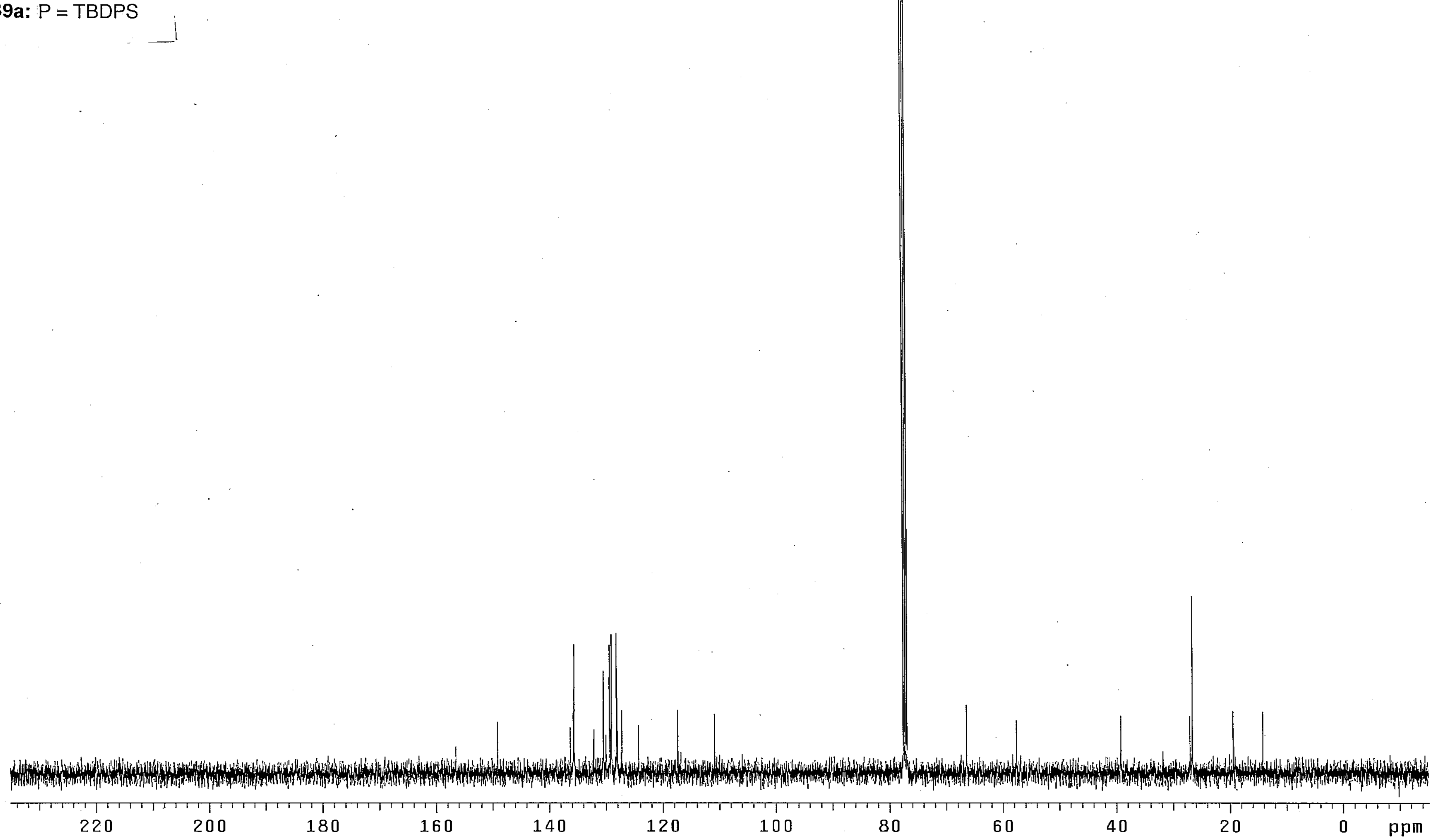

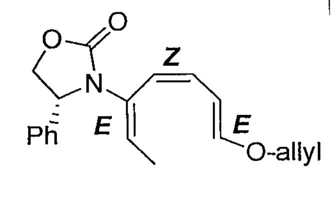

39b

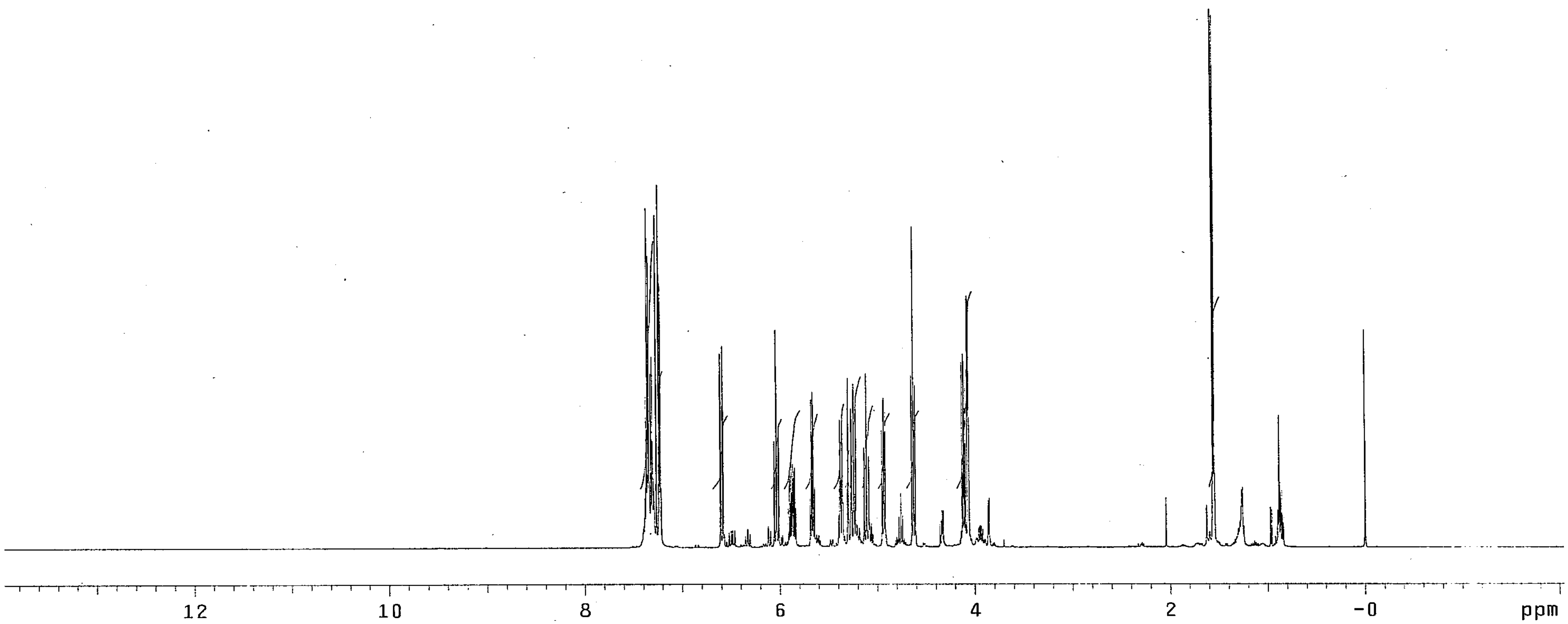

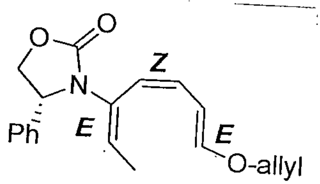

39b

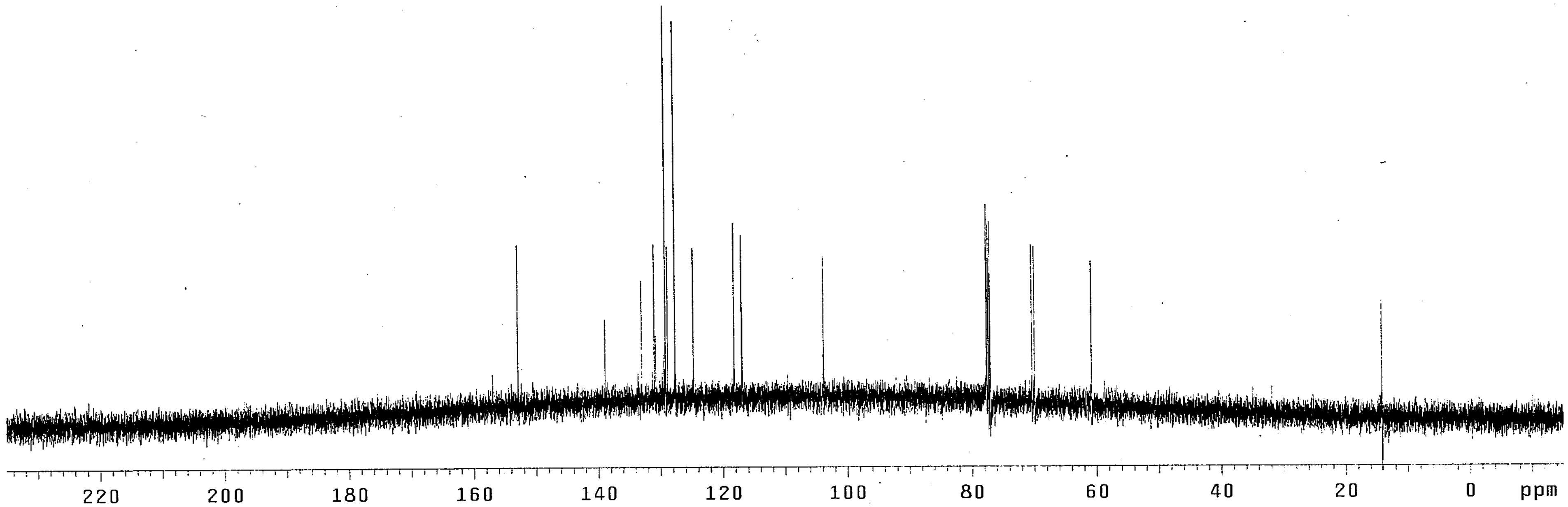

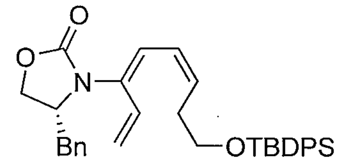

41

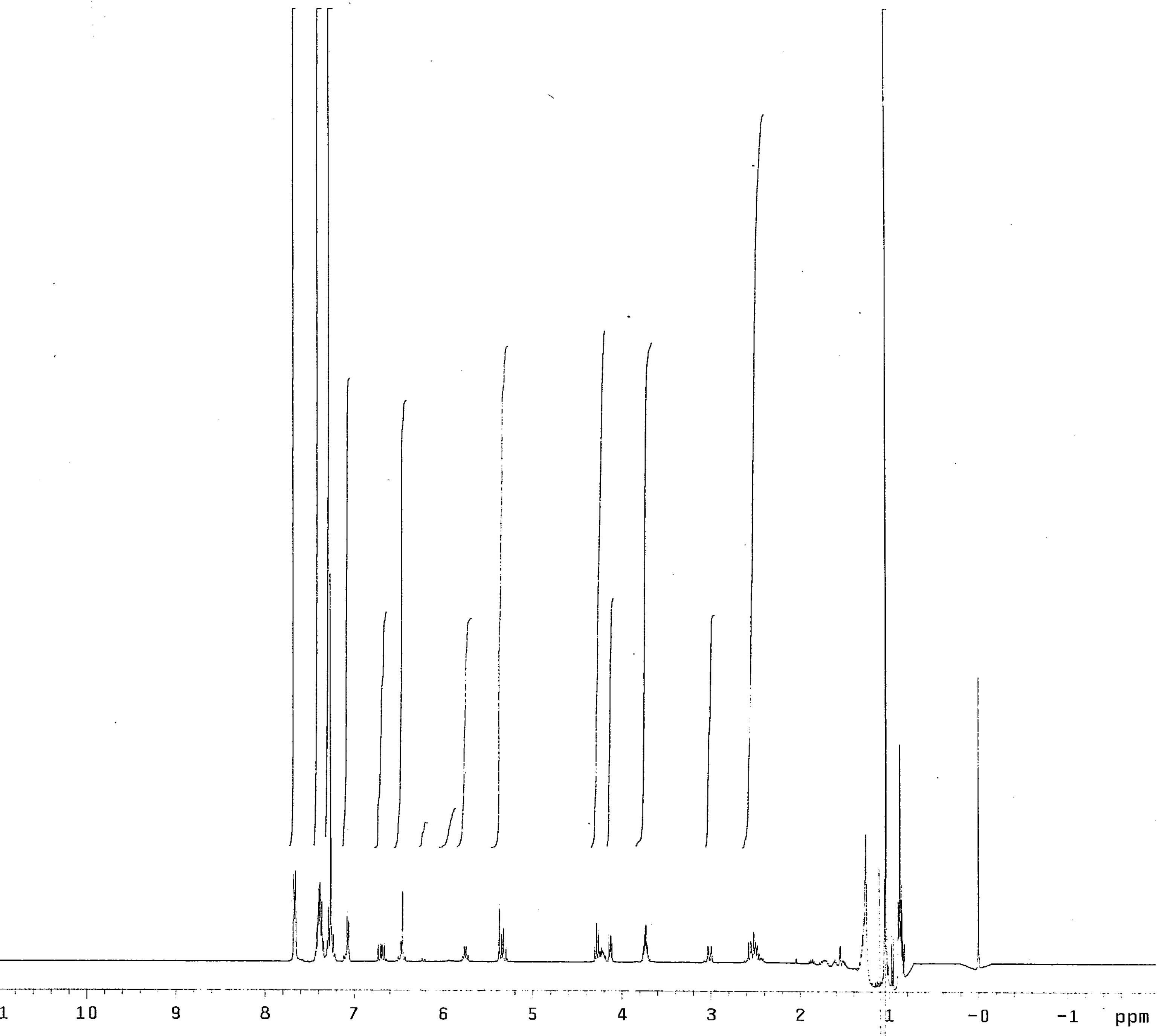

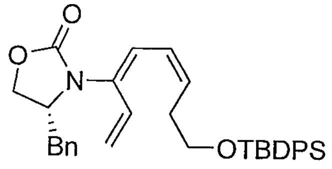

41

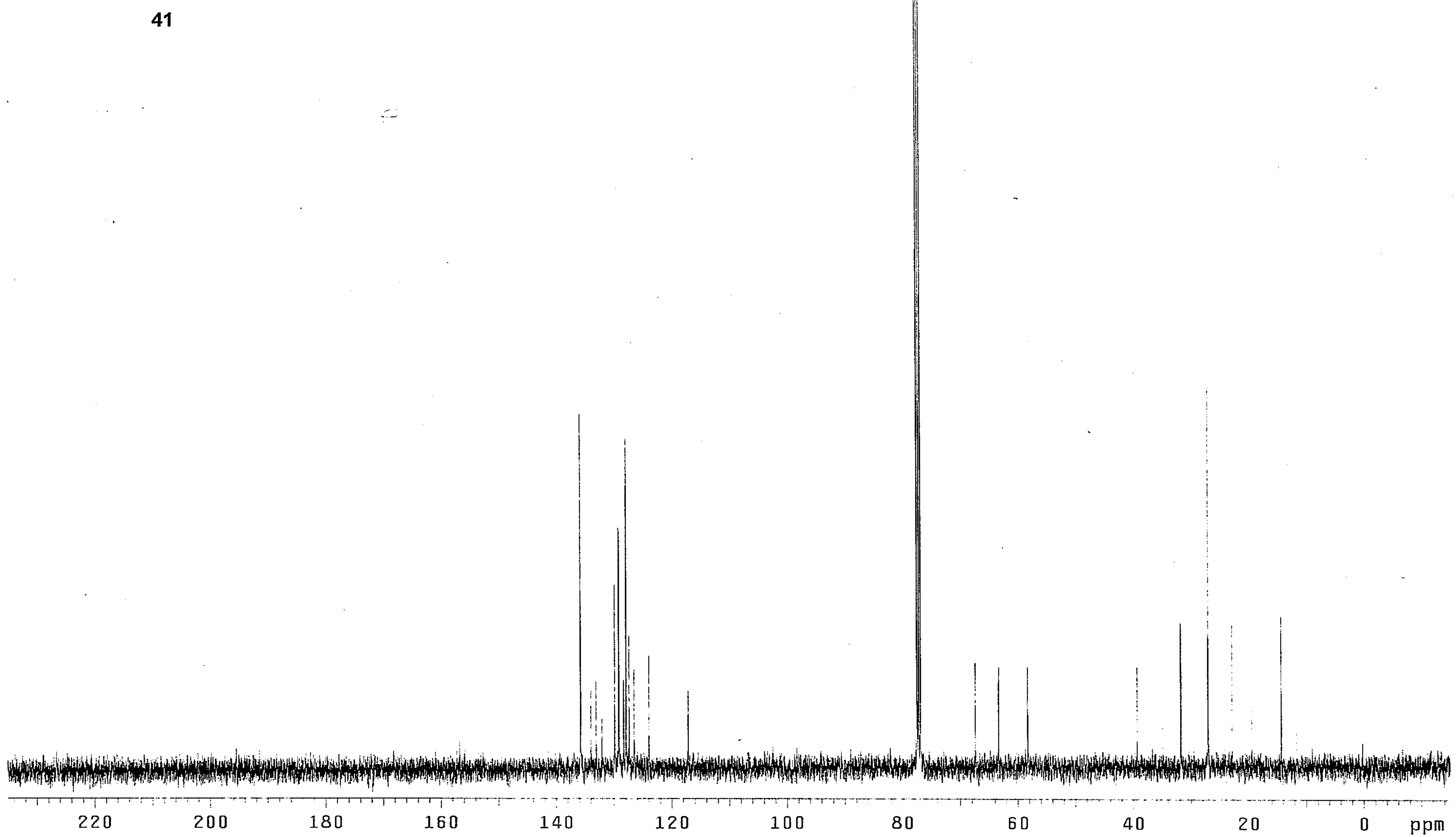

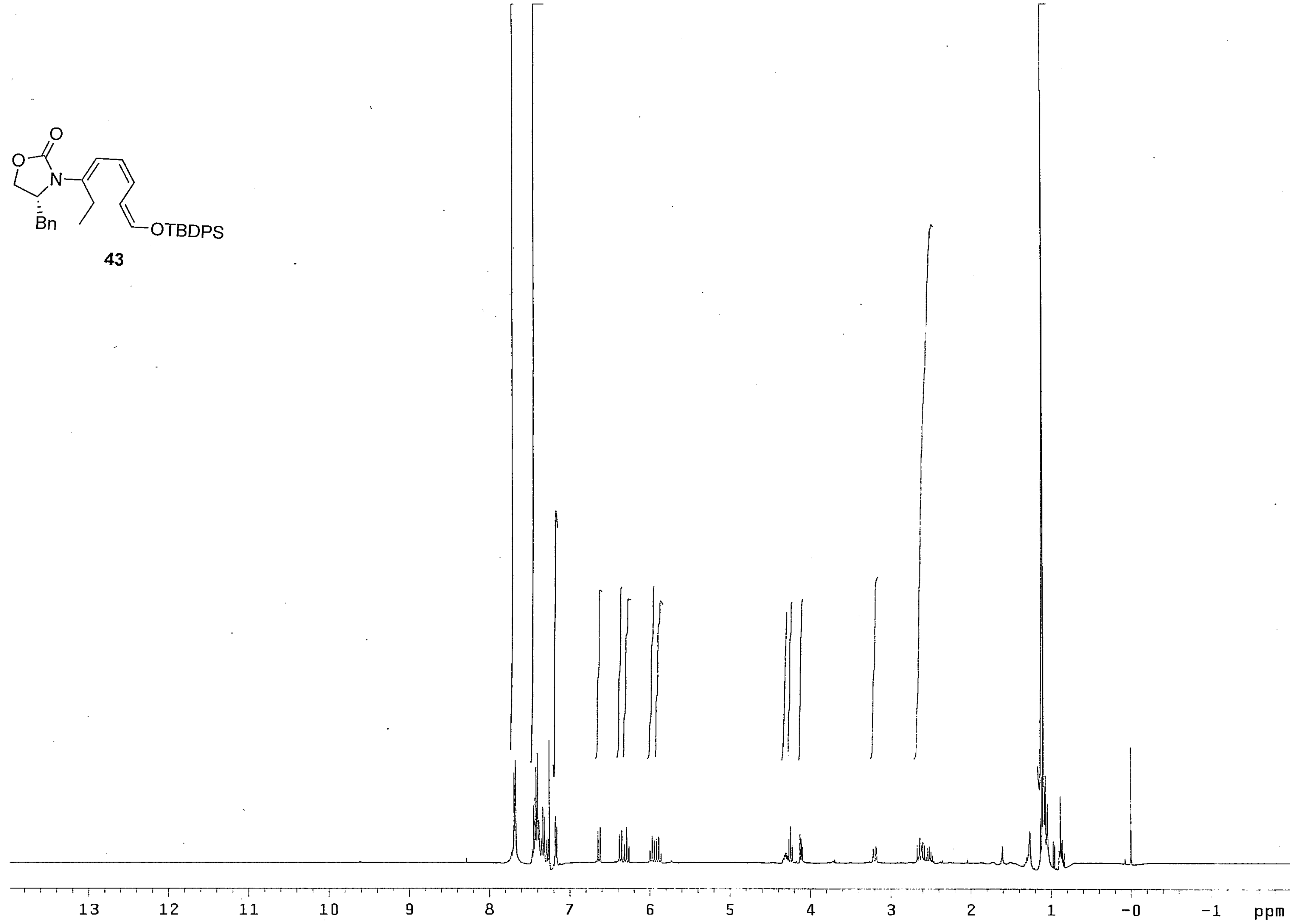

43

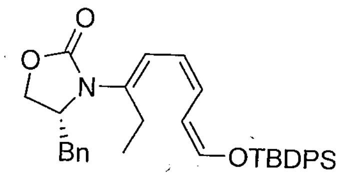

43

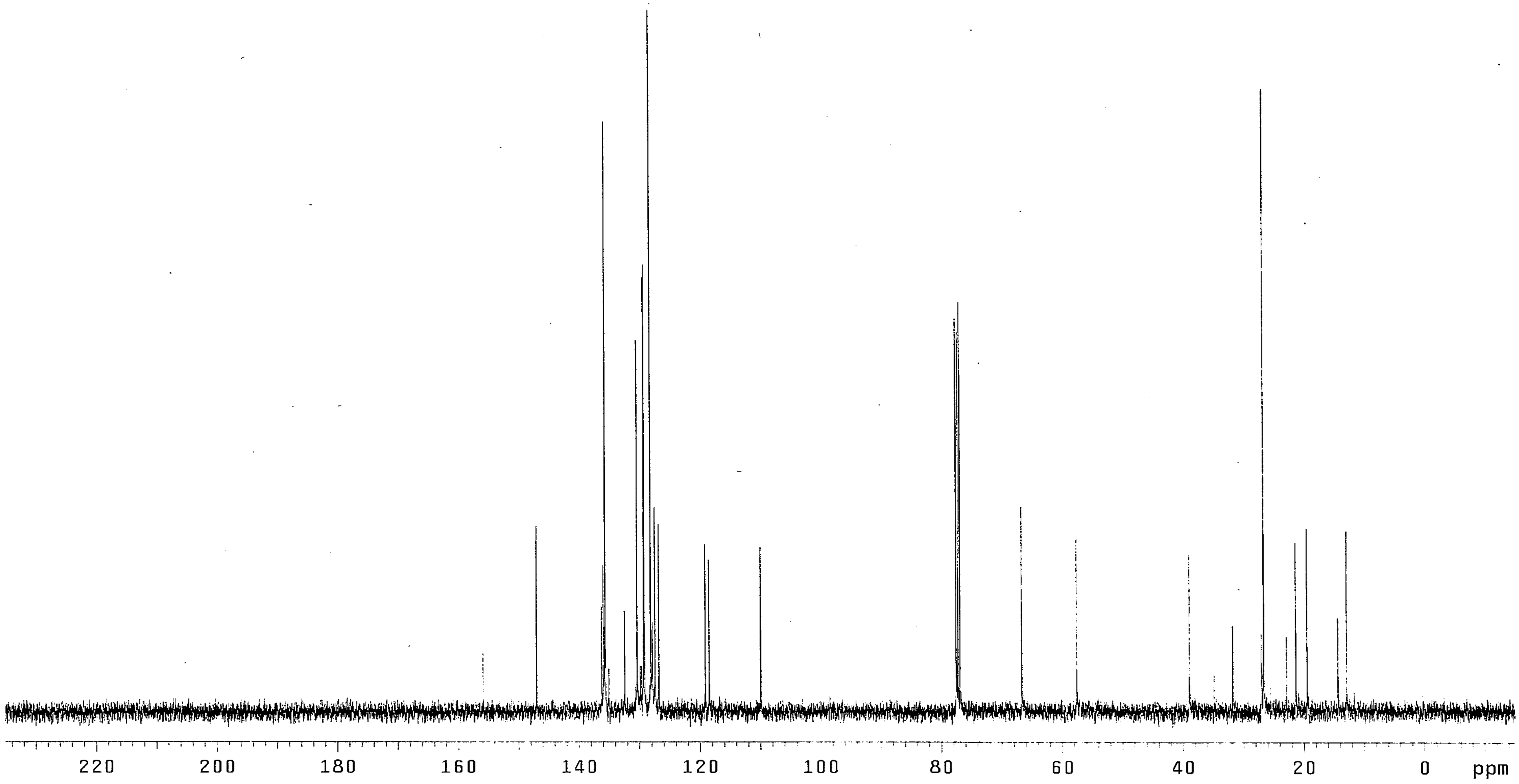

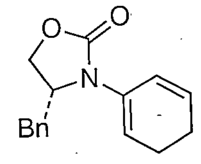

44a

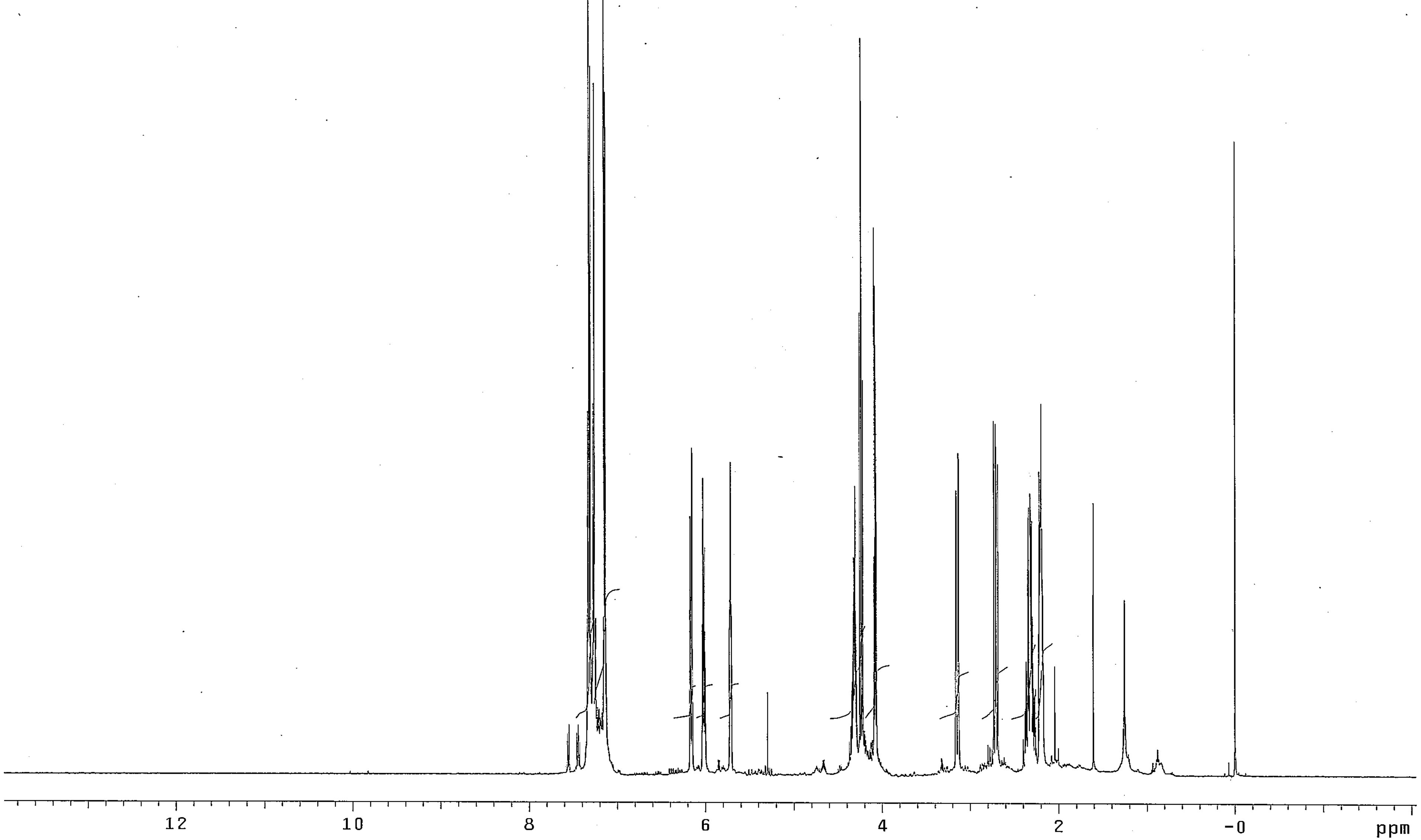

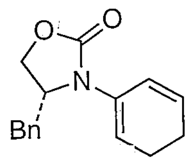

44a

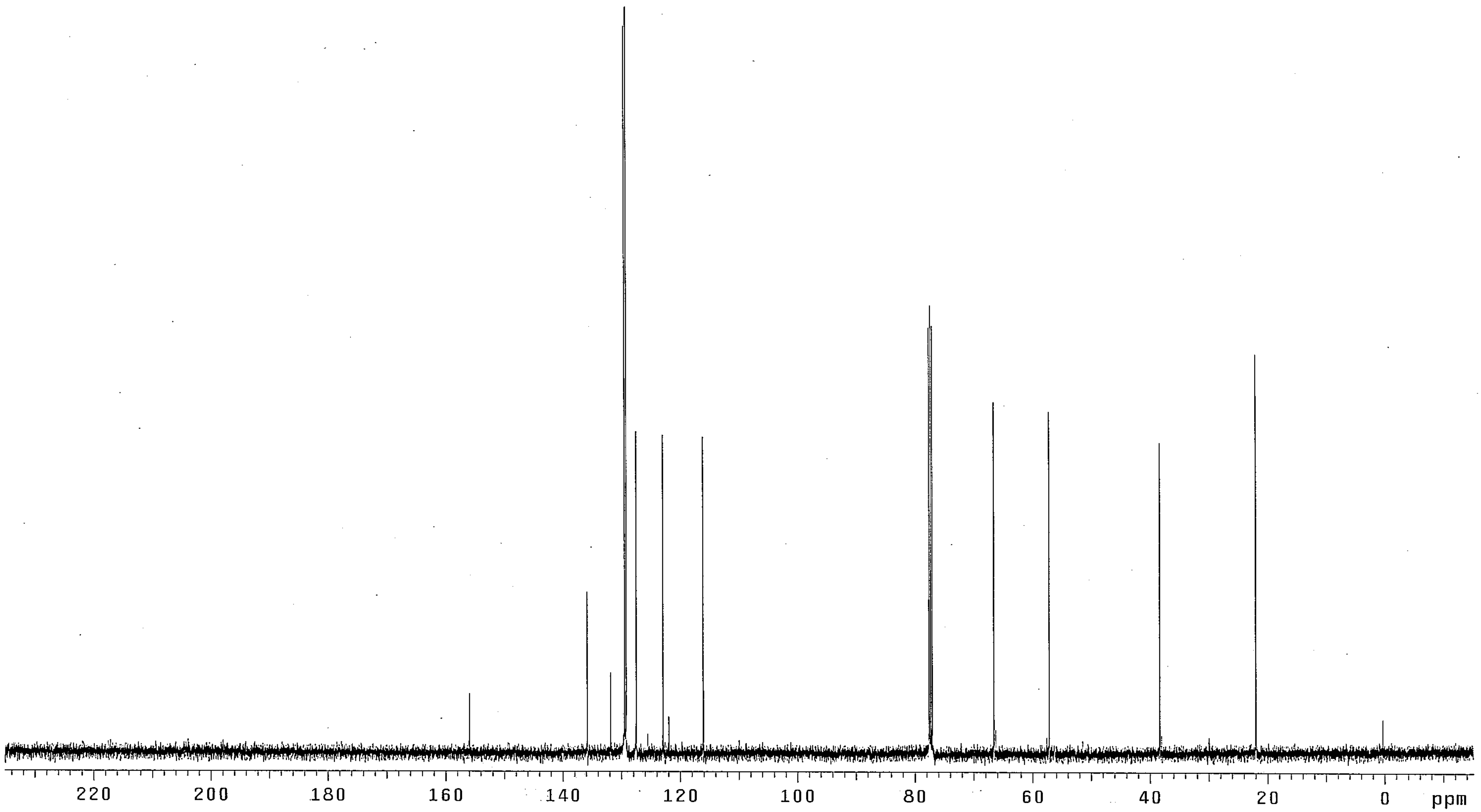

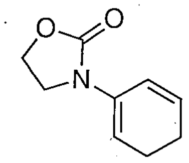

44b

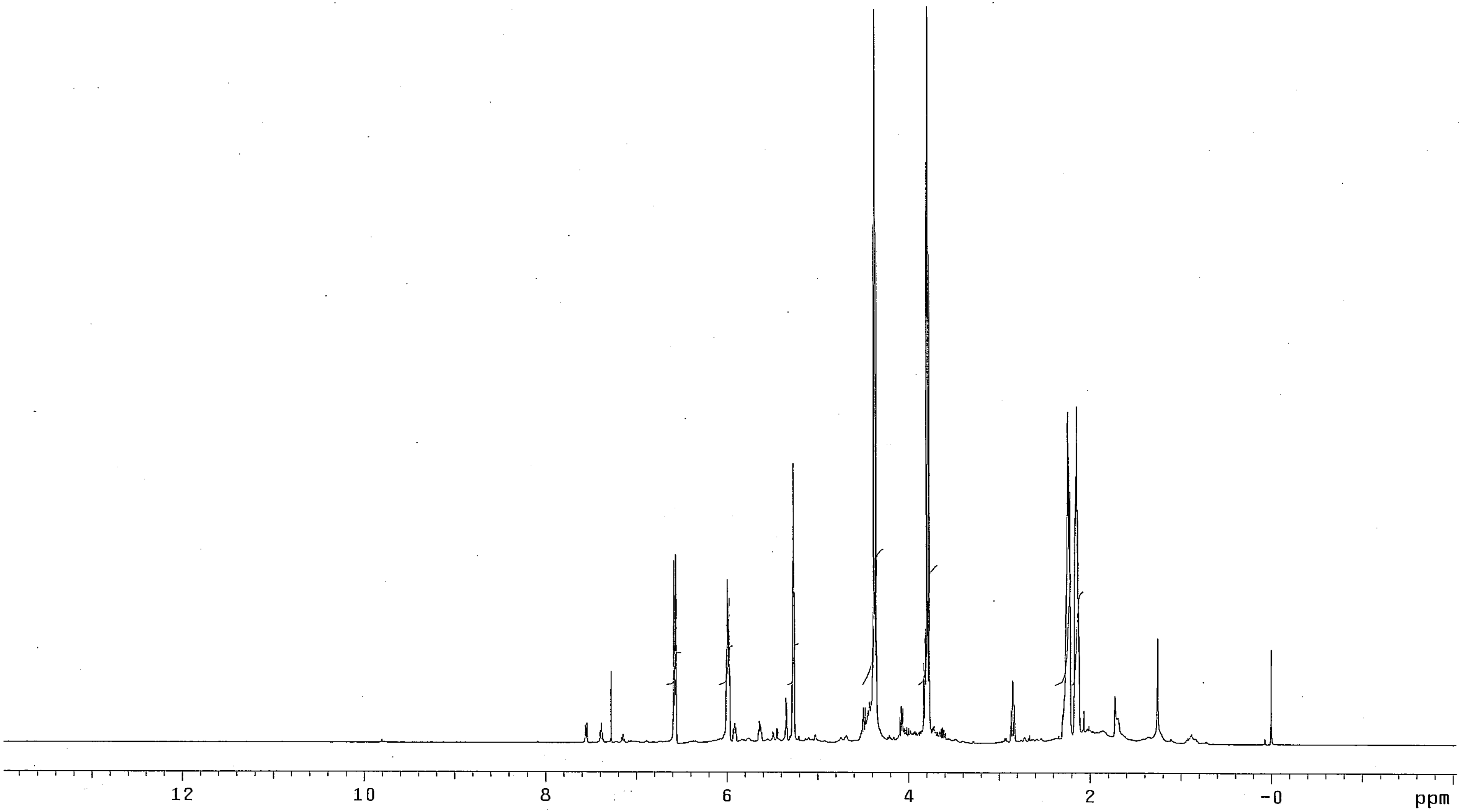

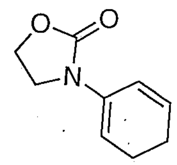

44b

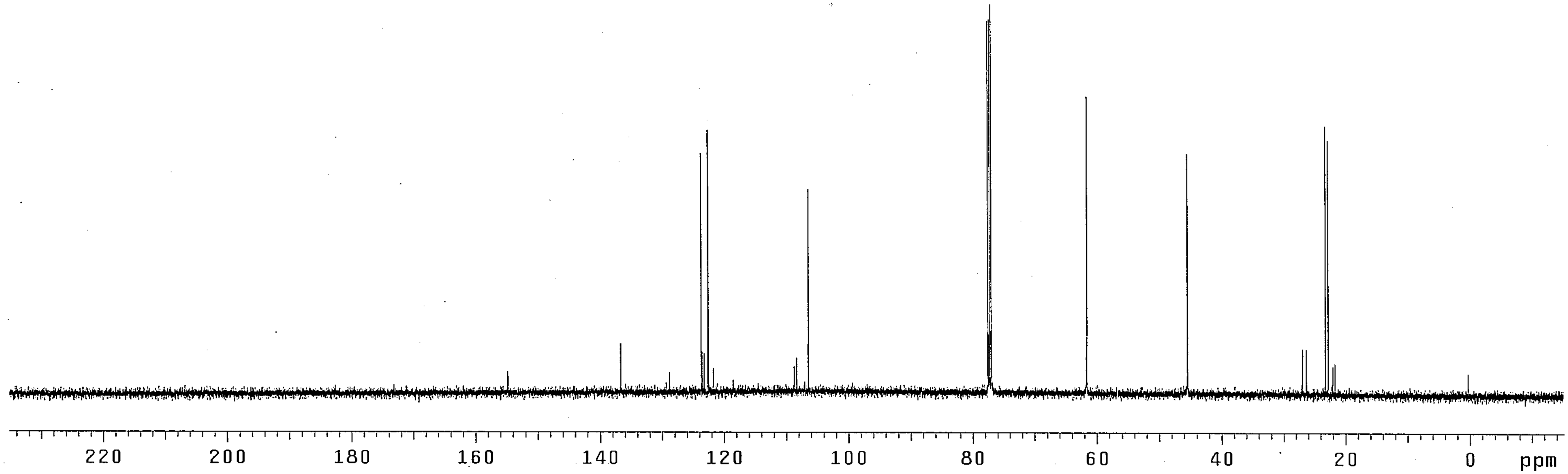

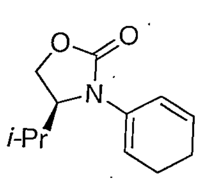

44c

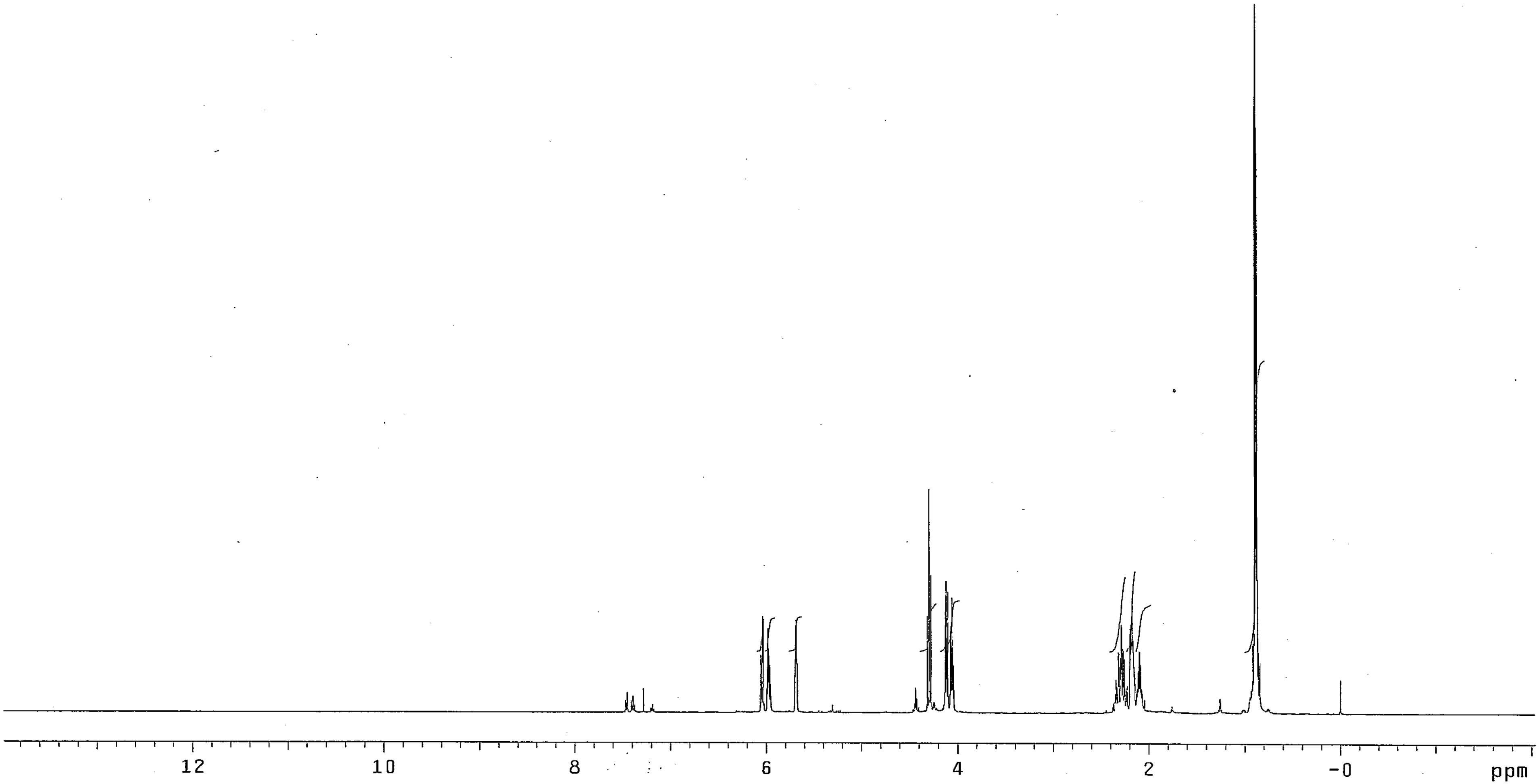

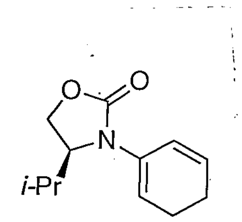

44c

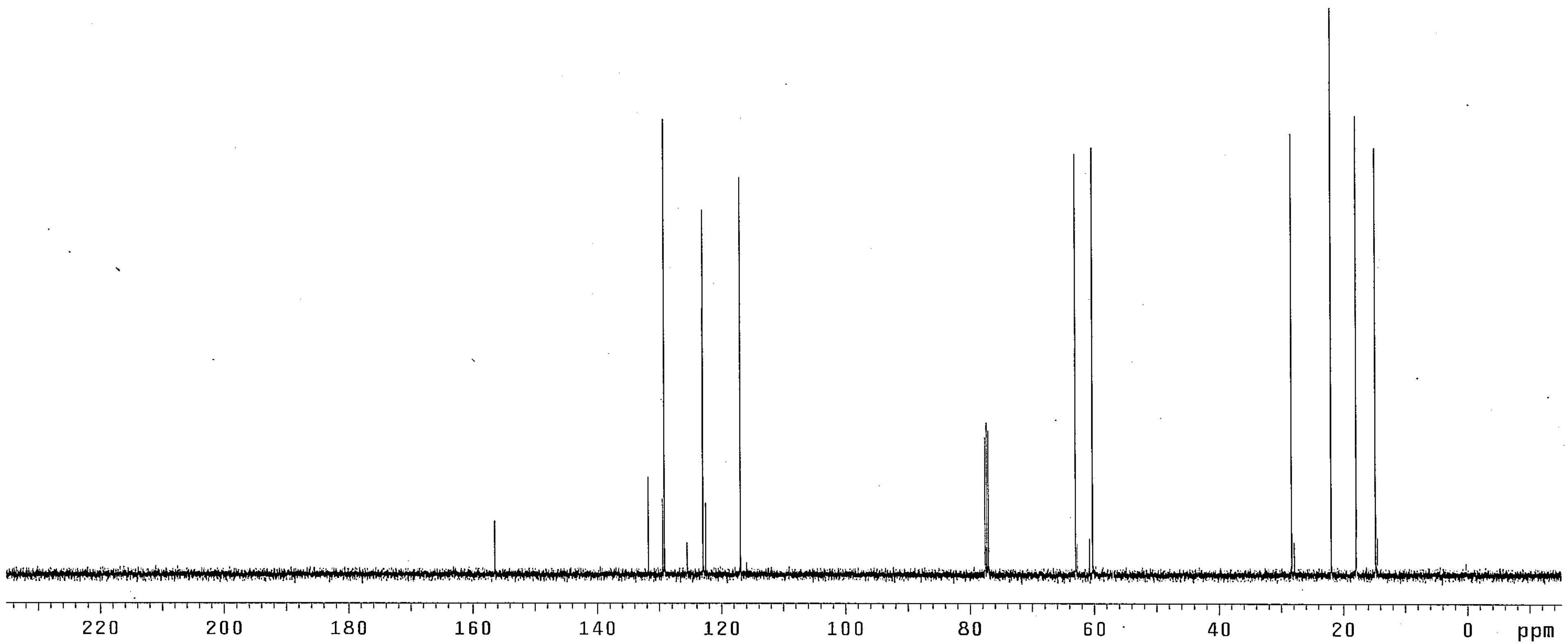

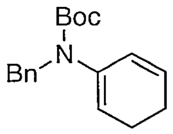

45

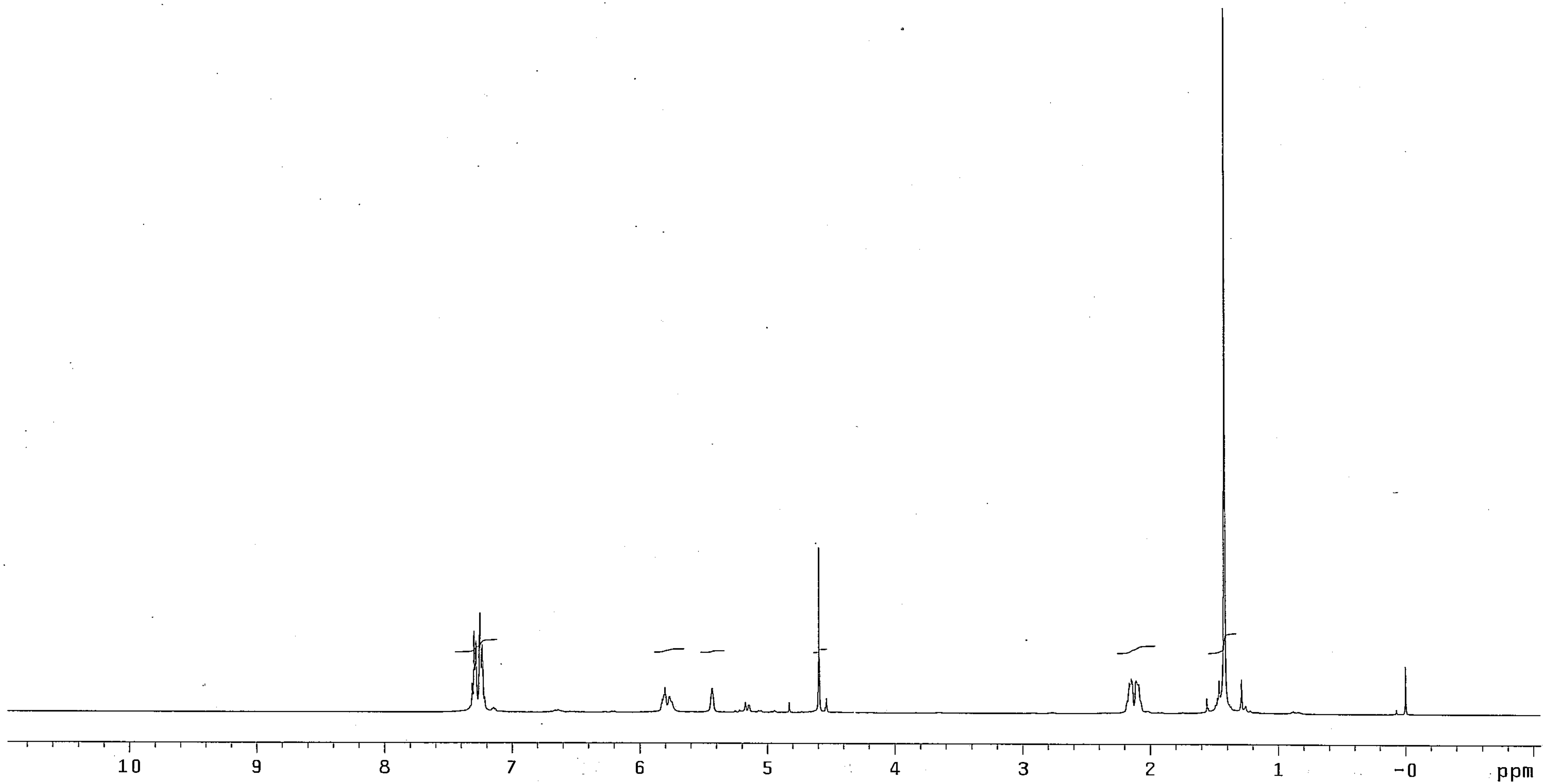

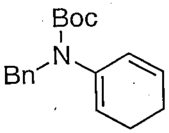

45

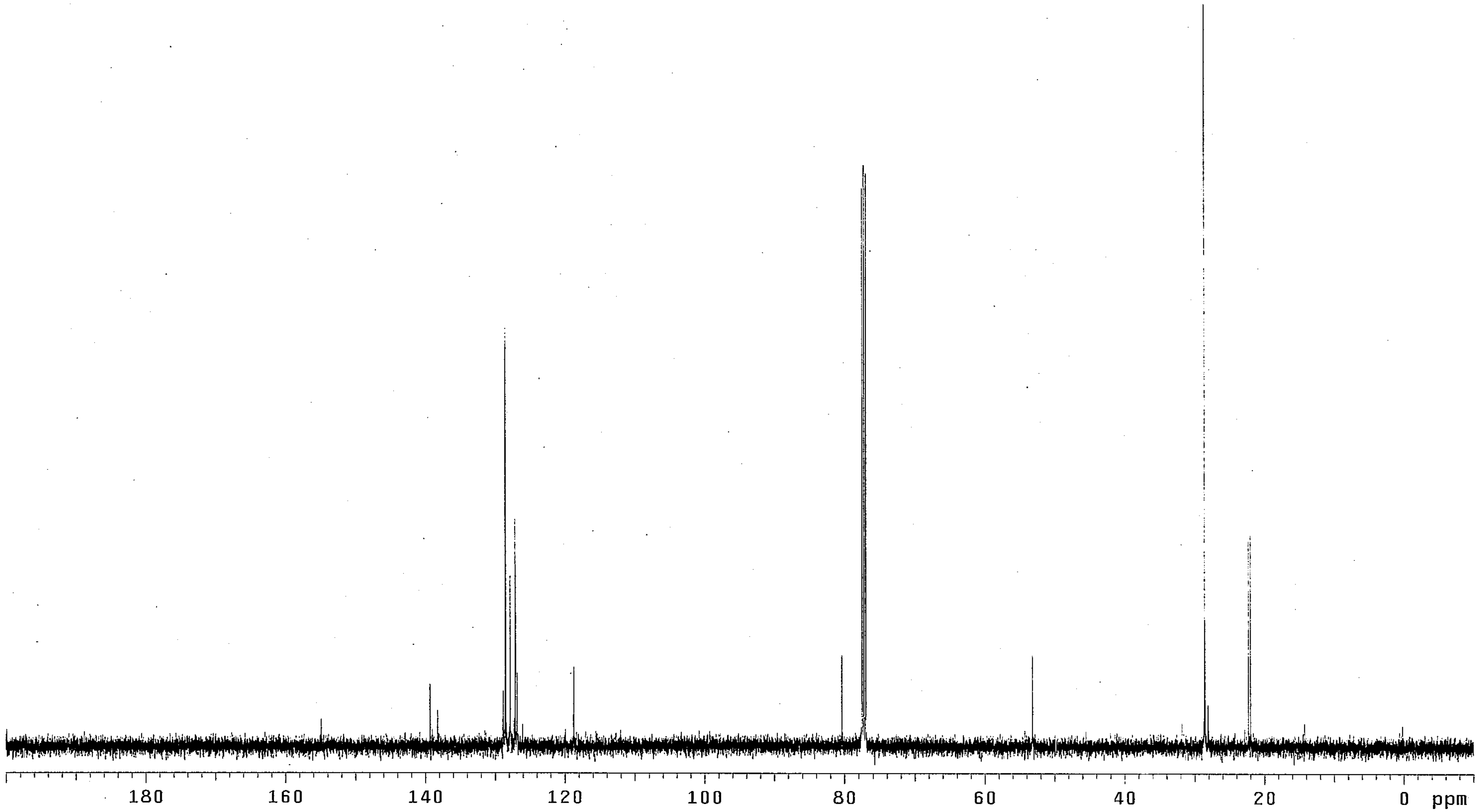

Supplement: File 2 — Proton and Carbon NMR spectra, and NOE data. [file Beilstein_J_Org_Chem-07-410-s002.pdf]
